# Isolation, semi-synthesis, docking-based prediction, and bioassay-based activity of *Dolichandrone spathacea* iridoids: new catalpol derivatives as glucosidase inhibitors†

**DOI:** 10.1039/d1ra00441g

**Published:** 2021-03-23

**Authors:** Tran Thi Phuong Thao, Thanh Q. Bui, Phan Tu Quy, Nguyen Chi Bao, Tran Van Loc, Tran Van Chien, Nguyen Linh Chi, Nguyen Van Tuan, Tran Van Sung, Nguyen Thi Ai Nhung

**Affiliations:** Institute of Chemistry, Vietnam Academy of Science and Technology 18 Hoang Quoc Viet Road, Cau Giay Hanoi Vietnam tranvansungvhh@gmail.com; Graduate University of Science and Technology, Vietnam Academy of Science and Technology 18 Hoang Quoc Viet Road, Cau Giay Hanoi Vietnam; Department of Chemistry, University of Sciences, Hue University Hue City Vietnam ntanhung@hueuni.edu.vn; Department of Natural Sciences & Technology, Tay Nguyen University Buon Ma Thuot Vietnam; Hue University Hue City Vietnam; Asean College of Medicine and Pharmacy Trung Trac street, Van Lam district Hung Yen province Vietnam

## Abstract

*Dolichandrone spathacea* iridoids are promising anti-diabetic inhibitors towards α-glucosidase protein (PDB-3W37) and oligo-1,6-glucosidase protein (PDB-3AJ7). Five catalpol iridoids (1, 2, 10, 13, 14) were isolated from mangrove plant *D. spathacea*, and their derivatives (3, 4, 5, 6, 7, 8, 9, 11, 12, 15) were obtained from reduction, acetylation, *O*-alkylation, acetonisation, or hydrolysation starting from naturally isolated compounds. They were identified by spectral methods such as IR, MS, and 1D and 2D NMR. Their glucosidase-related (3W37 and 3AJ7) inhibitability and physiological compatibility were predicted by molecular docking simulation and prescreened based on Lipinski's rule of five. Experimental α-glucosidase inhibition of 1–15 was evaluated using enzyme assays. Compounds 3, 4, 5, 6, and 9 are new iridoid derivatives, introduced to the literature for the first time, while all fifteen compounds 1–15 are studied for molecular docking for the first time. Regarding protein 3W37, the five strongest predicted inhibitors assemble in the order 2 > 10 > 1 > 9 > 14. In respect to 3AJ7, the corresponding order is 14 > 2 > 10 > 5 > 1 = 9. Lipinski's criteria suggest 10 as the candidate with the most potential for oral administration. The *in vitro* bioassay revealed that compound 10 is the most effective inhibitor with a respective IC_50_ value of 0.05 μM, in the order 10 > 2 > 14 > 13 > 1. The computational and experimental results show good consistency. The study opens an alternative approach for diabetes treatment based on inhibitability of natural and semi-synthesised catalpol iridoid derivatives towards carbohydrate-hydrolases.

## Abstract 2

*Dolichandrone spathacea* catalpol derivatives as glucosidase inhibitors: *in silico* and *in vitro* research.

## Graphical abstract

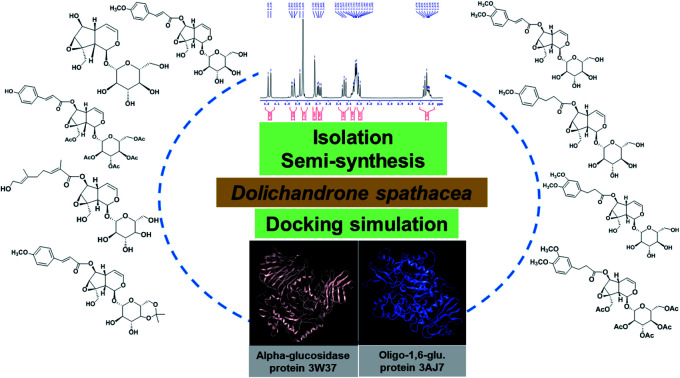

## 1. Introduction

Diabetes mellitus (DM) has become prevalent in the past decades, especially in the middle-income countries. According to the International Diabetes Federation, it was responsible for about 4.2 million deaths in 2019^1^ and is predicted to be the seventh leading cause of death by 2030 (WHO).^2^ Also, there is evidence suggesting a relationship between this disease and the causes of cardiovascular diseases, blindness, kidney failure, stroke, and limb amputation.^3^ In particular, type 2 DM is a noninsulin-dependent disorder, stemmed from the ineffective use of insulin in the body and accounting for 90–95% of total diabetes cases.^4^

**Fig. 1:**
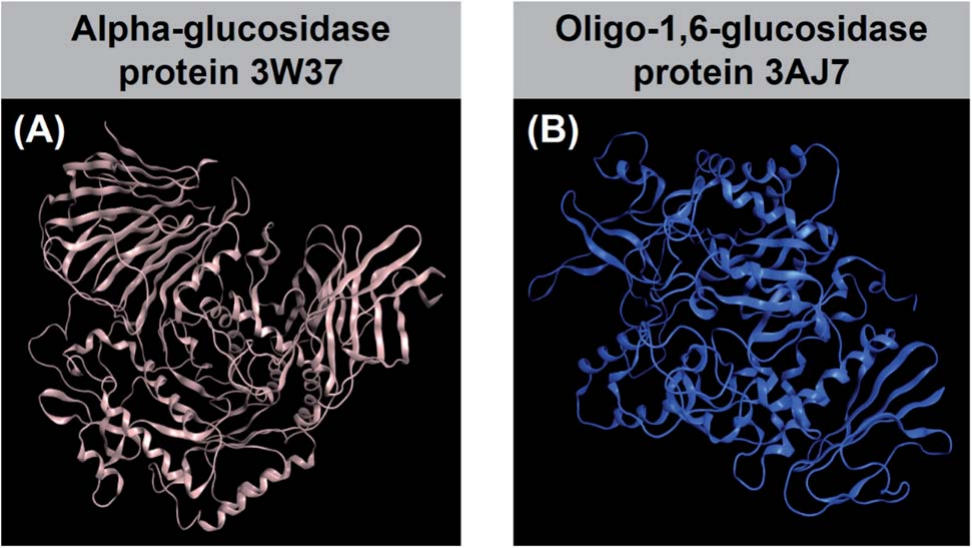
(A) α-Glucosidase protein 3W37 (DOI: 10.2210/pdb3W37/pdb) and (B) oligo-1,6-glucosidase protein 3AJ7 (DOI: 10.2210/pdb3AJ7/pdb) referenced from Worldwide Protein Data Bank.

There are many kinds of medicines for the treatment of type-2 diabetes, based on insulin sensitizers, sodium glucose transporter-2 (SGLT-2) inhibitors, glucagon like peptidase-1 (GLP-1) analogues and dipeptidyl peptidase-4 (DPP-IV) inhibitor mechanisms. However, these therapies have significant side effects such as weight gain, cardiovascular risks, and atherosclerosis as well as limited tolerability. Therefore, the approach for type 2 DM changes into the inhibition of glucosidase enzymes, which slows down of glucose absorption through intestinal wall. There are two main types of glucosidase responsible for carbohydrate break-down processes in the body, regarding their sources: α-glucosidase and oligo-1,6-glucosidase. First, α-glucosidase is an exoenzyme found in animal, plant, bacterial, or fungal organisms,^5^ especially from sugar beet seeds.^6^ This type of glucosidase was demonstrated only catalysing hydrolysis of α-(1→4) and α-(1→6) bonds to yield monosaccharides.^7^ Protein structural data of α-glucosidase can be referenced at Worldwide Protein Data Bank under entry PDB-3W37 (DOI: 10.2210/pdb3W37/pdb). Second, oligo-1,6-glucosidase, often called isomaltase, is a debranching endoenzyme, hydrolysing only the α-1,6 linkage in starch and glycogen to produce sugars with an α-configuration.^8^ Unlike α-glucosidase, they are not responsible for α-1,4 linkage hydrolysis. Oligo-1,6-glucosidase is mainly found in animals although some bacterial species, such as *Bacillus cereus*, can also synthesise it.^9^ In humans, it is located on the small intestine brush border.^10^ Information for isomaltase crystal structure can be downloaded directly from Worldwide Protein Data Bank database under entry PDB-3AJ7 (DOI: 10.2210/pdb3AJ7/pdb). Therefore, 3W37 and 3AJ7 are considered as highly promising drug targets for effective treatment of type-2 diabetes, especially medication through oral administration. In principle, multiple inhibition of these enzymes (3W37 and 3AJ7) is considered as an efficacious strategy to suppress the hyperglycemia and improve insulin sensitisation, simultaneously. Crystal structure of the proteins are shown in Fig. 1.

*In silico* technique, based on computational simulation and computing calculation, is currently seeing a gain of their popularity for medical science as prescreening research. It is for reducing cost and time of wet laboratory experiments by predicting the compounds with undesirable properties and the most promising candidates. The former substances are deemed to be eliminated from the next analysis or further-developed research, while the latter justifies the selection.^11^ Regarding ligand–protein interaction, molecular docking simulation is an effective method to investigate the potency of a ligand as an inhibitor towards its targeted protein. The method can estimate ligand-target binding energy and intermolecular interaction, thus predicting static stability of the inhibitory systems.^12^ Effectively inhibited by external ligands, the enzyme is likely to undergo conformational changes, thus loss of enzymatic functionality inevitably ensuing. This might help mitigating the amount of glucose catalytically synthesised and excreted to bloodstream. According to the methodology of molecular docking simulation applied for ligand–protein inhibitory systems in general and to the evaluation from.

Molecular Operating Environment (MOE)-based algorithms in particular, an associated docking score (DS) is considered as the main indicator for inhibitory effects, of which value lower than −3.2 kcal mol^−1^ indicates good binding capacity.^13–15^ In principle, the figure is yielded by the free-energy sum of all individual intermolecular interactions, of which affinity stems from hydrophilic bonding, *i.e.* various hydrogen-bond types, and hydrophobic binding, *i.e.* van der Waals forces. Besides, a root-mean-square deviation (RMSD) value over 3 Å reveals that the inhibition expects failure; meanwhile, the threshold of docking success is widely acceptable if ≤2 Å.^16^ Also, visual illustration for inhibitory morphology and interaction description is often provided. Specification to drug design for diabetes, molecular docking technique demonstrated its contributive significance regarding a variety of ligand families, *e.g.* thiazolidinedione,^17^ pyrazole–rhodanine,^18^ or wedelolactone.^19^

Catalpol and catalpol analogues, containing epoxy ring at C-7 and C-8, are widely found in many plants belonging to some families such as Plantaginaceae, Lamiaceae, Bignonicaceae.^20^ Recently, there is an increasing numbers of papers reported about the anti-diabetic efficacy of catalpol derivatives in the management of diabetes and its complications.^20,21^ It has been showed that catalpol-type iridoids are able to restore diabetic liver function by improving glucose and lipid metabolism, ameliorating oxidative stress, and restoring mitochondrial function. They improve glucose uptake and lipolysis by upregulating the expression of glucose transporter as well as downregulating the expressions of peroxisome proliferators in mice.^20^ Therefore, catalpol derivatives have become a very promising scaffold for the development of anti-diabetic drug candidates. Our previous study on chemical constituents of the mangrove plant *Dolichandrone spathacea* led to the isolation of four catalpol iridoids.^22^ This seems reasonable because according to folk experiences, the plant is currently reported as a natural symptom reliever for diabetes, especially in Mekong Delta, Vietnam. Nevertheless, its chemical properties and medical potentiality still remain almost untouched possibly because of their narrow distribution.

**Fig. 2:**
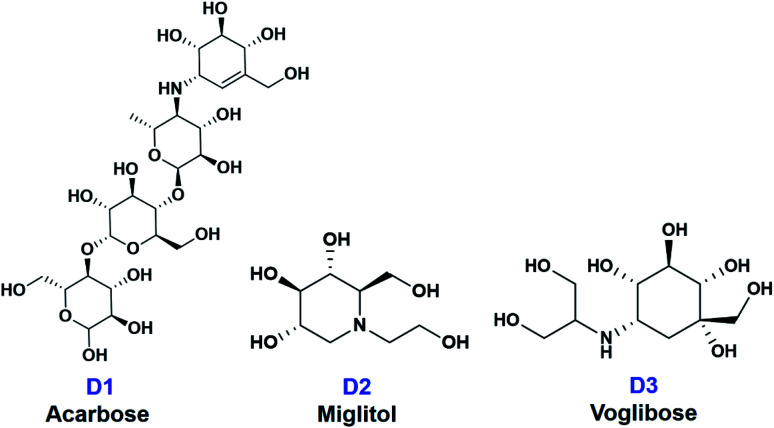
Structural formula of commercial medicines for diabetes treatment (D1) acarbose, (D2) miglitol, and (D3) voglibose.

In this paper, ten derivatives from the isolated compounds were synthesized, together with natural compounds they were studied in molecular docking experiment on sugar beet α-glucosidase protein (PDB-3W37) and oligo-1,6-glucosidase protein (PDB-3AJ7). Three commercial medicines acarbose (D1), miglitol (D2), and voglibose (D3) had been reported able to lengthen the duration time of carbohydrate absorption and flatten the blood-glucose concentrations, thus widely used in the treatment for patients with type 2 diabetes.^23^ Therefore, they were specified as references in this study. Their structural formula is presented in Fig. 2.

## 2. Methodology

### 2.1. Experiments

#### 2.1.1 Material and methods

All analytically graded reagents were purchased from either Sigma-Aldrich or Merck & Co. and used without further purification. Solvents for column chromatography were distilled before use. Melting points (in °C) were determined on a Thermo Mel-temp 3.0 (USA). Infrared (IR) spectra were recorded on an IMPACT 410 Nicolet Spectrometer. ESI-MS spectra were obtained from an 1100 Agilent LC/MS ion Trap. 1D (^1^H, ^13^C and DEPT) and 2D (HSQC, HMBC, COSY, NOESY) NMR spectra were recorded on a Bruker Avance 500 MHz using tetramethylsilane (TMS) as the internal standard for ^1^H and solvent signal for ^13^C NMR. Thin-layer chromatography (TLC) was carried out on a pre-coated silica gel 60 F254 (Merck & Co.), visualised under ultraviolet (UV) radiation at *λ*_max_ 254 nm, and stained by a solution of 1% (w/w) vanillin in sulfuric acid. Column chromatography (CC) was performed on silica gel 300–400 mesh (Merck & Co.). Enzyme α-glucosidase was purchased from Sigma-Aldrich under the name code G0660, detected by a Biotek Epoch 2 microplate readers (USA).

#### 2.1.2 Isolation of catalpol iridoids from *Dolichandrone spathacea*

##### 2.1.2.1 Isolation of catalpol iridoids 1, 2, 13, 14

The procedures for the isolation of 1, 2, 13 and 14 were described in ref. 22. Iridoid 1 and 14 were isolated from the leaves, while 2 and 13 were isolated from the barks of *Dolichandrone spathacea* collected in Soc Trang province, Vietnam. Their structures were identified as 6-*O*-[*E*-4-methoxycinnamoyl]catalpol (1),^24,25^ 6-*O*-[(*E*)-3,4-dimethoxycinnamoyl]catalpol (2),^26,27^ minecoside (13),^28^ specioside (14).^29^

##### 2.1.2.2 Isolation of catalpol iridoid 10

Compound 10 was isolated from the leaves of *Dolichandrone spathacea* collected in Phu Loc district, Thua Thien-Hue province, Vietnam in December 2018. The plant was identified by Dr Do Xuan Cam, University of Agriculture and Forestry, Hue University. The dried powder of the leaves of *Dolichandrone spathacea* (250 g) was extracted with EtOH 90% at 50 °C (3 × 3 hours × 1 L). The EtOH extracts were combined and evaporated the solvent under reduced pressure. The obtained residue (37 g) was suspended in 350 mL MeOH/H_2_O (1 : 2) and partitioned with CH_2_Cl_2_. The organic layer was separated, then the remaining aqueous layer was dried to obtain the residue (25 g). The residue was subjected to a silica gel column with a gradient of CH_2_Cl_2_/MeOH 100 : 0 to 70 : 30 to give four fractions Fr.1–Fr.4. Fraction Fr.1 (2.0 g) was further purified by RP-18 column chromatography, eluting with MeOH/H_2_O (1 : 1) to afford compound 10 (5.1 mg).

#### 2.1.3 Synthesis of catalpol derivatives

##### 2.1.3.1 General procedure for the synthesis of 3, 4

To a stirred solution of 1 or 2 (0.1 mmol) dissolved in MeOH (5 mL) was added NiCl_2_·6H_2_O (0.5 mmol). The reaction mixture was stirred for 10 minutes at room temperature and cooled to 0 °C. NaBH_4_ (18.9 mg, 0.5 mmol) was slowly added to the mixture, which was then allowed to stir at 0 °C for 30 minutes and one additional hour at room temperature. The reaction mixture was then removed the solvent to obtain a white solid. The solid was dissolved in CH_2_Cl_2_ and the solution was washed with water (2 × 30 mL), dried over anhydrous Na_2_SO_4_, and evaporated the solvent. The crude product was purified over a silica gel column (CH_2_Cl_2_/MeOH 9 : 1) to obtain 3 (46 mg, yield: 83%) or 4 (44.5 mg, yield: 80.6%).

##### 2.1.3.2 Synthesis of 5

To a solution of 4 (55.2 mg, 0.1 mmol) in pyridine (2 mL) was added anhydride acetic acid (2 mL). After stirring for 12 hours at room temperature, the reaction mixture was added CH_2_Cl_2_ (20 mL) and HCl (1 N) to pH = 6, following by washing with a solution of NaHCO_3_ (5%) (2 × 10 mL) and water (2 × 10 mL). The organic layer was evaporated to obtain the crude product which was purified by silica gel column chromatography (*n*-hexane/ethyl acetate 2 : 1) to afford 5 (61.1 mg, 80%).

##### 2.1.3.3 Synthesis of 6

To a solution of 2 (55.2 mg, 0.1 mmol) in dried CHCl_3_ (3 mL) and pyridine (0.5 mL) was added triphenylmethyl chloride (55.6 mg, 0.2 mmol). The reaction mixture was allowed to stir for 12 hours at room temperature. The solvent was then removed under reduced pressure to give white solid which was dissolved in CHCl_3_ (30 mL) and washed with water (2 × 30 mL). The organic layer was dried over anhydrous Na_2_SO_4_, evaporated the solvent to afford a yellow solid, which was purified by silica gel column chromatography (CH_2_Cl_2_/MeOH/pyridine 98 : 2 : 0.5) to obtain 6.

##### 2.1.3.4 General procedure for the synthesis of 7, 8, 11, 12

To a solution of 1 or 2 or 13 or 14 (0.1 mmol) in pyridine (1 mL) was added anhydride acetic acid (1 mL). The solution was stirred for 24 h at room temperature then ice cold water (10 mL) was added and extracted with EtOAc (3 × 30 mL). The EtOAc extract was combined, dried with anhydrous Na_2_SO_4_, evaporated the solvent to give yellow solid. The crude product was subjected to a Sephadex LH-20 column (100% MeOH) to obtain the corresponding acetylated catalpol iridoids.

##### 2.1.3.5 Synthesis of 9

To a solution of 1 (52.4 mg, 0.1 mmol) and TsOH (4 mg) in dried DMF was added dropwise 2,2-dimethoxypropane (0.26 mg, 0.25 mmol). The reaction mixture was heated to 50 °C for three hours, neutralized by triethylamine, following by the addition of EtOAc (100 mL) and water (50 mL). The organic layer was separated, washed with water (2 × 50 mL), dried over anhydrous Na_2_SO_4_ and evaporated the solvent to give a white solid, this was then purified by a silica gel column chromatography (DCM/MeOH 95 : 5) to obtain 9 (40 mg, 71%).

##### 2.1.3.6 Synthesis of 15

Compound 2 (110.4 mg, 0.2 mmol) was dissolved in a mixture of (H_2_O/THF/EtOH 1 : 1 : 1) and TEA (1 mL). Hydroxyl amine hydrochloride (69.5 mg, 1 mmol) was then added to the solution. The reaction mixture was heated to 100–110 °C for 6 hours. The solvent was evaporated to give a residue which was then suspended in water and partitioned with EtOAc (3 × 20 mL). The organic layers were combined, dried over anhydrous Na_2_SO_4_ and evaporated the solvent to afford a white solid, this was subjected to a silica gel column chromatography (CH_2_Cl_2_/MeOH 9 : 1) to obtain 15 (65.2 mg, 90%).

#### 2.1.4 Enzyme assay of α-glucosidase

Each assay was performed on 96 well-plates (200 μL per well). The test samples were dissolved in DMSO 10% and further diluted to the concentrations: 1024, 256, 64, 16, 4, 1 μg.mL^−1^. Each concentration of the test sample (10 μL) was incubated at 37 °C with phosphate buffer 100 mM (pH 6.8, 40 μL), α-glucosidase 0.4 U mL^−1^ (25 μL), *p*-nitrophenyl *α*-d-glucopyranoside 2.5 mM (25 μL). After 30 minutes, the reaction was terminated by adding 100 μL of 0.1 M Na_2_CO_3_ solution. The enzyme activities were determined by measuring the absorbance at *λ*_max_ 410 nm. The control was prepared using the same procedure replacing the test sample by distilled water, while activity of the reference was tested by replacing the test sample with acarbose^30^ Percentage of inhibition was calculated as followed:^31^
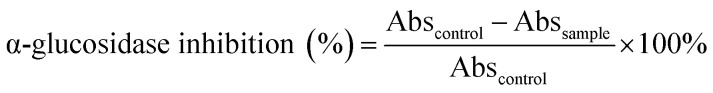


### 2.2. Computation

#### 2.2.1 Molecular docking simulation

The simulation was carried out on MOE 2015.10 software. The structural information of targeted proteins (3W37 and 3AJ7) and docked ligands (iridoid compounds and referenced drugs) were required as the precursors. As conducted, this served as inputs to simulate intermolecular interaction between the agents, thereby yielding information on the respective ligand-protein inhibitory complex. The output included inhibitory configurations, docking score (DS) energy, root-mean-square deviation (RMSD), interaction types, and distances between ligands and proteins. In a typical procedure, a molecular docking simulation follows three steps.^13–15,32^

##### 2.2.1.1 Pre-docking preparation

Structural data of carbohydrate-hydrolase proteins 3W37 (DOI: 10.2210/pdb3W37/pdb) and 3AJ7 (DOI: 10.2210/pdb3AJ7/pdb) were downloaded from Worldwide Protein Data Bank. The proteins and their 3D protonation were prepared by MOE QuickPrep functionality. Determination of protein active zones based upon a radius set at 4.5 Å from their amino acids and the inhibitory ligands. The preparatory protein structures obtained were saved in format *.pdb, ready for docking simulation. Independently, the ligands, including iridoid compounds and referenced drugs, were structurally optimised by Conj Grad for energy minimisation. The optimisation configuration was set: termination for energy change = 0.0001 kcal mol^−1^; max interactions = 1000; charge modification by Gasteiger–Huckel method.

##### 2.2.1.2 Docking investigation

Simulation on intermolecular interaction between the investigated agents was performed on MOE 2015.10 and the obtained inhibitory structures were saved in format *.sdf. Docking simulation parameters were configured as: poses retaining for further inhibition analysis = 10; maximum solutions per iteration = 1000; maximum solutions per fragmentation = 200.

##### 2.2.1.3 Post-docking analysis

The inhibitability of a certain duo-system was primarily predicted by docking score (DS) energy, which represents binding affinity of ligands and their targeted proteins in the site–site distance. Intermolecular interactions formed between the ligands and in-pose amino acids of the proteins were also probed. These include hydrophilic binding and hydrophobic interaction. The former is a variety of hydrogen-bond types, *e.g.* electron-transferring (H-acceptor/donor), cation–arene (H–π), arene–arene (π–π), and ionic; while, van der Waals forces constitute the latter. The results provide in-bonding amino acids, bonding lengths, and their Gibbs free energy. Besides, binding stability of the complex static conformation was evaluated by the respective values of root-mean-square deviation (RMSD) calculated. This is based on the fact that RMSD represent the average between neighbouring atoms; therefore, a smaller value means a more tightly bound conformation is formed. In addition, ligand conformation and orientation in its inhibited-protein active site was visualised on 2D and 3D planes.

#### 2.2.2 Physicochemical and pharmaceutical compatibility

The docking parameters including DS_average_ (kcal mol^−1^), molecular mass (Da), polarizability (Å^3^) and volume or size (Å), and dispersion coefficients (log *P* and log *S*) were achieved by Gasteiger–Marsili method using QSARIS system.^33^ They were then prescreened in an attempt to evaluate their orally pharmacological compatibility. This was based on Lipinski's rule of five, a well-known set of indicators to predict drug-likeness.^34^ According to Lipinski's criteria, a well membrane-permeable molecule should satisfy the requirements: (1) molecular mass < 500 Da; (2) no more than 5 groups for hydrogen bonds; (3) no more than 10 groups receiving hydrogen bonds; (4) the value of log *P* is less than +5 (log *P* < 5).^35,36^

## 3. Results and discussion

### 3.1. Characterisation

#### 3.1.1 Compound 1: 6-*O*-[(*E*)-4-methoxycinnamoyl]catalpol

White amorphous powder, C_25_H_30_O_12_, ESI-MS (*m*/*z*): 523.1 [M + H]^+^, 521.0 [M − H]^−^; IR (KBr, *ν*_max_, cm^−1^): 2936 (–CH alkane), 1716 (C=O ester), 1633 (C=C alkene), 1602 (C=C–Ar), 1063 (C–O ether); ^1^H-NMR (CD_3_OD + CDCl_3_, 500 MHz), *δ*_H_ (ppm), *J* (Hz): 7.69 (1H, d, *J* = 16.0, H-7′′), 7.55 (2H, d, *J* = 8.0, H-2′′, H-6′′), 6.95 (2H, d, *J* = 8.0, H-3′′, H-5′′), 6.41 (1H, d, *J* = 16.0, H-8′′), 6.36 (1H, dd, *J* = 6.0, 1.5, H-3), 5.15 (1H, d, *J* = 9.0, H-1), 5.05 (1H, dd, *J* = 7.5, 1.0, H-6), 5.00 (1H, dd, *J* = 6.0, 1.5, H-4), 4.81 (1H, d, *J* = 8.0, H-1′), 4.16 (1H, d, *J* = 13.0, H-10), 3.94 (1H, dd, *J* = 12.0, 2.0, H-6′), 3.87 (1H, d, *J* = 13.0, H-10), 3.84 (3H, s, 4′′-OCH_3_), 3.73 (1H, br s, H-7), 3.68 (1H, dd, *J* = 12.0, 2.0, H-6′), 3.46–3.42 (1H, m, H-3′), 3.35–3.34 (1H, m, H-5′), 3.33–3.32 (1H, m, H-2′), 3.30–3.28 (1H, m, H-4′), 2.67–2.64 (1H, m, H-9), 2.63–2.61 (1H, m, H-5); ^13^C-NMR (CD_3_OD + CDCl_3_, 125 MHz), *δ*_C_ (ppm): 168.61 (C-9′′), 162.98 (C-4′′), 146.69 (C-7′′), 142.17 (C-3), 130.98 (C-6′′), 130.90 (C-2′′), 127.92 (C-1′′), 115.31 (C-8′′), 115.26 (C-3′′, C-5′′), 102.74 (C-4), 99.47 (C-1′), 94.88 (C-1), 81.09 (C-6), 78.25 (C-5′), 77.38 (C-3′), 74.50 (C-2′), 71.39 (C-4′), 66.67 (C-8), 62.63 (C-6′), 61.12 (C-10), 60.05 (C-7), 55.81 (4′′-OCH_3_), 42.89 (C-9), 36.46 (C-5).

#### 3.1.2 Compound 2: 6-*O*-[(*E*)-3,4-dimethoxycinnamoyl]catalpol

White amorphous powder, C_26_H_32_O_13_, (+)-ESI-MS (*m*/*z*): 588.2 [M + Cl]^−^; IR (KBr, *ν*_max_, cm^−1^): 2903 (–CH alkane), 1717 (C=O ester), 1635 (C=C alkene), 1594 (C=C–Ar), 1086 (C–O ether); ^1^H-NMR (CD_3_OD + CDCl_3_, 500 MHz), *δ*_H_ (ppm), *J* (Hz): 7.68 (1H, d, *J* = 15.5, H-7′′), 7.14 (1H, dd, *J* = 8.5, 1.5, H-6′′), 7.09 (1H, d, *J* = 1.5, H-2′′), 6.90 (1H, d, *J* = 8.5, H-5′′), 6.38 (1H, d, *J* = 15.5, H-8′′), 6.34 (1H, dd, *J* = 4.5, 2.0, H-3), 5.05–5.00 (3H, m, H-1, H-4, H-6), 4.84 (1H, d, *J* = 8.0, H-1′), 4.03 (1H, d, *J* = 13.0, H-10), 3.95 (1H, m, H-10), 3.92 (6H, s, 3′′-OCH_3_, 4′′-OCH_3_), 3.88 (1H, dd, *J* = 12.0, 5.5, H-6′), 3.78 (1H, dd, *J* = 12.0, 5.5, H-6′), 3.74 (1H, br s, H-7), 3.52–3.48 (2H, m, H-3′, H-5′), 3.39–3.36 (2H, m, H-2′, H-4′), 2.71–2.66 (1H, m, H-9), 2.65–2.64 (1H, m, H-5); ^13^C-NMR (CD_3_OD + CDCl_3_, 125 MHz), *δ*_C_ (ppm): 167.32 (C-9′′), 151.30 (C-4′′), 149.10 (C-3′′), 145.90 (C-7′′), 141.05 (C-3), 127.01 (C-1′′), 122.86 (C-6′′), 114.64 (C-8′′), 111.00 (C-2′′), 109.72 (C-5′′), 102.06 (C-4), 98.58 (C-1′), 94.26 (C-1), 79.65 (C-6), 76.41 (C5′), 75.97 (C-3′), 73.02 (C-2′), 69.29 (C-4′), 65.42 (C-8), 60.91 (C-6′), 60.53 (C-10), 59.12 (C-7), 55.81 (3′′-OCH_3_), 55.76 (4′′-OCH_3_), 41.76 (C-9), 35.17 (C-5).

#### 3.1.3 Compound 3: 6-*O*-(4-methoxyphenylpropanoyl)catalpol

White amorphous powder, C_25_H_32_O_12_, HR-ESI-MS (*m*/*z*): 547.1779 [M + Na]^+^ (calculated for C_25_H_32_O_12_Na 547.1791), ESI-MS (*m*/*z*): 559.0 [M + Cl]^−^; ^1^H-NMR (CD_3_OD, 500 MHz), *δ*_H_ (ppm), *J* (Hz): 7.15 (2H, d, *J* = 8.5, H-2′′, H-6′′), 6.85 (2H, d, *J* = 8.5, H-3′′, H-5′′), 6.34 (1H, dd, *J* = 6.0, 1.5, H-3), 5.12 (1H, d, *J* = 9.5, H-1), 4.92 (1H, dd, *J* = 8.0, 1.5, H-4), 4.79 (1H, d, *J* = 7.5, H-6), 4.15 (1H, d, *J* = 13.0, H-10), 3.94 (1H, dd, *J* = 12.0, 2.0, H-6′), 3.82 (1H, d, *J* = 13.0, H-10), 3.77 (3H, s, 4′′-OCH_3_), 3.65 (1H, dd, *J* = 12.0, 6.5, H-6′), 3.61 (1H, s, H-7), 3.41 (1H, t, *J* = 9.0, H-3′), 3.35–3.26 (3H, m, H-2′, H-4′, H-5′), 2.91 (2H, t, *J* = 7.5, H-7′′), 2.69 (2H, t, *J* = 7.5, H-8′′), 2.60–2.57 (1H, m, H-9), 2.48–2.44 (1H, m, H-5); ^13^C-NMR (CD_3_OD, 125 MHz), *δ*_C_ (ppm): 174.52 (C-9′′), 159.70 (C-4′′), 142.25 (C-3), 133.69 (C-1′′), 130.34 (C-2′′, C-6′′), 114.93 (C-3′′, C-5′′), 102.85 (C-4), 99.70 (C-1′), 95.04 (C-1), 81.32 (C-6), 78.62 (C-5′), 77.70 (C-3′), 74.83 (C-2′), 71.77 (C-4′), 66.77 (C-8), 62.92 (C-10), 61.24 (C-6′), 60.07 (C-7), 55.68 (4′′-OCH_3_), 43.13 (C-9), 37.04 (C-8′′), 36.60 (C-5), 31.15 (C-7′′).

#### 3.1.4 Compound 4: 6-*O*-(3,4-dimethoxyphenylpropanoyl)catalpol

White amorphous powder, C_26_H_34_O_13_, HR-ESI-MS (*m*/*z*): 577.1867 [M + Na]^+^ (calculated for C_26_H_34_O_13_Na 577.1897), ESI-MS (*m*/*z*): 589.1 [M + Cl]^−^; ^1^H-NMR (CD_3_OD, 500 MHz), *δ*_H_ (ppm), *J* (Hz): 6.88 (2H, d, *J* = 8.0, H-2′′, H-6′′), 6.80 (1H, dd, *J* = 8.0, 2.0, H-5′′), 6.34 (1H, dd, *J* = 6.0, 2.0, H-3), 5.12 (1H, d, *J* = 9.5, H-1), 4.92 (1H, dd, *J* = 7.0, 1.5, H-4), 4.79 (1H, d, *J* = 8.0, H-6), 4.15 (1H, d, *J* = 13.0, H-10), 3.94 (1H, dd, *J* = 12.0, 2.0, H-6′), 3.84–3.82 (1H, m, H-10), 3.83 (3H, s, OCH_3_), 3.81 (3H, s, OCH_3_), 3.65 (1H, dd, *J* = 12.0, 6.5, H-6′), 3.61 (1H, s, H-7), 3.41 (1H, t, *J* = 9.0, H-3′), 3.33–3.25 (3H, m, H-2′, H-4′, H-5′), 2.93 (2H, t, *J* = 7.5, H-7′′), 2.72 (2H, t, *J* = 7.5, H-8′′), 2.60–2.57 (1H, m, H-9), 2.48–2.43 (1H, m, H-5); ^13^C-NMR (CD_3_OD, 125 MHz), *δ*_C_ (ppm): 174.55 (C-9′′), 150.47 (C-4′′), 149.07 (C-3′′), 142.28 (C-3), 134.74 (C-1′′), 121.70 (C-6′′), 113.59 (C-2′′), 113.33 (C-5′′), 102.82 (C-4), 99.70 (C-1′), 95.04 (C-1), 81.36 (C-6), 78.64 (C-5′), 77.71 (C-3′), 74.84 (C-2′), 71.78 (C-4′), 66.78 (C-8), 62.93 (C-10), 61.24 (C-6′), 60.07 (C-7), 56.61 (4′′-OCH_3_), 56.49 (3′′-OCH_3_), 43.14 (C-9), 36.96 (C-8′′), 36.62 (C-5), 31.60 (C-7′′).

#### 3.1.5 Compound 5: 6-*O*-(3,4-dimethoxyphenylpropanoyl)catalpolpentaacetate

White amorphous powder, C_36_H_44_O_18_, HR-ESI-MS (*m*/*z*): 787.2389 [M + Na]^+^ (calculated for C_36_H_44_O_18_Na 787.2425), ESI-MS (*m*/*z*): 765.0 [M + H]^+^; ^1^H-NMR (CD_3_OD, 500 MHz), *δ*_H_ (ppm), *J* (Hz): 6.88 (2H, d, *J* = 7.5, H-2′′, H-6′′), 6.78 (1H, dd, *J* = 7.5, 2.0, H-5′′), 6.34 (1H, dd, *J* = 6.0, 1.5, H-3), 5.30 (1H, t, *J* = 9.5, H-6), 5.11–5.03 (3H, m, H-1, H-4, H-5′), 4.93–4.87 (3H, m, H-2′, H-3′, H-4′), 4.36 (1H, dd, *J* = 12.0, 2.5, H-6′), 4.23 (1H, dd, *J* = 12.5, 4.0, H-10), 3.90 (1H, m, H-6′), 3.84 (3H, s, OCH_3_), 3.81 (3H, s, OCH_3_), 3.80 (1H, d, *J* = 12.5 Hz, H-10), 3.63 (1H, d, *J* = 1.0, H-7), 2.93 (2H, t, *J* = 7.0, H-7′′), 2.72 (2H, t, *J* = 7.0, H-8′′), 2.56–2.52 (1H, m, H-9), 2.45–2.41 (1H, m, H-5), 10,2′,3′,4′,6′-OCOCH_3_: 2.13, 2.10, 2.04, 2.03, 2.00. ^13^C-NMR (CD_3_OD, 125 MHz), *δ*_C_ (ppm): 174.42 (C-9′′), 172.64, 172.45, 171.60, 171.14, 170.92 (5×-OCOCH_3_), 150.49 (C-4′′), 149.10 (C-3′′), 142.29 (C-3), 134.68 (C-1′′), 121.70 (C-6′′), 113.61 (C-2′′), 113.35 (C-5′′), 102.96 (C-4), 98.09 (C-1′), 95.92 (C-1), 81.01 (C-6), 74.04 (C-5′), 73.33 (C-3′), 72.22 (C-2′), 69.61 (C-4′), 64.68 (C-10), 63.65 (C-8), 62.28 (C-6′), 59.93 (C-7), 56.61 (4′′-OCH_3_), 56.49 (3′′-OCH_3_), 42.72 (C-9), 36.88 (C-8′′), 36.44 (C-5), 31.56 (C-7′′), 20.75, 20.71, 20.57, 20.54, 20.51 (5×-OCOC̲H_3_).

#### 3.1.6 Compound 6: 6-*O*-[(*E*)-3,4-dimethoxy]catalpol-10,6′-*O*-ditriphenylmethyl

White amorphous powder, C_64_H_60_O_13_, HR-ESI-MS (*m*/*z*): 817.2788 (100) [C_45_H_46_O_13_Na]^+^ [M − C_19_H_15_ + Na + H]^+^ (calculated for C_45_H_46_O_13_Na: 817.2830); ^1^H-NMR (CDCl_3_, 500 MHz), *δ*_H_ (ppm), *J* (Hz): 7.69 (1H, d, *J* = 15.5, H-7′′), 7.39–7.15 (30H, m, 2×-OC(C_6_H_5_)_3_), 7.13 (1H, dd, *J* = 8.0, 2.0, H-6′′), 7.07 (1H, d, *J* = 2.0, H-2′′), 6.88 (1H, d, *J* = 8.0, H-5′′), 6.38 (1H, d, *J* = 15.5, H-8′′), 6.31 (1H, d, *J* = 6.5, H-3), 5.01 (1H, dd, *J* = 6.0, 4.0, H-1), 4.96 (1H, d, *J* = 6.5, H-4), 4.74 (1H, d, *J* = 9.0, H-1′), 4.59 (1H, d, *J* = 8.0, H-6), 3.91 (6H, s, 3′′,4′′-OCH_3_), 3.86 (1H, m, H-10), 3.75 (1H, d, *J* = 11.5, H-10), 3.45 (1H, t, *J* = 9.0, H-7), 3.36 (4H, m, H-2′, H-3′, H-4′, H-5′), 3.27 (1H, t, *J* = 8.5, H-6′), 3.11 (1H, m, H-6′), 2.76 (1H, t, *J* = 8.5, H-9), 2.68 (1H, d, *J* = 8.5, H-5); ^13^C-NMR (CDCl_3_, 125 MHz), *δ*_C_ (ppm): 166.98 (C-9′′), 151.43 (C-4′′), 149.34 (C-3′′), 146.92 (2×-OC̲(C_6_H_5_)_3_), 128.80–127.25 (2×-OC(C̲_6_H_5_)_3_), 87.48 and 86.60 (C-10 and C-6′), 145.68 (C-7′′), 141.09 (C-3), 127.09 (C-1′′), 122.83 (C-6′′), 115.15 (C-8′′), 111.18 (C-2′′), 109.82 (C-5′′), 102.44 (C-4), 97.27 (C-1′), 93.72 (C-1), 79.65 (C-6), 76.80 (C-5′), 76.30 (C-3′), 73.57 (C-2′), 72.54 (C-4′), 65.09 (C-8), 64.48 (C-6′), 61.22 (C-10), 59.04 (C-7), 55.93 (3′′,4′′-OCH_3_), 42.80 (C-9), 35.53 (C-5).

#### 3.1.7 Compound 7: minecoside pentaacetate

White amorphous powder, C_37_H_42_O_19_, HR-ESI-MS (*m*/*z*): 813.2209 [M + Na]^+^ (calculated for C_37_H_42_O_19_Na 813.2218), 791.2336 [M + H]^+^ (calculated for C_37_H_43_O_19_ 791.2399); ^1^H-NMR (CDCl_3_, 500 MHz), *δ*_H_ (ppm), *J* (Hz): 7.63 (1H, d, *J* = 16.0, H-7′′), 7.08–7.07 (3H, m, H-2′′, H-3′′, H-6′′), 7.00 (1H, d, *J* = 8.0, H-5′′), 6.38 (1H, d, *J* = 16.0, H-8′′), 6.27 (1H, dd, *J* = 6.0, 1.5, H-3), 5.18–5.15 (1H, m, H-1), 5.09–5.05 (1H, m, H-6), 4.96–4.90 (4H, m, H-4, H-2′, H-3′, H-4′), 4.82–4.79 (2H, m, H-1′, H-5′), 4.26 (1H, dd, *J* = 12.0, 2.0, H-6′), 4.13 (1H, dd, *J* = 12.0, 4.0, H-6′), 3.92 (1H, d, *J* = 12.5, H-10), 3.80 (3H, s, 4′′-OCH_3_), 3.69 (1H, overlap, H-10), 3.66 (1H, br s, H-7), 2.61–2.58 (1H, m, H-9), 2.57–2.54 (1H, m, H-5), 2.25 (3H, s), 2.06 (6H, s), 1.98 (3H, s), 1.97 (3H, s), 1.95 (3H, s) (5×-OCOC̲H_3_); ^13^C-NMR (CDCl_3_, 125 MHz), *δ*_C_ (ppm): 170.96, 170.87, 170.37, 169.51, 169.32, 168.97 (5×-OC̲OCH_3_), 166.72 (C-9′′), 151.32 (C-4′′), 245.26 (C-7′′), 141.57 (C-3′′), 141.03 (C-3), 132.97 (C-1′′), 123.18 (C-6′′), 121.32 (C-8′′), 117.20 (C-2′′), 111.30 (C-5′′), 101.89 (C-4), 96.50 (C-1′), 94.16 (C-1), 79.50 (C-6), 72.50 (C-5′), 72.12 (C-3′), 68.12 (C-4′), 62.58 (C-8), 62.32 (C-6′), 58.77 (C-7), 55.80 (4′′-OCH_3_), 41.47 (C-9), 34.96 (C-5), 20.51 (–OCOC̲H_3_), 20.46 (–OCOC̲H_3_), 20.43 (3×-OCOC̲H_3_).

#### 3.1.8 Compound 8: 6-*O*-[(*E*)-3,4-dimethoxycinnamoyl]catalpolpentaacetate

White amorphous powder, C_36_H_42_O_18_, (+)-ESI-MS (*m*/*z*): 763.9 [M + H]^+^; ^1^H-NMR (CD_3_OD, 500 MHz), *δ*_H_ (ppm), *J* (Hz): 7.68 (1H, d, *J* = 16.0, H-7′′), 7.12 (1H, dd, *J* = 8.0, 1.5, H-6′′), 7.07 (1H, d, *J* = 1.5, H-2′′), 6.87 (1H, d, *J* = 8.0, H-5′′), 6.36 (1H, d, *J* = 16.0, H-8′′), 6.32 (1H, dd, *J* = 6.0, 1.5, H-3), 5.25–5.21 (1H, m, H-1), 5.16–5.12 (1H, m, H-6), 5.03–4.96 (4H, m, H-4, H-2′, H-3′, H-4′), 4.89–4.85 (2H, m, H-1′, H-5′), 4.31 (1H, dd, *J* = 12.5, 2.5, H-6′), 4.21 (1H, dd, *J* = 12.5, 4.0, H-10), 3.99 (1H, d, *J* = 12.5, H-10), 3.91 (6H, s, 3′′-OCH_3_, 4′′-OCH_3_), 3.73 (1H, m, H-6′), 3.71 (1H, br s, H-7), 2.70–2.65 (1H, m, H-9), 2.64–2.62 (1H, m, H-5); ^13^C-NMR (CDCl_3_, 125 MHz), *δ*_C_ (ppm): 170.70, 170.54, 170.22, 169.28, 169.07 (5×OC̲OCH_3_), 167.05 (C-9′′), 151.39 (C-4′′), 149.21 (C-3′′), 145.92 (C-7′′), 141.06 (C-3), 127.07 (C-1′′), 122.91 (C-6′′), 114.72 (C-8′′), 111.01 (C-2′′), 109.59 (C-5′′), 102.09 (C-4), 96.57 (C-1′), 94.23 (C-1), 79.28 (C-6), 72.51 (C-5′), 72.26 (C-3′), 68.15 (C-4′), 62.57 (C-8), 62.35 (C-6′), 61.16 (C-10), 58.85 (C-7), 55.95 (3′′-OCH_3_), 55.85 (4′′-OCH_3_), 41.53 (C-9), 35.01 (C-5), 20.70 (–OCOC̲H_3_), 20.65 (–OCOC̲H_3_), 20.61 (–OCOC̲H_3_), 20.57 (2×-OCOC̲H_3_).

#### 3.1.9 Compound 9: 6-*O*-[(*E*)-4-methoxycinnamoyl]catalpol-4′,6′-acetonide

White amorphous powder, C_28_H_34_O_12_, HR-ESI-MS (*m*/*z*): 585.1956 [M + Na]^+^ (calculated for C_28_H_34_O_12_Na 585.1948); ^1^H-NMR (CDCl_3_, 500 MHz), *δ*_H_ (ppm), *J* (Hz): 7.68 (1H, d, *J* = 15.5, H-7′′), 7.39 (2H, dd, *J* = 7.0, 2.0, H-2′′, H-6′′), 6.91 (2H, dd, *J* = 7.0, 2.0, H-3′′, H-5′′), 6.37 (1H, m, H-8′′), 6.35 (1H, m, H-3), 5.04 (1H, m, H-1), 5.02 (1H, m, H-6), 4.93 (1H, m, H-4), 4.89 (1H, m, H-1′), 4.03 (1H, d, *J* = 10.5, H-6′), 3.97 (1H, dd, *J* = 11.0, 5.5, H-4′), 3.76 (1H, d, *J* = 10.5, H-6′), 3.53 (1H, m, H-3′), 3.38 (2H, m, H-2′, H-5′), 2.73 (1H, dd, *J* = 10.0, 8.0, H-9), 2.66 (1H, m, H-5), 1.53 and 1.45 (each 3H, s, 7′-CH_3_); ^13^C-NMR (CDCl_3_, 125 MHz), *δ*_C_ (ppm): 167.21 (C-9′′), 161.69 (C-4′′), 145.66 (C-7′′), 141.06 (C-3), 129.95 (C-2′′, C-6′′), 125.96 (C-1′′), 114.67 (C-8′′), 114.44 (C-3′′, C-5′′), 102.51 (C-4), 99.97 (C-7′), 99.43 (C-1′), 94.84 (C-1), 79.46 (C-6), 74.25 (C-5′), 73.54 (C-3′), 72.92 (C-2′), 67.89 (C-4′), 65.20 (C-8), 61.92 (C-6′), 61.42 (C-10), 59.19 (C-7), 55.40 (4′′-OCH_3_), 42.10 (C-9), 36.50 (C-5), 29.00 and 20.70 (7′-CH_3_).

#### 3.1.10 Compound 10: nemoroside

White amorphous powder, C_25_H_36_O_12_; (+)-ESI-MS (*m*/*z*): 551.1 [M + Na]^+^, (−)-ESI-MS (*m*/*z*): 563.0 [M + Cl]^−^; ^1^H-NMR (CD_3_OD, 500 MHz), *δ*_H_ (ppm), *J* (Hz): 6.74 (1H, dd, *J* = 7.0, 6.5, H-3′′), 6.26 (1H, dd, *J* = 6.0, 1.5, H-3), 5.30 (1H, m, H-7′′), 5.06 (1H, d, *J* = 9.5, H-1), 4.85 (2H, m, H-4, H-6), 4.70 (1H, overlap, H-1′), 4.05 (1H, d, *J* = 13.0, H-10), 3.99 (2H, d, *J* = 6.5, H-8′′), 3.83 (1H, dd, *J* = 12.0, 1.5, H-6′), 3.72 (1H, d, *J* = 13.0, H-10), 3.57 (1H, s, H-7), 3.54 (1H, dd, *J* = 12.0, 6.5, H-6′), 3.30 (1H, t, *J* = 9.0, H-2′), 3.21 (1H, m, H-3′), 3.16–3.14 (1H, m, H-4′, H-5′), 2.52–2.46 (2H, m, H-5, H-9), 2.26 (2H, m, H-4′′), 2.08 (2H, m, H-5′′), 1.77 (3H, s, H-9′′), 1.60 (3H, s, H-10′′); ^13^C-NMR (CD_3_OD, 125 MHz), *δ*_C_ (ppm): 169.34 (C-1′′), 144.15 (C-3′′), 142.38 (C-3), 138.37 (C-6′′), 128.60 (C-2′′), 125.74 (C-7′′), 102.91 (C-4), 99.72 (C-1′), 95.08 (C-1), 81.62 (C-6), 78.65 (C-3′), 77.72 (C-2′), 74.85 (C-5′), 71.78 (C-4′), 66.79 (C-8), 62.93 (C-10), 61.27 (C-6′), 60.17 (C-7), 59.37 (C-8′′), 43.17 (C-9), 39.06 (C-5′′), 36.67 (C-5), 28.06 (C-4′′), 16.18 (C-10′′), 12.47 (C-9′′).

#### 3.1.11 Compound 11: 6-*O*-[(*E*)-4-methoxycinnamoyl]catalpolhexaacetate

White amorphous powder, C_35_H_40_O_17_, ESI-MS (*m*/*z*): 755.0 [M + Na + H_2_O]^+^, 791.1 [M + Na + 2H_2_O]^+^; ^1^H-NMR (CD_3_OD, 500 MHz), *δ*_H_ (ppm), *J* (Hz): 7.70 (1H, d, *J* = 15.5, H-7′′), 7.49 (2H, d, *J* = 8.5, H-2′′, H-6′′), 6.91 (2H, d, *J* = 8.5, H-3′′, H-5′′), 6.35 (1H, d, *J* = 15.5, H-8′′), 6.31 (1H, d, *J* = 6.0, H-3), 5.25–5.23 (1H, m, H-1), 5.16–5.12 (1H, m, H-6), 5.02–4.97 (1H, m, H-4), 4.98–4.96 (3H, m, H-2′, H-3′, H-4′), 4.89–4.86 (2H, m, H-1′, H-5′), 4.30 (1H, dd, *J* = 12.0, 2.0, H-6′), 4.21 (1H, dd, *J* = 12.0, 4.0, H-6′), 3.99 (1H, d, *J* = 12.5, H-10), 3.83 (3H, s, 4′′-OCH_3_), 3.73 (1H, overlap, H-10), 3.71 (1H, br s, H-7), 2.69–2.66 (1H, m, H-5), 2.64–2.62 (1H, m, H-9), 2.13 (3H, s), 2.12 (3H, s), 2.05 (3H, s), 2.05 (3H, s), 2.04 (3H, s), 2.01 (3H, s) (6×-OCOC̲H_3_); ^13^C-NMR (CDCl_3_, 125 MHz), *δ*_C_ (ppm): 170.62 (2×-OC̲OCH_3_), 170.46 (–OC̲OCH_3_), 170.16 (–OC̲OCH_3_), 169.23 (–OC̲OCH_3_), 169.02 (–OC̲OCH_3_), 167.12 (C-9′′), 161.64 (C-4′′), 145.63 (C-7′′), 141.01 (C-3), 129.88 (C-2′′, C-6′′), 126.85 (C-1′′), 114.54 (C-8′′), 114.38 (C-3′′, C-5′′), 102.13 (C-4), 96.59 (C-1′), 94.26 (C-1), 79.26 (C-6), 72.53 (C-5′), 72.28 (C-3′), 70.57 (C-2′), 68.20 (C-4′), 62.53 (C-8), 62.32 (C-6′), 61.19 (C-10), 58.83 (C-7), 55.34 (4′′-OCH_3_), 41.55 (C-9), 34.99 (C-5), 20.66 (–OCOC̲H_3_), 20.61 (–OCOC̲H_3_), 20.58 (–OCOC̲H_3_), 20.54 (3×-OCOC̲H_3_).

#### 3.1.12 Compound 12: specioside hexaacetate

White amorphous powder, C_36_H_40_O_18_; (+)-ESI-MS (*m*/*z*): 783.2 [M + Na]^+^; ^1^H-NMR (CDCl_3_, 500 MHz), *δ*_H_ (ppm), *J* (Hz): 7.77 (1H, d, *J* = 16.0, H-7′′), 7.70 (2H, d, *J* = 8.5, H-2′′, H-6′′), 7.19 (2H, d, *J* = 8.5, H-3′′, H-5′′), 6.60 (1H, d, *J* = 16.0, H-8′′), 6.40 (1H, d, *J* = 5.5, H-3), 5.31 (1H, t, *J* = 9.5, H-1), 5.13–5.12 (1H, m, H-6), 5.09–5.02 (4H, m, H-4, H-2′, H-3′, H-4′), 5.00–4.98 (1H, m, H-5′), 4.94 (1H, m, H-1′), 4.38 (1H, dd, *J* = 12.5, 2.5, H-6′), 4.23 (1H, dd, *J* = 12.5, 4.0, H-6′), 3.93 (1H, overlap, H-10), 3.81 (1H, d, *J* = 12.5, H-10), 3.76 (1H, br s, H-7), 2.61–2.60 (2H, m, H-5, H-9), 2.30 (3H, s), 2.14 (3H, s), 2.12 (3H, s), 2.04 (6H, s), 2.00 (3H, s) (6×-OCOC̲H_3_); ^13^C-NMR (CDCl_3_, 125 MHz), *δ*_C_ (ppm): 172.68, 172.49, 171.62, 171.16, 170.95, 170.77 (6×OC̲OCH_3_), 168.13 (C-9′′), 154.03 (C-4′′), 146.03 (C-7′′), 142.43 (C-3), 133.31 (C-1′′), 130.56 (C-2′′, C-6′′), 123.45 (C-3′′, C-5′′), 118.39 (C-8′′), 103.02 (C-4), 98.13 (C-1′), 95.98 (C-1), 81.26 (C-6), 74.05 (C-5′), 73.36 (C-3′), 72.23 (C-2′), 69.63 (C-4′), 64.76 (C-8), 63.73 (C-6′), 60.06 (C-7), 42.79 (C-9), 36.59 (C-5), 20.90, 20.77, 20.73, 20.59, 20.54, 20.52 (6× OCOC̲H_3_).

#### 3.1.13 Compound 13: minecoside

White amorphous powder, C_25_H_30_O_13_; ^1^H-NMR (CD_3_OD, 500 MHz), *δ*_H_ (ppm), *J* (Hz): 7.68 (1H, d, *J* = 16.0, H-7′′), 7.21 (1H, br s, H-2′′), 7.11 (1H, dd, *J* = 8.0, 1.5, H-6′′), 6.82 (1H, d, *J* = 8.0, H-5′′), 6.40–6.38 (1H, m, H-3), 6.39 (1H, overlap, H-8′′), 5.18 (1H, d, *J* = 9.5, H-1), 5.05 (1H, d, *J* = 7.0, H-6), 5.00 (1H, dd, *J* = 6.0, 4.0, H-4), 4.78 (1H, d, *J* = 8.0, H-1′), 4.18 (1H, d, *J* = 13.0, H-10), 3.95 (1H, dd, *J* = 12.0, 2.5, H-6′), 3.92 (3H, s, 4′′-OCH_3_), 3.88 (1H, d, *J* = 13.0, H-10), 3.72 (1H, br s, H-7), 3.67 (1H, dd, *J* = 12.0, 6.5, H-6′), 3.45–3.41 (1H, m, H-3′), 3.35–3.34 (1H, m, H-5′), 3.31–3.27 (2H, m, H-2′, H-4′), 2.66–2.62 (1H, m, H-9), 2.61–2.60 (1H, m, H-5); ^13^C-NMR (CD_3_OD, 125 MHz), *δ*_C_ (ppm): 167.54 (C-9′′), 151.25 (C-4′′), 147.85 (C-3′′), 145.74 (C-7′′), 142.39 (C-3), 127.98 (C-1′′), 122.59 (C-6′′), 114.62 (C-8′′), 112.06 (C-2′′), 110.85 (C-5′′), 102.93 (C-4), 99.69 (C-1′), 95.07 (C-1), 81.29 (C-6), 78.66 (C-5′), 77.70 (C-3′), 74.85 (C-2′), 71.78 (C-4′), 66.82 (C-8), 62.93 (C-6′), 61.30 (C-10), 60.27 (C7), 56.41 (4′′-OCH_3_), 43.17 (C-9), 36.76 (C-5).

#### 3.1.14 Compound 14: specioside

White amorphous powder, C_24_H_28_O_12_; (+)-ESI-MS (*m*/*z*): 509.1 [M − H]^+^; ^1^H-NMR (CD_3_OD, 500 MHz), *δ*_H_ (ppm), *J* (Hz): 7.70 (1H, d, *J* = 16.0, H-7′′), 7.49 (2H, d, *J* = 8.5, H-2′′, H-6′′), 6.83 (2H, d, *J* = 8.5, H-3′′, H-5′′), 6.40 (1H, d, *J* = 16.0, H-8′′), 6.39–6.38 (1H, m, H-3), 5.18 (1H, d, *J* = 9.5, H-1), 5.04 (1H, d, *J* = 7.0, H-6), 5.00 (1H, dd, *J* = 6.0, 4.0, H-4), 4.81 (1H, overlap, H-1′), 4.20 (1H, d, *J* = 13.0, H-10), 3.95 (1H, dd, *J* = 12.0, 2.0, H-6′), 3.87 (1H, d, *J* = 13.0, H-10), 3.72 (1H, br s, H-7), 3.67 (1H, dd, *J* = 12.0, 6.5, H-6′), 3.45–3.42 (1H, m, H-3′), 3.37–3.35 (1H, m, H-5′), 3.34–3.27 (2H, m, H-2′, H-4′), 2.66–2.62 (1H, m, H-9), 2.61–2.59 (1H, m, H-5); ^13^C-NMR (CD_3_OD, 125 MHz), *δ*_C_ (ppm): 168.61 (C-9′′), 161.39 (C-4′′), 147.20 (C-7′′), 142.36 (C-3), 131.30 (C-2′′, C-6′′), 127.08 (C-1′′), 116.86 (C-3′′C-5′′), 114.61 (C-8′′), 102.94 (C-4), 99.71 (C-1′), 95.09 (C-1), 81.30 (C-6), 78.61 (C-5′), 77.69 (C-3′), 74.84 (C-2′), 71.75 (C-4′), 66.82 (C-8), 62.92 (C-6′), 61.29 (C-10), 60.26 (C-7), 43.19 (C-9), 36.74 (C-5).

#### 3.1.15 Compound 15: catalpol

White amorphous powder, C_15_H_22_O_10_; HR-ESI-MS (*m*/*z*): 385.1111 [M + Na]^+^ (calculated for C_15_H_22_O_10_Na 385.1089); ^1^H-NMR (CD_3_OD, 500 MHz), *δ*_H_ (ppm), *J* (Hz): 6.36 (1H, dd, *J* = 6.0, 1.5, H-3), 5.09 (1H, dd, *J* = 6.0, 4.5, H-4), 5.06 (1H, d, *J* = 10.0, H-1), 4.80 (1H, d, *J* = 8.0, H-1′), 4.13 (1H, d, *J* = 13.0, H-10), 3.93 (1H, m, H-6), 3.91 (1H, m, H-6′), 3.81 (1H, d, *J* = 13.0, H-10), 3.65 (1H, dd, *J* = 12.0, 6.0, H-6′), 3.47 (1H, s, H-7), 3.43 (1H, t, *J* = 9.0, H-3′), 3.33 (1H, m, H-5′), 3.29 (1H, m, H-4′), 3.26 (m, H-2′), 2.55 (1H, dd, *J* = 9.5, 7.5, H-9), 2.29 (1H, m, H-5); ^13^C-NMR (CD_3_OD, 125 MHz), *δ*_C_ (ppm): 141.73 (C-3), 104.01 (C-4), 99.70 (C-1′), 95.30 (C-1), 79.51 (C-6), 78.53 (C-5′), 77.65 (C-3′), 74.79 (C-2′), 71.70 (C-4′), 66.24 (C-8), 62.84 (C-6′), 62.57 (C-7), 61.51 (C-10), 43.57 (C-9), 39.05 (C-5).

### 3.2. Synthesis of catalpol derivatives

**Fig. 3:**
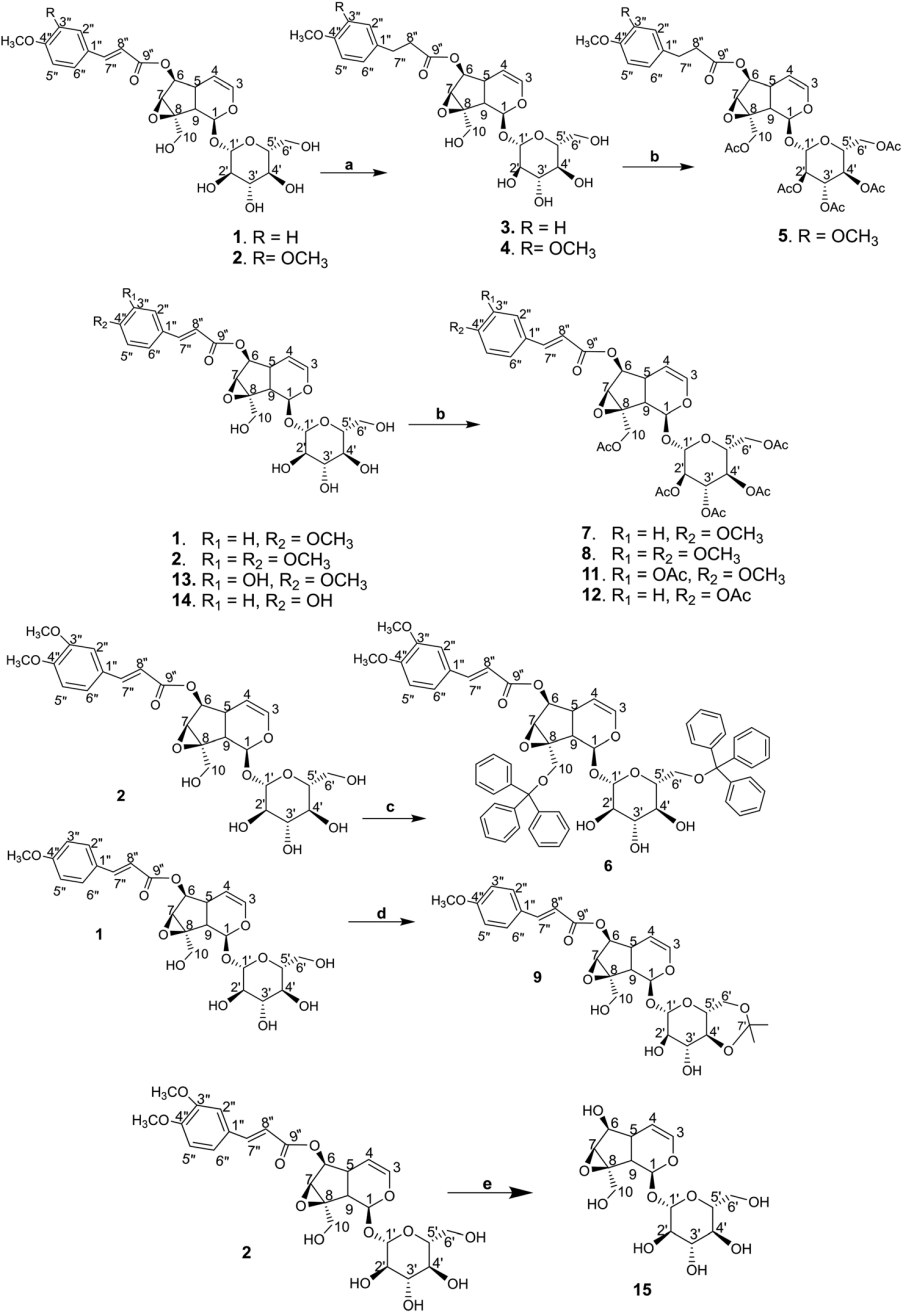
(a) NiCl_2_/MeOH, NaBH_4_, 0 °C, 30 minutes, then rt, 1 h; (b) acetic anhydride/Pyr, rt, 12 h; (c) triphenylmethyl chloride/CHCl_3_, Pyr, rt, 12 h; (d) 2,2-dimethoxypropane/DMF, TsOH·H_2_O, 50 °C; (e) NH_2_OH·HCl/(H_2_O/THF/EtOH) (1 : 1 : 1), TEA, 110 °C, 6 h.

The synthetic derivations are schematically summarised in Fig. 3. During our investigation on the phytochemical constituents of *Dolichandrone spathacea*, five iridoid compounds (1, 2, 10, 13, 14) have been isolated and structural elucidated. Four of them (1, 2, 13, 14), with the exception of 10, due to small amount, were converted into their derivatives by acetylation, reduction, O-alkylation, acetonisation and hydrolysation to obtain 3, 4, 5, 6, 7, 8, 9, 11, 12 and 15. Initially, the reduction of the olefin group in cinnamoyl and iridoid moiety in the structure of 1 and 2 was designed. The reduction was carried out by different kind of reductive reagents such as NaBH_4_/MeOH, LiAlH_4_/MeOH. However, many unwanted products were afforded under these condition. This might due to the strong base condition formed during the working up, causing a damage of iridoid ring. The trial of reduction with hydrogen catalyzed by Pd/C did not work, even though the reaction was heated to high temperature. We therefore used an additional catalyst Lewis acid NiCl_2_·6H_2_O in combination with NaBH_4_ reagent. The reaction was carried out under mild condition at 0 °C for 30 minutes. In this case, the products 3, 4 were obtain in good yield (83% and 80.6%, respectively). Interestingly, this reductive condition gave regioselective reduction only at olefinic bond of cinnamoyl group, but not at the Δ-3,4-double bond of iridoid ring. The dihydro derivative 4 was continuously acetylated with acetic anhydride in pyridine, giving the corresponding pentaacetate 5 in 80% yield. In order to investigate the effect of free hydroxyl groups on hyperglycemic activity in catalpol iridoids, compound 2 was *O*-alkylated with triphenylmethyl chloride and compound 1 was reacted with 2,2-dimethoxypropane/TsOH·H_2_O to provide product 6 and 9, respectively. The trityl groups were attached only to the primary oxymethylene groups at C-10 in catalpol ring and at C-6′ in glucose unit as well. In case of 9, cyclic ketal was formed only at C-4′ and C-6′ of the glucose unit, due to the steric hindrance of the iridoid and cinnamoyl moiety. Iridoids 1, 2, 13 and 14 were reacted with acetic anhydride in pyridine to give the corresponding acetyl products 7, 8, 11 and 12. To find out the role of cinnamoyl moiety, this moiety was tried to remove under different basic condition such as NaOH, LiOH, or KOH. However, these conditions gave many unwanted products. Therefore, an another milder basic condition using NH_2_OH. HCl in TEA was applied to obtain the hydrolysation derivative 15 (catalpol) from compound 2.

### 3.3. Structural elucidation

Elucidations of the structures for compounds 1, 2 and 13, 14 using spectral methods as FT-IR, MS, HR-MS, NMR (1D and 2D) were reported in ref. 24–29. In-detail data for the characterisations of each compound is provided in ESI† for in-depth reference and cross-checking.

**Structural elucidation of compound**10: The ^1^H and ^13^C NMR spectra of 10 exhibit typical signals for a catalpol moiety quite similar to that of compounds 1, 2, 13 and 14. The side chain seems to be a monoterpenoid with ten carbon signals in the ^13^C NMR spectrum, among them 2,3-substituted double bonds at *δ*_C_ (ppm): 144.15 (C-3′′), 138.37 (C-6′′), 128.60 (C-2′′), 125.74 (C-7′′), one ester carbonyl (*δ*_C_ 169.34, C-1′′), one oxymethylene (54.37, C-8′′) and two vinylic methyls (*δ*_H_ 1.77/*δ*_C_ 12.47: C-9′′; *δ*_H_ 1.60/*δ*_C_ 16.16: C-10′′), the linkage between the side chain and the catalpol moiety is determined as C-6 to C-1 by the correlation of signals at *δ*_C_ 169.34 (C-1′′) to *δ*_H_ 4.85 (H-6) in the HMBC spectrum. The structure of 10 is elucidated using its 1D and 2D NMR spectra. Its ESI-MS spectrum strongly supports this evidence by the pseudomolecular ion peaks at *m*/*z* 551.1 [M + Na]^+^ and *m*/*z* 563.0 [M + 2H_2_O–H]^−^ in positive and negative mode. In comparison with the reported data,^37^ compound 10 is determined as nemoroside, C_25_H_36_O_12_.

The hydrogenation of the main iridoid components 1 and 2 led to the formation of the dihydro product 3 and 4. The reduction occurs only at the double bond of cinnamoyl moiety of 1 and 2, evidenced by the appearance of two methylene at *δ*_H_/*δ*_C_ 2.91 (t, *J* = 7.5 Hz, H-7′′)/31.15 (C-7′′) and 2.69 (t, *J* = 7.5 Hz, H-8′′)/37.04 (C-8′′) for 3 and 2.93 (t, *J* = 7.5 Hz, H-7′′)/31.60 (C-7′′) and 2.72 (t, *J* = 7.5 Hz, H-8′′)/36.96 (C-8′′) for 4. The signals for the double bond in the iridoid ring (C-3 and C-4) are still remained in the NMR spectra of 3 and 4. Compound 5 was the acetylated product of 4. Five acetyl groups are formed in the structure of 5, deducing by the appearance of five acetyl methyl singlet at *δ*_H_/*δ*_C_ 2.00, 2.03, 2.04, 2.10, 2.13 (each 3H, s)/20.51, 20.54, 20.57, 20.71, 20.75 as well as the observation of five carbonyl groups at *δ*_C_ 172.64, 172.45, 171.60, 171.14, 170.92. Similarly, the totally acetylation of hydroxyl groups in 11 and 8 is confirmed by the signals of five acetyl groups in the NMR spectra of these compounds. NMR spectra of 7 and 12 indicate six acetyl groups corresponding to the acetylation of six hydroxyl groups in their structures. The cleavage of the cinnamoyl moiety is determined by the disappearance of the signal of cinnamoyl group in the NMR spectra of 15. The highfield chemical shift of H-6/C-6 at *δ*_H_/*δ*_C_ 3.93/79.51, compared to those of 2 (H-6/C-6 at *δ*_H_/*δ*_C_ 5.05–5.00/79.65), is due to the free hydroxyl group forming after hydrolysation.

**Fig. 4:**
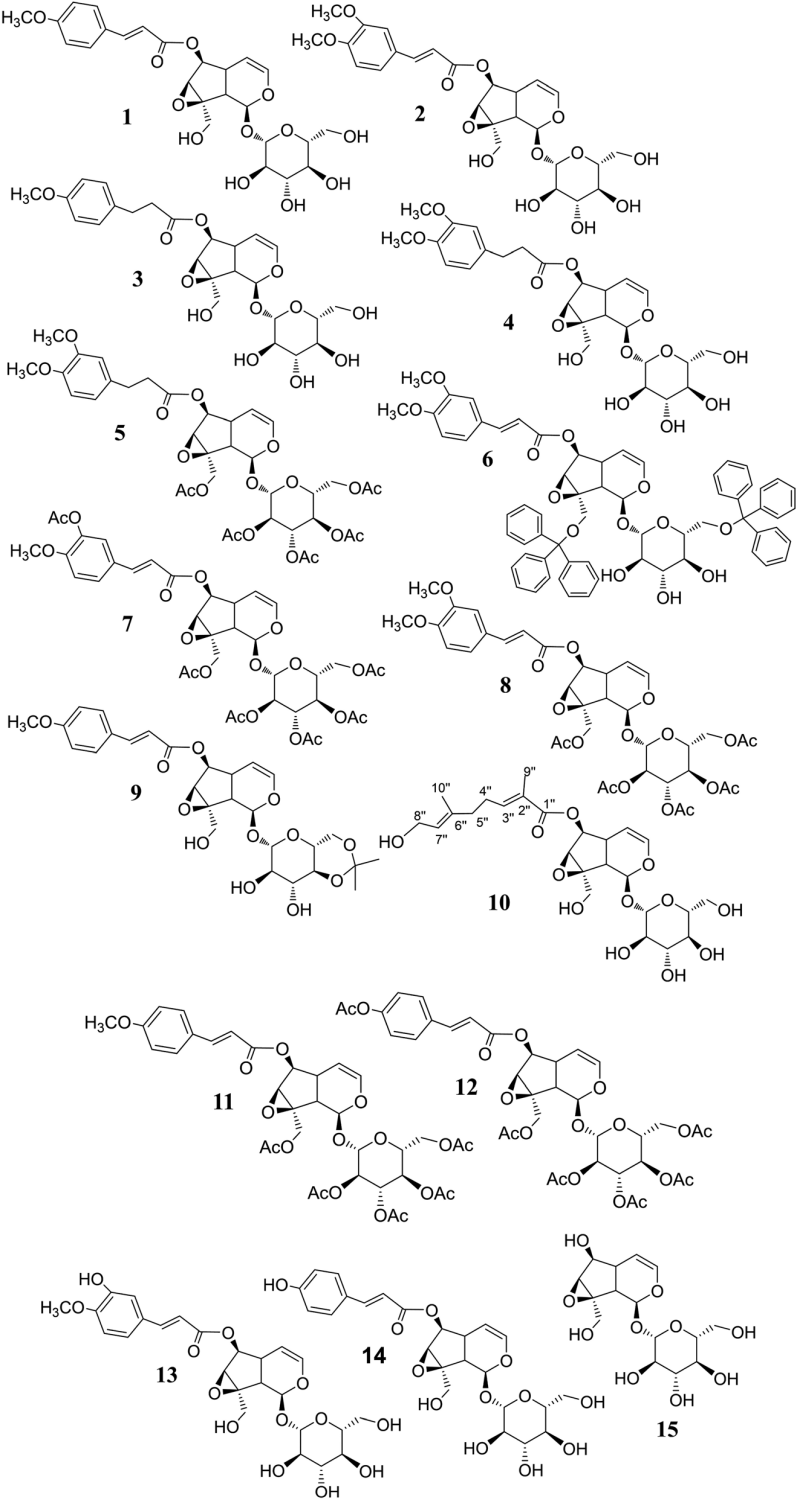
The isolated (1, 2, 10, 13, 14) and synthetic (3–9, 11, 12, 15) iridoid compounds.

All the elucidated structural formulae are presented in Fig. 4. To the best of our knowledge, the structures elucidated regarding compounds 3, 4, 5, 6, and 9 are in respect of new semi-synthesised iridoid-derivatives that found having unreported in the literature, given our reachable referencing. The HR-ESIMS spectra of all the new synthetic compounds in Section 3.1 (characterisation) also further confirmed the elucidation of their structure.

### 3.4. Simulation of enzyme inhibitability

Static interaction between the studied inhibitory agents (iridoid compounds and referenced drugs) and the targeted proteins (3W37 and 3AJ7) was simulated by molecular docking technique. List of descriptive symbols are given in ESI† (Section 2.3).

Overall, preliminary screening groups all the iridoid compounds into two inhibitability-based categories; in detail, 1, 2, 3, 4, 5, 9, 10, 14, and 15 are considered performing elevated inhibitory effects in comparison to those of 6, 7, 8, 11, 12, and 13. This is regardless of the targeted proteins, either 3W37 or 3AJ7. The justification is due to referencing of corresponding simulations on selected commercial drugs D1, D2, and D3.

#### 3.4.1 Molecular docking simulation for iridoid-3W37

**Fig. 5:**
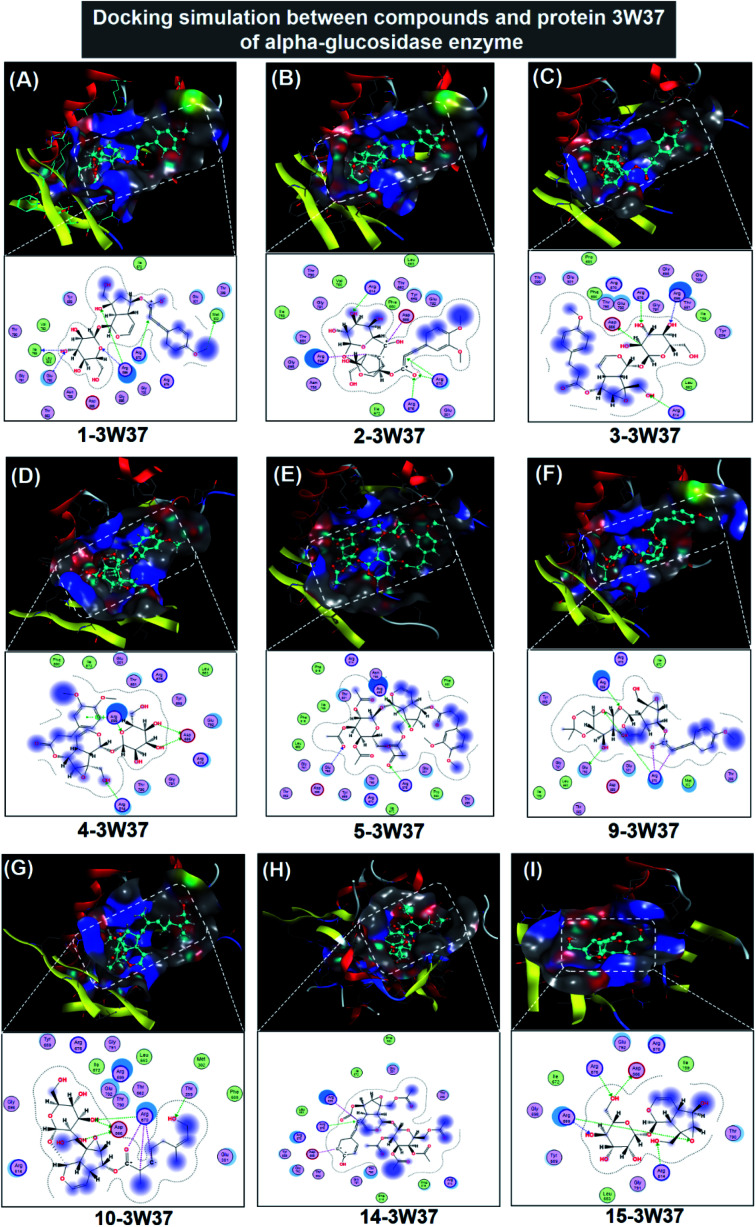
Visual presentation and in-pose interaction map of ligand-3W37 inhibitory complexes: (A) 1-3W37, (B) 2-3W37, (C) 3-3W37, (D) 4-3W37, (E) 5-3W37, (F) 9-3W37, (G) 10-3W37, (H) 14-3W37, and (I) 15-3W37.

Regarding 1, 2, 3, 4, 5, 9, 10, 14, and 15, the docking results related to their inhibitory complexes with protein 3W37 are summarised in Table 1 (in-detail parameters included in ESI† – Section 2.4) and the complex structures are virtually represented in Fig. 5. The former provides inhibition parameters (DS value and RMSD), and in-depth bonding details building up the host complexes; while, the latter visualises the optimised orientation of each ligand in an active site of protein 3W37. DS value for 2-3W37 registers −15.1 kcal mol^−1^, the lowest value calculated. This means the complex is at the lowest free-energy level, thereby most stable. In detail, compound 2 seems to exhibit great affinity with a specific amino acid in 3W37 structure, aka. arginine, by creating seven ionic and four H-acceptor bonds with four different arginine molecule, *i.e.* Arg 814, Arg 670, Arg 676, and Arg 699, and one ionic bond with Asp 666. They altogether compose −39.2 kcal mol^−1^ in respect of hydrophilic attraction, of which bonding lengths are in 2.78–3.95 Å. This stability is followed by those of 10-3W37 and 1-3W37 with the DS values −14.7 and −14.1 kcal mol^−1^, respectively, both composed by seven hydrogen bonds to a variety of amino acid types. However, their hydrophilic energy distribution sees different patterns. While each 10-3W37 ligand–amino acid hydrogen bond contributes rather evenly to the total hydrophilic free energy of −11.9 kcal mol^−1^, C-Agr 670 H-acceptor bonding holds −8.7 kcal mol^−1^ of free energy accounting for over a haft of 1-3W37 total figure, −17.2 kcal mol^−1^. The former contains the binding performances in distance 2.71–3.30 Å, while the corresponding figures for the latter are slightly elevated, *i.e.* 2.75–3.80 Å. This also justifies the slight outweighing of 10-3W37 stability, previously evaluated by DS values. On the other hand, hydrophobic binding contributes fourteen van der Waals interactions to each 2-3W37, 10-3W37 or 1-3W37 inhibitory structure. Otherwise, 3-3W37, 4-3W37, 5-3W37, 9-3W37, 14-3W37, and 15-3W37 seem less stable given their inferior DS values, *i.e.* −12.9, −12.3, −11.6, −13.3, −13.0, and −12.3 kcal mol^−1^. Therefore, the inhibitability of the regarded compounds accords with an order: 2 > 10 > 1 > 9 > 14 > 3 > 4 = 15 > 5. In addition, all RMSD values registering under 2 Å indicates that the associated complexes are tolerable as biological rigid bodies. In particular, although exhibiting a moderate DS value, the average distance between neighbouring atoms in 15-3W37 structure is significantly short given its RMSD value of 0.67 Å. This suggests a ligand-bound conformation if the inhibitory structure is formed. The reason might relate to ligand-pose spatial complementarity. Also, illustrative description for the intermolecular interactions detected in each active site is projected on a 2D diagram for each complex. It is also clear that all iridoid ligands are in high exposure to their targeted protein shown by densely blurred-violet zones, while long and continuous proximity contour bounding large in-pose areas likely indicate unintermittent interactability between the docking agents. Finally, visual observations reinforce the inhibitability since the docked sites are still spacious after the inhibition of 1, 2, 3, 4, 5, 9, 10, 14, or 15.

**Table 1:** Molecular docking simulation results for inhibitory complexes between the compounds and the protein 3W37 with amino acids: 1-3W37, 2-3W37, 3-3W37, 4-3W37, 5-3W37, 6-3W37, 7-3W37, 8-3W37, 9-3W37, 10-3W37, 11-3W37, 12-3W37, 13-3W37, 14-3W37, and 15-3W37^a^

Ligand–protein complex			Number of interaction	
Name	DS	RSMD	Hydrogen bond	van der Waals
1-3W37	−14.1	1.47	7	14
2-3W37	−15.1	1.96	12	14
3-3W37	−12.9	1.33	4	14
4-3W37	−12.3	1.72	6	11
5-3W37	−11.6	1.40	3	18
6-3W37	—	—	—	—
7-3W37	−8.1	1.84	6	16
8-3W37	−10.5	1.76	6	18
9-3W37	−13.3	1.69	6	9
10-3W37	−14.7	1.52	7	14
11-3W37	−10.1	1.46	4	16
12-3W37	−7.8	1.41	7	18
13-3W37	−8.8	1.47	7	18
14-3W37	−13.0	1.01	4	13
15-3W37	−12.3	0.67	5	9

a DS: docking score energy (kcal mol^−1^); RMSD: root-mean-square deviation (Å).

In respect of 7, 8, 11, 12, and 13, DS values associated with their ligand-3W37 inhibitory complexes register over −10.5 kcal mol^−1^, thus deemed as less competent inhibitors towards the protein. The respective calculated RMSD varies between 1.41 and 1.84 Å, acceptable values for a firm conformation. Furthermore, in-depth results of the inhibitory complexes are also summarised in Table 1 while their corresponding 3D- and 2D-virtual renderings are included in ESI† (Section 2.1). In exceptional, 6 was found not able to perform inhibition towards protein 3W37.

#### 3.4.2 Molecular docking simulation for iridoid-3AJ7

**Fig. 6:**
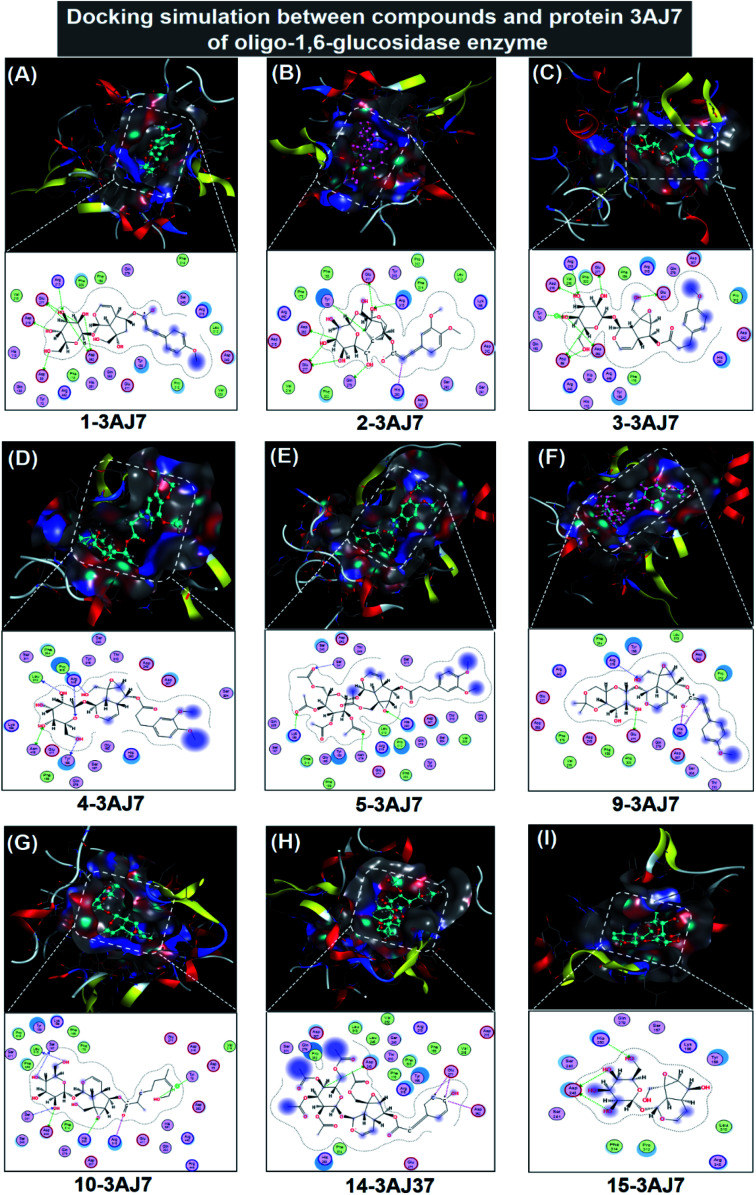
Visual presentation and in-pose interaction map of ligand-3W37 inhibitory complexes: (A) 1-3AJ7, (B) 2-3AJ7, (C) 3-3AJ7, (D) 4-3AJ7, (E) 5-3AJ7, (F) 9-3AJ7, (G) 10-3AJ7, (H) 14-3AJ7, and (I) 15-3AJ7.

Regarding 1, 2, 3, 4, 5, 9, 10, 14, and 15, their inhibitory complexes with protein 3AJ7 are virtually presented in Fig. 6 and their respective docking results are summarised in Table 2 (in-detail parameters included in ESI† – Section 2.4). The former predicts the ligand conformation and orientation in an active site of the protein; while, the latter provides in-depth docking parameters of the complexes, including DS value, RMSD, and interaction detail. The highest DS value obtained is −14.5 kcal mol^−1^, responsible for the stability of 14-3AJ7 by representing the lowest free-energy level. However, there are only five hydrophilic interactions detected as the complex forms, including four ionic and one H-donor bonds. The targeted amino acids are aspartic acid (Asp 242, Asp 352) and glutamic acid (Glu 277), which create hydrogen-bond binding with 14 carbon atoms in distance 2.93–3.82 Å. The total hydrophilic potential calculated is −9.9 kcal mol^−1^, insignificantly. Hence, 14-3AJ7 stability seems accumulated from van der Waals forces arisen between its two components as they include twenty different 3AJ7 amino acids in involvement. This is followed by 2-3AJ7 of which stability is expressed *via* −13.5 kcal mol^−1^ of DS value. The value is likely cultivated from both hydrophilic and hydrophobic contribution. There are nine hydrogen bonds formed between molecule 2 and 3AJ7 amino acids in distance 2.58–4.0 Å, accounting for −28.8 kcal mol^−1^ of the former free energy; while, the latter is from van der Waals attraction constituted by fifteen different in-pose amino acids. In contrast, 10-3AJ7 binding affinity is likely based more on hydrophobic interactions than on hydrophilic counterparts as the former includes twenty-one amino acids while the latter results in a total energy just −7.5 kcal mol^−1^. Its DS value registers −12.4 kcal mol^−1^, the third highest figure. Otherwise, DS values of 1-3AJ7, 3-3AJ7, 4-3AJ7, 5-3AJ7, 9-3AJ7, and 15-3AJ7 are −11.4, −10.6, −10.2, −11.7, −11.5, and −11.0 kcal mol^−1^, respectively. Therefore, the ligand inhibitability towards protein 3AJ7 follows an order: 14 > 2 > 10 > 5 > 1 = 9 > 15 > 3 > 4. In addition, by possessing RMSD value under 2 Å, all the inhibitory complexes are accepted performing biological rigidity. Moreover, visual illustration of interacting activity between the ligand and in-pose amino acids clarify the predominance of hydrophobic interactions in the establishment of ligand-3AJ7 inhibitory structures. This is justified by a myriad of amino acids presenting around the docked compounds. Resembling to observations on ligand-3W37, spaciousness inside the active sites are highly conducive to motion of 1, 2, 3, 4, 5, 9, 10, 14, or 15 when inhibition processes are on, thus their inhibitability towards protein 3AJ7 affirmed.

**Table 2:** Molecular docking simulation results for inhibitory complexes between the compounds and the protein 3AJ7 with amino acids: 1-3AJ7, 2-3AJ7, 3-3AJ7, 4-3AJ7, 5-3AJ7, 6-3AJ7, 7-3AJ7, 8-3AJ7, 9-3AJ7, 10-3AJ7, 11-3AJ7, 12-3AJ7, 13-3AJ7, 14-3AJ7, and 15-3AJ7^a^

Ligand–protein complex			Number of interaction	
Name	DS	RSMD	Hydrogen bond	van der Waals
1-3AJ7	−11.4	1.14	7	20
2-3AJ7	−13.5	1.64	9	15
3-3AJ7	−10.6	1.71	8	16
4-3AJ7	−10.2	1.79	6	15
5-3AJ7	−11.7	1.80	4	22
6-3AJ7	—	—	—	—
7-3AJ7	−9.3	1.98	3	22
8-3AJ7	−8.8	1.90	4	20
9-3AJ7	−11.5	1.74	4	17
10-3AJ7	−12.4	1.33	7	21
11-3AJ7	−6.3	1.99	4	15
12-3AJ7	−6.4	1.06	6	32
13-3AJ7	−7.5	1.99	4	27
14-3AJ7	−14.5	1.71	5	20
15-3AJ7	−11.0	1.12	3	10

a DS: docking score energy (kcal mol^−1^); RMSD: root-mean-square deviation (Å).

In respect of 7, 8, 11, 12, and 13, DS values associated with their ligand-3AJ7 inhibitory complexes are also included in Table 2. The respective figures are −9.3, −8.8, −6.4, and −7.5 kcal mol^−1^, clearly expressing inferiority of overall stability. Their RMSD values are all under 1.99 Å. Also, their corresponding 3D morphology and 2D interaction map are given in ESI† (Section 2.2). Similar to investigation on ligand-3W37, failure was also recorded in an attempt to establish a 6-3AJ7 inhibitory structure. No possible intermolecular interaction is given, thus no inhibitability of molecule 6 towards protein 3AJ7 ensuing. The reason for both could be referred to spatial restraint due to 6 structural bulkiness and branching, which possibly prohibit its entry into the host active site. However, more in-depth investigations, either theoretical or experimental, are needed to reach a more accurate conclusion.

#### 3.4.3 Molecular docking simulation for drug–protein complexes

**Fig. 7:**
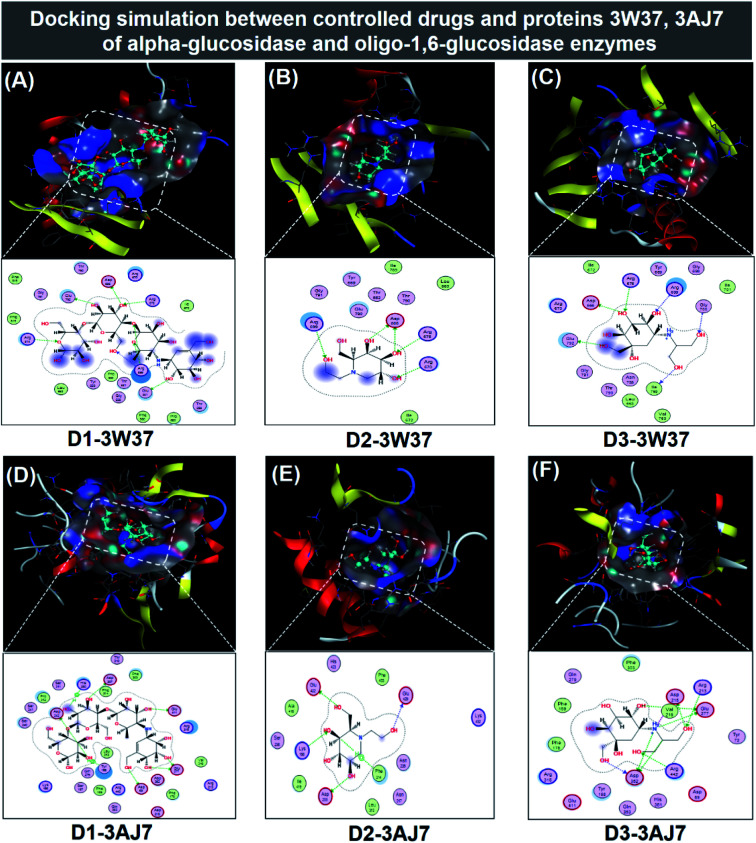
Visual presentation and in-pose interaction map of ligand-protein inhibitory complexes: (A) D1-3W37, (B) D2-3W37, (C) D3-3W37, (D) D1-3W37, (E) D2-3AJ7, and (F) D3-3AJ7.

The inhibitability of three commercial medicines acarbose (D1), miglitol (D2), and voglibose (D3) towards the carbohydrate-hydrolase proteins (3W37 and 3AJ7) were investigated for referencing purposes. The inhibitions are virtually rendered in Fig. 7, while in-detail docking results are summarised in Table 3 (in-detail parameters included in ESI† – Section 2.4). All drugs seem fully fitting with their inhibiting structural topography given their continuous proximity contours. This indicates high degree of complementarity. Even quasi-macromolecule D1 still fulfils this topographical fitting. However, different duo-systems see different patterns of intermolecular interaction between the adducts. In particular, D1 is able to create a myriad of interactions with its in-pose amino acids regardless of either the host protein or types of binding, thus conducive to its prominent inhibitability in comparison to the others. DS values of D1-3W37 and D1-3AJ7 are −14.2 and −15.3 kcal mol^−1^, respectively. These were followed by the corresponding figures for D3-protein structures, *i.e.* −12.7 and −13.6 kcal mol^−1^, respectively. Calculation on D2-protein inhibition results in relatively inferior DS values, *i.e.* 11.1 kcal mol^−1^ for D2-3W37 and −10.7 kcal mol^−1^ for D2-3AJ7. Nevertheless, these complexes are considered possessing ligand-bound conformations given their short average distance between in-active atoms represented by RMSD of 0.68 and 1.03 Å. Other RMSD values register under 2 Å and most binding length are predicted under 3 Å. The inhibitability of the medicines towards carbohydrate-hydrolase proteins (3W37 and 3AJ7) can be briefed as: acarbose (D1) > voglibose (D3) > miglitol (D2). In referencing, there are no significant differentials of the inhibitory indicators between these commercial medicines and the selective iridoid compounds. This implies promising applicability of these substances as carbohydrate-hydrolase inhibitors in particular and in diabetes treatment in general. 1, 2, 10 regarding 3W37 inhibition and 2, 10, 14 regarding 3AJ7 inhibition are even highly competent.

**Table 3:** Molecular docking simulation results for inhibitory complexes between the drugs and the two protein 3W37, 3AJ7 with amino acids: D1-3W37, D2-3W37, D3-3W37; D1-3AJ7, D2-3AJ7, and D3-3AJ7^a^

Ligand–protein complex			Number of interaction	
Name	DS	RSMD	Hydrogen bond	van der Waals
D1-3W37	−14.2	1.58	8	14
D2-3W37	−11.1	0.68	5	8
D3-3W37	−12.7	1.52	6	10
D1-3AJ7	−15.3	1.79	9	20
D2-3AJ7	−10.7	1.03	5	8
D3-3AJ7	−13.6	0.94	12	12

a DS: docking score energy (kcal mol^−1^); RMSD: root-mean-square deviation (Å).

### 3.5. Screening of physicochemical and pharmaceutical compatibility

Several properties of studied ligands are summarised in Table 4 in order to screen their physicochemical and pharmaceutical compatibility based on Lipinski's rule of five. The most promising candidates, aka. 1, 2, and 10, weigh *ca.* 500 amu and possess negative dispersion coefficients (log *P* and log *S*). Only 10 creates less than both ten hydrogen bond acceptors and five hydrogen bond donors regardless of the targeted protein, either 3W37 or 3AJ7. This preliminarily suggests 10 as the most potential compound for oral administration in attempt to tackle diabetes. However, other promising candidates should be in great consideration. First, 507.1 amu 14 might still be suitable since the commercial product Acarbose weigh 645.6 amu, over Lipinski's first criterion. The highly efficacy-demonstrated medicine also forms seven hydrogen bond donor with protein 3AJ7. This justifies the applicability of 1 and 2. In addition, it is noticeable that their polarisability is over 10 Å^3^, indicating high polarisation. The property is of significance because it is highly conducive to protein inhibition as the polypeptide molecule is made of polarised amino acids. Therefore, the selective iridoid compounds, especially 10, are likely compatible with pharmaceutical applications in physiological medium. However, *in vitro* and *in vivo* experiments are required in order to confirm the computational predictions.

**Table 4:** Summarisation of physiochemical properties of studied compounds including docking score energy values (DS, kcal mol^−1^), molecular mass (*M*_a_, amu), polarizability (Å^3^), volume or size (Å), log *P*, log *S* and the total of hydrogen bonds of the 15 potential substances docked with the two proteins 3W37 and 3AJ7

Compound (ligand)	DS	Mass	Polarizability	Volume	Dispersion coefficients		Hydrogen bond (3W37/3AJ7)	
					Log *P*	Log *S*	H-acceptor	H-donor
1	−12.8	522.5	49.3	559.5	0.8	−2.0	4/1	2/6
2	−14.3	552.5	51.8	595.3	1.2	−1.8	4/2	0/6
3	−11.8	524.5	45.3	561.2	1.1	−1.6	3/1	1/6
4	−11.3	554.5	50.7	593.9	1.5	−1.5	4/2	2/4
5	−11.7	764.7	49.6	814.6	1.8	−4.8	3/4	0/0
6	0.0	1037.2	29.3	1105.6	10.4	−13.8	0/0	0/0
7	−8.7	790.7	33.8	832.7	1.9	−5.2	4/1	0/1
8	−9.7	762.7	37.2	810.7	2.1	−5.1	3/1	0/0
9	−12.4	562.6	48.4	604.5	1.0	−3.4	2/1	1/1
10	−13.6	528.6	51.1	568.1	1.2	−1.4	2/2	2/3
11	−8.2	732.3	31.5	785.1	2.4	−5.3	2/1	0/2
12	−7.1	760.2	32.7	868.9	1.8	−5.4	3/2	1/0
13	−8.2	538.0	32.1	801.8	2.0	−4.9	2/1	0/2
14	−13.8	508.4	47.4	518.7	2.1	−4.9	1/0	0/1
15	−11.7	362.3	31.4	162.5	3.4	−0.9	4/1	1/2
D1	−14.8	645.6	60.9	613.9	2.7	−0.8	5/0	3/7
D2	−10.9	207.2	20.8	235.9	2.3	−0.5	3/1	2/3
D3	−13.2	267.3	26.0	280.8	2.3	−0.2	3/2	3/6

In summary, the results obtained altogether are likely to justify the applicability of the novel-synthesised iridoid compounds in cytological in general, and for inhibition of carbohydrate-hydrolase proteins (3W37 and 3AJ7) in particular.

### 3.6. α-Glucosidase inhibition

The enzyme inhibitory activity of fourteen compounds (excluding 15) were assayed towards α-glucosidase. Acarbose was used as a reference whose IC_50_ is determined registering 285.00 μM in the resembling assay conditions. The obtained results are shown in Table 5, which are relatively consistent with those predicted by computational simulation. In detail, 10 > 2 > 14 > 13 > 1 assemble the most effective inhibitors towards α-glucosidase whose attained IC_50_ values register lower than 25 μM. Compound 10 shows its marked inhibition towards the enzyme with an IC_50_ value of 0.05 μM, higher than that of acarbose by 5700 times. This was followed by 2 and 14, both exhibiting significant α-glucosidase inhibition with IC_50_ value 7.24 and 8.65 μM, respectively. The corresponding figure for 13 is 20.84 μM and for 1 is 24.89 μM. The predominance of 10 inhibitability seems signifying the role of (2*E*,6*E*)-8-hydroxy-2,6-dimethyl-2,6-octadienoate moiety as it is the only compound containing this functional group. Its flexibility and polarity acquired from an octadienoate configuration might be advantageous to structural transformation, which in turn conducive to either protein-site entrance, polar–polar interaction, or in-pose topographical fitting. However, in-depth investigations are still required in order to reach more accurate view. Although predicted exhibiting insignificant inhibitability towards α-glucosidase molecule (3W37) by molecular docking simulation, experimental results reflect otherwise. On the other hand, 9 also sees noticeable simulation-experiment inconsistency, in which the compound is expected to perform pronounced α-glucosidase inhibition.

**Table 5:** Experimental α-glucosidase inhibitory activity of catalpol iridoids^a^

Compound	IC_50_ (μM)
1	24.89
2	7.24
3	NA
4	NA
5	NA
6	224.78
7	167.76
8	NA
9	NA
10	0.05
11	NA
12	71.56
13	20.84
14	8.65
Arcabose (D1)	285.00

a NA: no activity (IC_50_ > 390 μM).

Compounds 3, 4, and 5, without double bond at C-7′′ and C-8′′, are inactive towards α-glucosidase observed from enzyme assays. This is relatively consistent with the computational prediction as these compounds are not expected to be effective 3W37 inhibitors by the simulation. Therefore, double bond at C-7′′ and C-8′′ could be subjected for further investigations on α-glucosidase inhibition. Regarding acetylation, the corresponding derivatives are either inactive including 8 and 11, or unfavoured such as 7 (IC_50_ 167.76 μM) and 12 (IC_50_ 71.56 μM) as α-glucosidase inhibitors. The corresponding computing also predicts this unfavourableness. The lessening of polarity, caused by total acetyl-hydroxyl substitution, might reason this, thus justifying the important role of catalpol hydroxyl groups to the inhibition. In addition, computationally expected possessing no inhibitability towards 3W37, 6 performs assaying inhibition towards α-glucosidase with a notably high IC_50_ value of 224.78 μM. This reinforces the implication that two bulky triphenyl methyl groups at C-10 and C-6′ seem to limit the host-molecule entrance into the protein sites.

From the obtained results, several brief views on structural activity relationship of iridoid catalpol derivatives on α-glucosidase inhibition can be speculated. Acetylation of the hydroxyl groups at C-10 and glucose unit leads to reduced activity. Reduction of the double bond in the cinnamoyl moiety as well as locking the hydroxyl groups at C-6′ and C-4′ of the glucose unit by a cyclic acetal group totally deteriorate the activity. The presence of a hydroxyl group at C-4′′ of cinnamoyl moiety is likely to play an important role of activity, much better than the corresponding property for methoxy or acetyl group. The attachment of two bulky triphenylmethyl groups at C-10 and C-6′ strongly reduces the activity, 32-fold decrease of inhibition. Finally, the location of a long, linear unsaturated alkyl ester chain at C-6 (octadienonate) instead of cinnamoyl moiety significantly rises the α-glucosidase inhibitory activity. Nemoroside (10) is the most active compound with an IC_50_ value of 0.05 μM, 5.700 times better than acarbose. This could be explained by the flexibility of its bonding structure, thus conducive to its topological fitness while inside the inhibitory holes.

## 4. Conclusions

This study suggests a promising approach to the use of iridoid chemotype from *Dolichandrone spathaceae* as α-glucosidase (protein 3W37) and oligo-1,6-glucosidase (protein 3AJ7) inhibitors. Five isolated natural iridoids and ten synthetic derivatives were successfully identified by structural elucidation. These include five new iridoid-derived structures not found having reported before in the literature, *i.e.*3, 4, 5, 6, and 9. Molecular docking simulation indicates that 1, 2, 3, 4, 5, 9, 10, 14, and 15 could perform elevated inhibitability towards both carbohydrate-hydrolase proteins. Regarding protein 3W37, five strongest predicted inhibitors accords with an order assemble 2 (DS – 15.1 kcal mol^−1^) > 10 (DS – 14.7 kcal mol^−1^) > 1 (DS – 14.1 kcal mol^−1^) > 9 (DS – 13.3 kcal mol^−1^) > 14 (DS – 13.0 kcal mol^−1^). In respect to 3AJ7, the corresponding order is 14 (DS – 14.5 kcal mol^−1^) > 2 (DS – 13.5 kcal mol^−1^) > 10 (DS – 12.4 kcal mol^−1^) > 5 (DS – 11.7 kcal mol^−1^) > 1 = 9 (DS – 11.5 kcal mol^−1^). Exceptionally, compound 6 is predicted unable to establish an inhibitory structure with any given proteins. Lipinski's rule of five suggests 10 as the most potential candidate for oral administration; while, 1, 2, and 14 are highly promising for pharmaceutical applications in physiological medium. All compounds possess high polarisability, which is suitable for inhibition of polarised protein molecules. Enzyme assays on α-glucosidase revealed that compound 10 is the most effective inhibitor with an IC_50_ value of 0.05 μM, higher than that of acarbose by 5700 times. It is followed by 1, 2, 13, and 14 in the order 10 (IC_50_ 0.05 μM) > 2 (IC_50_ 7.24 μM) > 14 (IC_50_ 8.65 μM) > 13 (IC_50_ 20.84 μM) > 1 (IC_50_ 24.89 μM), showing good consistency with the docking-based predictions and significant predominant in comparison to acarbose (IC_50_ 285.00 μM). Compound 6 performs unfavoured inhibitability in the *in vitro* bioassay with IC_50_ value of 224.78 μM. The results are considered highly advantageous to preparation of therapeutic medicines based on iridoid chemotype in an attempt to develop an alternative for diabetes treatment.

## Conflicts of interest

There are no potential conflicts of interest by the authors.

## Supplementary Material

RA-011-D1RA00441G-s001
