# Supplementary material for: Isolation, semi-synthesis, docking-based prediction, and bioassay-based activity of Dolichandrone spathacea iridoids: new catalpol derivatives as glucosidase inhibitors
Source: RSC Adv. 2021 Mar 23;11(20):11959–75. doi: 10.1039/d1ra00441g (PMC8696980; doi:10.1039/d1ra00441g)
Supplement: RA-011-D1RA00441G-s001 [file RA-011-D1RA00441G-s001.pdf]

Electronic Supplementary Material (ESI) for RSC Advances.
This journal is © The Royal Society of Chemistry 2021

# Isolation, semi-synthesis, docking-based prediction, and bioassay-based activity of *Dolichandrone spathacea* new catalpol derivatives as glucosidase inhibitors

**Tran Thi Phuong Thao[1,2,§], Thanh Q. Bui[3,§], Phan Tu Quy[4], Nguyen Chi Bao[5], Tran Van Loc[1,2], Tran Van Chien[1,2], Nguyen Linh Chi[1,2], Nguyen Van Tuan[2,6], Tran Van Sung[1,2]*, Nguyen Thi Ai Nhung[3]***

[1]Institute of Chemistry, Vietnam Academy of Science and Technology, 18 Hoang Quoc Viet Road, Cau Giay, Hanoi, Vietnam

[2]Graduate University of Science and Technology, Vietnam Academy of Science and Technology, 18 Hoang Quoc Viet Road, Cau Giay, Hanoi, Vietnam

[3]Department of Chemistry, University of Sciences, Hue University, Hue City, Vietnam

[4]Department of Natural Sciences & Technology, Tay Nguyen University, Buon Ma Thuot, Vietnam

[5]Hue University, Hue City, Vietnam

[6]Asean college of Medicine and Pharmacy, Trung Trac street, Van Lam district, Hung Yen province, Vietnam

[§]These authors contributed equally to this work

*Correspondence to:

Tran Van Sung (E-mail: tranvansungvhh@gmail.com)

Nguyen Thi Ai Nhung (E-mail: ntanhung@hueuni.edu.vn)

## SUPPORTING INFORMATION


| No. | Content | Page |
|---|---|---|
| **1** | **EXPERIMENTAL CHARACTERISATIONS** | **2** |
| 1.1 | Compound 1 | 2 |
| 1.2 | Compound 2 | 11 |
| 1.3 | Compound 3 | 20 |
| 1.4 | Compound 4 | 25 |
| 1.5 | Compound 5 | 30 |
| 1.6 | Compound 6 | 36 |
| 1.7 | Compound 7 | 42 |
| 1.8 | Compound 8 | 46 |
| 1.9 | Compound 9 | 51 |
| 1.10 | Compound 10 | 56 |
| 1.11 | Compound 11 | 67 |
| 1.12 | Compound 12 | 72 |
| 1.13 | Compound 13 | 77 |
| 1.14 | Compound 14 | 81 |
| 1.15 | Compound 15 | 84 |
| **2** | **COMPUTATIONAL VIRTUAL RENDERING** | **89** |
| 2.1 | Visual presentation and in-pose interaction map of ligand-3W37 inhibitory complexes | 89 |
| 2.2 | Visual presentation and in-pose interaction map of ligand-3AJ7 inhibitory complexes | 90 |
| 2.3 | Descriptive denotation for in-pose interactions projected by MOE 2015.10 molecular docking simulation | 91 |
| 2.4 | Molecular docking simulation results for inhibitory complexes | 92 |

## 1. EXPERIMENTAL CHARACTERISATIONS

### 1.1. Compound 1

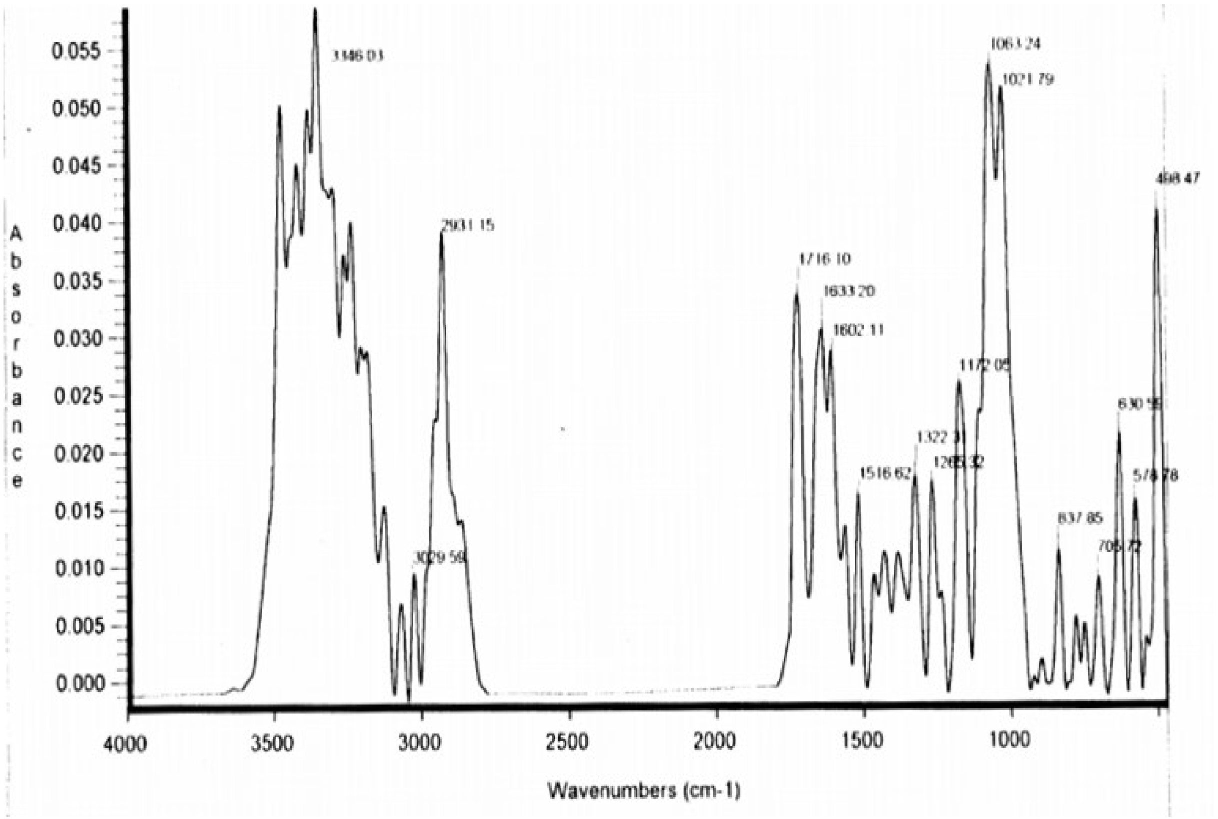


IR spectrum of compound **1**

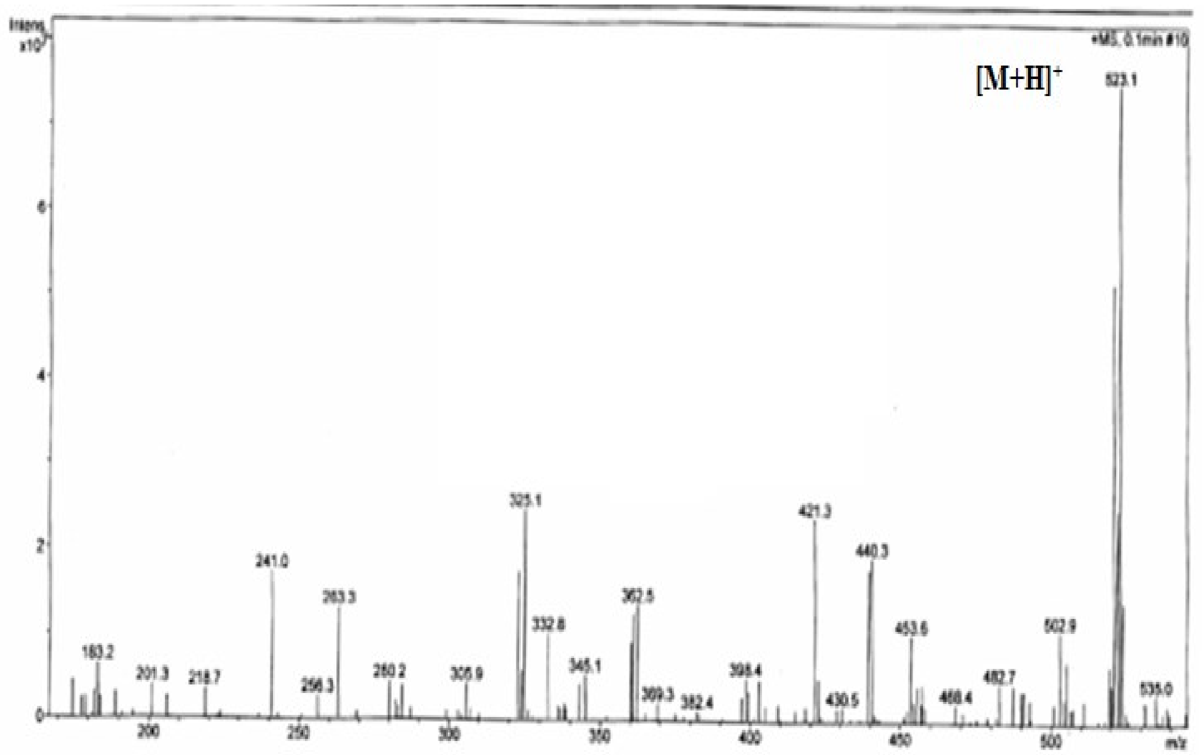


(+)-ESI-MS spectrum of compound **1**

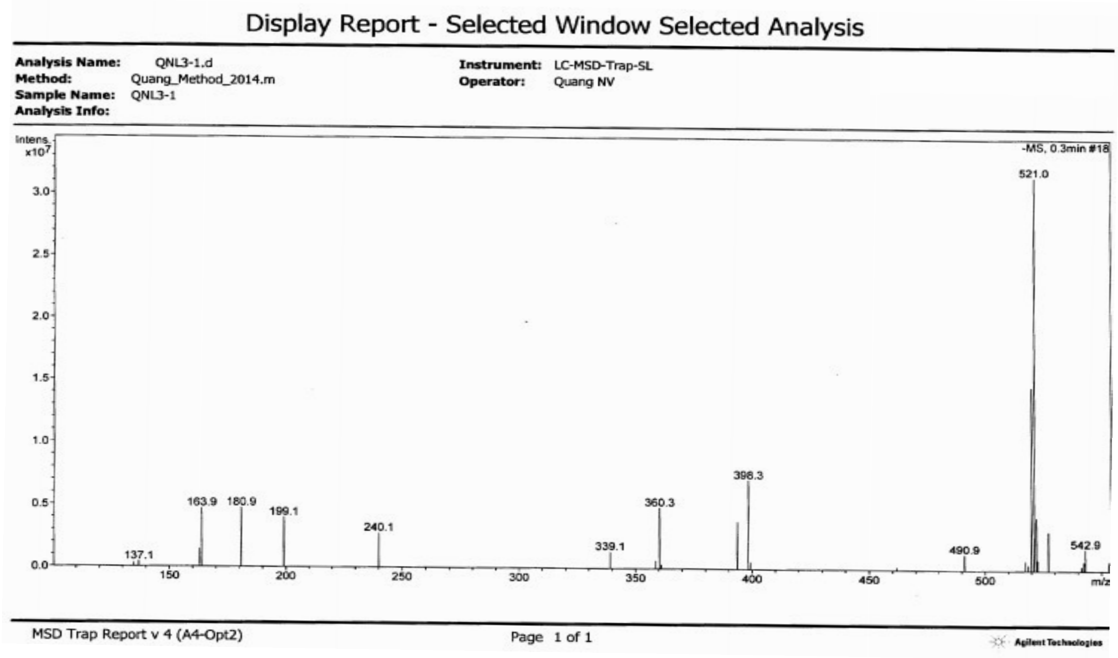


(-)-ESI-MS spectrum of compound **1**

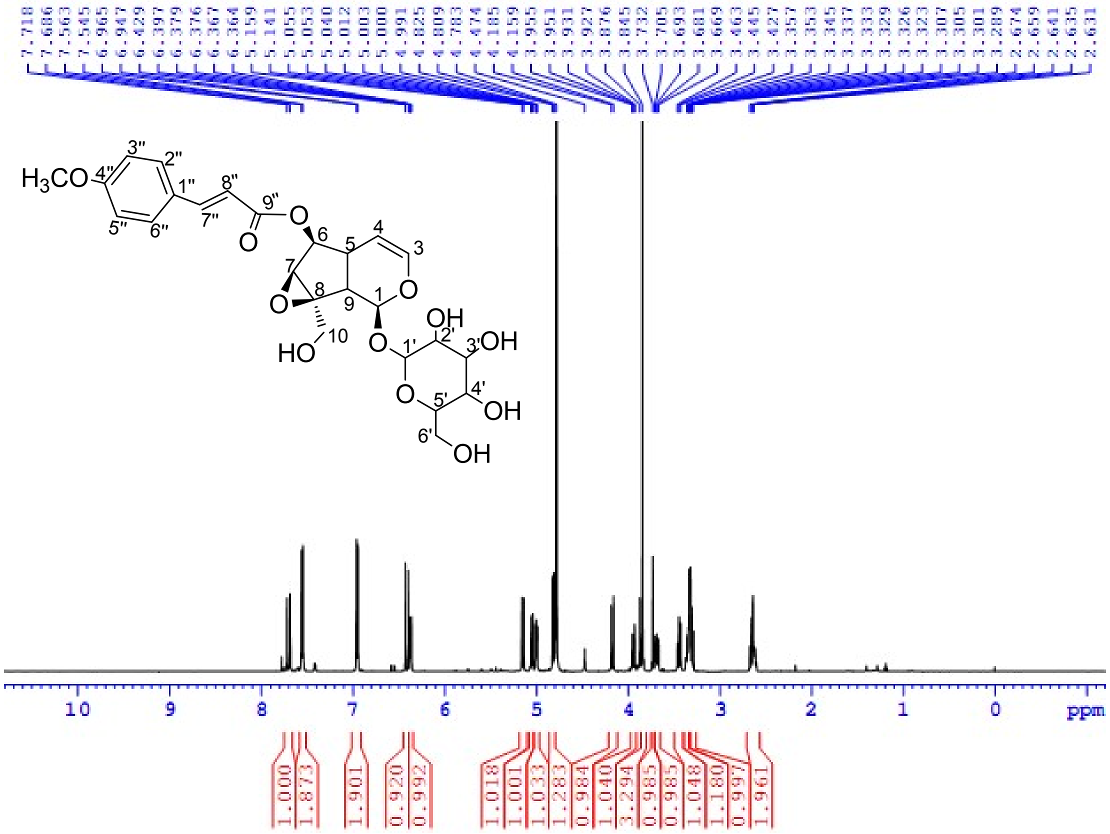

$^{1}$H-NMR spectrum (500 MHz, $CDCl_3$ + $CD_3OD$) of compound **1**

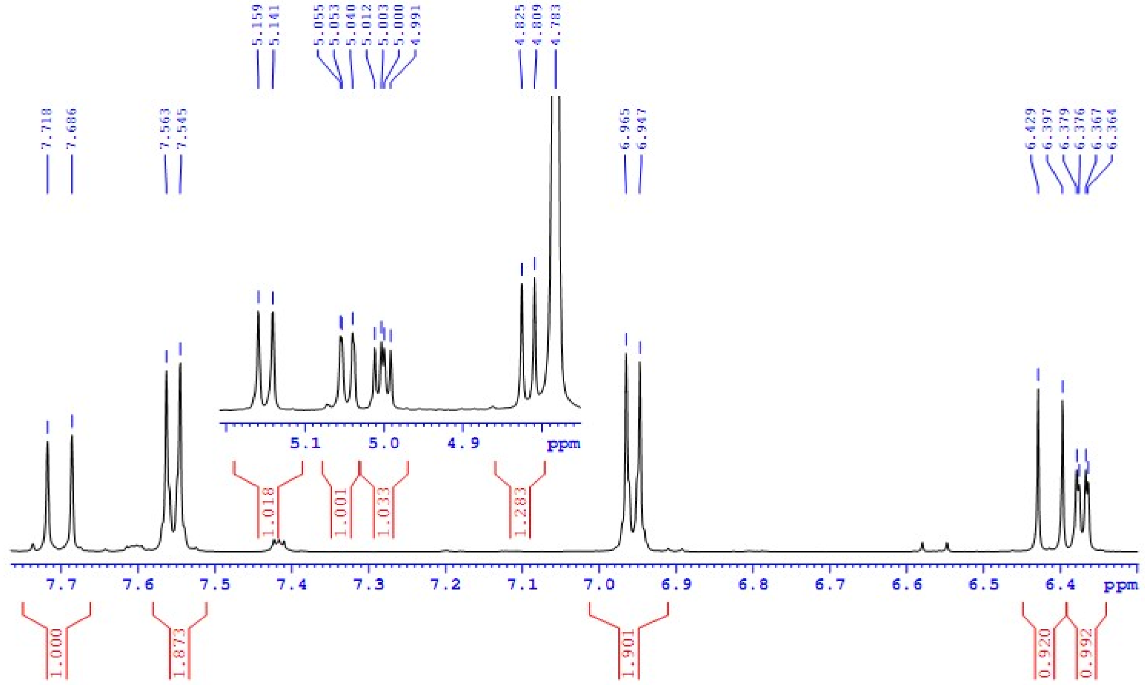

$^{1}$H-NMR spectrum (expansion) of compound **1**

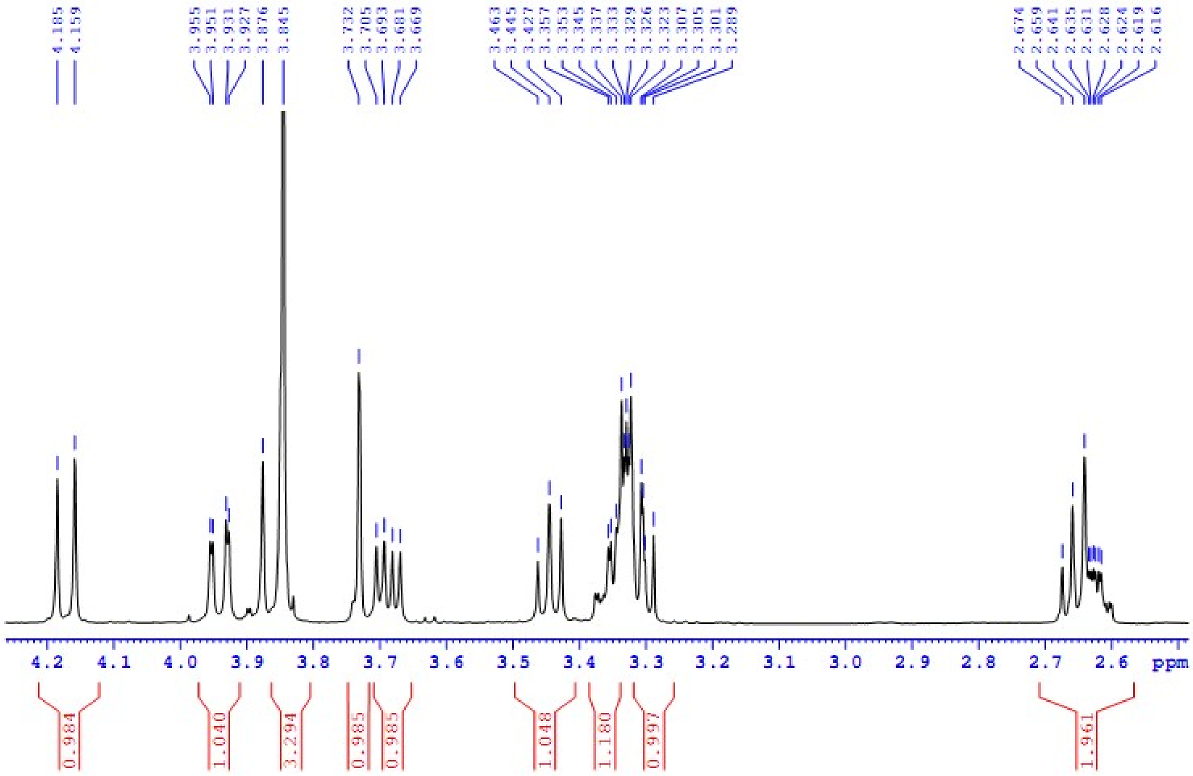

$^{1}$H-NMR spectrum (expansion) of compound **1**

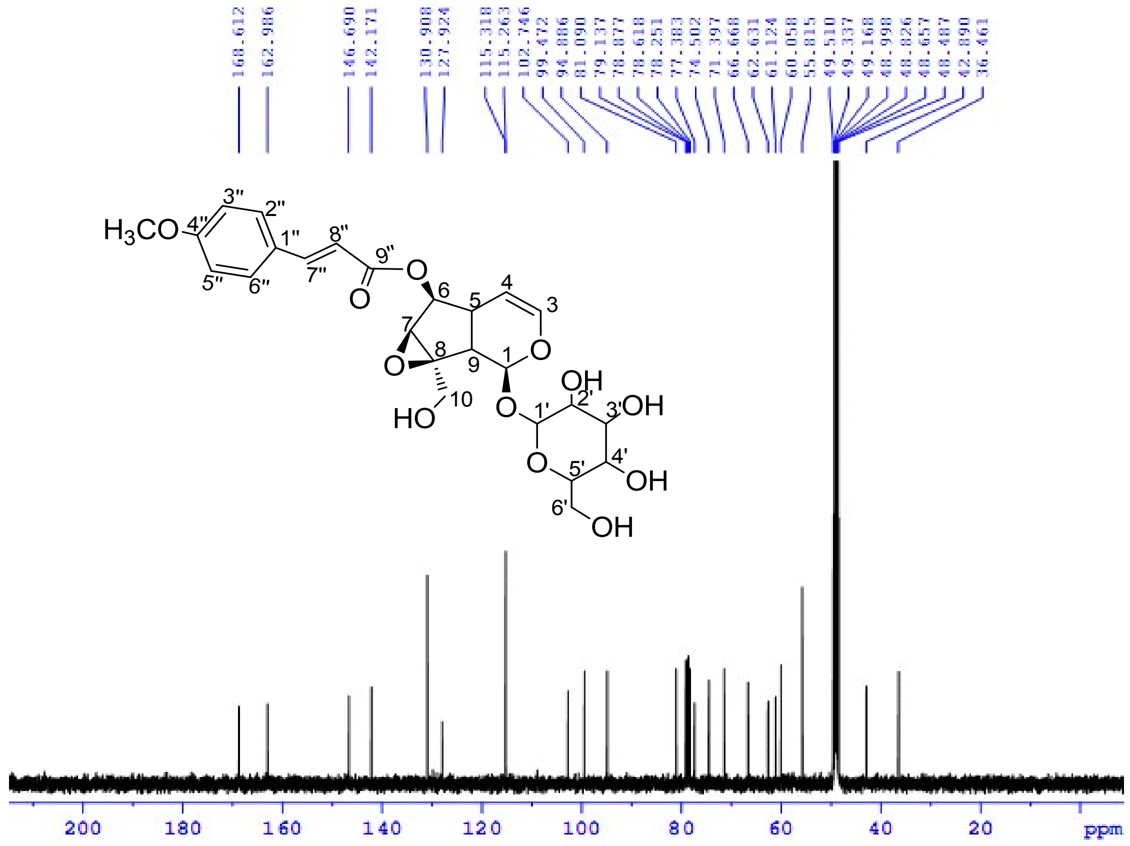


$^{13}$C-NMR spectrum of compound **1**

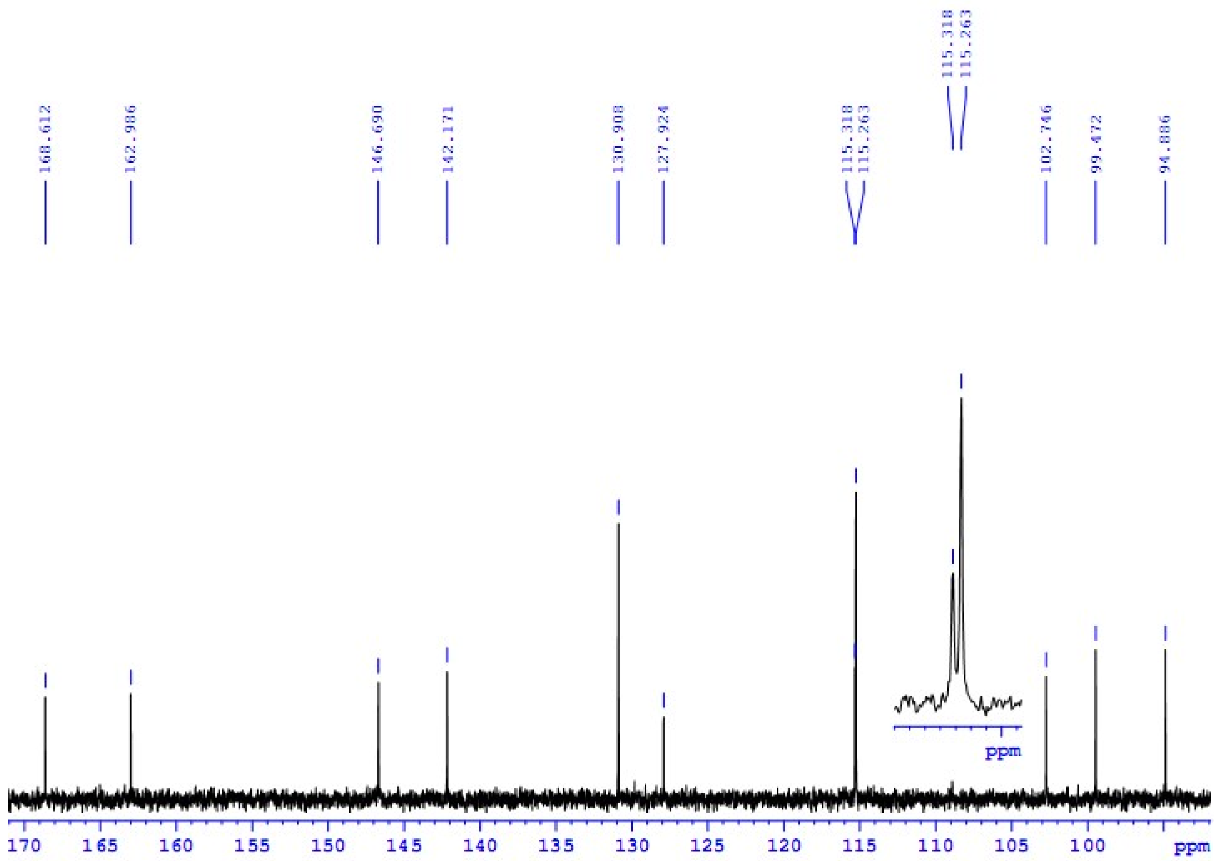


$^{13}$C-NMR spectrum (expansion) of compound **1**

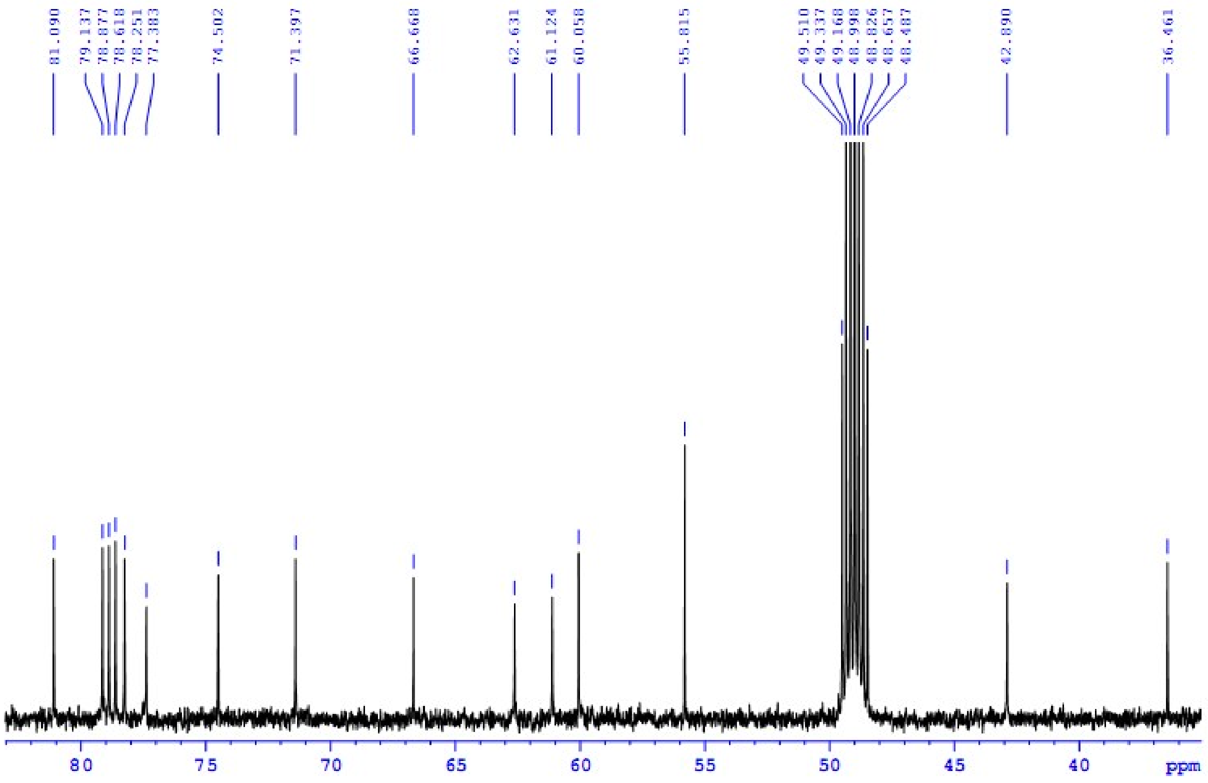


$^{13}$C-NMR spectrum (expansion) of compound **1**

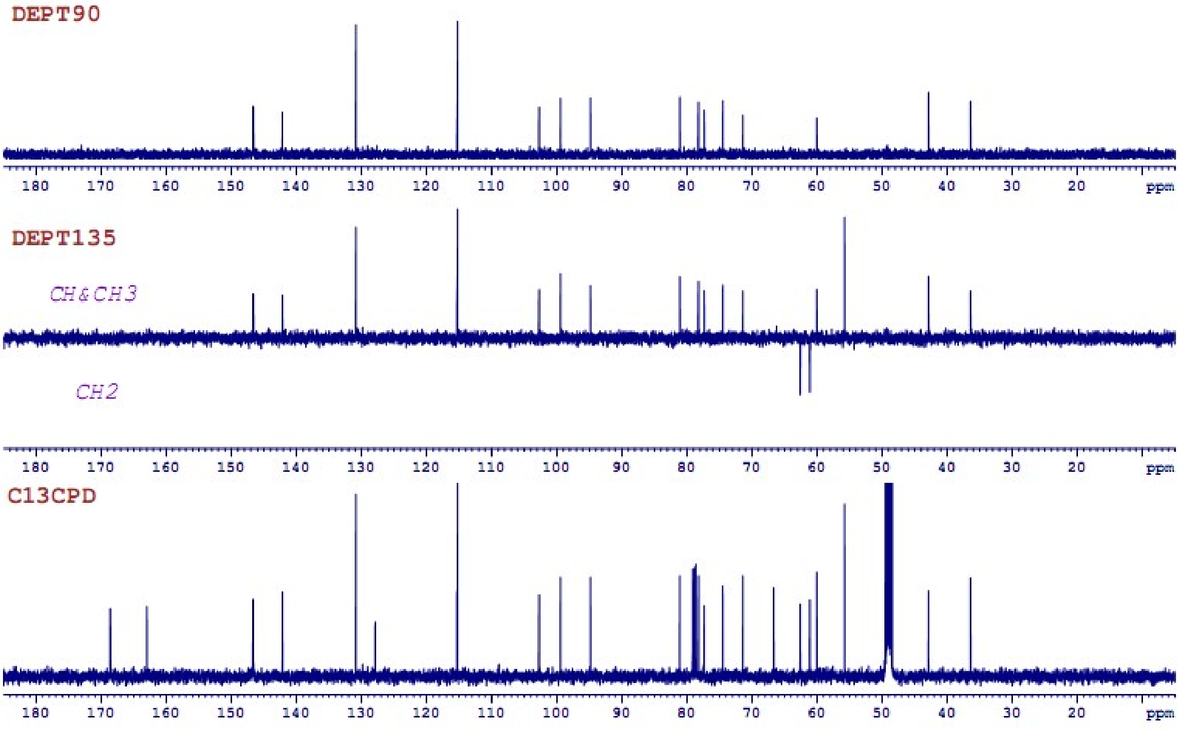


DEPT spectrum of compound **1**

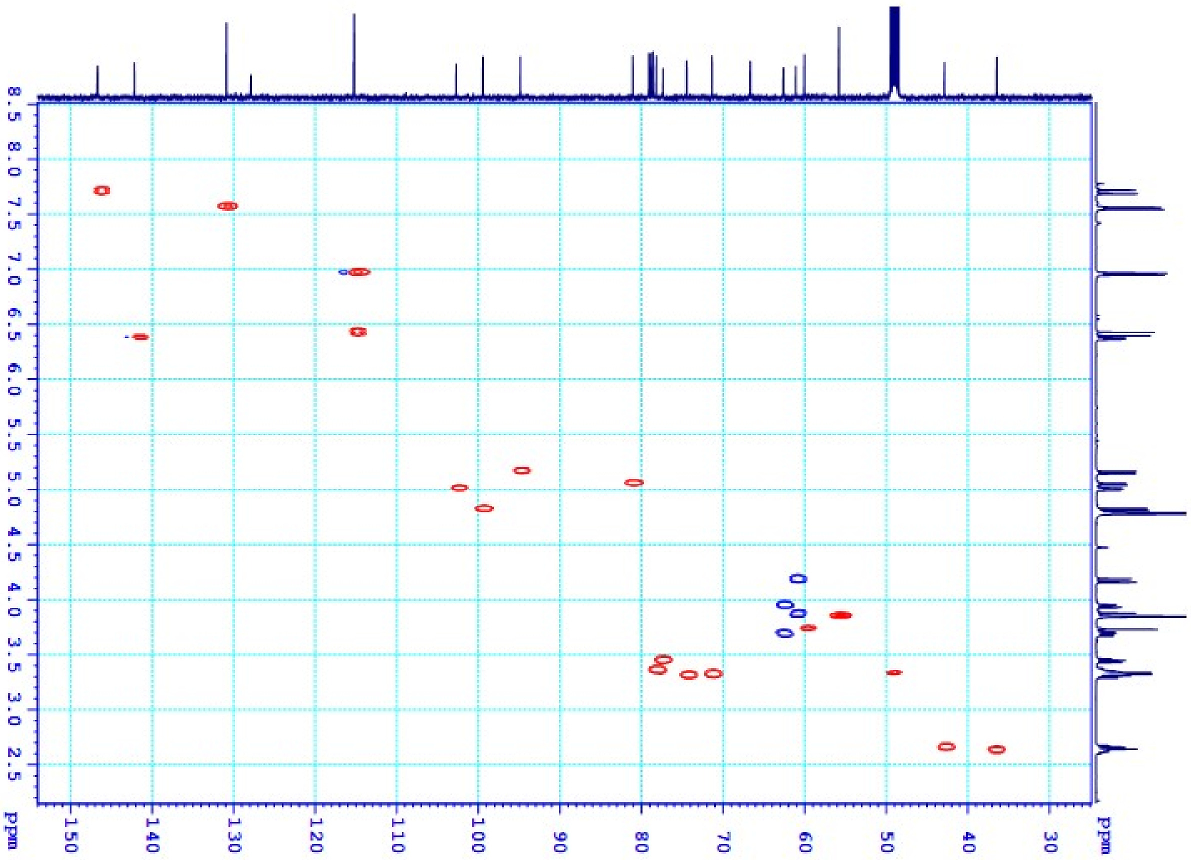

HSQC spectrum of compound **1**

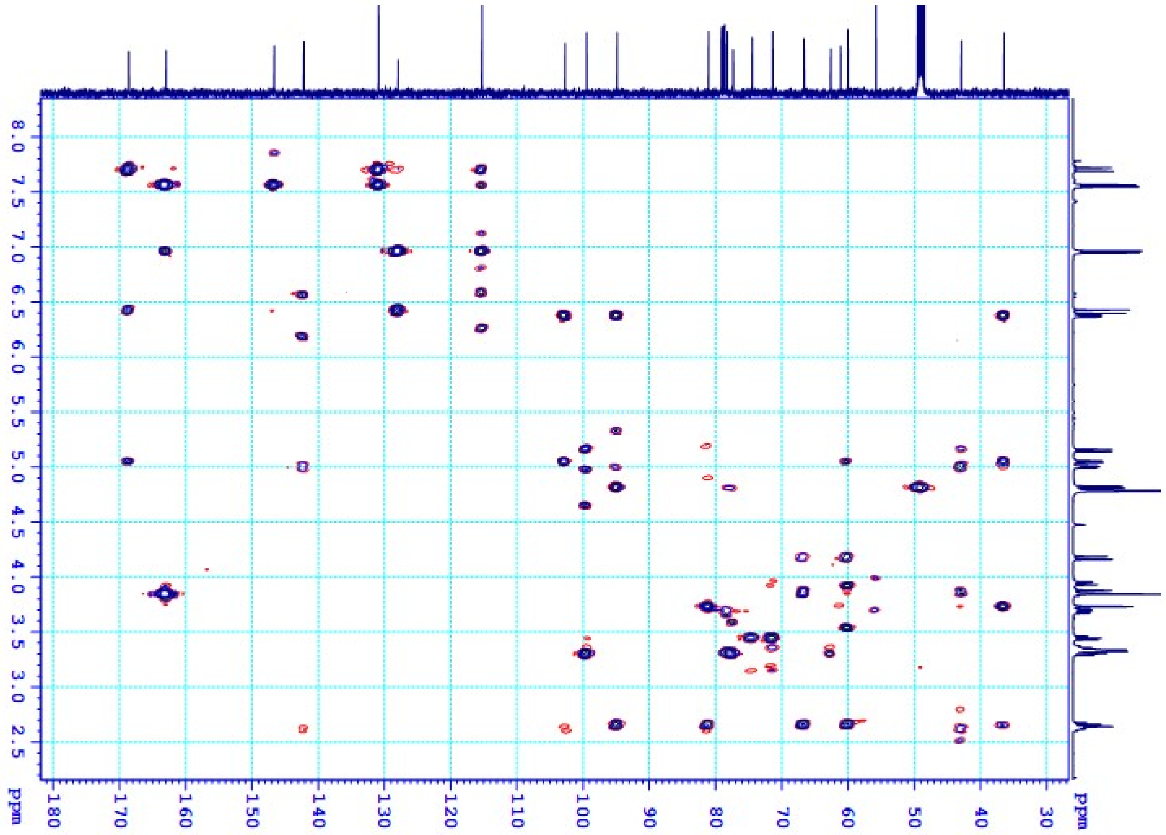

HMBC spectrum of compound **1**

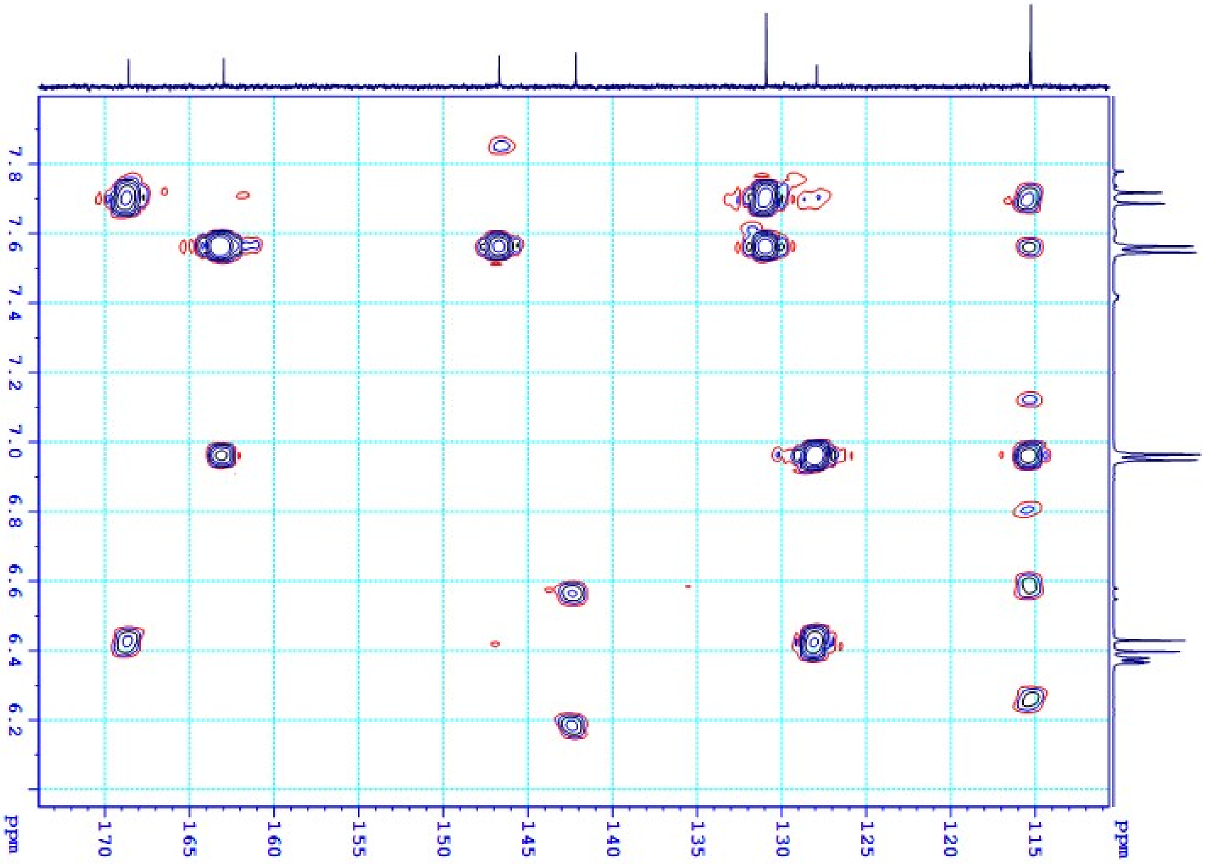


HMBC spectrum (expansion) of compound **1**

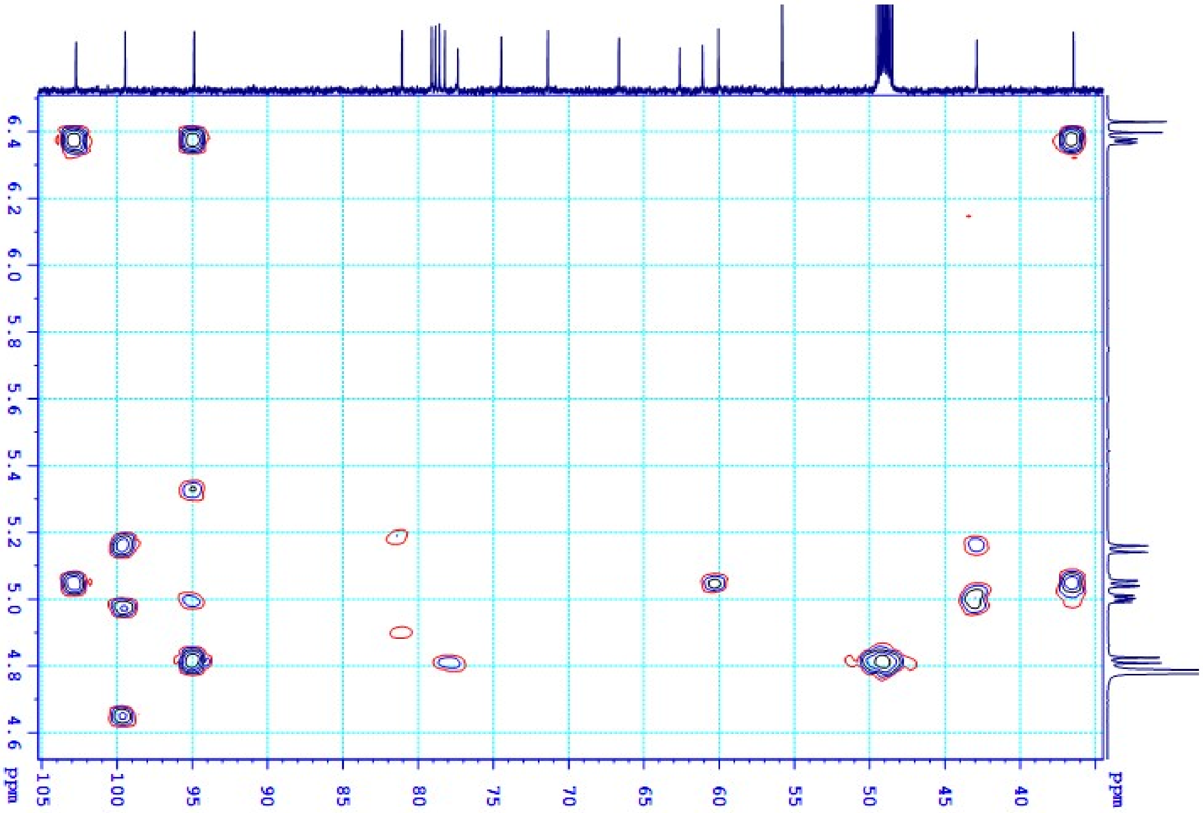


HMBC spectrum (expansion) of compound **1**

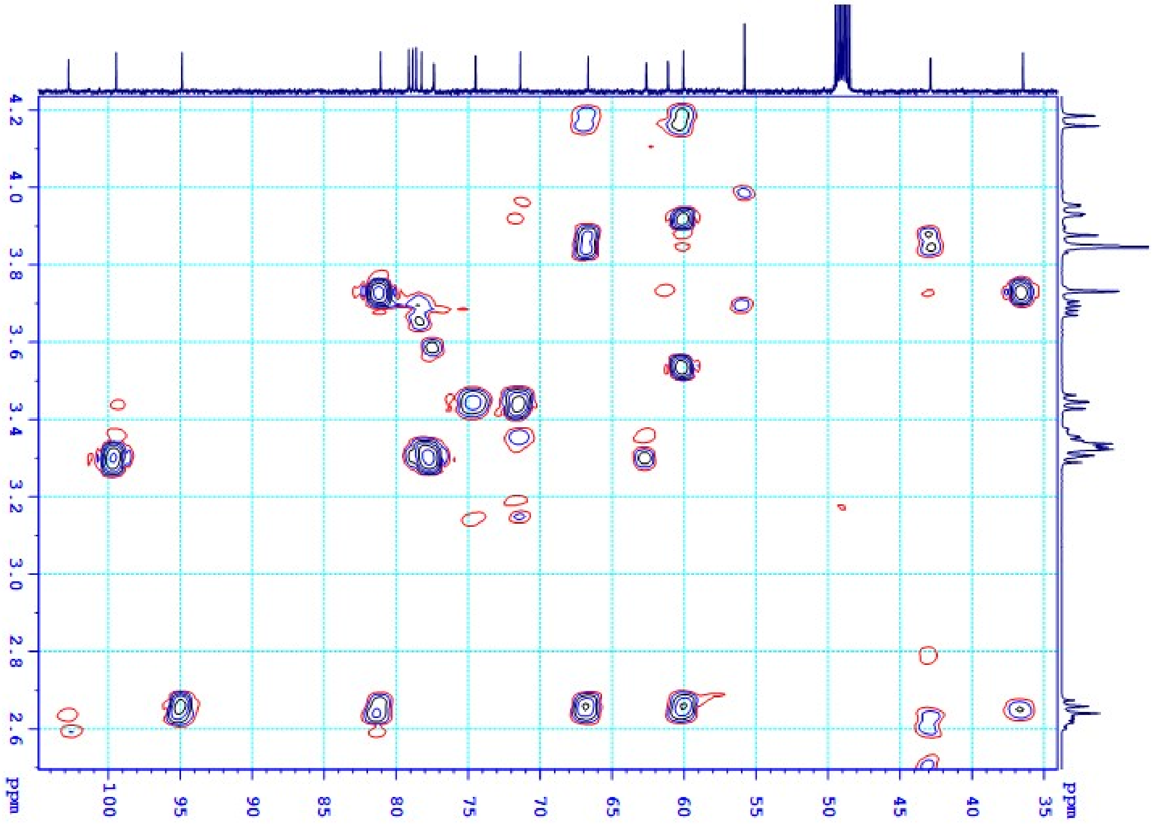

HMBC spectrum (expansion) of compound **1**

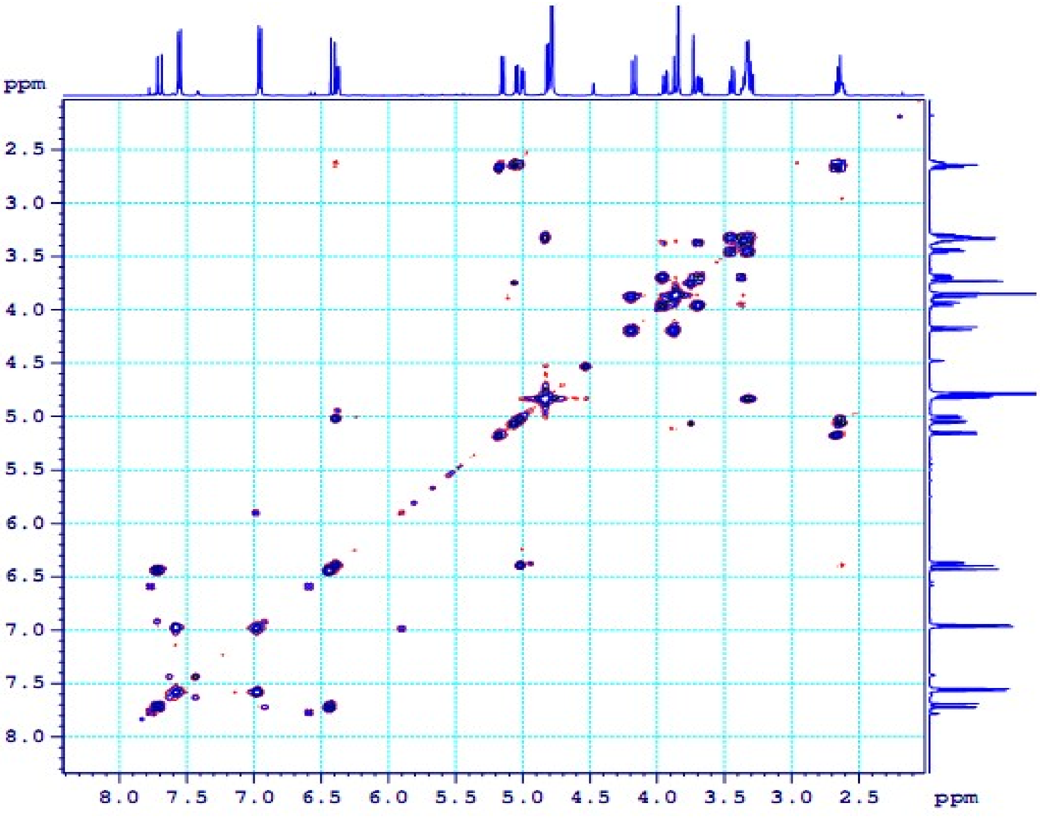

COSY spectrum of compound **1**

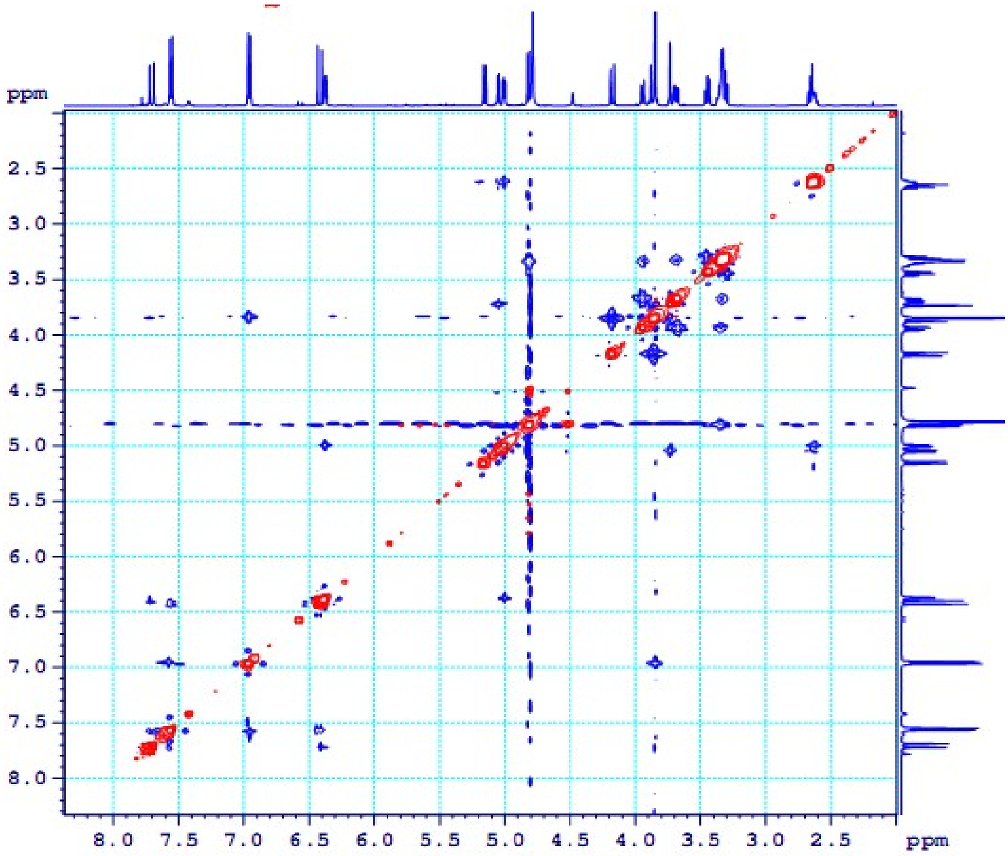


NOESY spectrum of compound **1**

## 1.2. Compound 2

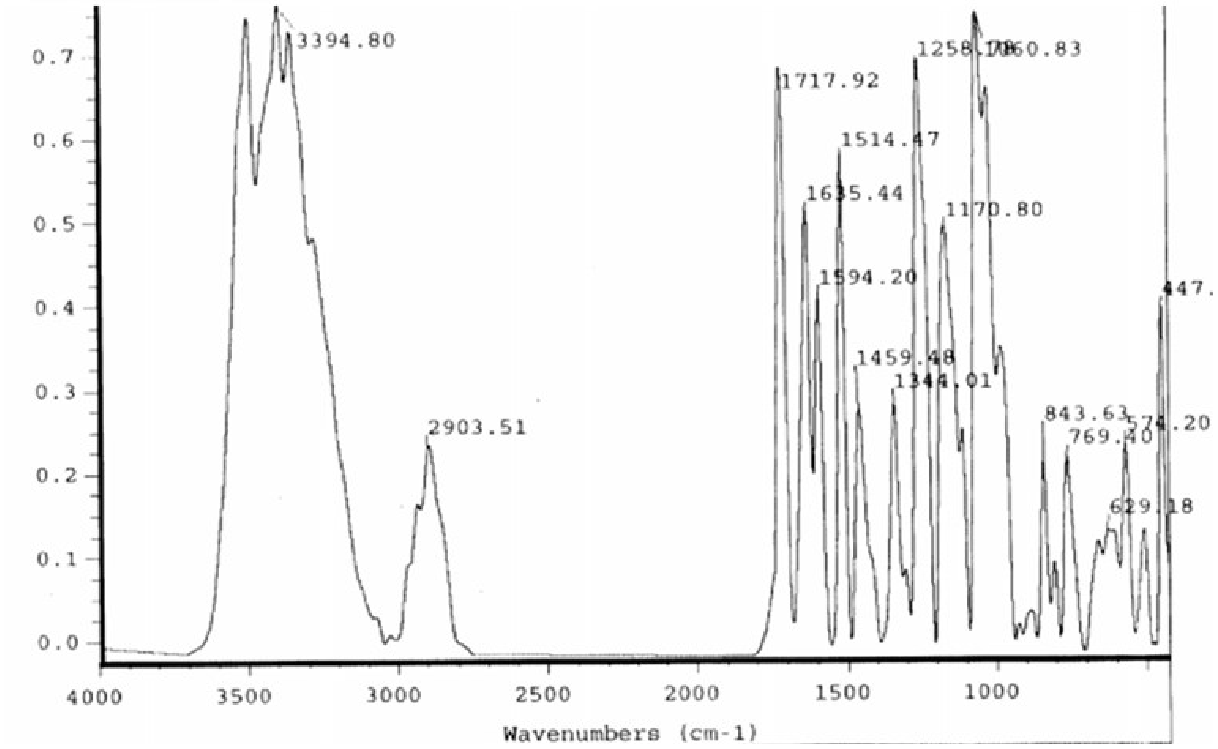


IR spectrum of compound **2**

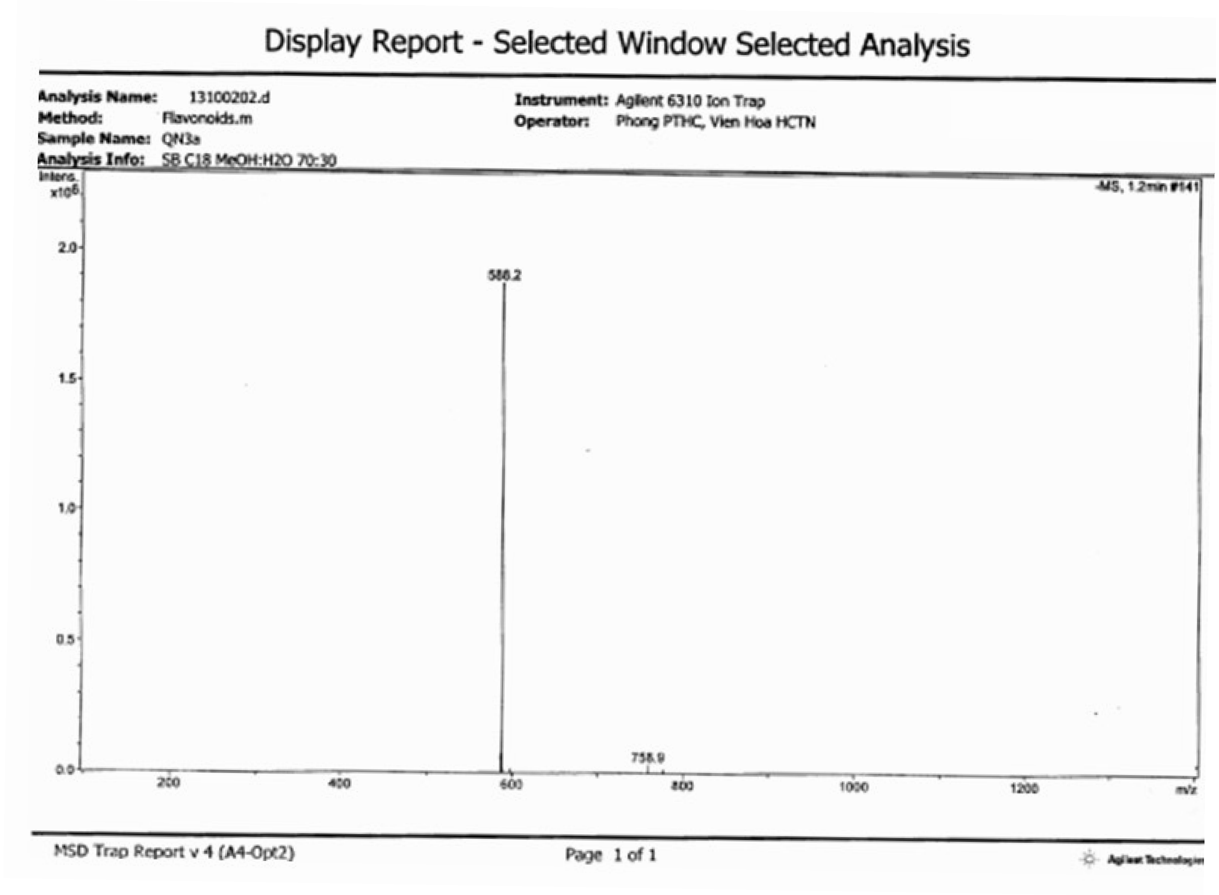


(-)-ESI-MS spectrum of compound **2**

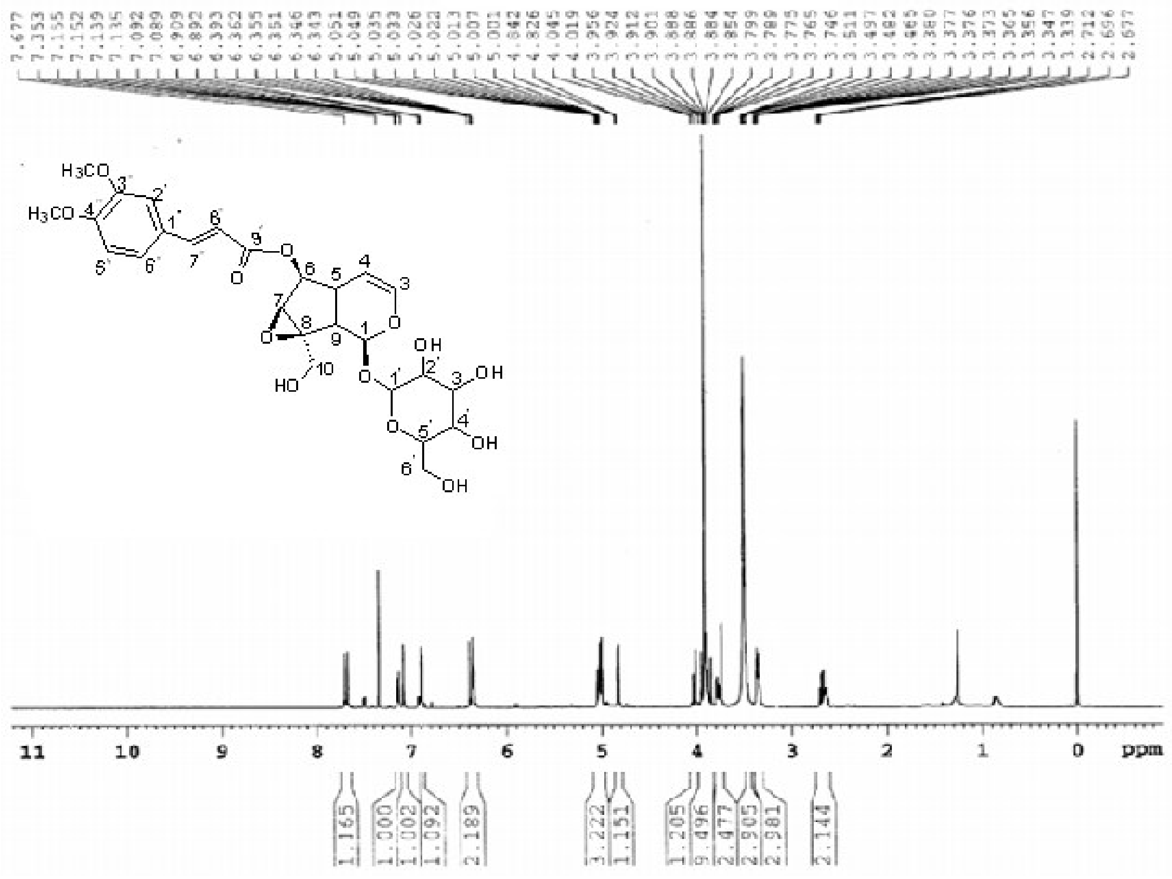


¹H-NMR spectrum ($CDCl_3$ + $CD_3OD$) of compound **2**

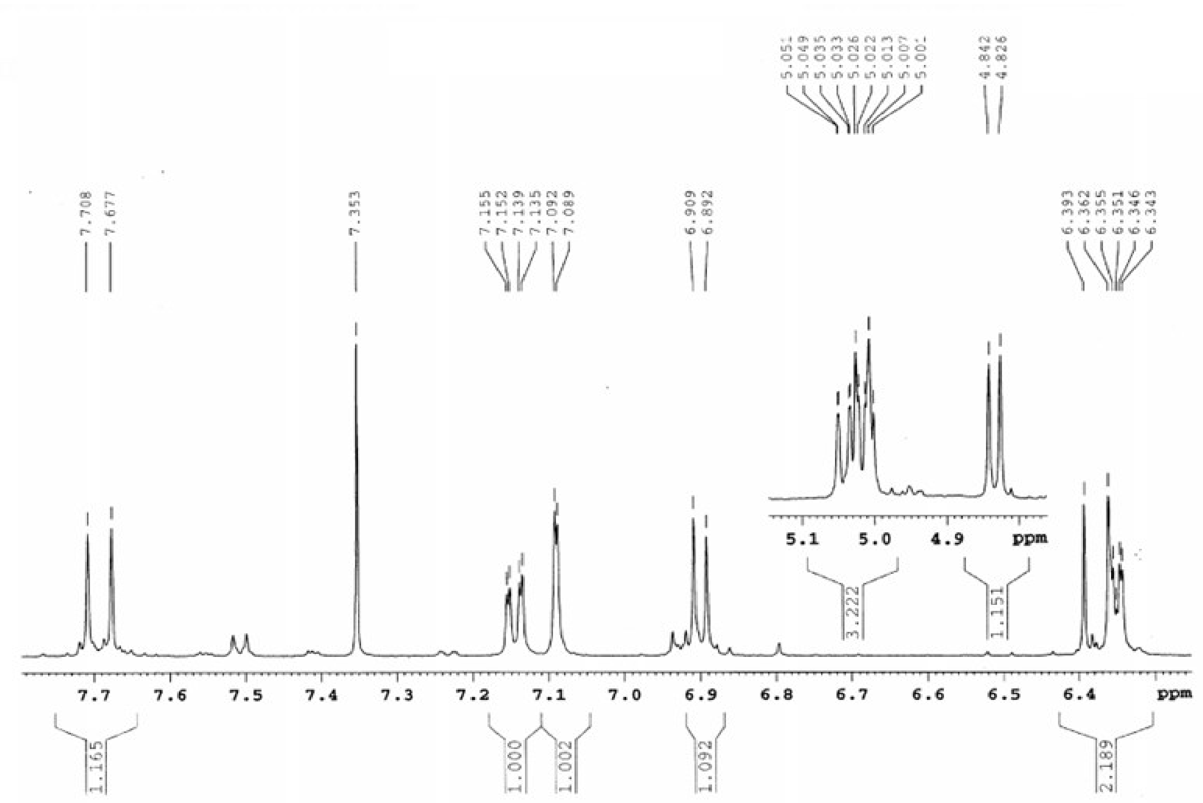


¹H-NMR spectrum (expansion) of compound **2**

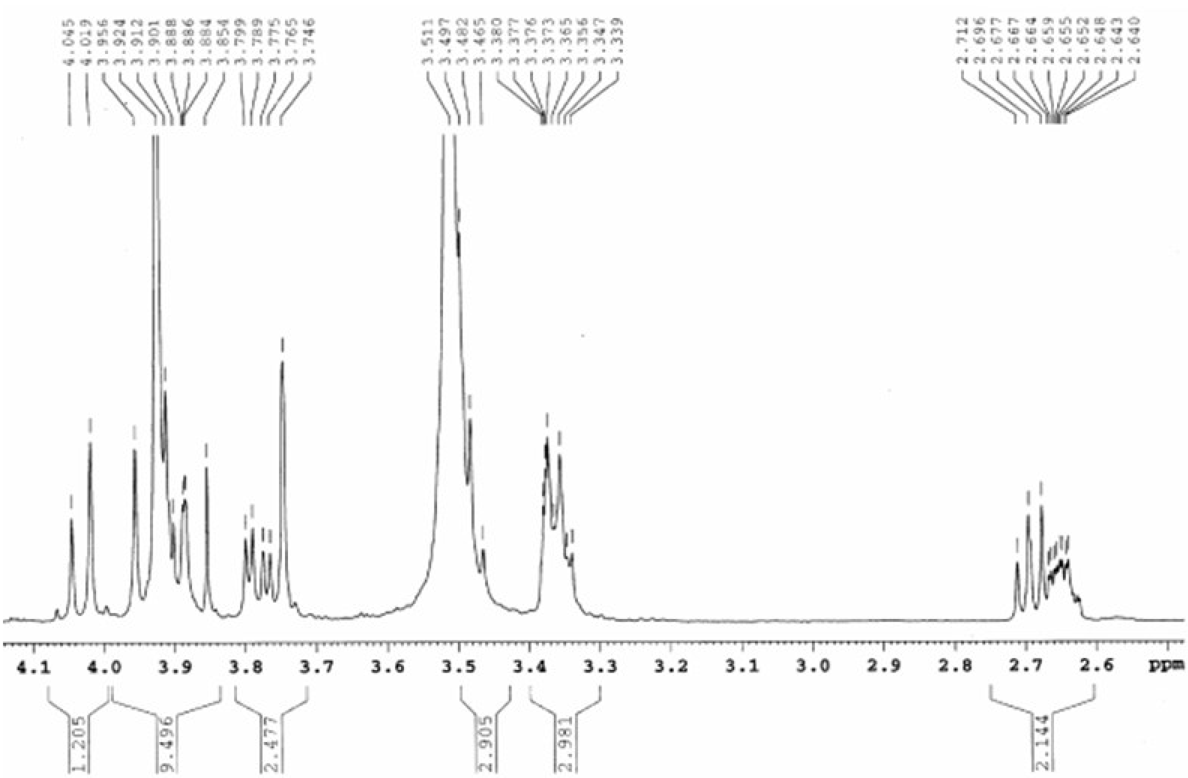


$^{1}$H-NMR spectrum (expansion) of compound **2**

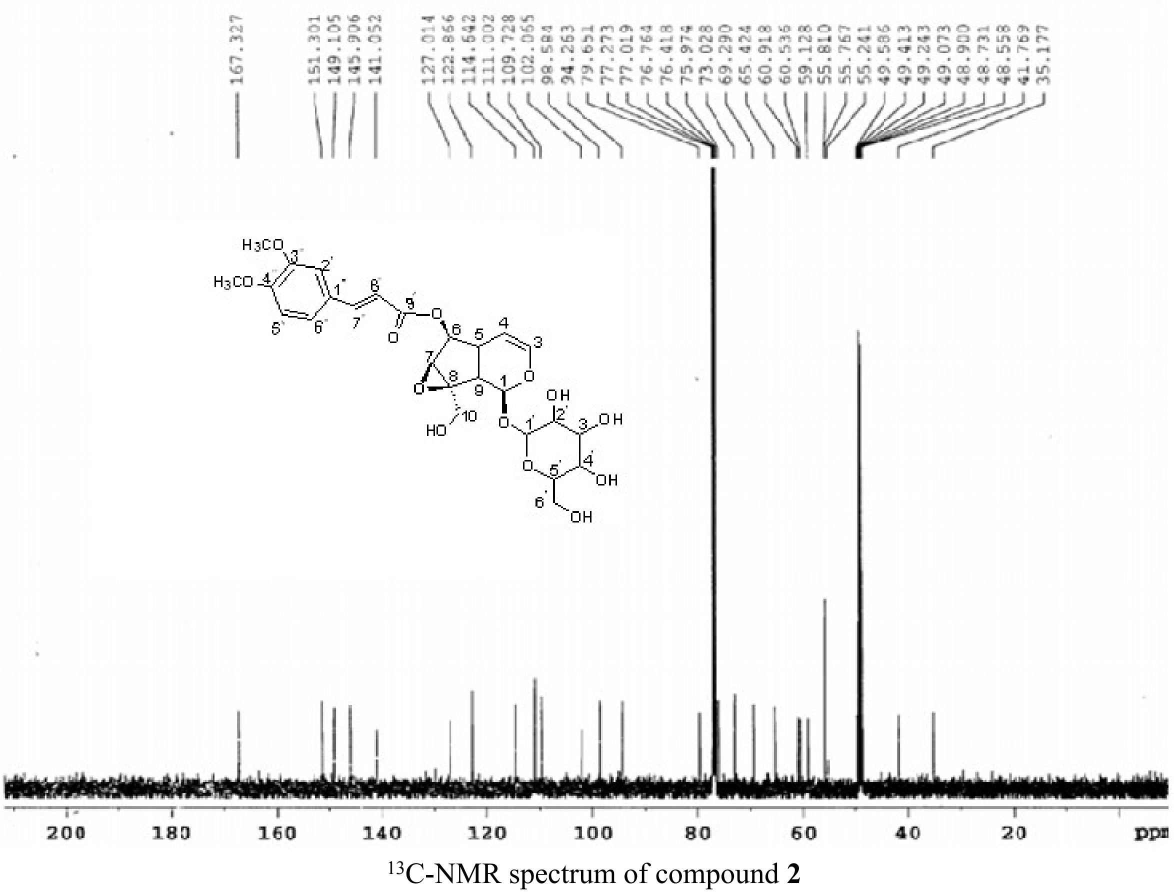


$^{13}$C-NMR spectrum of compound **2**

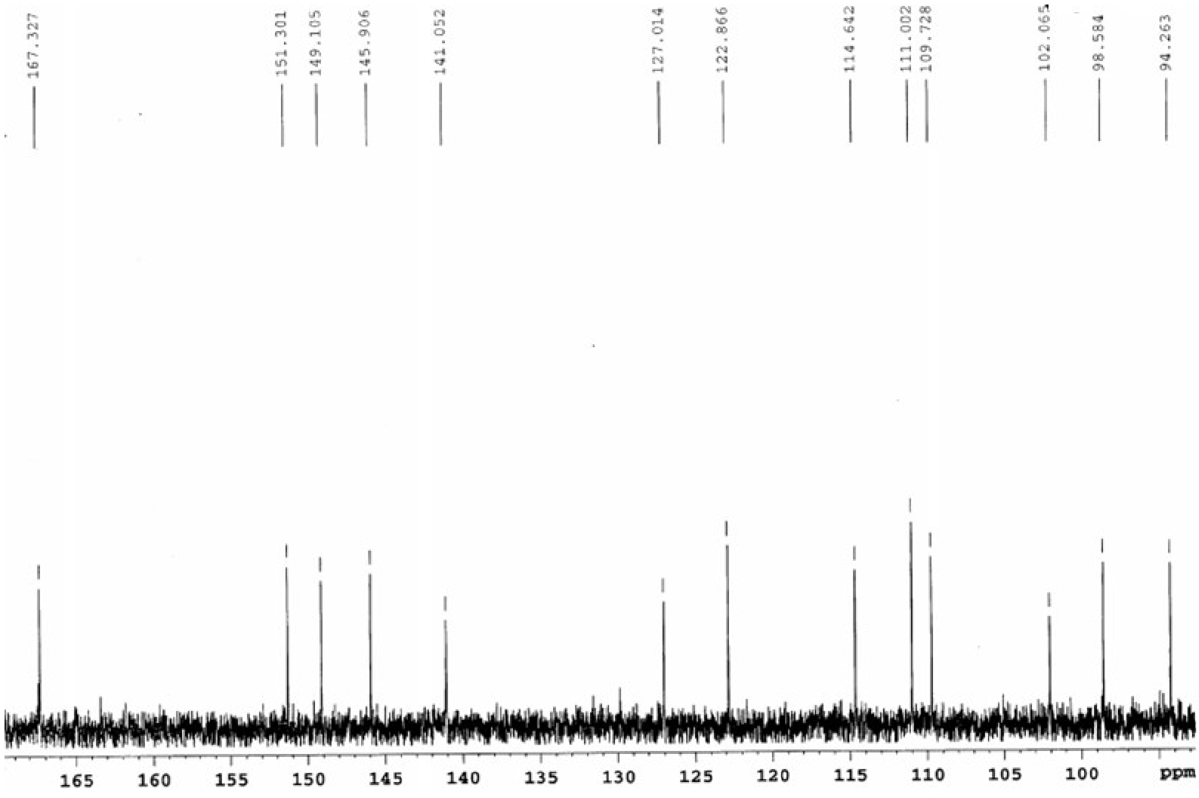


$^{13}$C-NMR spectrum (expansion) of compound **2**

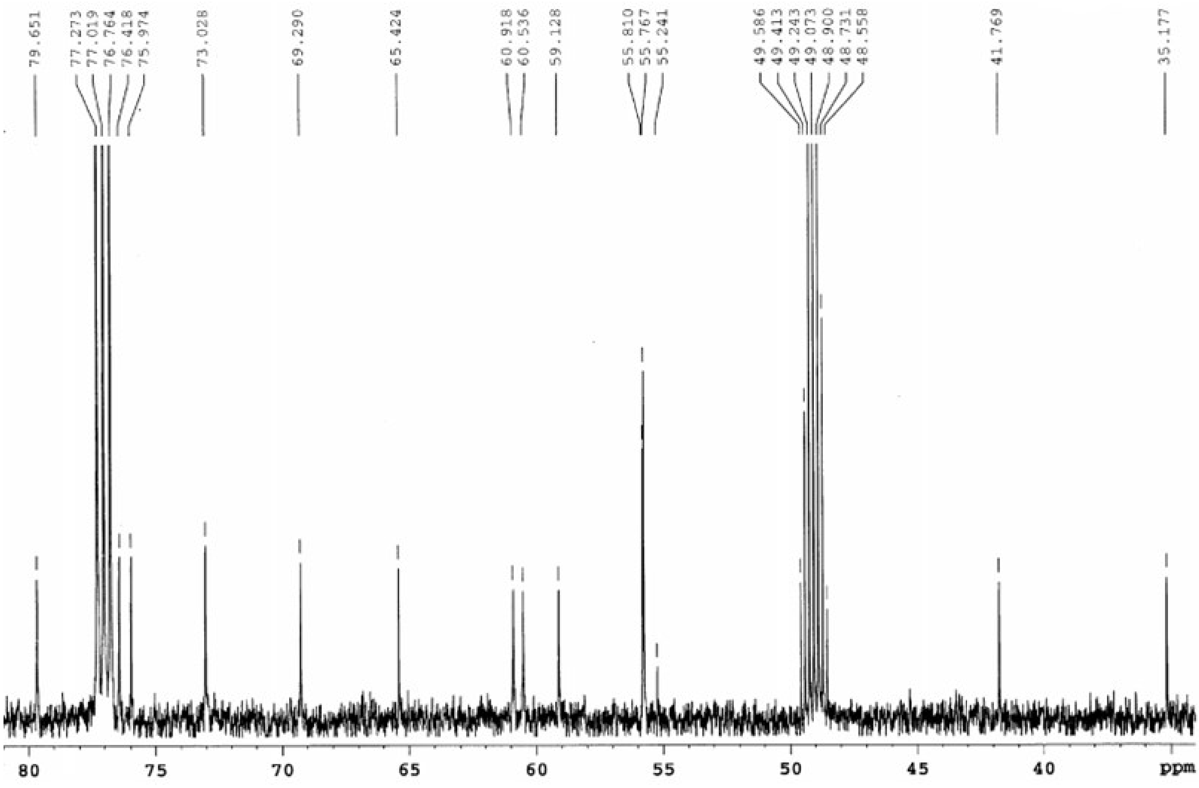


$^{13}$C-NMR spectrum (expansion) of compound **2**

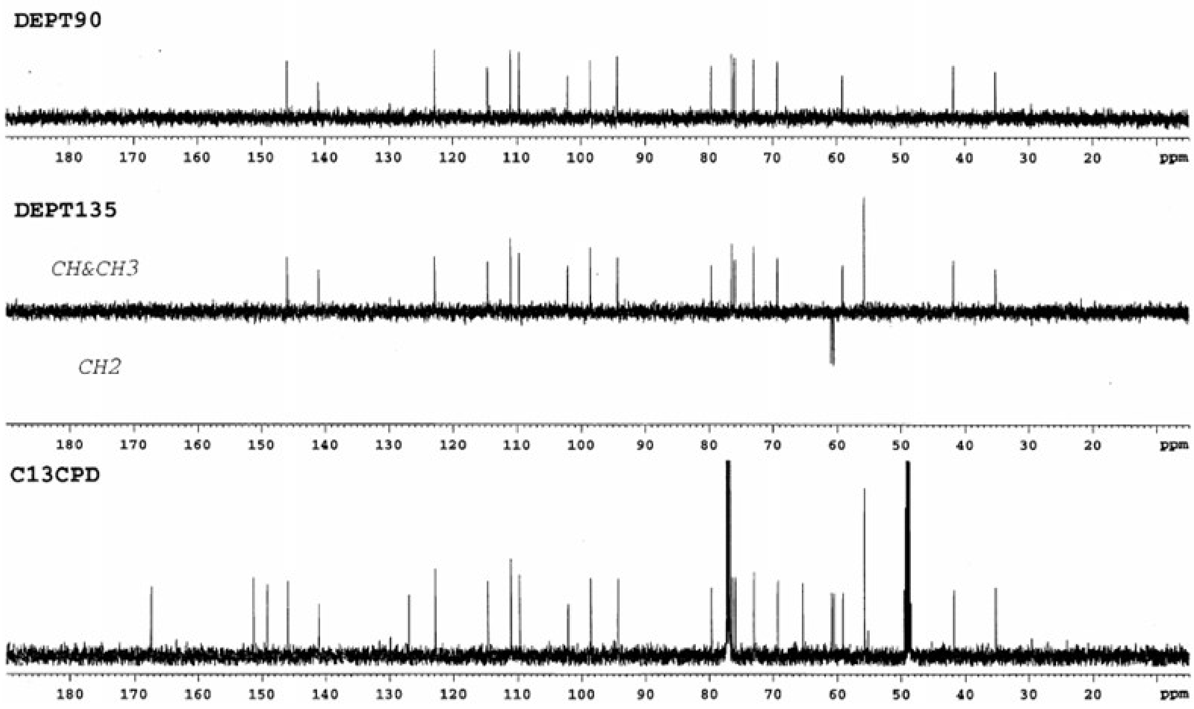

DEPT spectrum of compound **2**

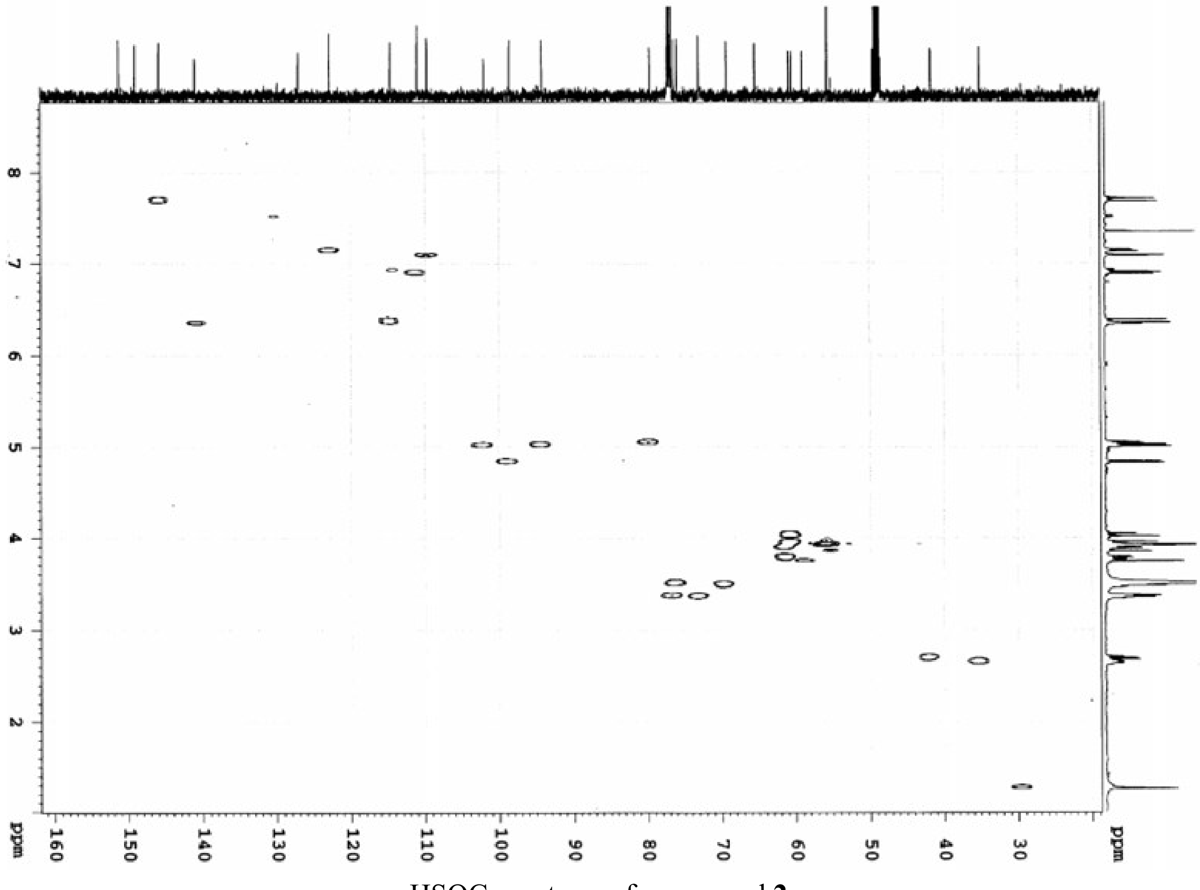
HSQC spectrum of compound **2**

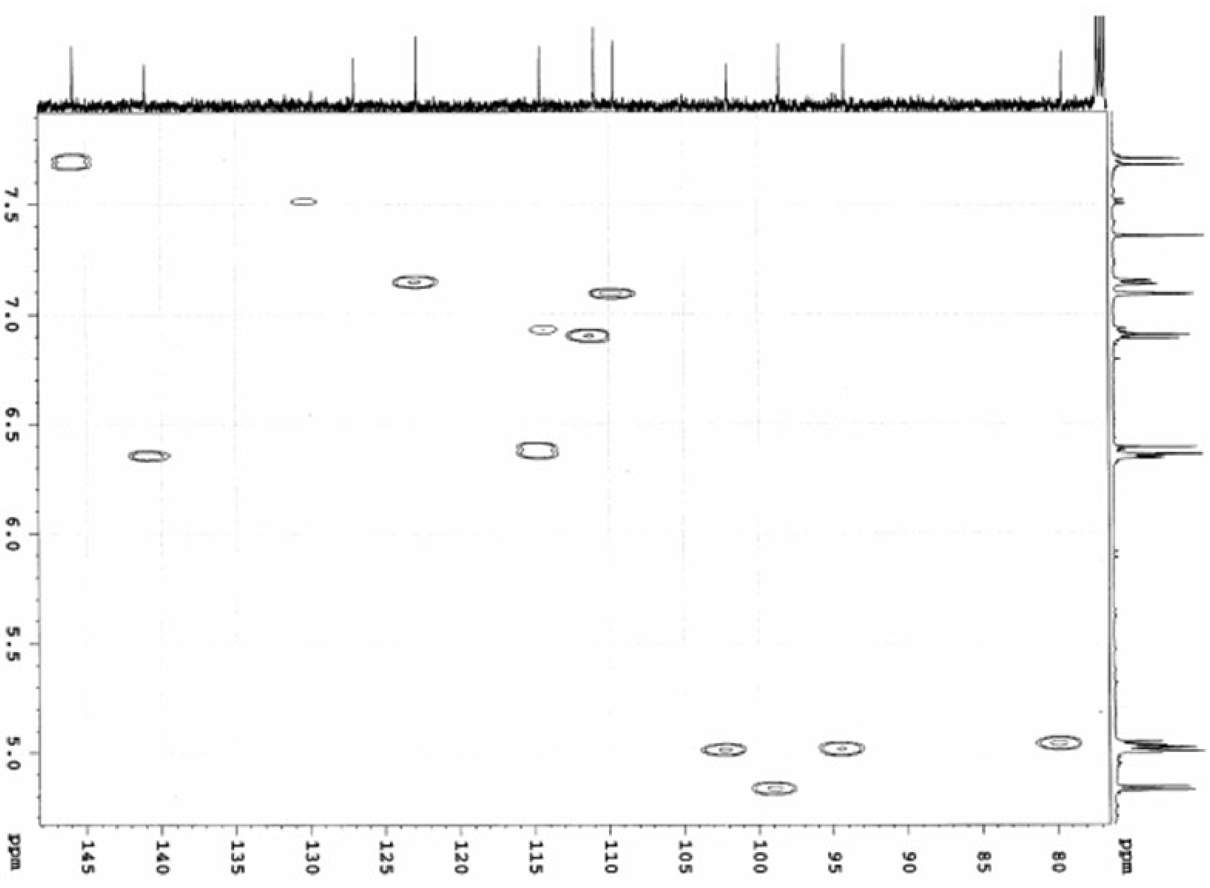

HSQC spectrum (expansion) of compound **2**

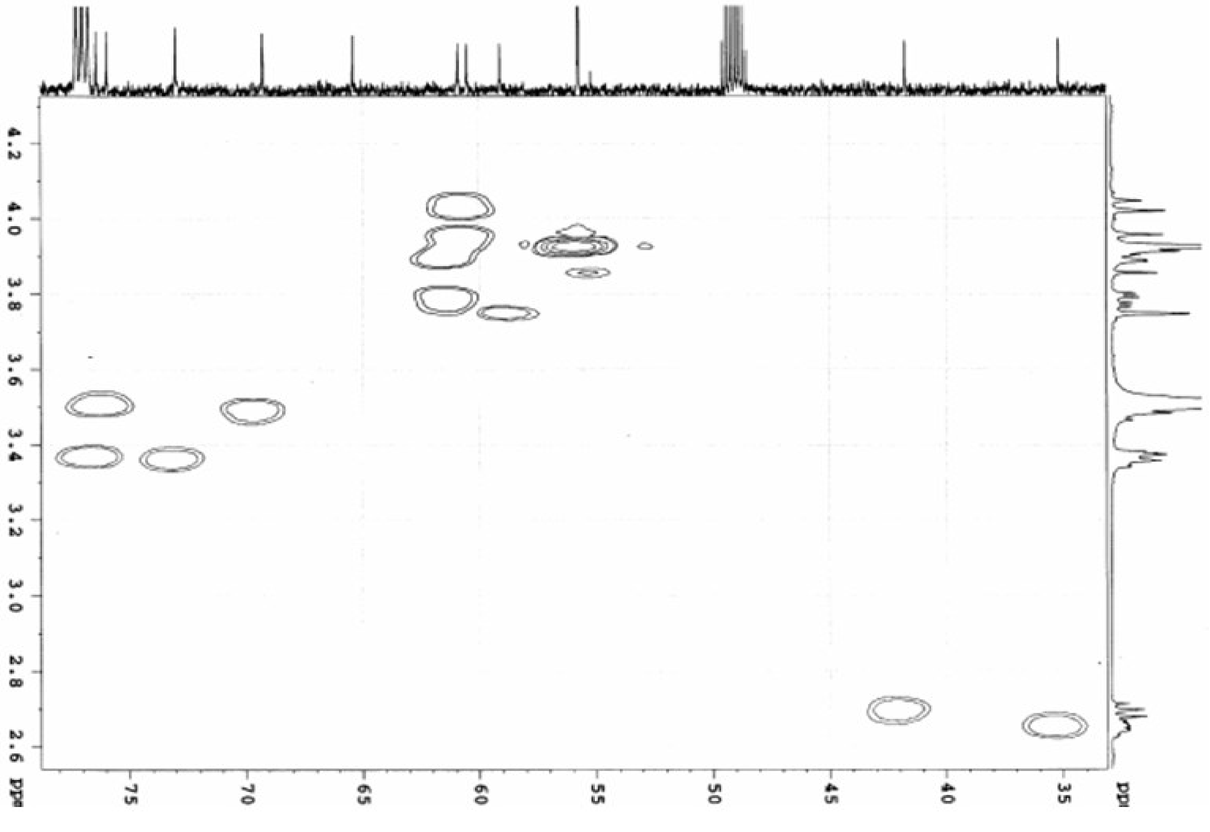

HSQC spectrum (expansion) of compound **2**

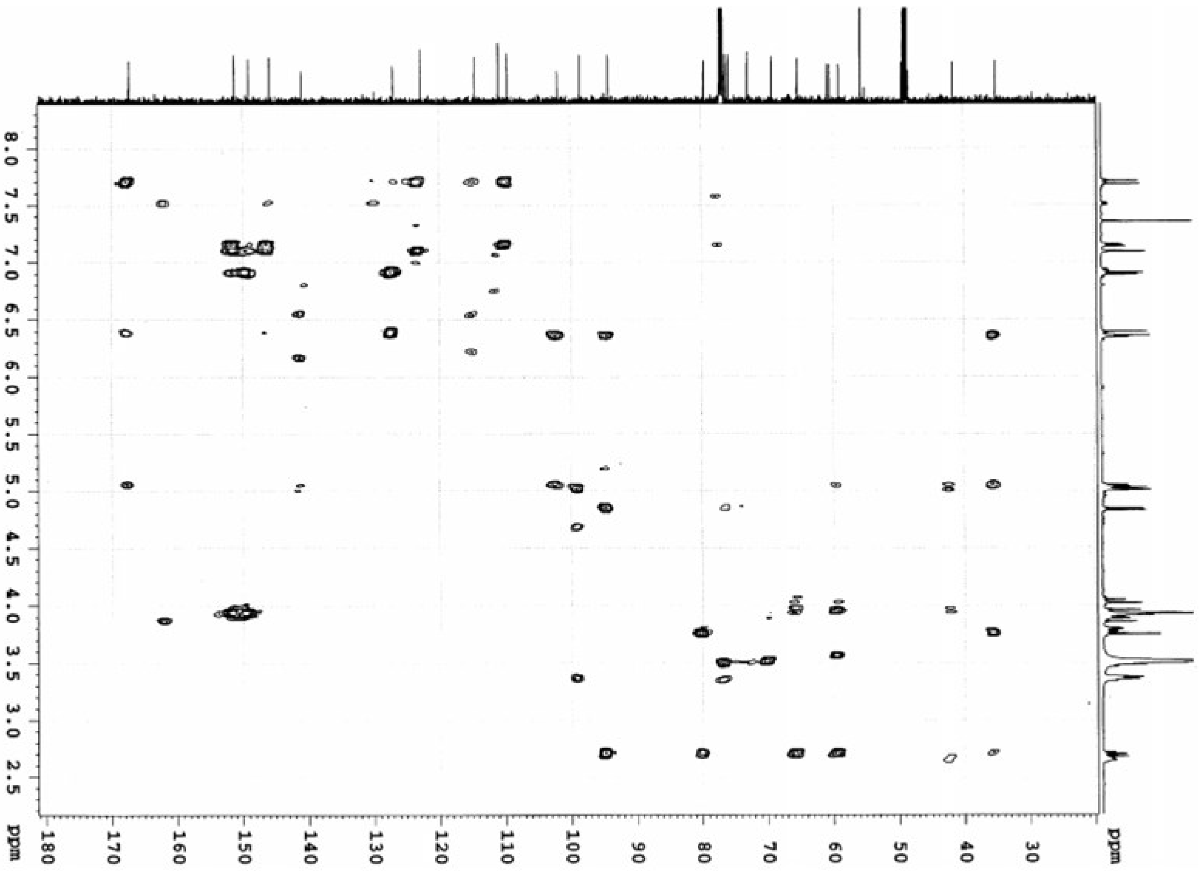


HMBC spectrum of compound **2**

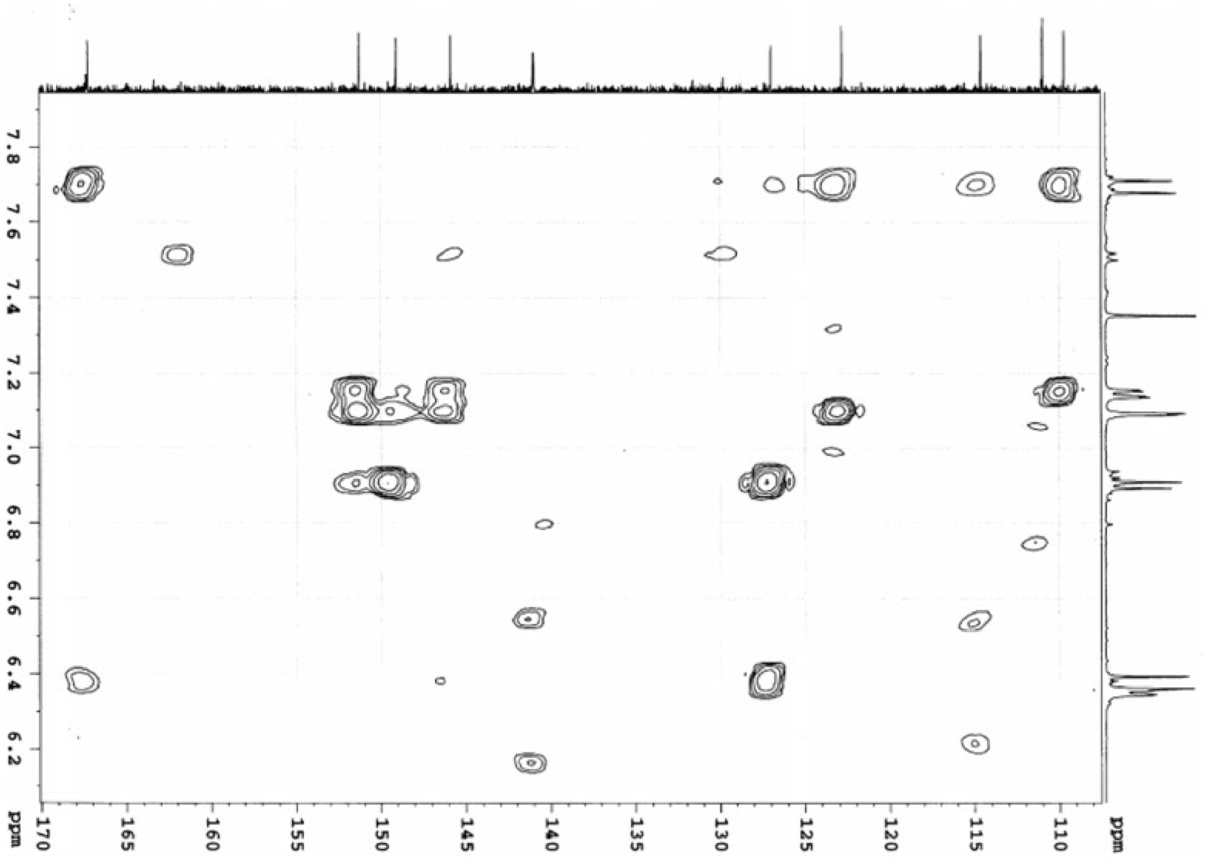


HMBC spectrum (expansion) of compound **2**

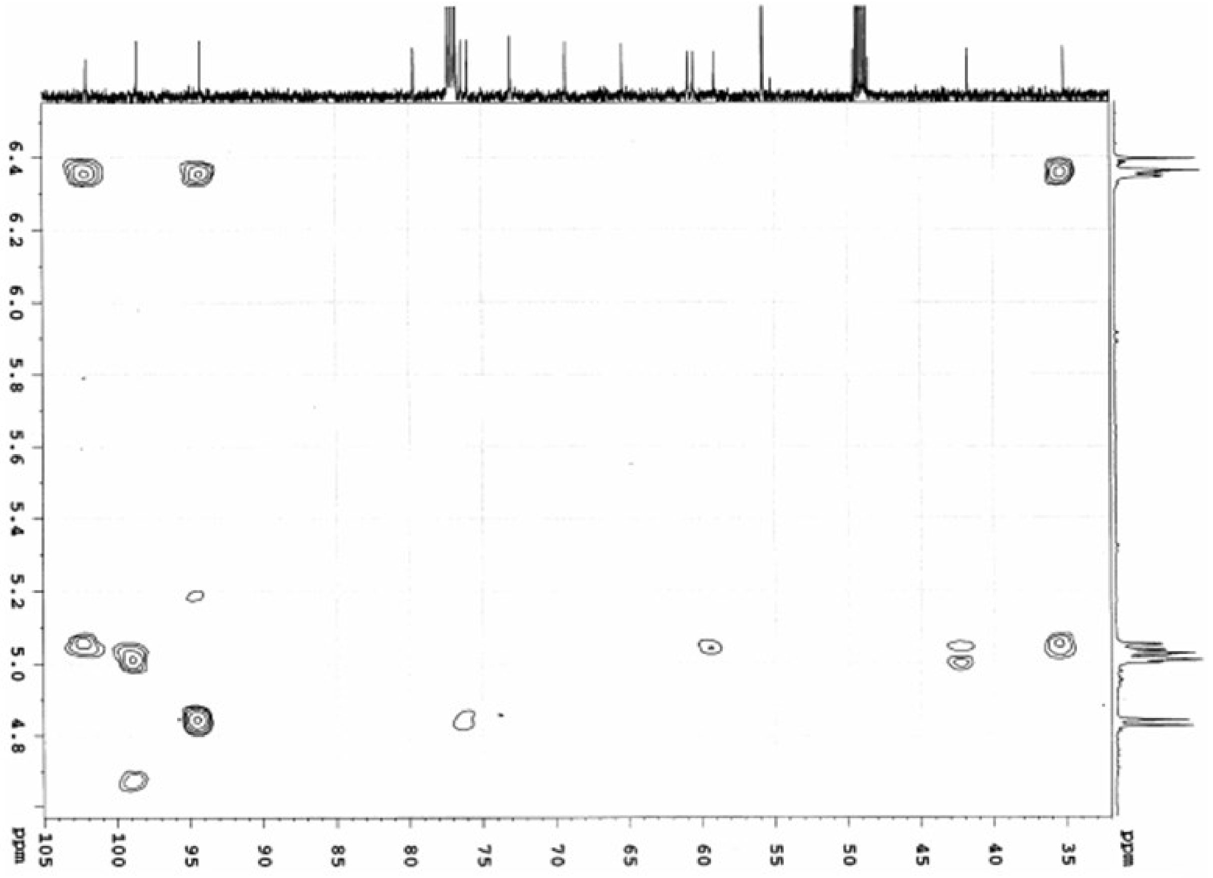

HMBC spectrum (expansion) of compound **2**

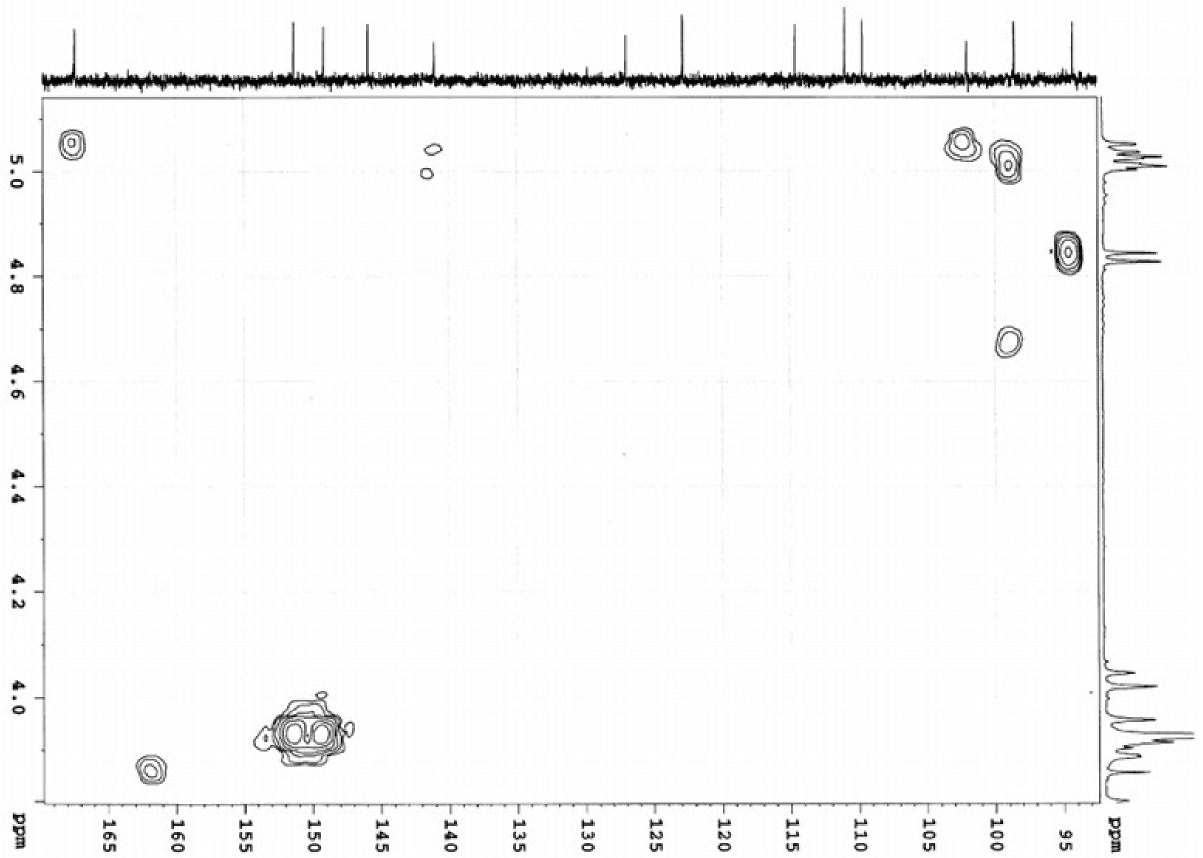

HMBC spectrum (expansion) of compound **2**

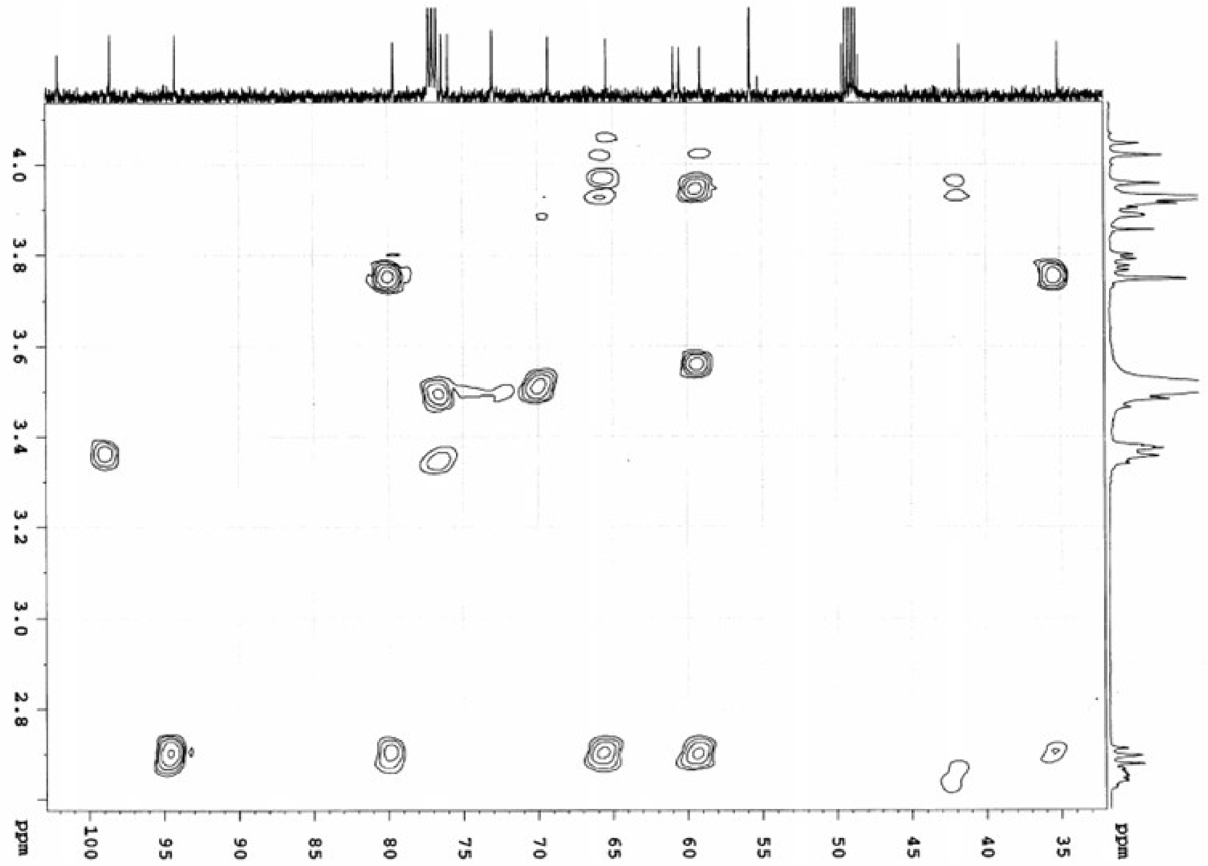

HMBC spectrum (expansion) of compound **2**

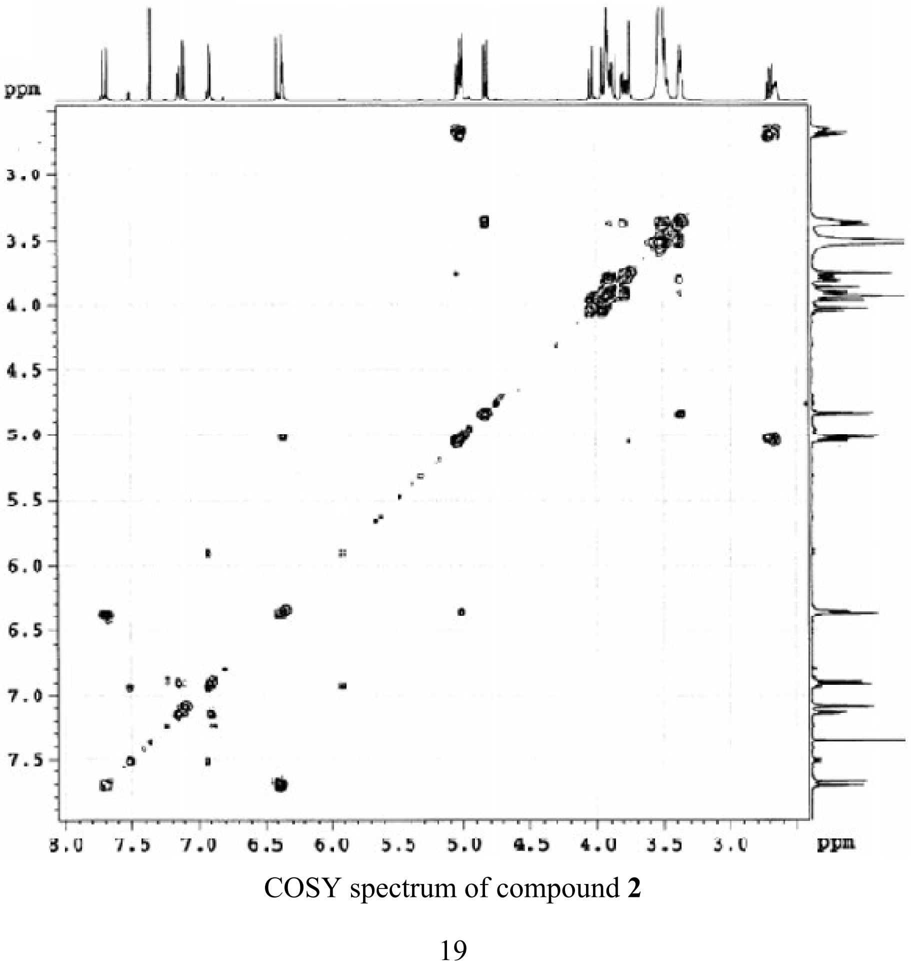

COSY spectrum of compound **2**

### 1.3. Compound 3

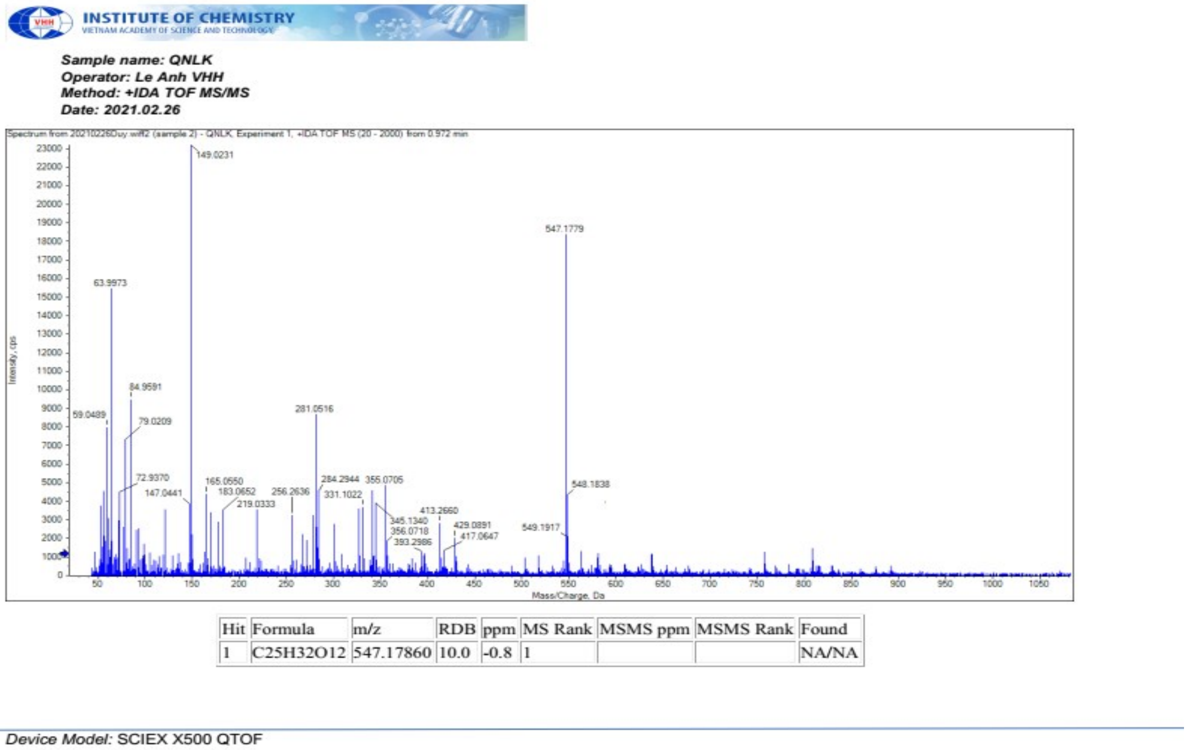


| Hit | Formula | m/z | RDB | ppm | MS Rank | MSMS ppm | MSMS Rank | Found |
|---|---|---|---|---|---|---|---|---|
| 1 | C25H32O12 | 547.17860 | 10.0 | -0.8 | 1 | | | NA/NA |

HR-ESI-MS spectrum of compound **3**

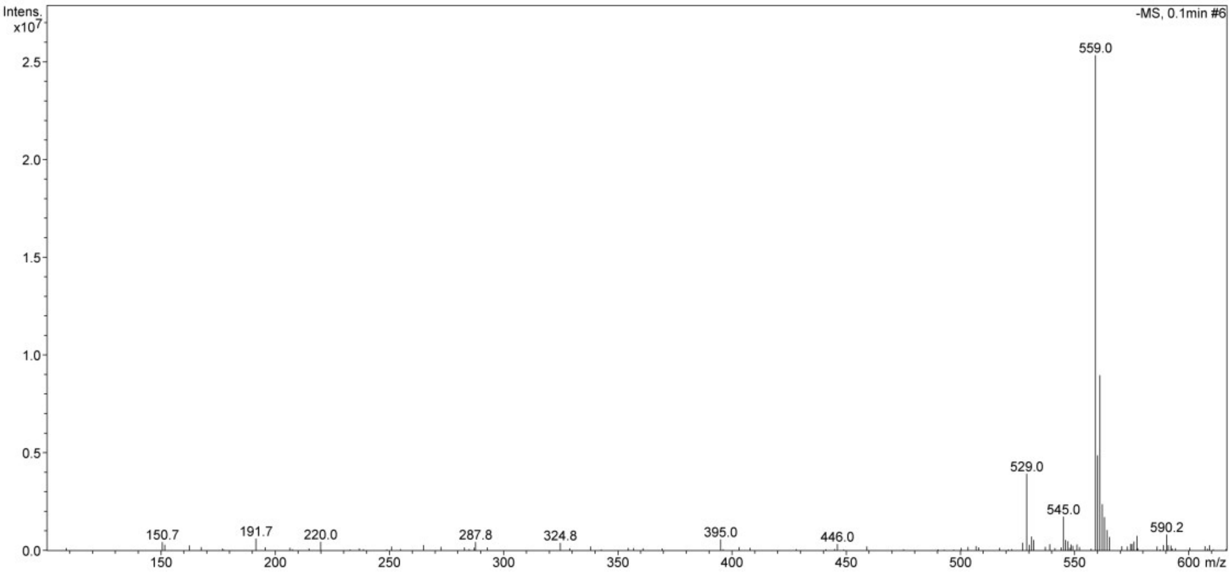


(+)-ESI-MS spectrum of compound **3**

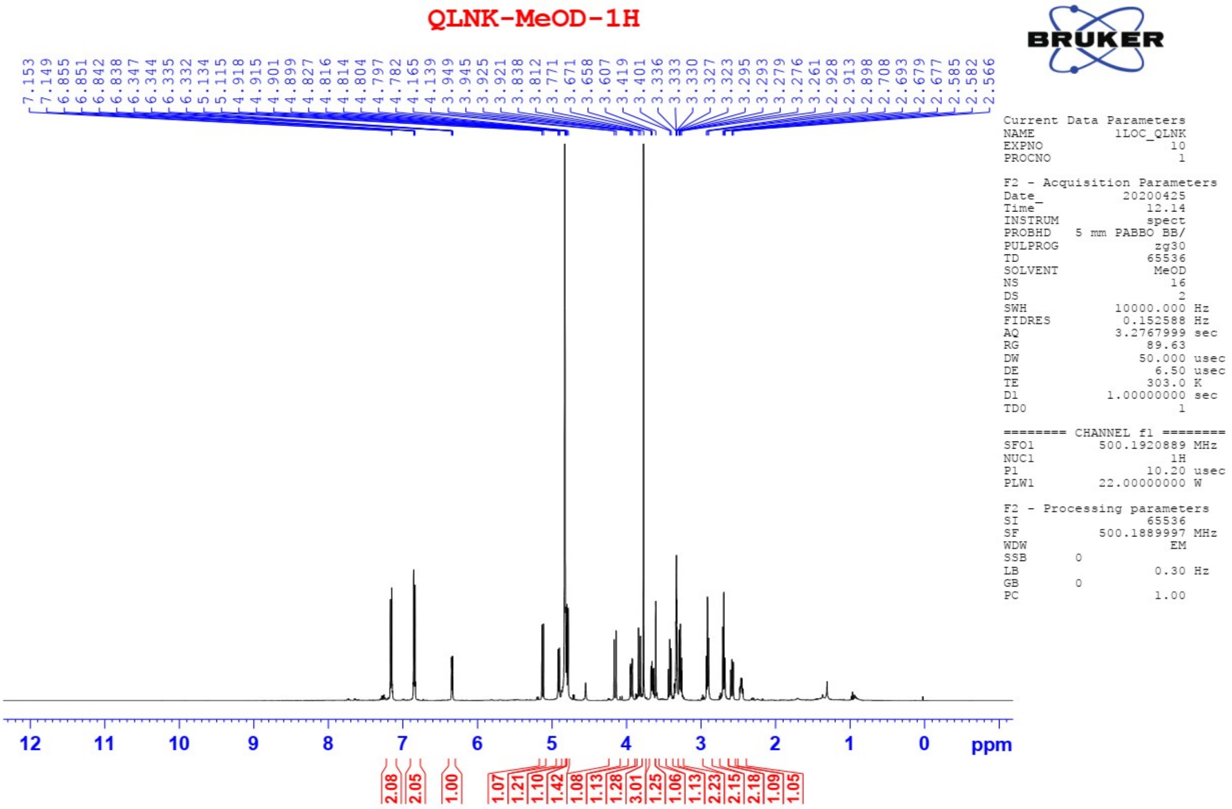

$^{1}$H-NMR spectrum of compound **3**

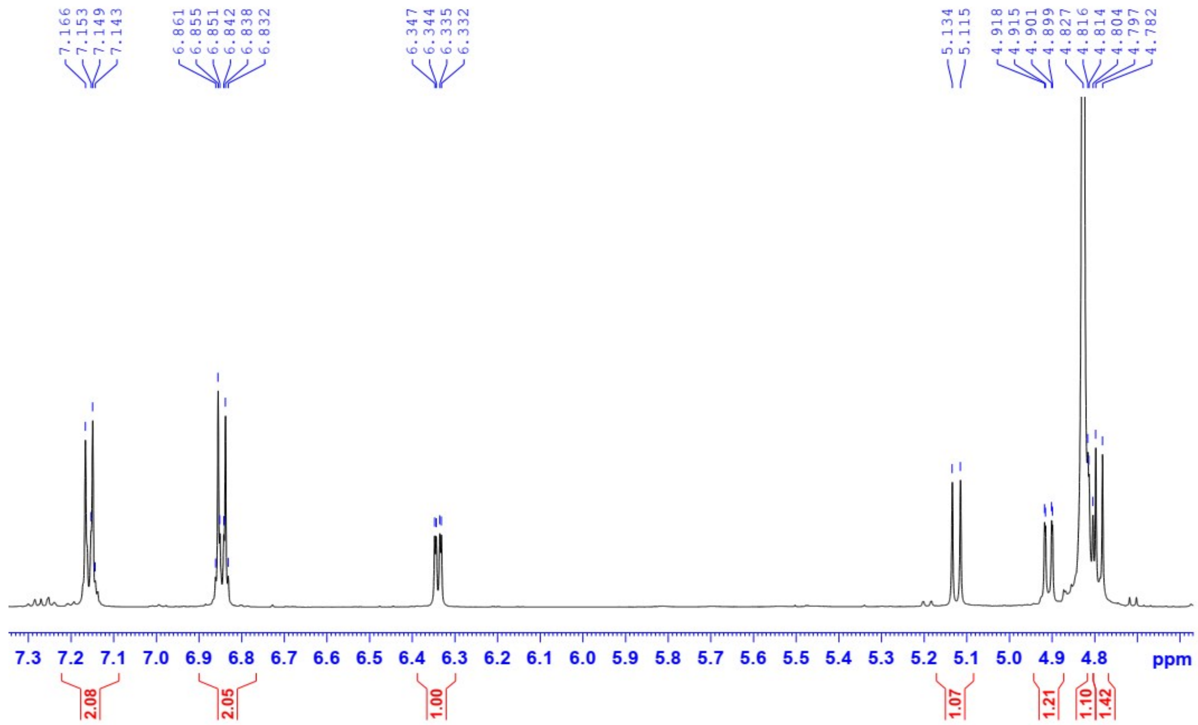

$^{1}$H-NMR spectrum (expansion) of compound **3**

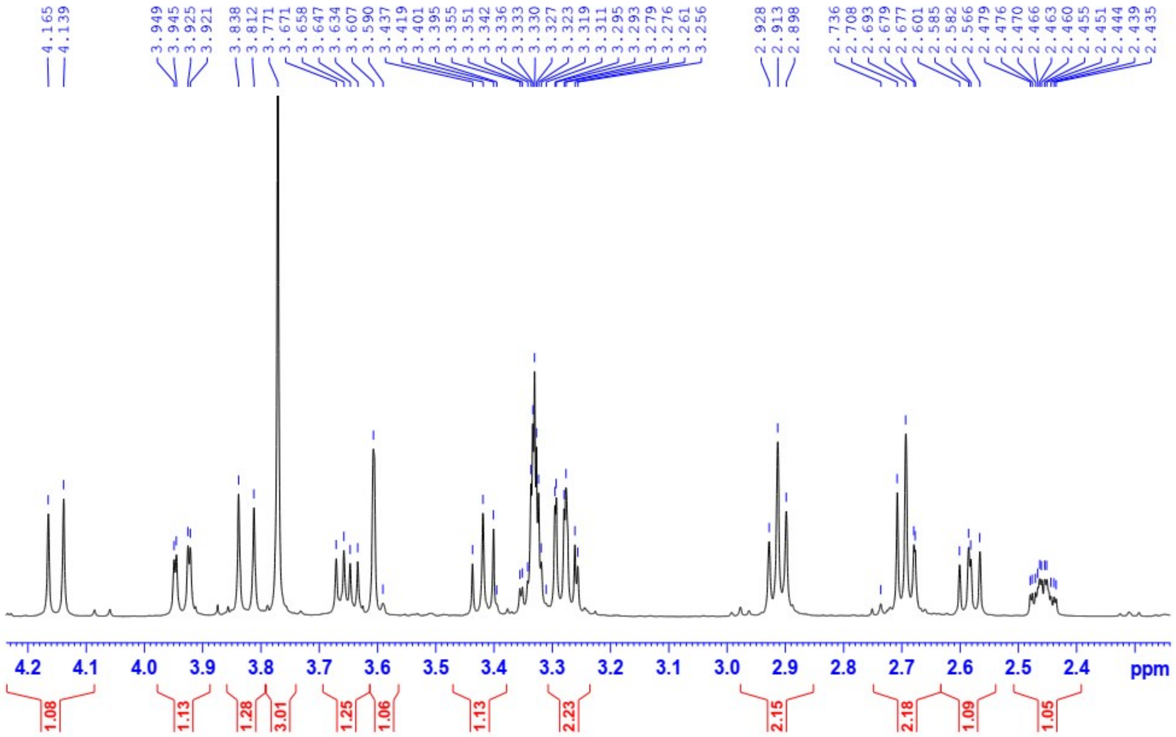


$^{1}$H-NMR spectrum (expansion) of compound **3**

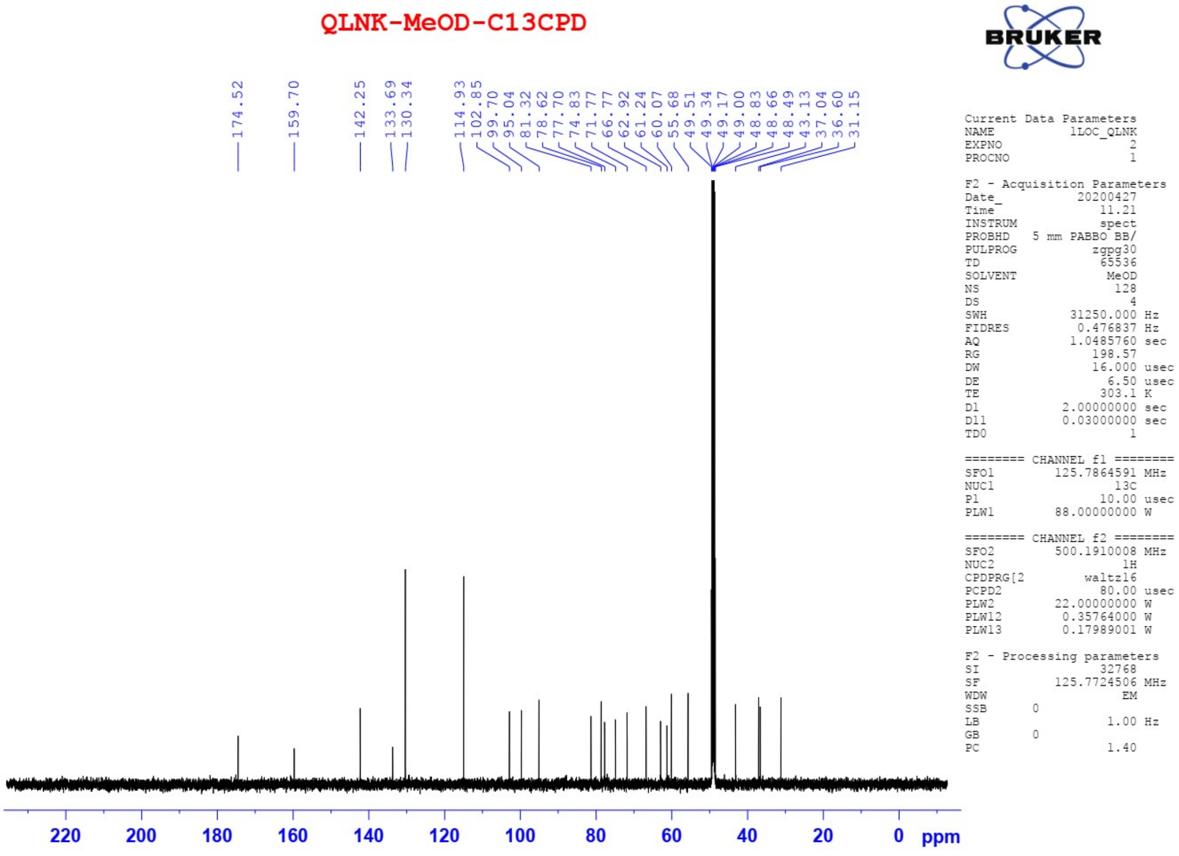


$^{13}$C-NMR spectrum of compound **3**

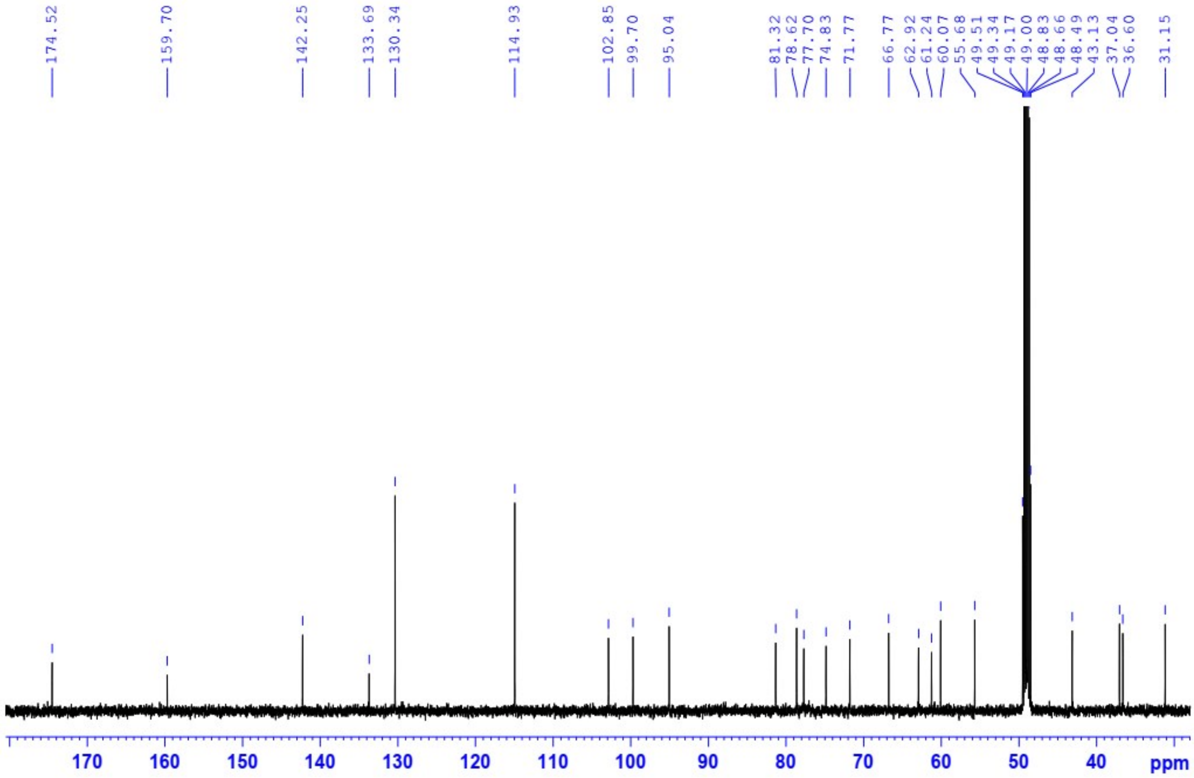


$^{13}$C-NMR spectrum (expansion) of compound **3**

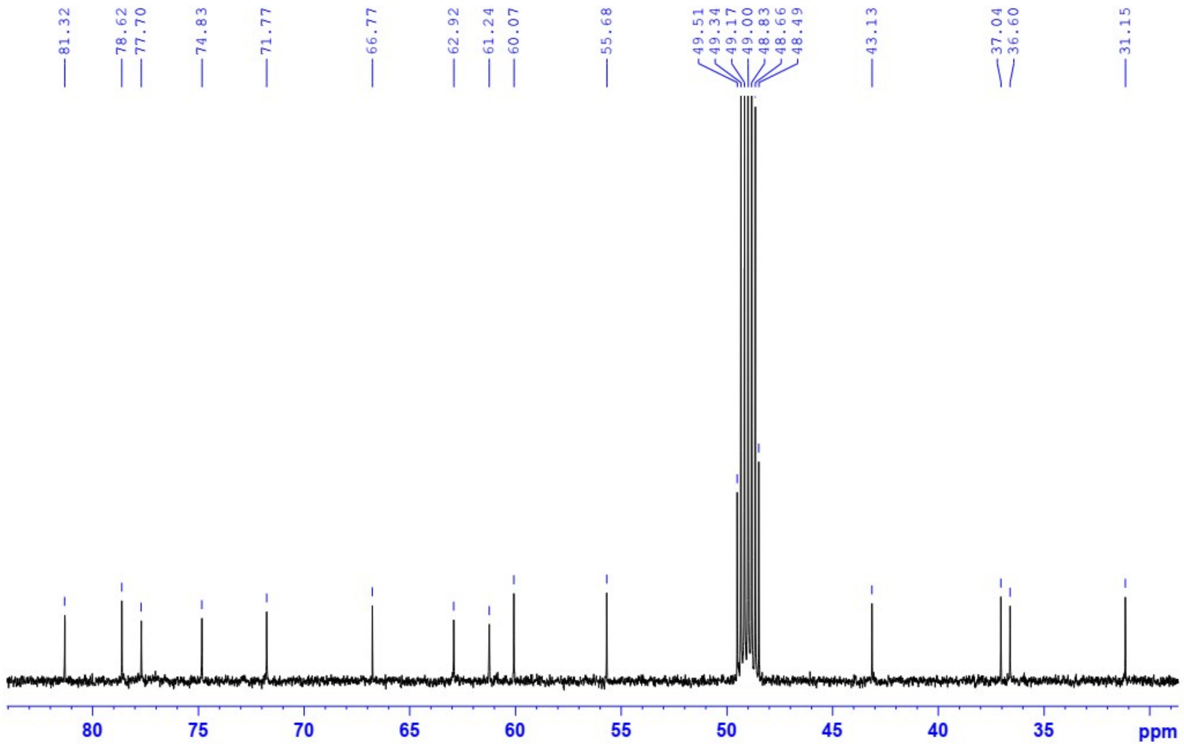


$^{13}$C-NMR spectrum (expansion) of compound **3**

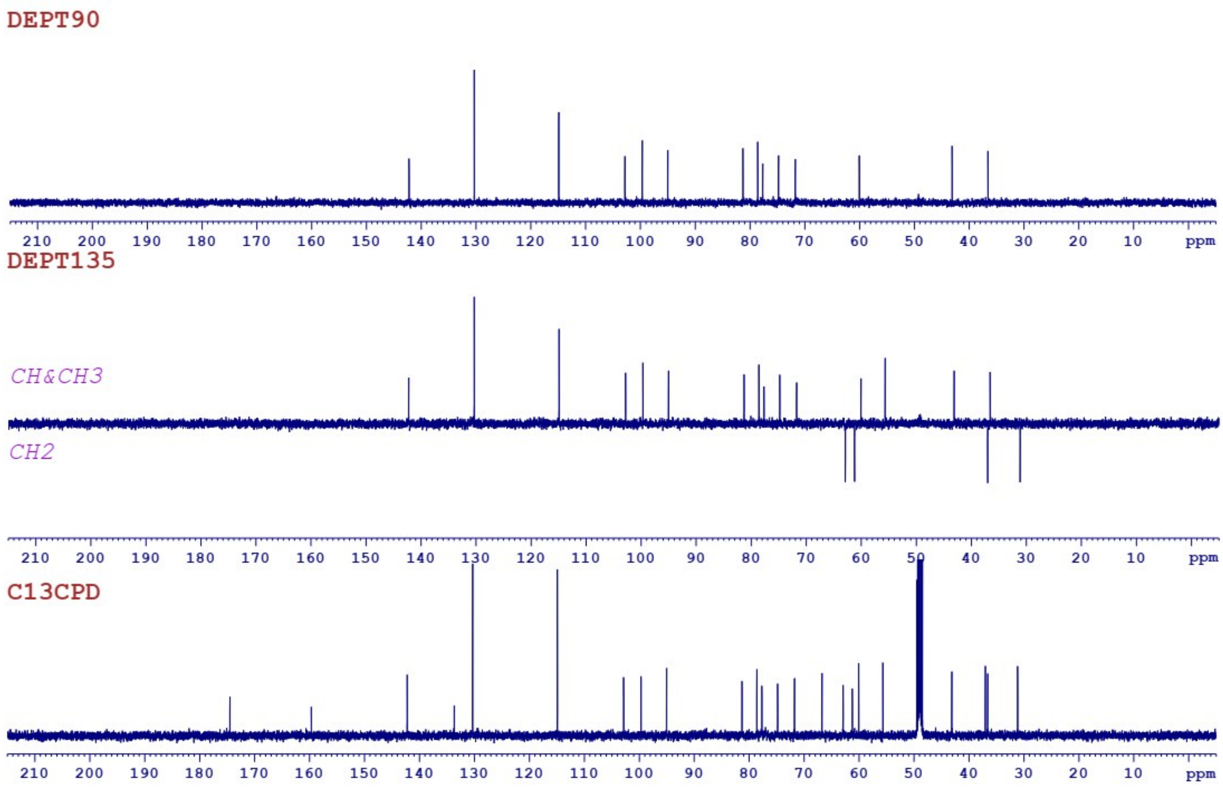


DEPT spectrum of compound **3**

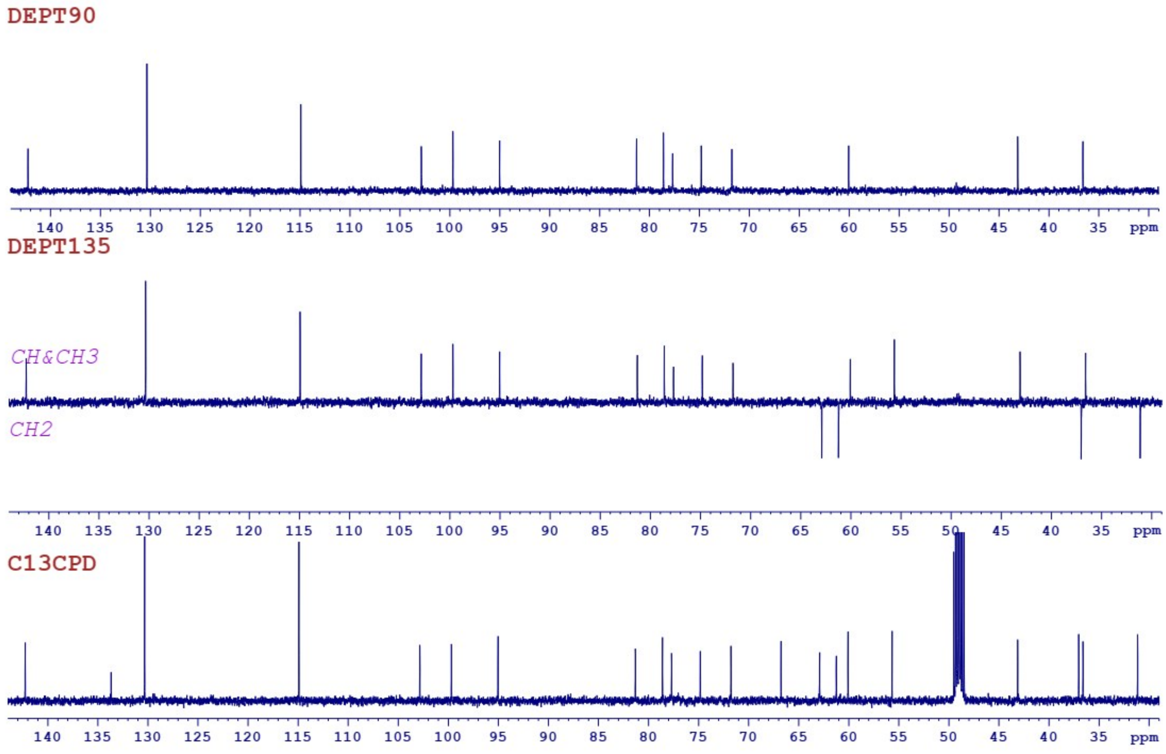


DEPT spectrum (expansion) of compound **3**

### 1.4. Compound 4

INSTITUTE OF CHEMISTRY
VIETNAM ACADEMY OF SCIENCE AND TECHNOLOGY

*Sample name: QN3K*
*Operator: Le Anh VHH*
*Method: +IDA TOF MS/MS*
*Date: 2021.02.26*

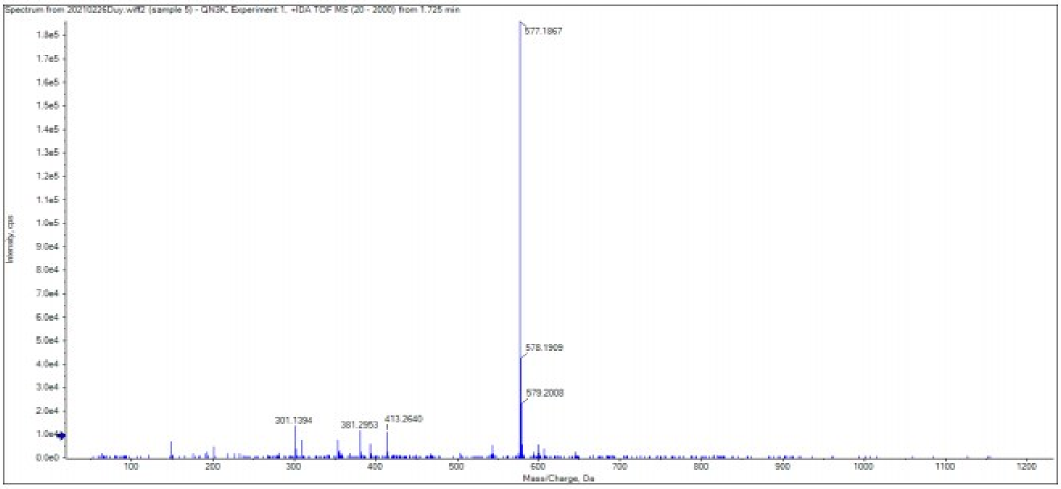


| Hit | Formula | m/z | RDB | ppm | MS Rank | MSMS ppm | MSMS Rank | Found |
|---|---|---|---|---|---|---|---|---|
| 1 | C26H34O13 | 577.18916 | 10.0 | -4.3 | 1 | | | NA/NA |

*Device Model:* SCIEX X500 QTOF

HR-ESI-MS spectrum of compound **4**

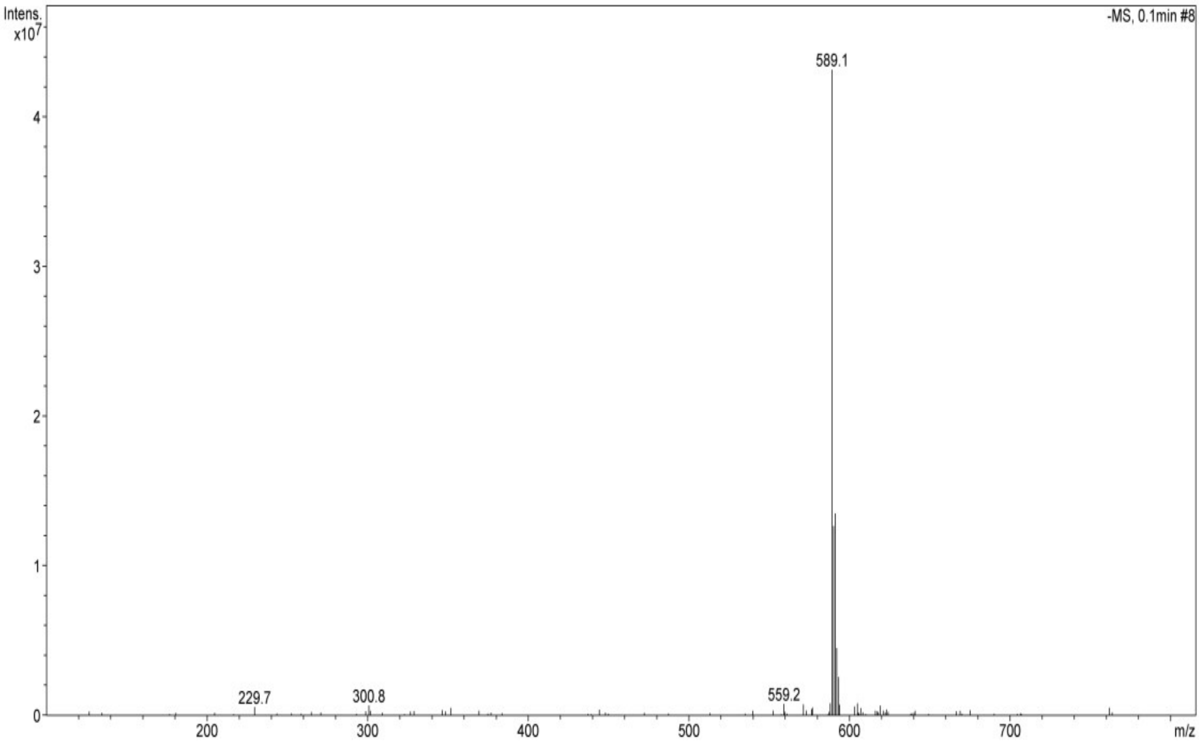


(-)-ESI-MS spectrum of compound **4**

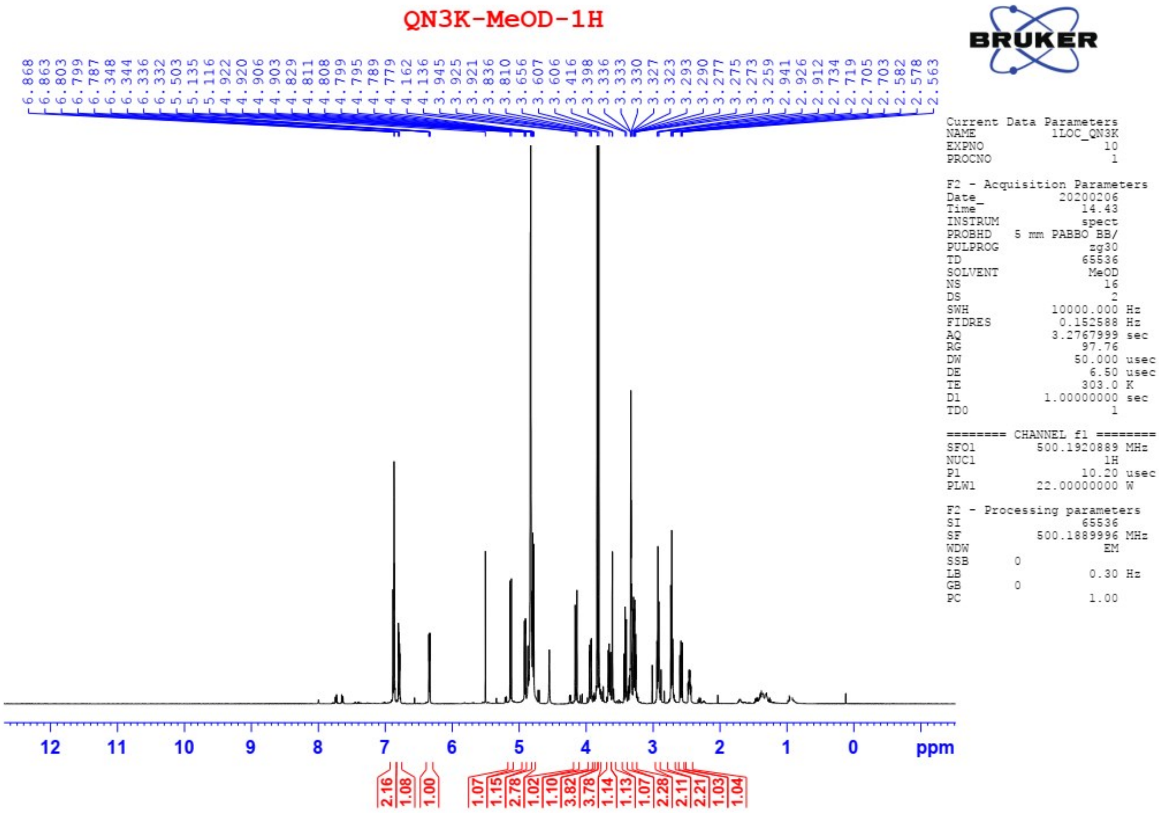

$^{1}$H-NMR spectrum of compound **4**

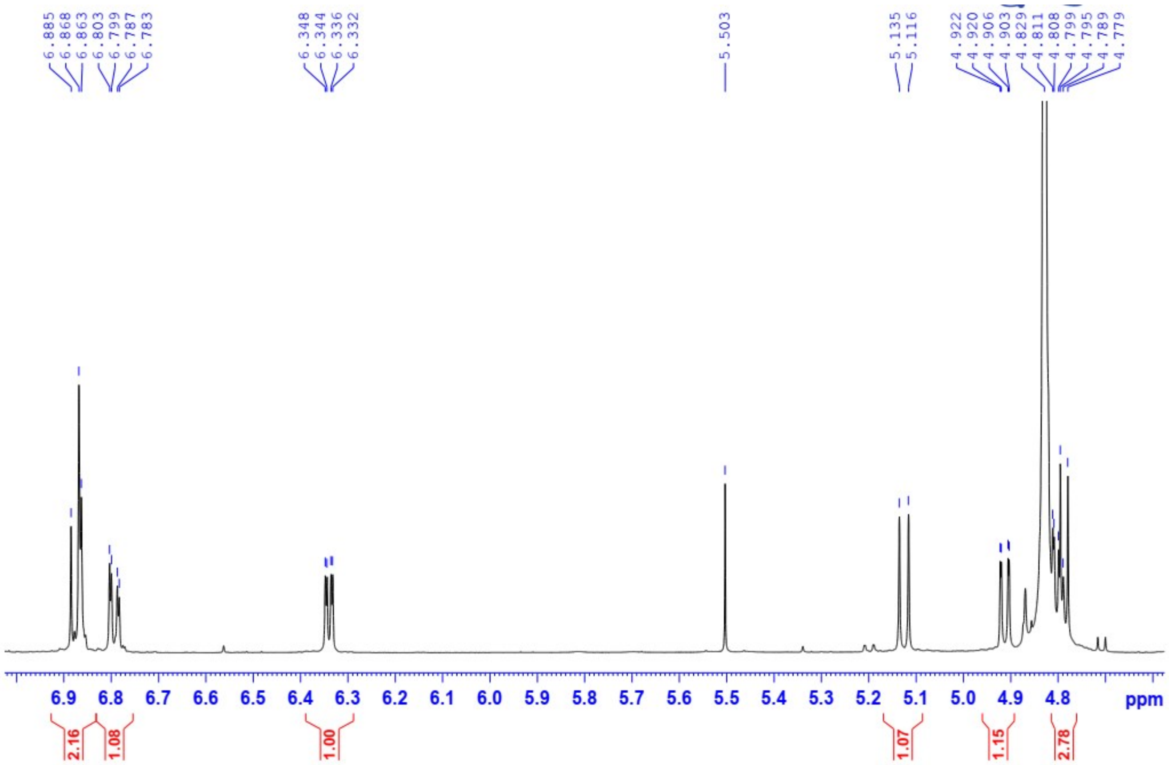

$^{1}$H-NMR spectrum (expansion) of compound **4**

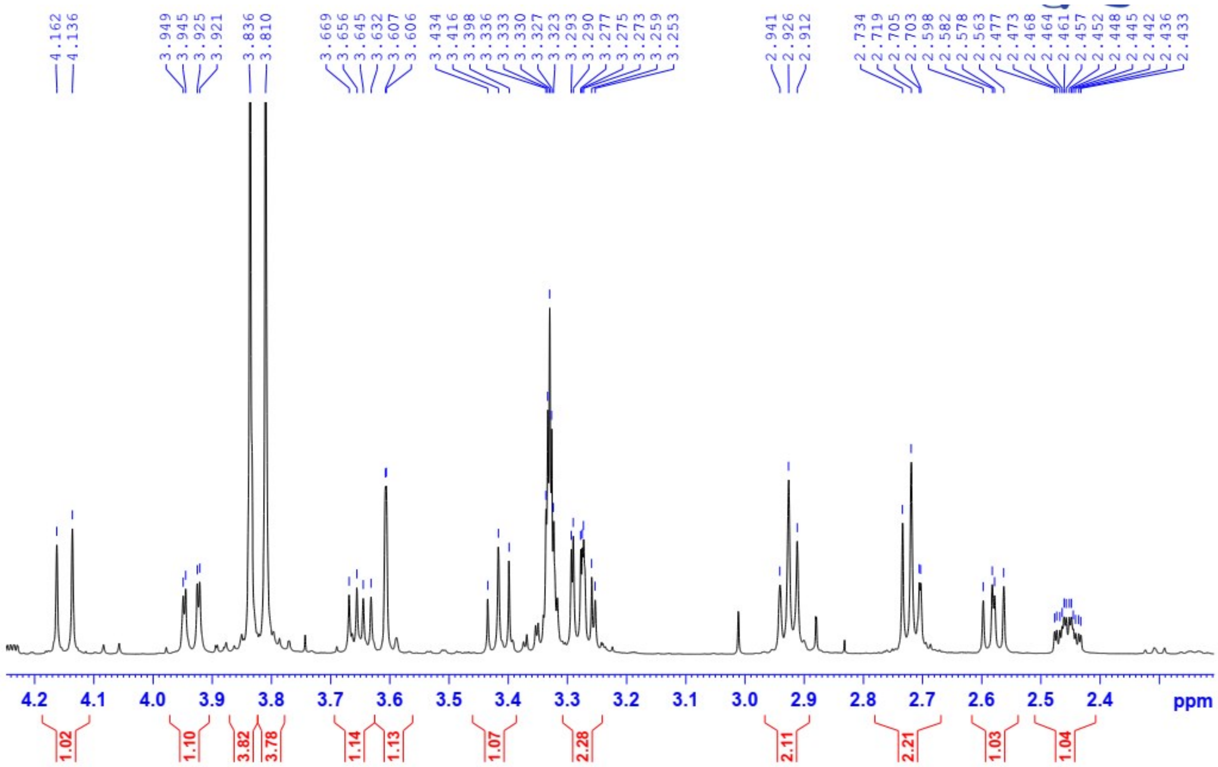


$^{1}$H-NMR spectrum (expansion) of compound **4**

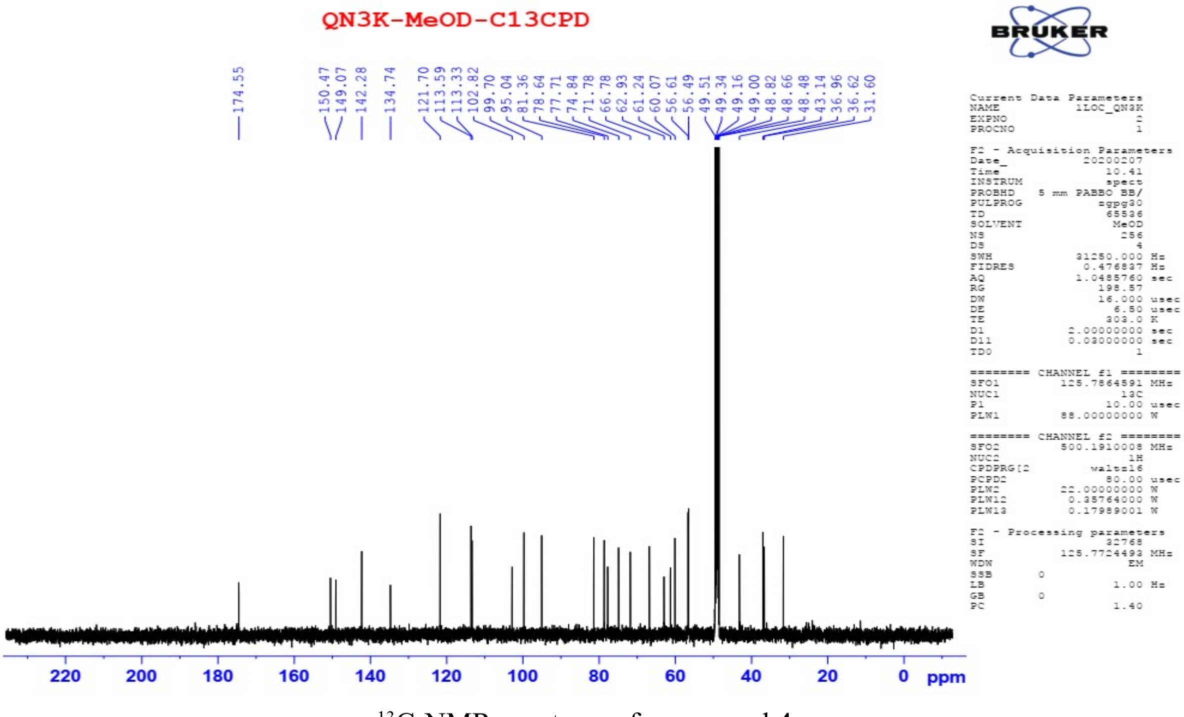


$^{13}$C-NMR spectrum of compound **4**

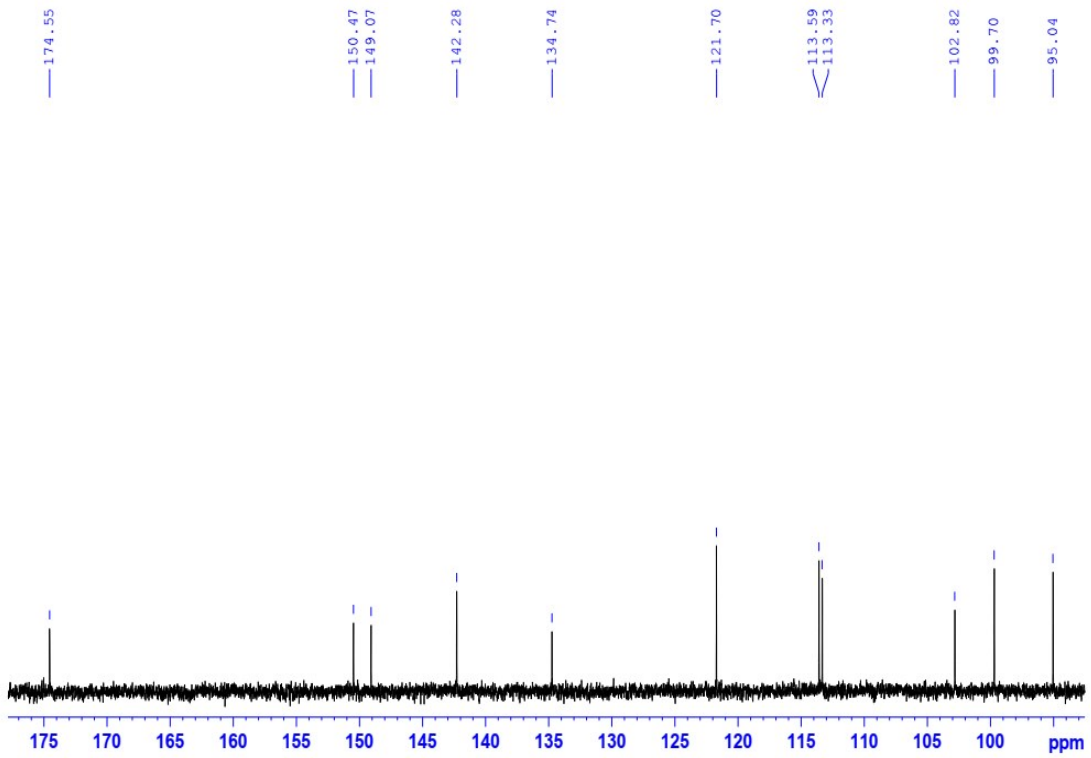


$^{13}$C-NMR spectrum (epansion) of compound **4**

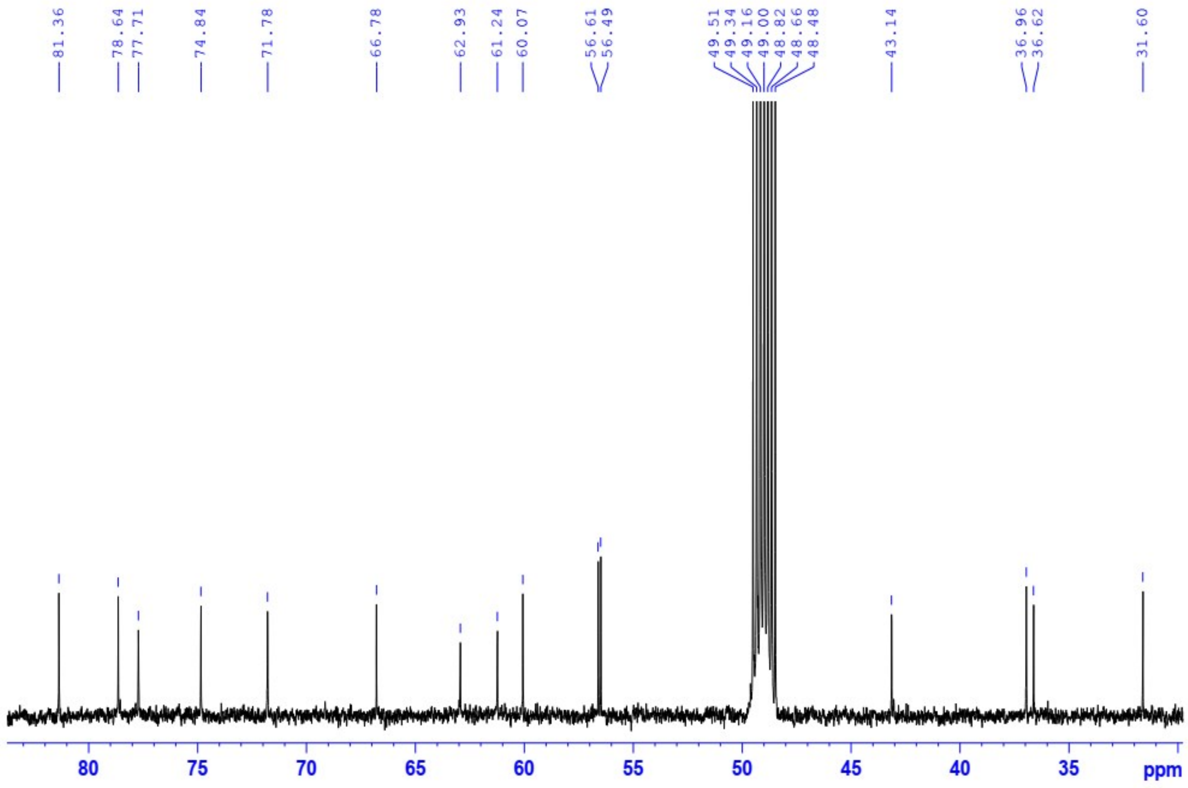


$^{13}$C-NMR spectrum (epansion) of compound **4**

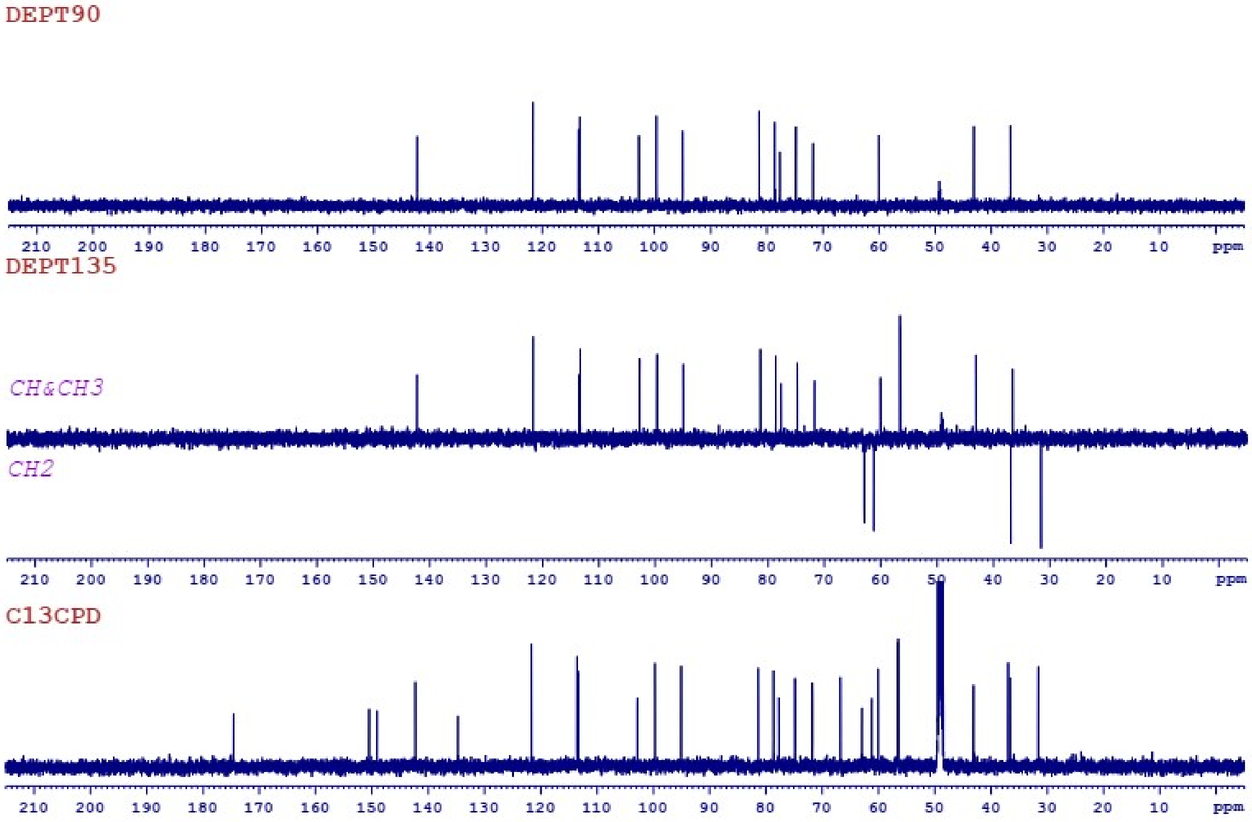


DEPT spectrum of compound **4**

## 1.5. Compound 5

INSTITUTE OF CHEMISTRY
VIETNAM ACADEMY OF SCIENCE AND TECHNOLOGY

***Sample name: QN3KAc***
***Operator: Le Anh VHH***
***Method: +IDA TOF MS/MS***
***Date: 2021.02.26***

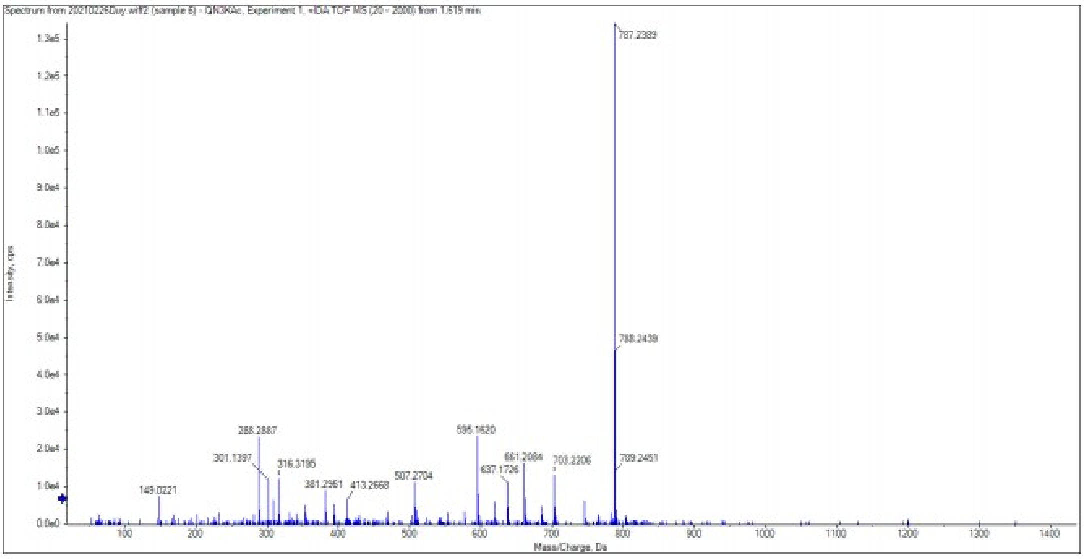


| Hit | Formula | m/z | RDB | ppm | MS Rank | MSMS ppm | MSMS Rank | Found |
|---|---|---|---|---|---|---|---|---|
| 1 | C36H44O18 | 787.24199 | 15.0 | -3.9 | 1 | | | NA/NA |

*Device Model:* SCIEX X500 QTOF

HR-ESI-MS spectrum of compound **5**

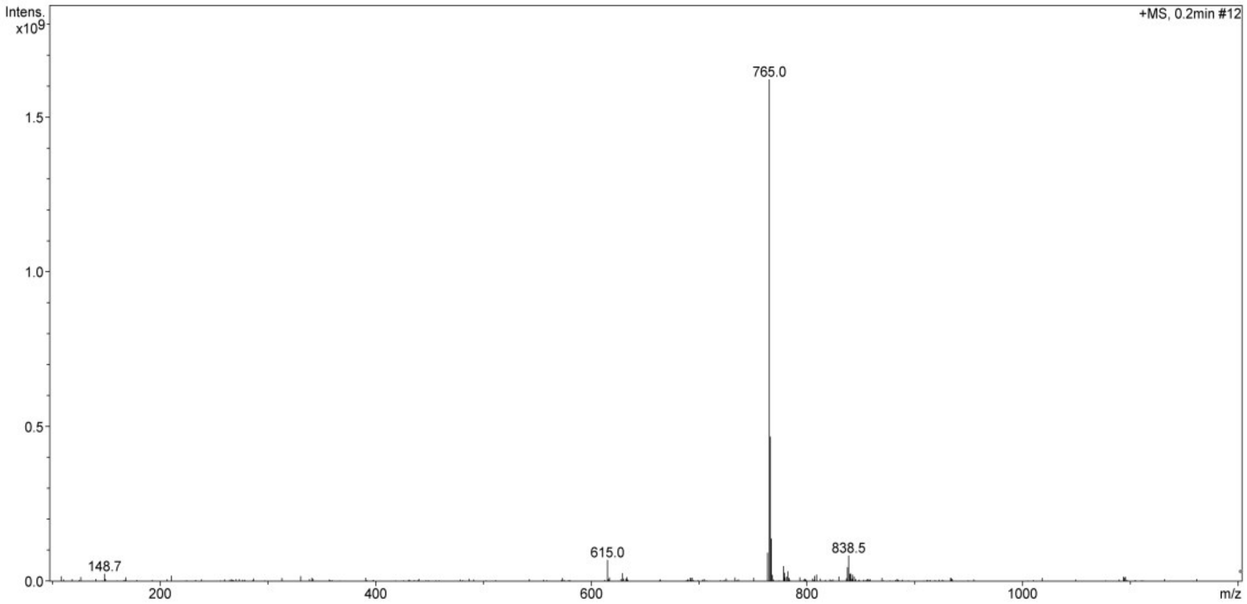


(+)-ESI-MS spectrum of compound **5**

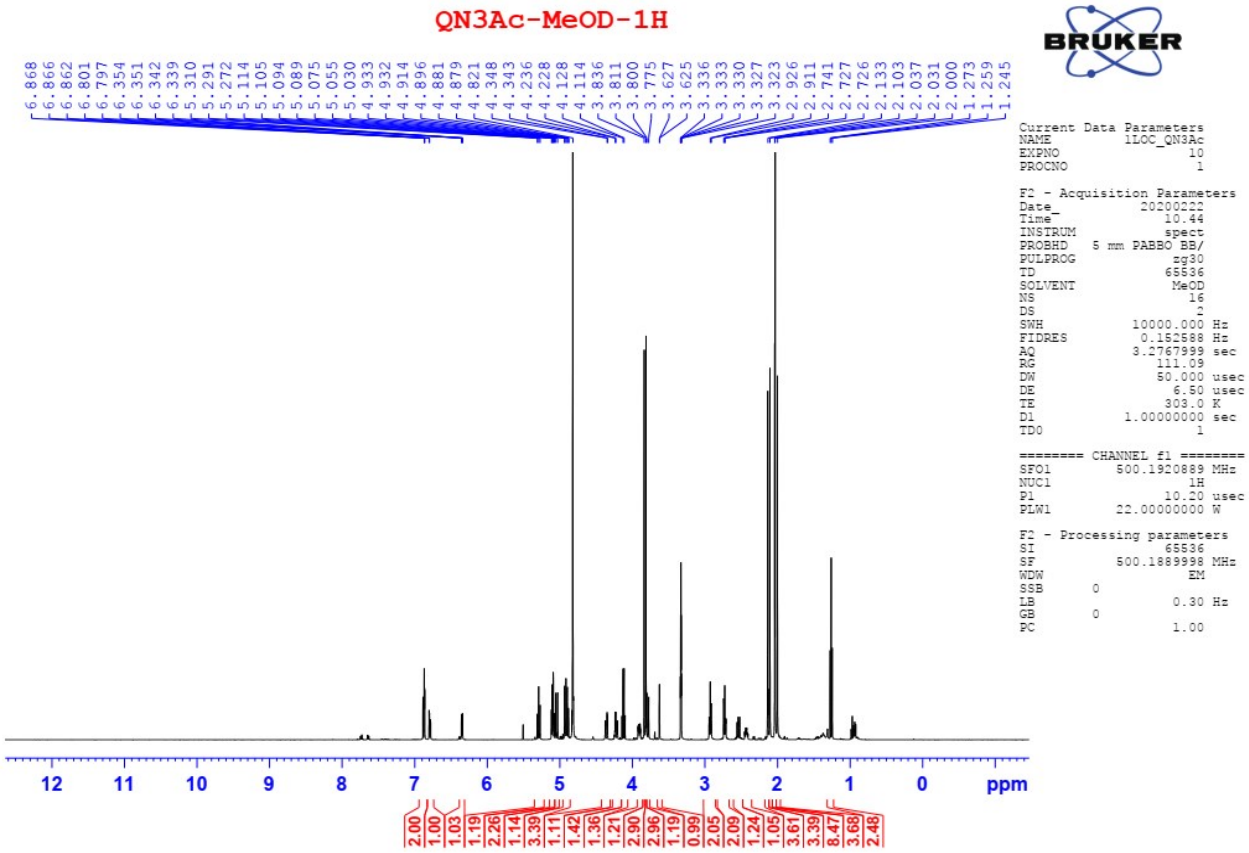


$^{1}$H-NMR spectrum of compound **5**

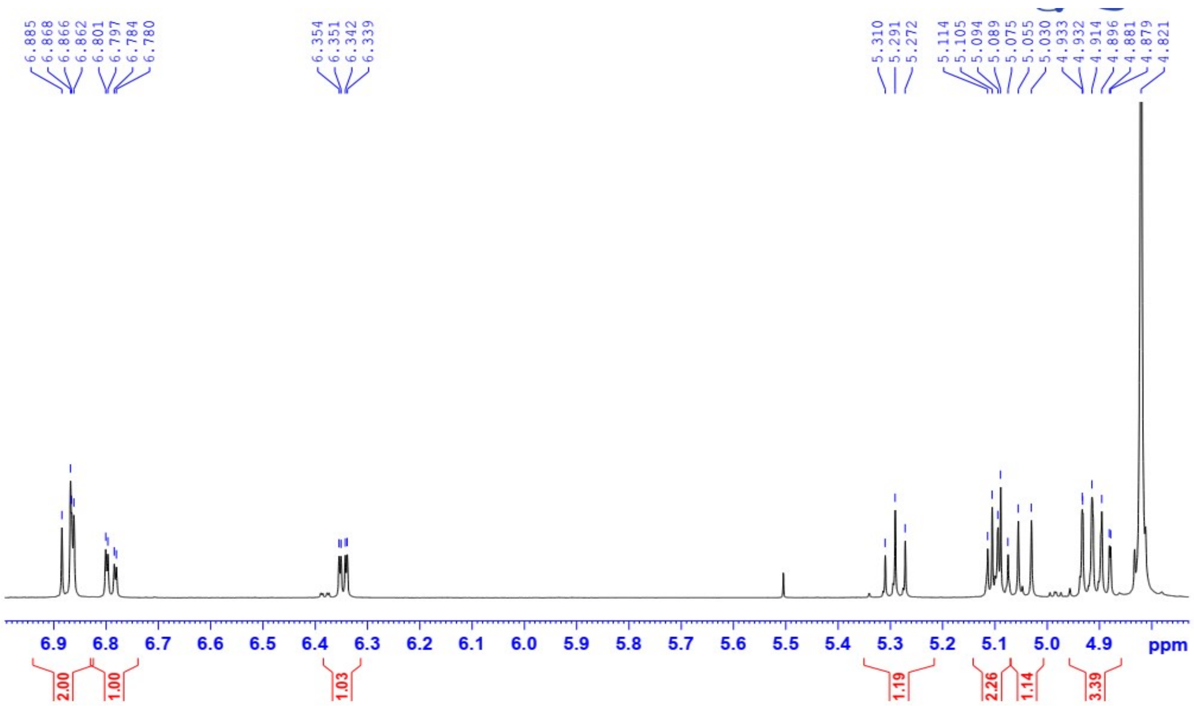

$^{1}$H-NMR spectrum (expansion) of compound **5**

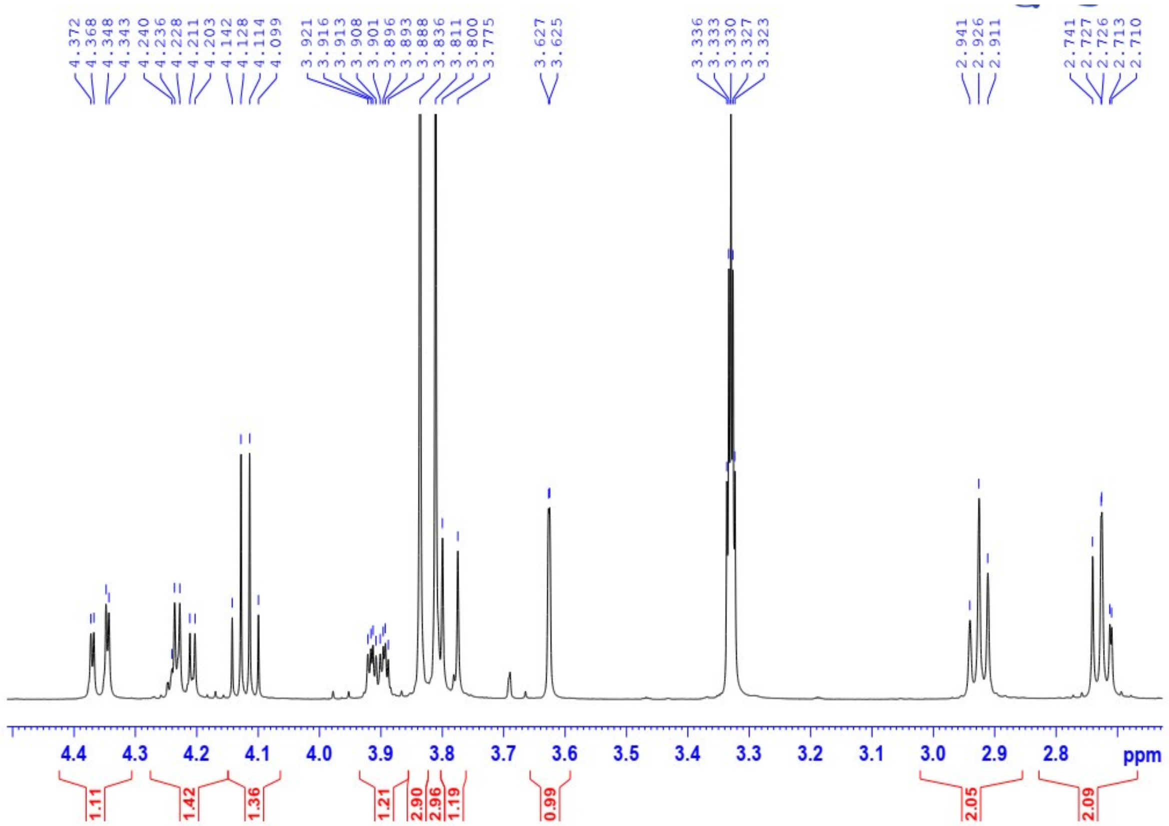

$^{1}$H-NMR spectrum (expansion) of compound **5**

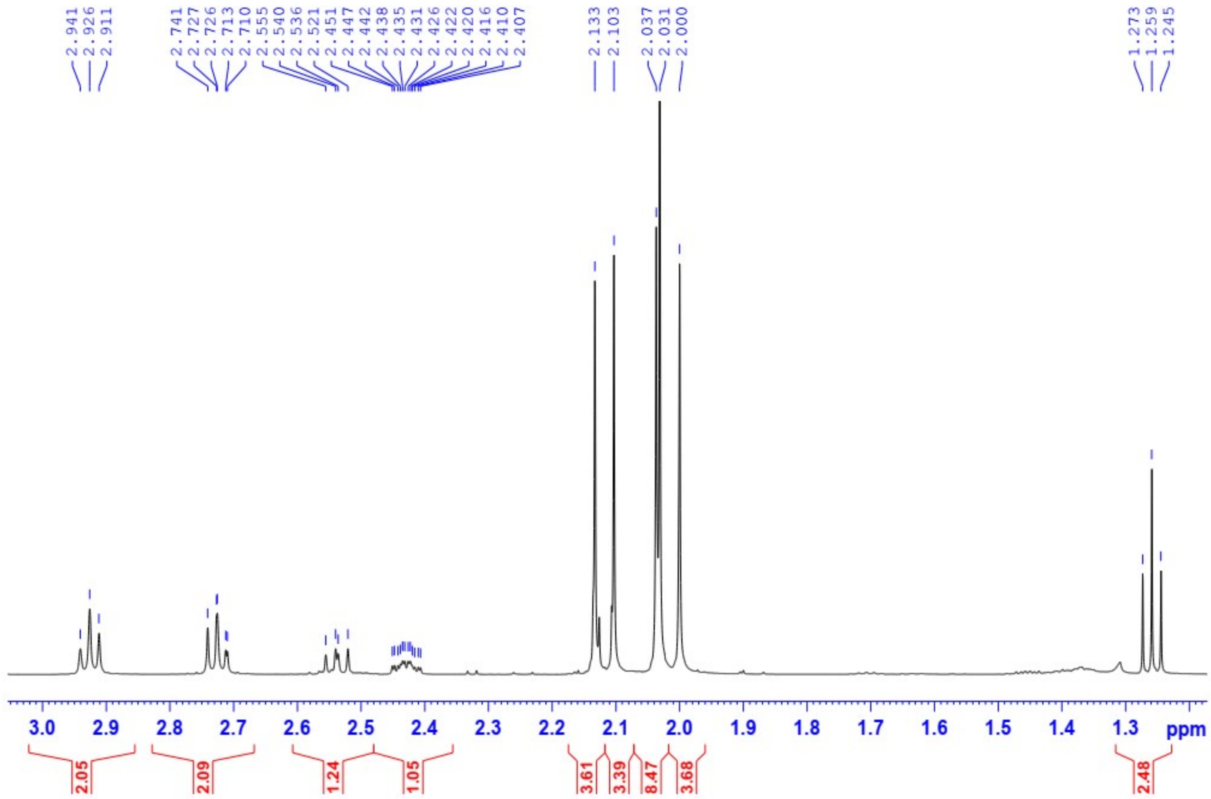

$^{1}$H-NMR spectrum (expansion) of compound **5**

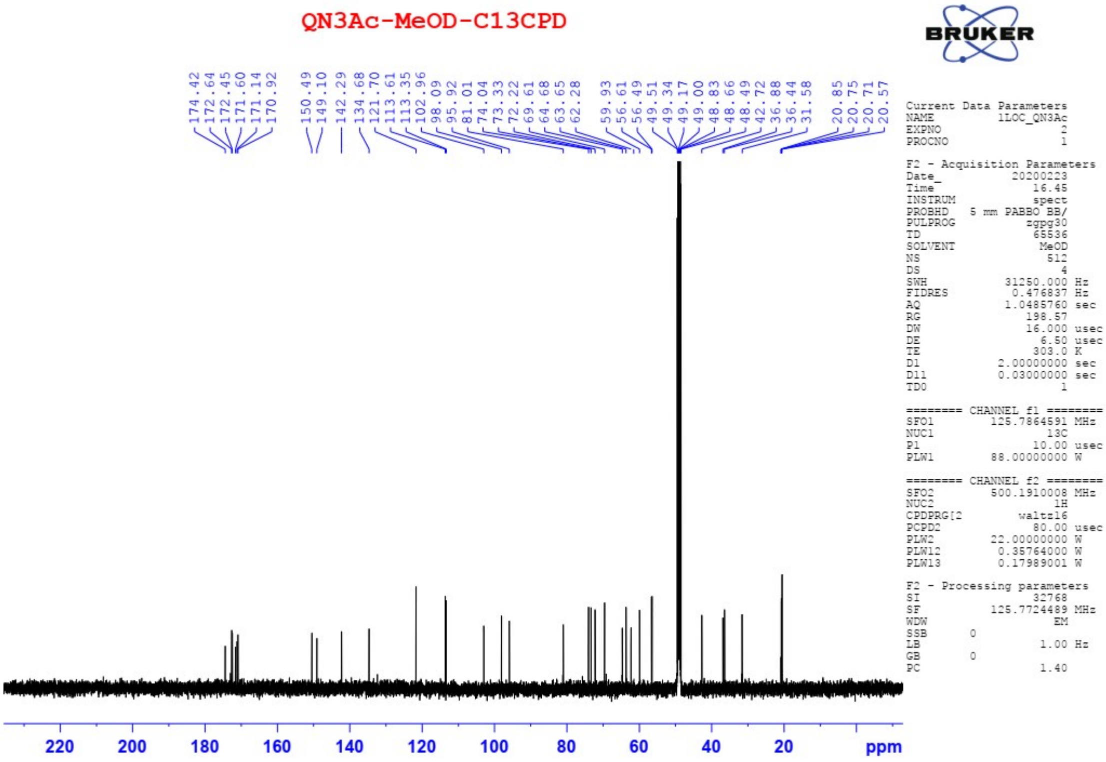


$^{13}$C-NMR spectrum of compound **5**

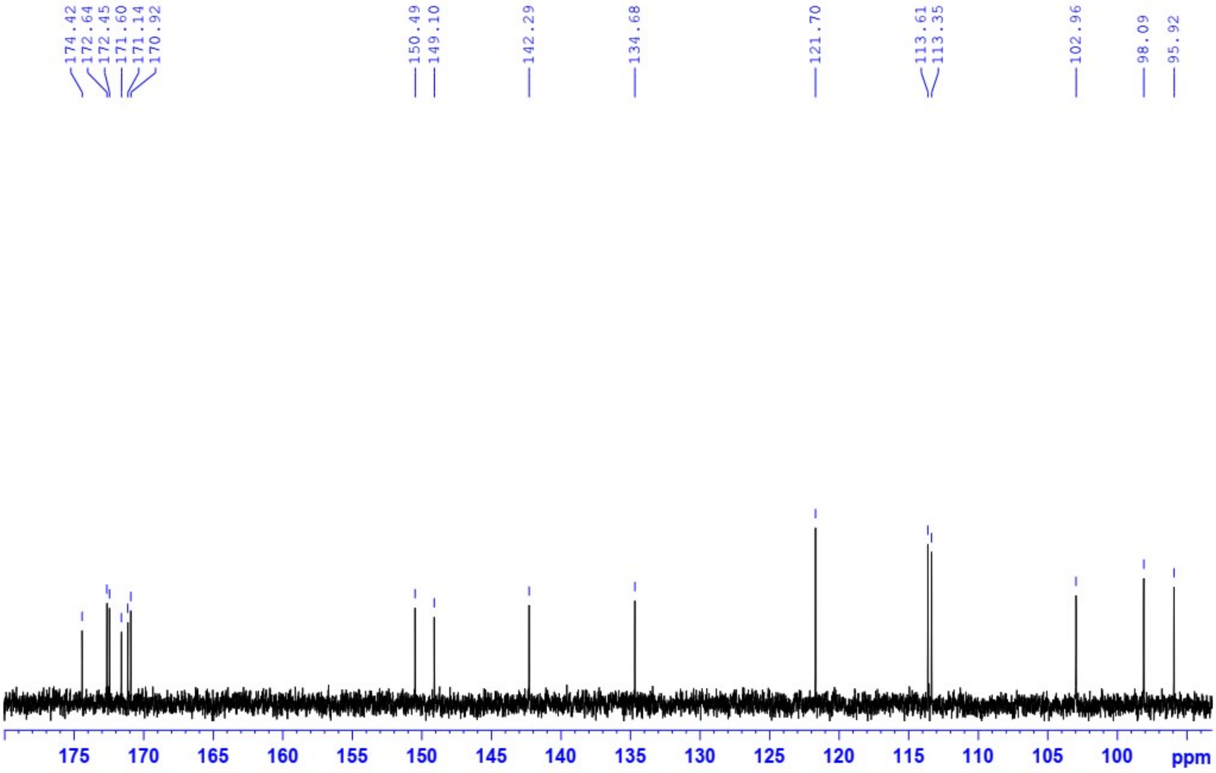


$^{13}$C-NMR spectrum (expansion) of compound **5**

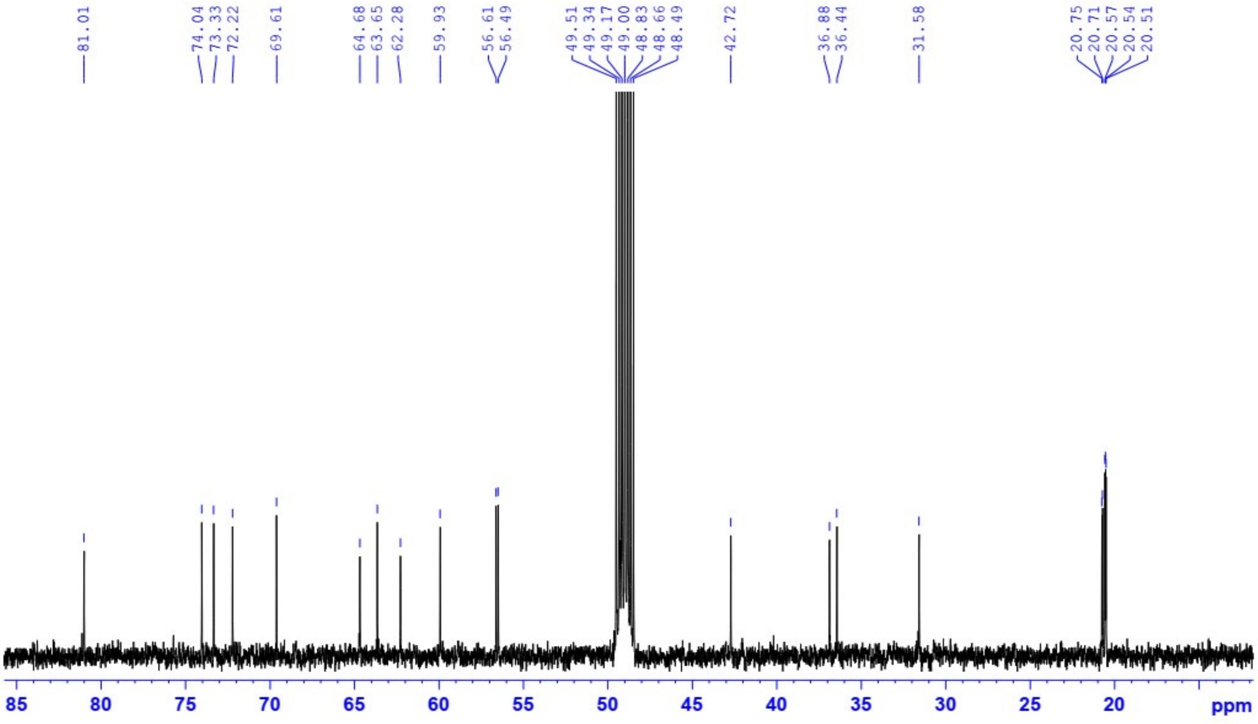


$^{13}$C-NMR spectrum (expansion) of compound **5**

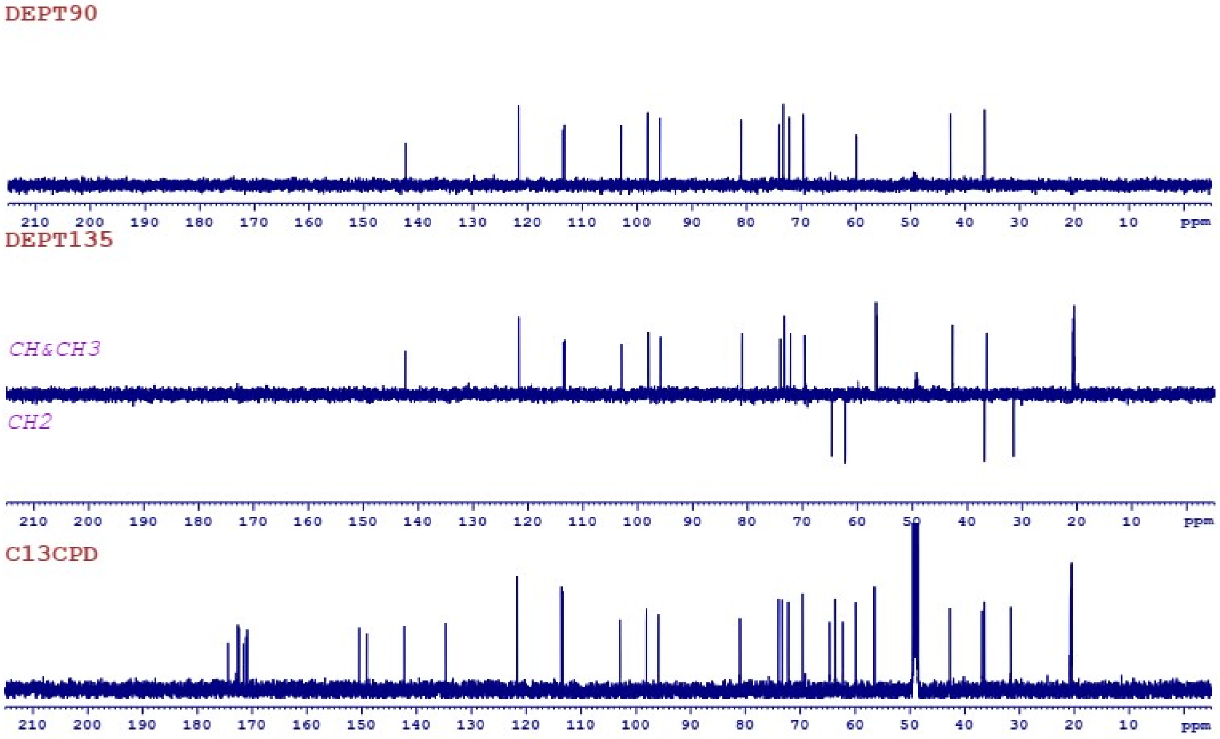


DEPT spectrum of compound **5**

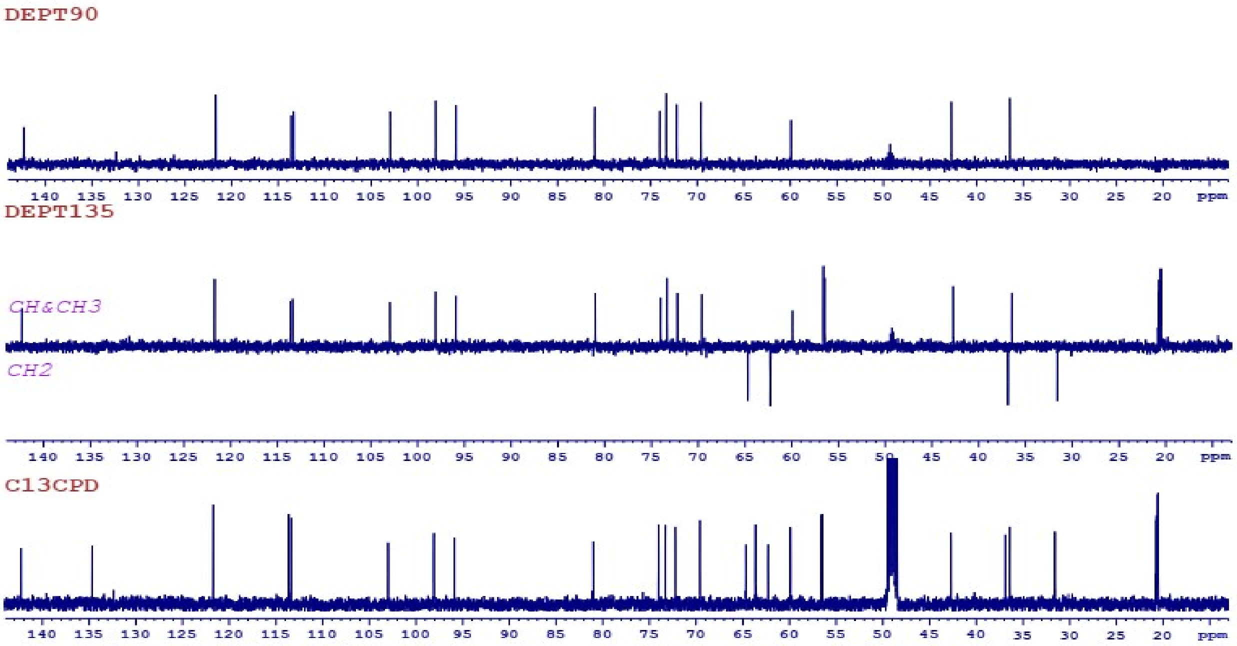


DEPT spectrum (epansion) of compound **5**

## 1.6. Compound 6

INSTITUTE OF CHEMISTRY
VIETNAM ACADEMY OF SCIENCE AND TECHNOLOGY

***Sample name:* QN3Ph**
***Operator:* Le Anh VHH**
***Method:* +IDA TOF MS/MS**
***Date:* 2021.02.26**

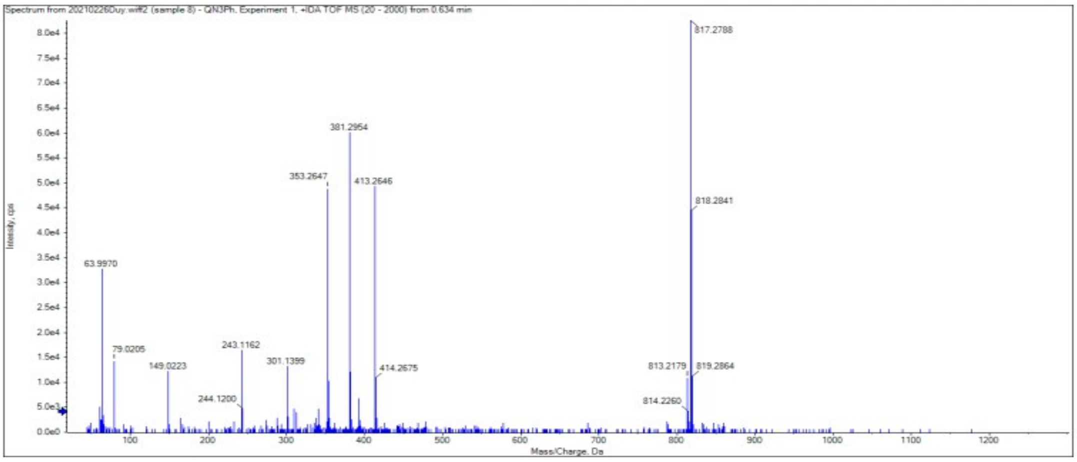


| Hit | Formula | m/z | RDB | ppm | MS Rank | MSMS ppm | MSMS Rank | Found |
|---|---|---|---|---|---|---|---|---|
| 1 | C45H46O13 | 817.28306 | 23.0 | -5.2 | 1 | | | NA/NA |

*Device Model:* SCIEX X500 QTOF

HR-ESI-MS spectrum of compound **6**

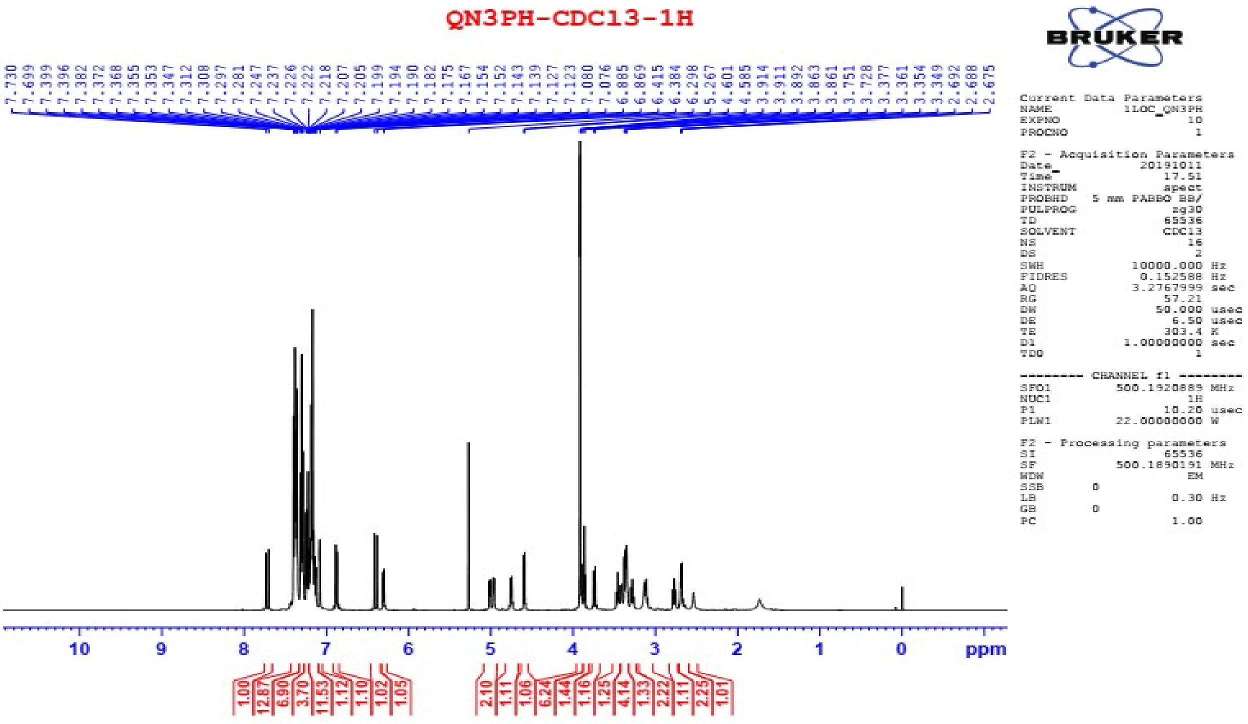


$^{1}$H-NMR spectrum of compound **6**

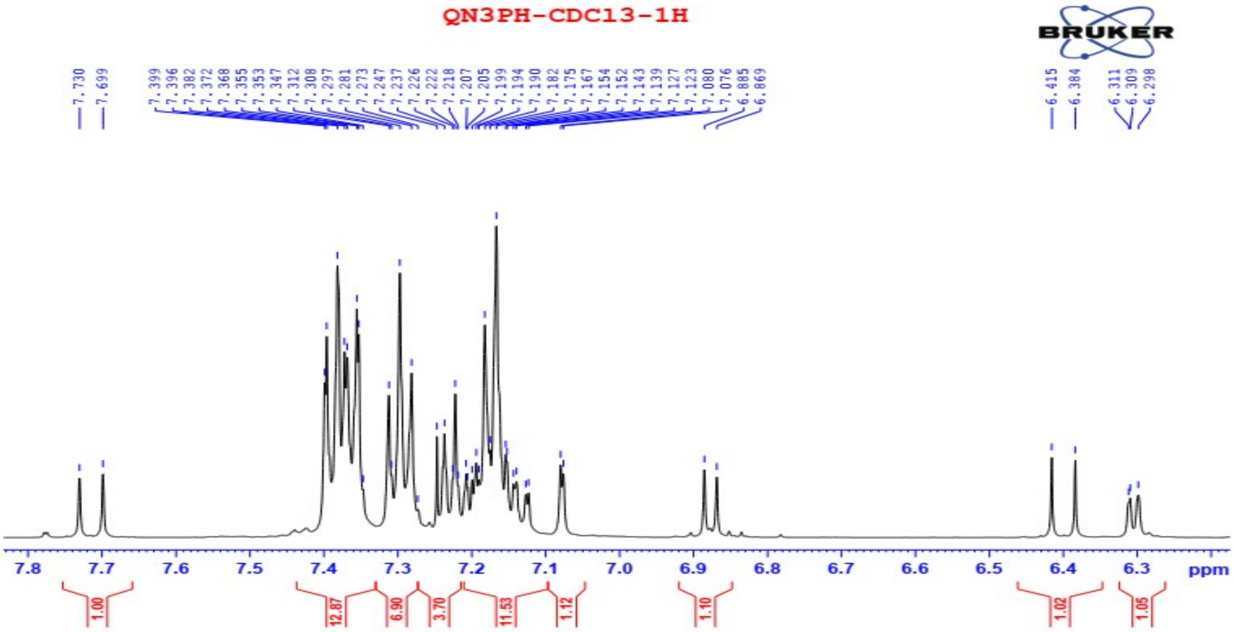


$^{1}$H-NMR spectrum (expansion) of compound **6**

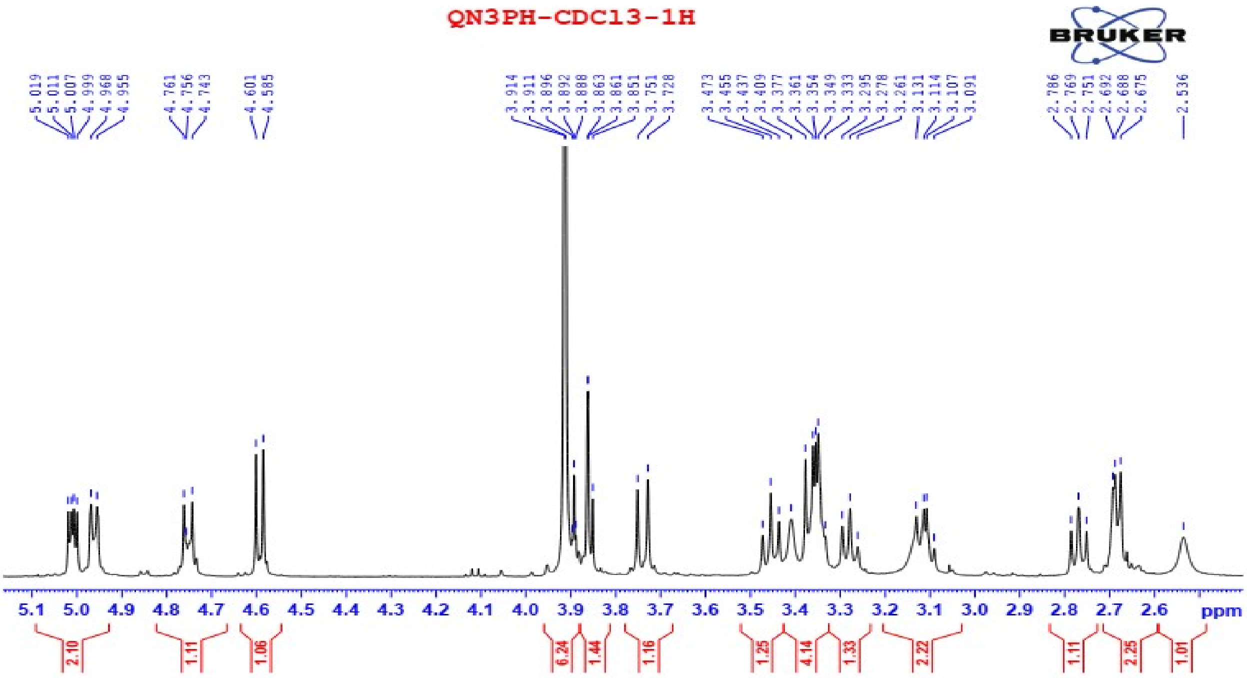


$^{1}$H-NMR spectrum (expansion) of compound **6**

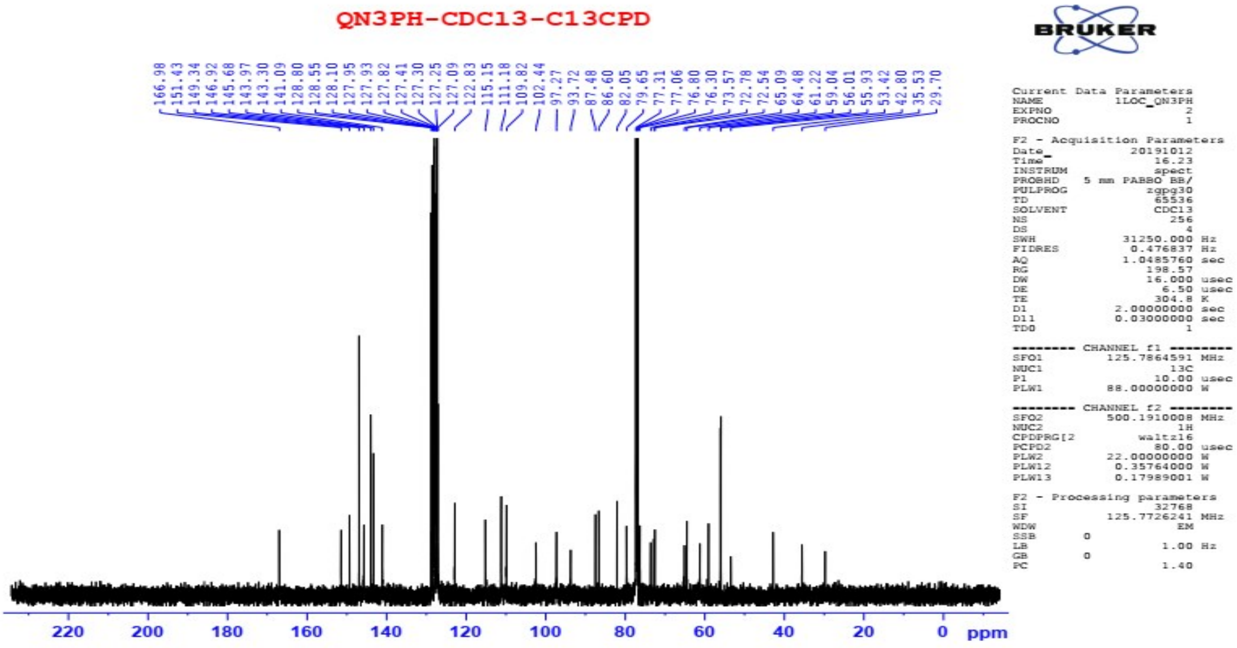


$^{13}$C-NMR spectrum of compound **6**

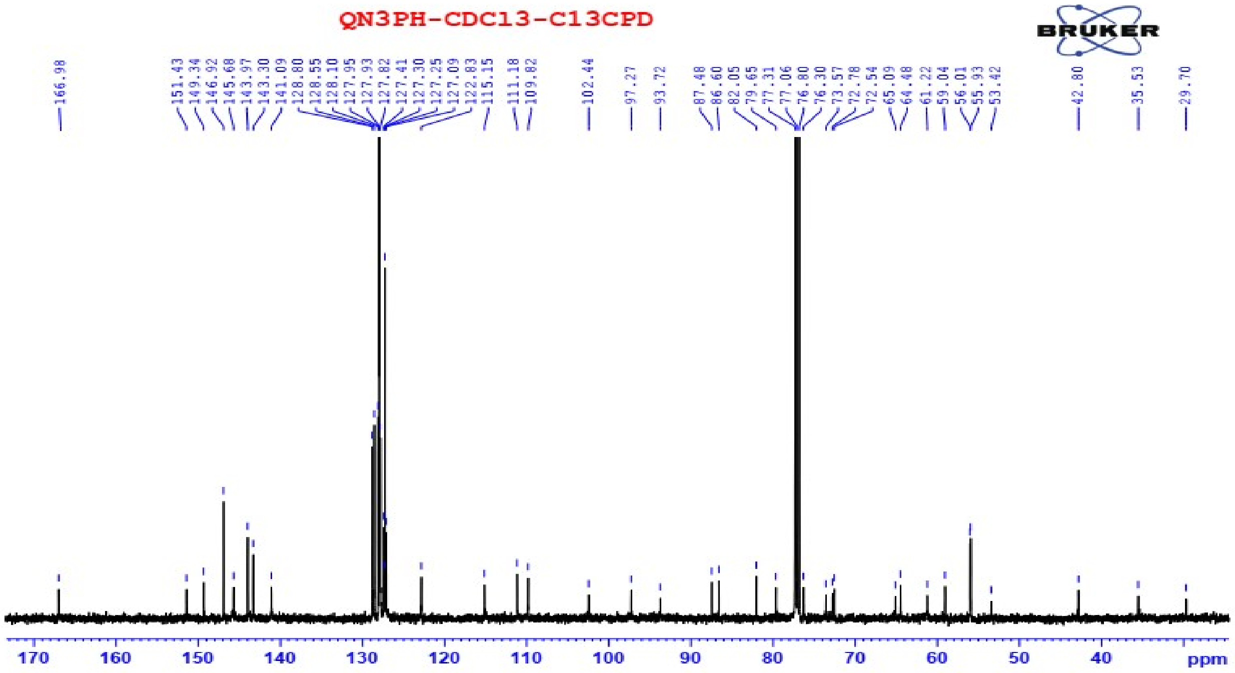


$^{13}$C-NMR spectrum (expansion) of compound **6**

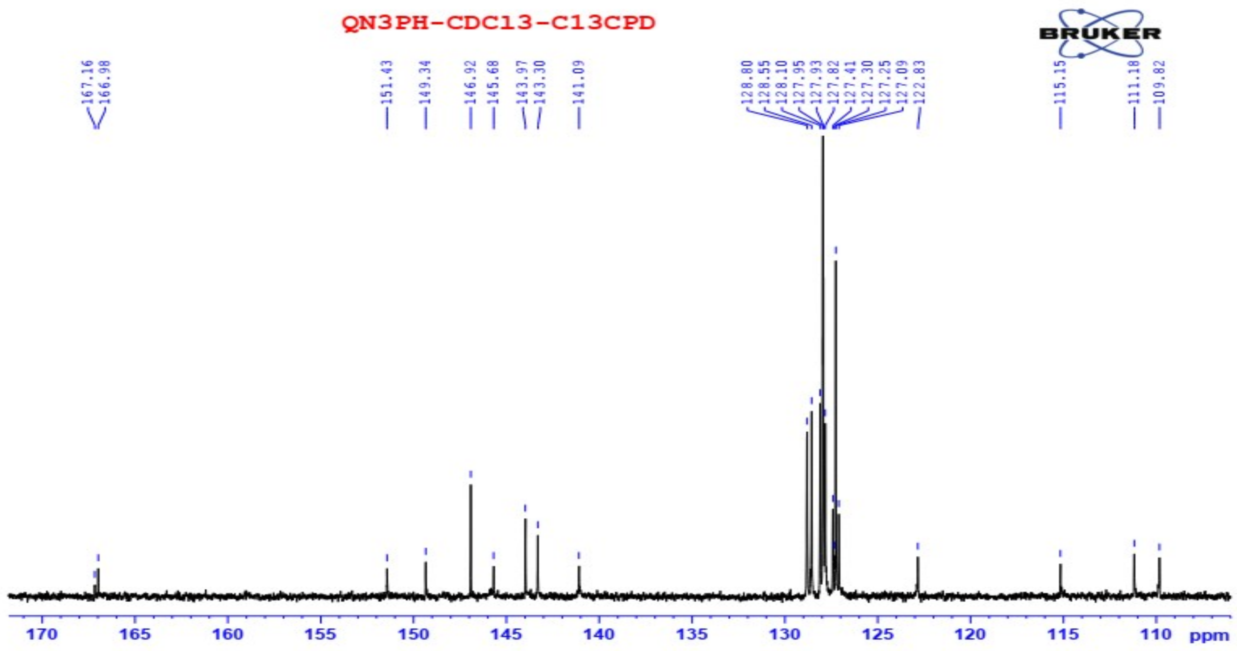


$^{13}$C-NMR spectrum (expansion) of compound **6**

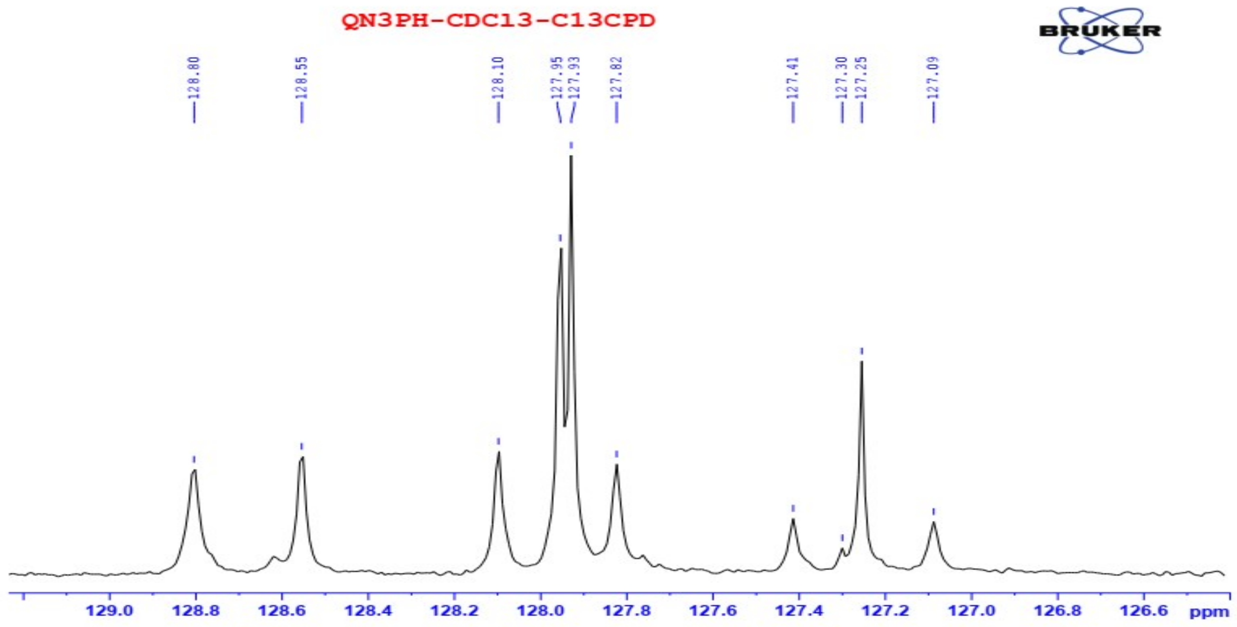


$^{13}$C-NMR spectrum (expansion) of compound **6**

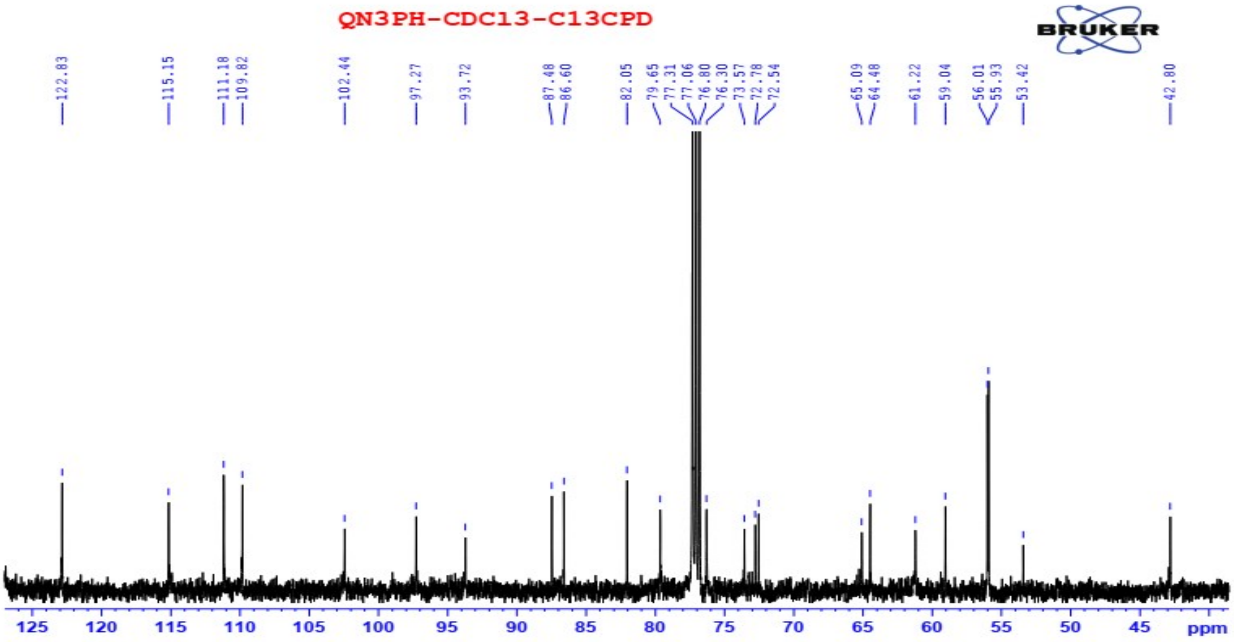


$^{13}$C-NMR spectrum (expansion) of compound **6**

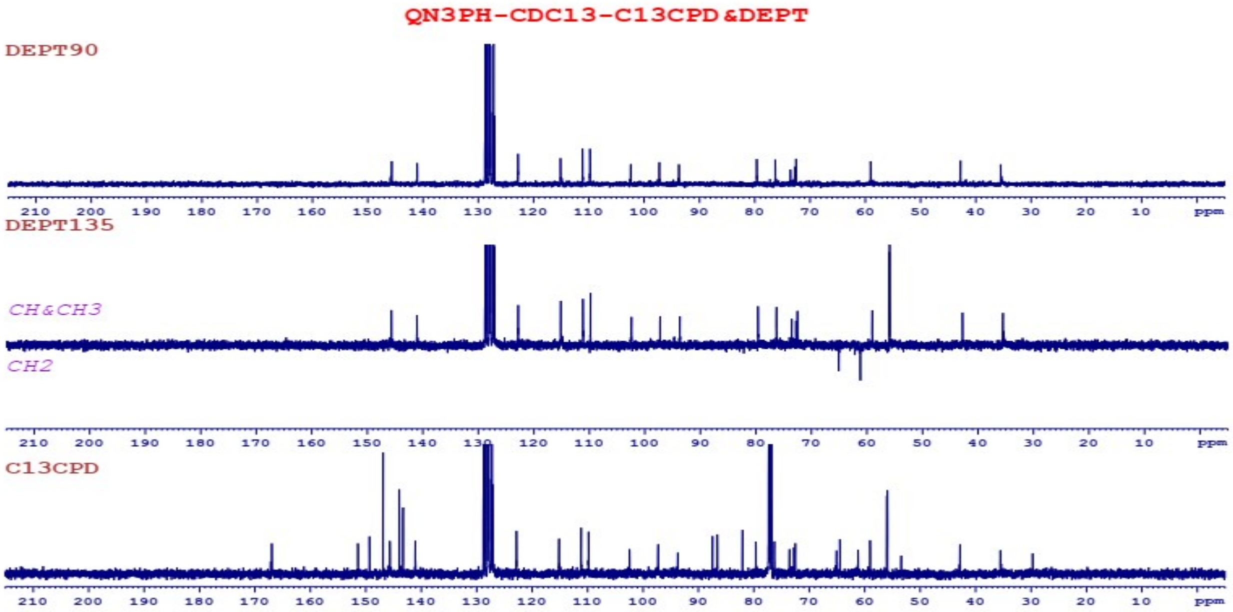


DEPT spectrum of compound **6**

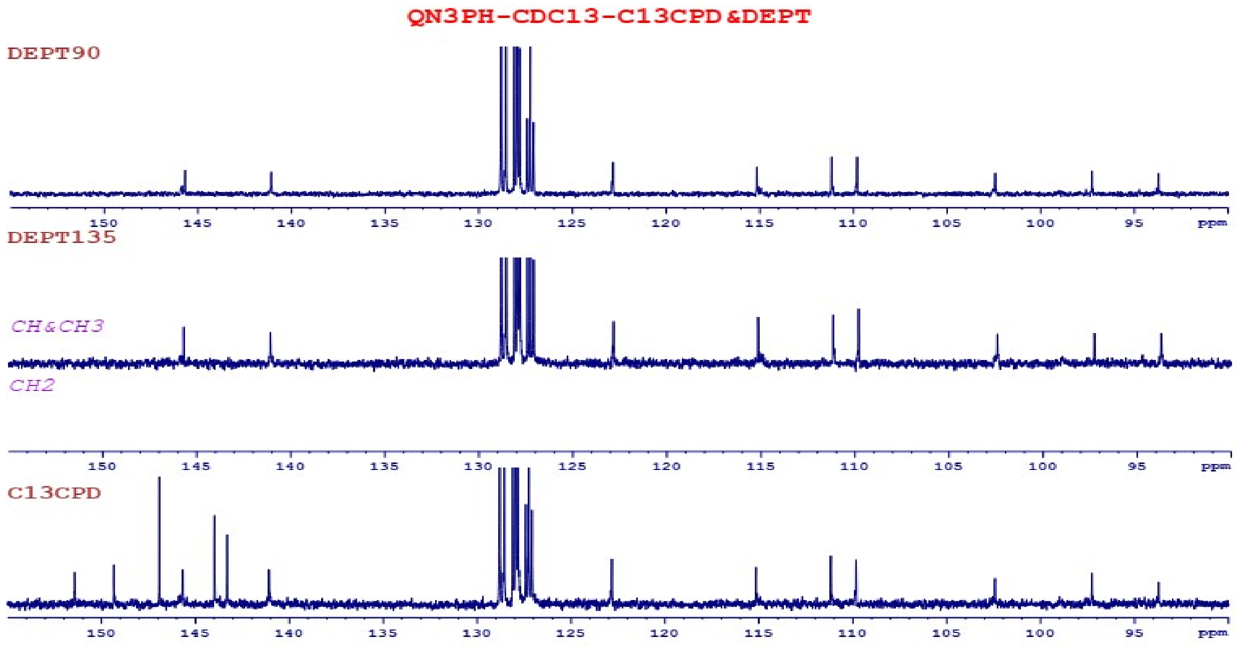


DEPT spectrum (epansion) of compound **6**

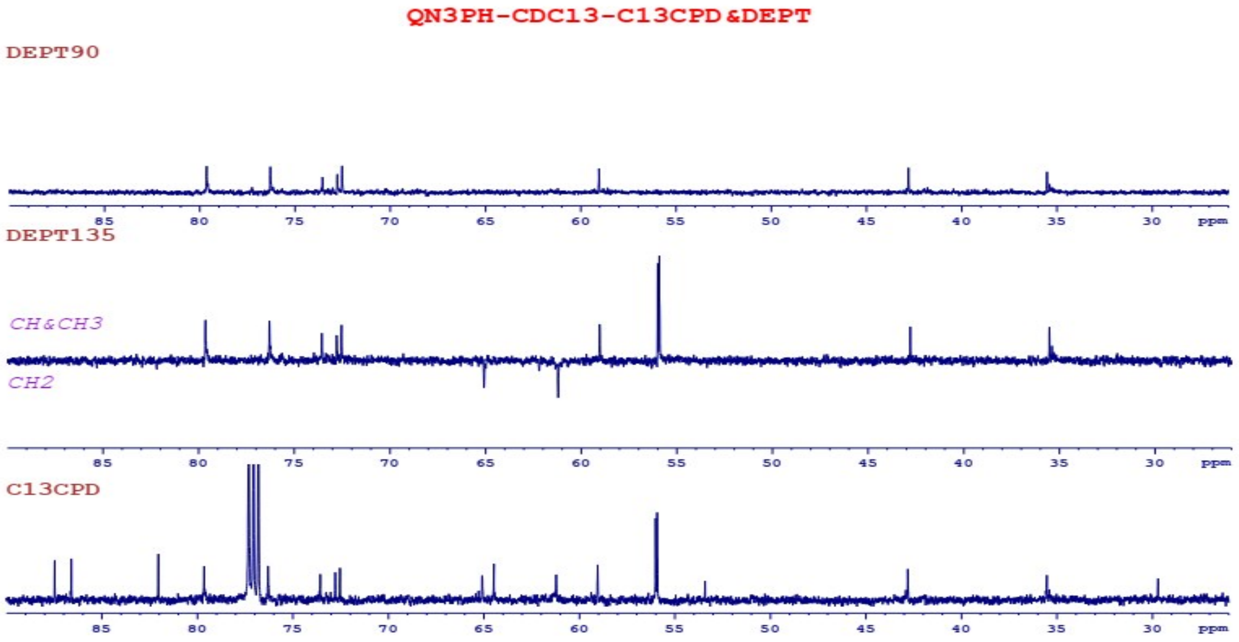


DEPT spectrum (epansion) of compound **6**

## 1.7. Compound 7

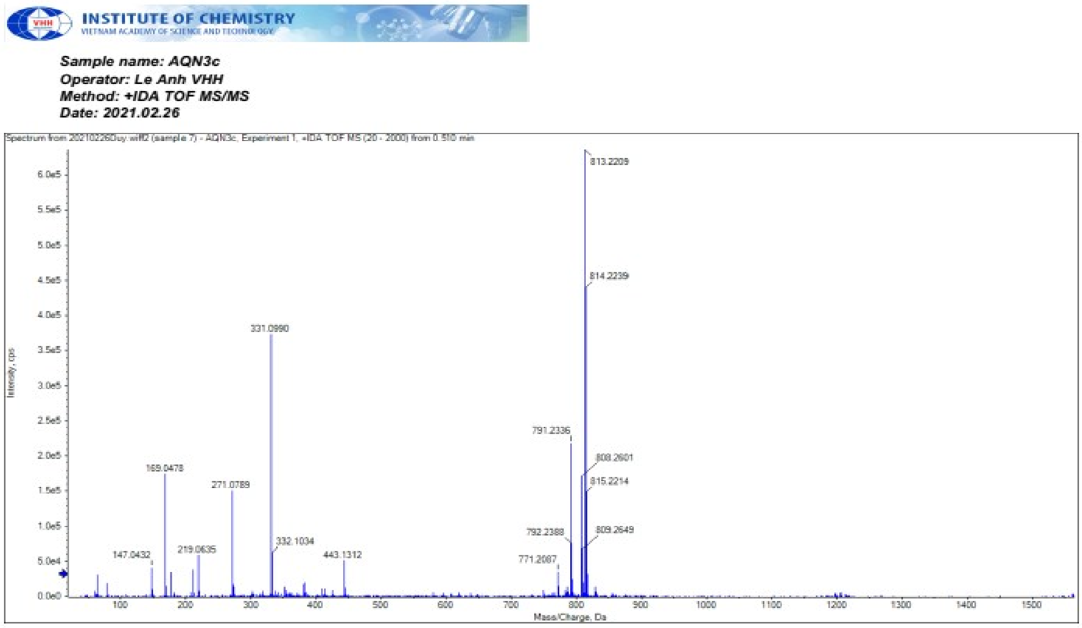


| Hit | Formula | m/z | RDB | ppm | MS Rank | MSMS ppm | MSMS Rank | Found |
|---|---|---|---|---|---|---|---|---|
| 1 | C37H42O19 | 813.22125 | 17.0 | -1.7 | 1 | | | NA/NA |

*Device Model:* SCIEX X500 QTOF

HR-ESI-MS spectrum of compound **7**

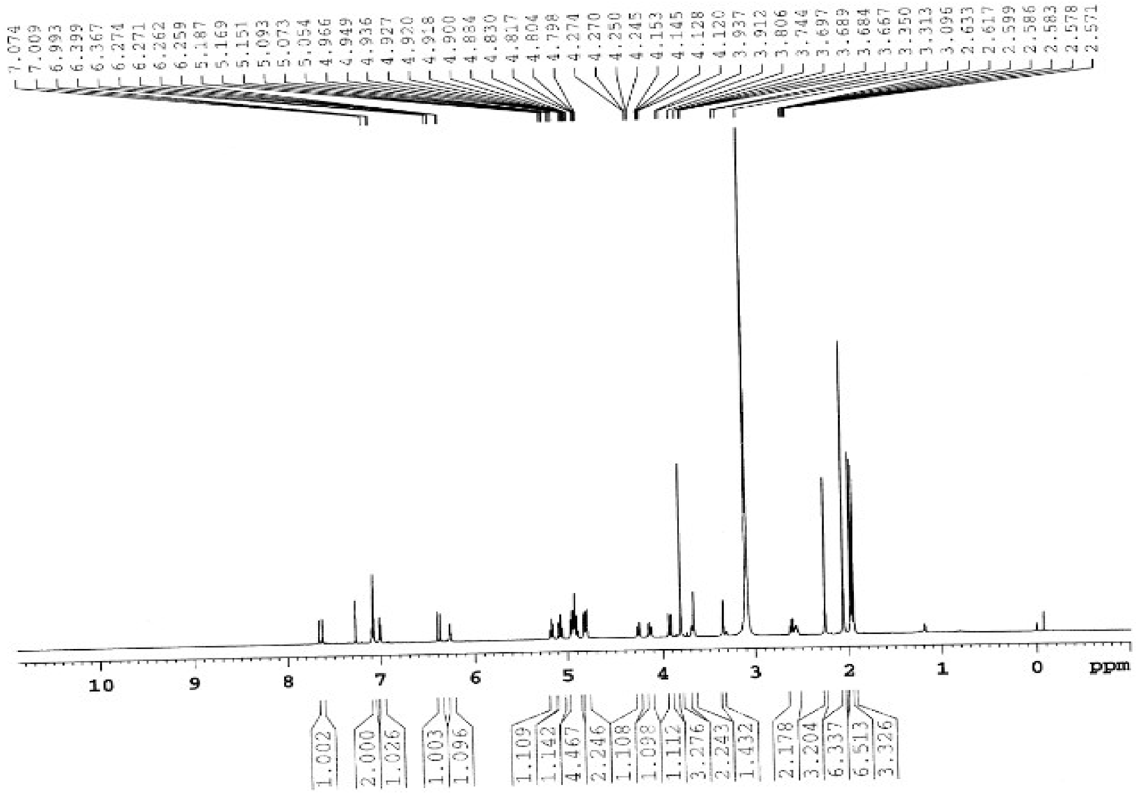


$^{1}$H-NMR spectrum (500 MHz, $CDCl_3$) of compound **7**

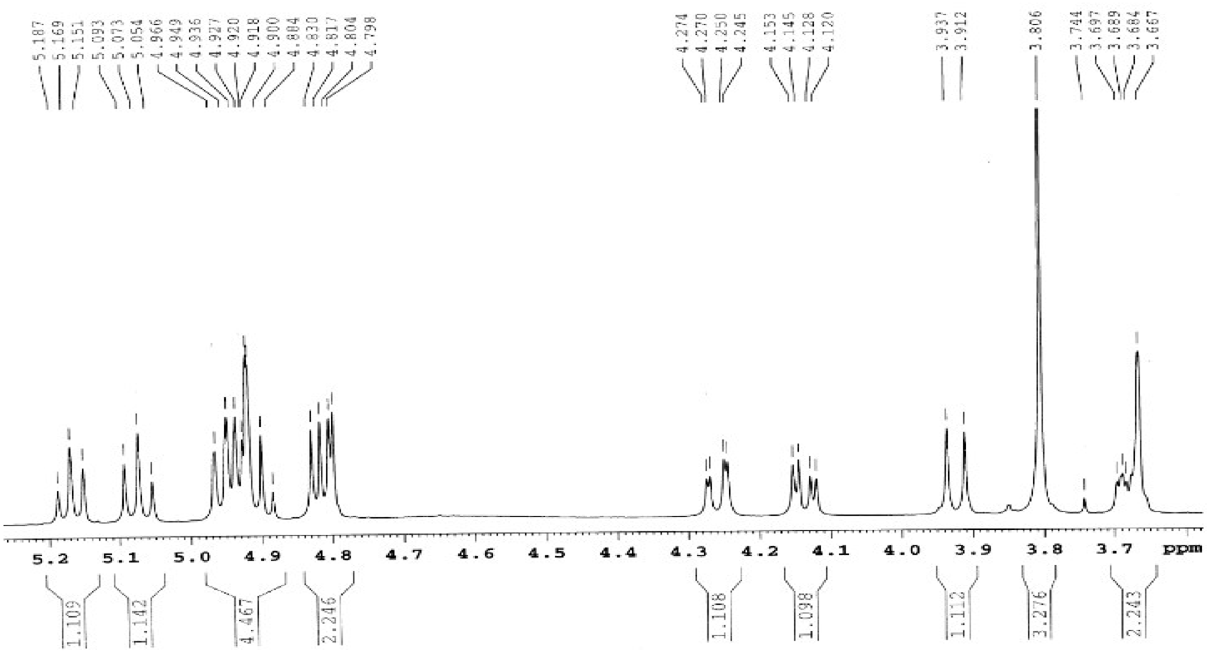


$^{1}$H-NMR spectrum (expansion) of compound **7**

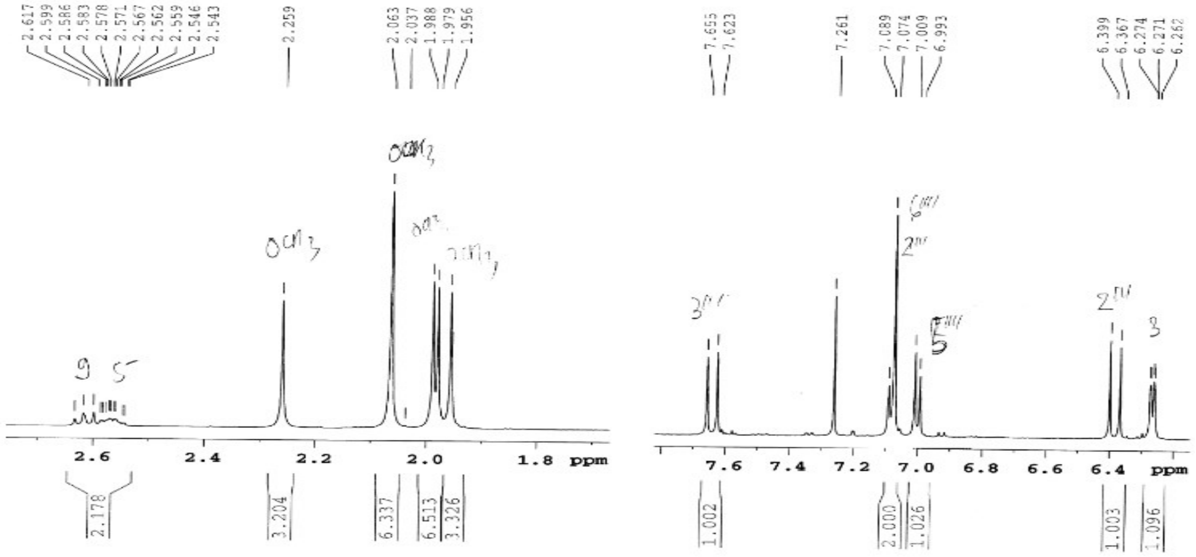


$^{1}$H-NMR spectrum (expansion) of compound **7**

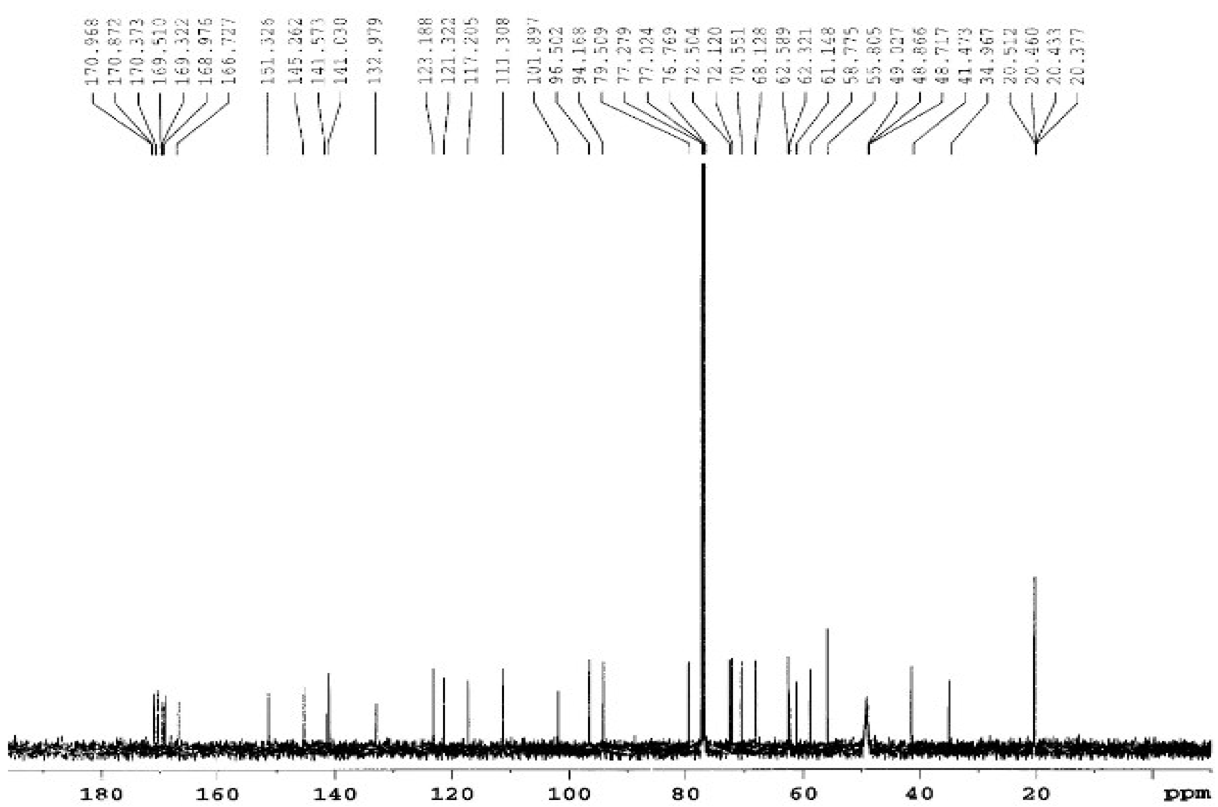


$^{13}$C-NMR spectrum of compound **7**

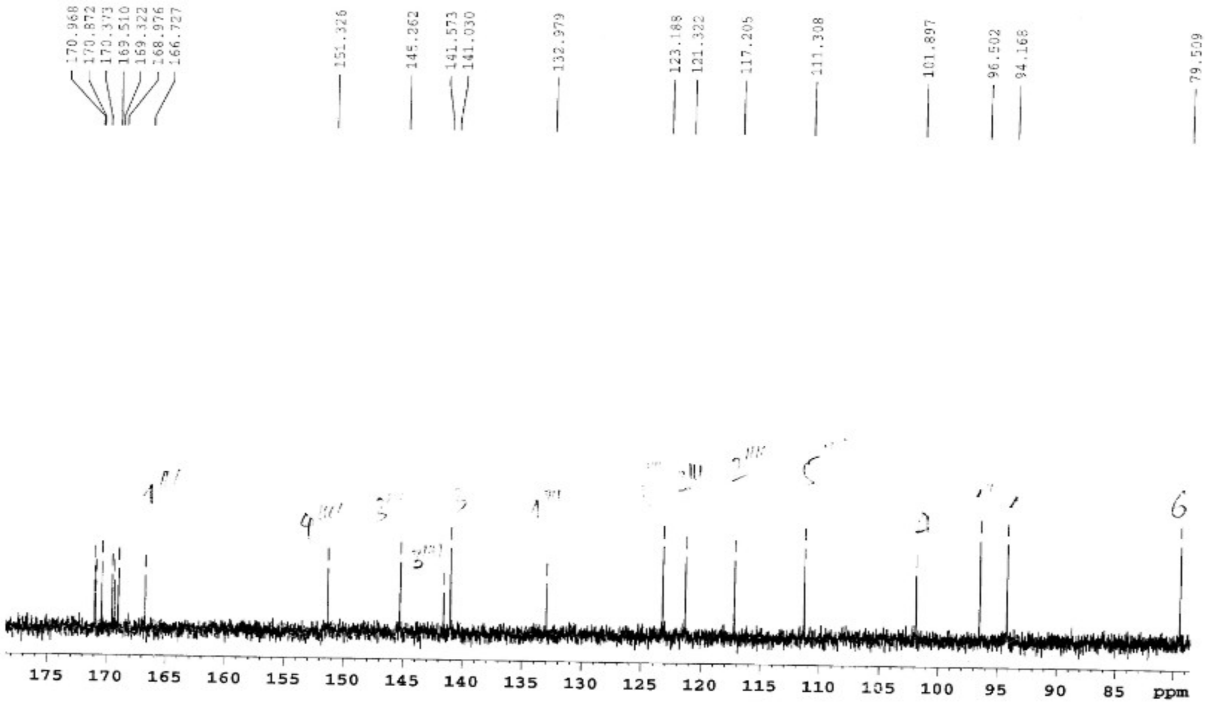


$^{13}$C-NMR spectrum (expansion) of compound **7**

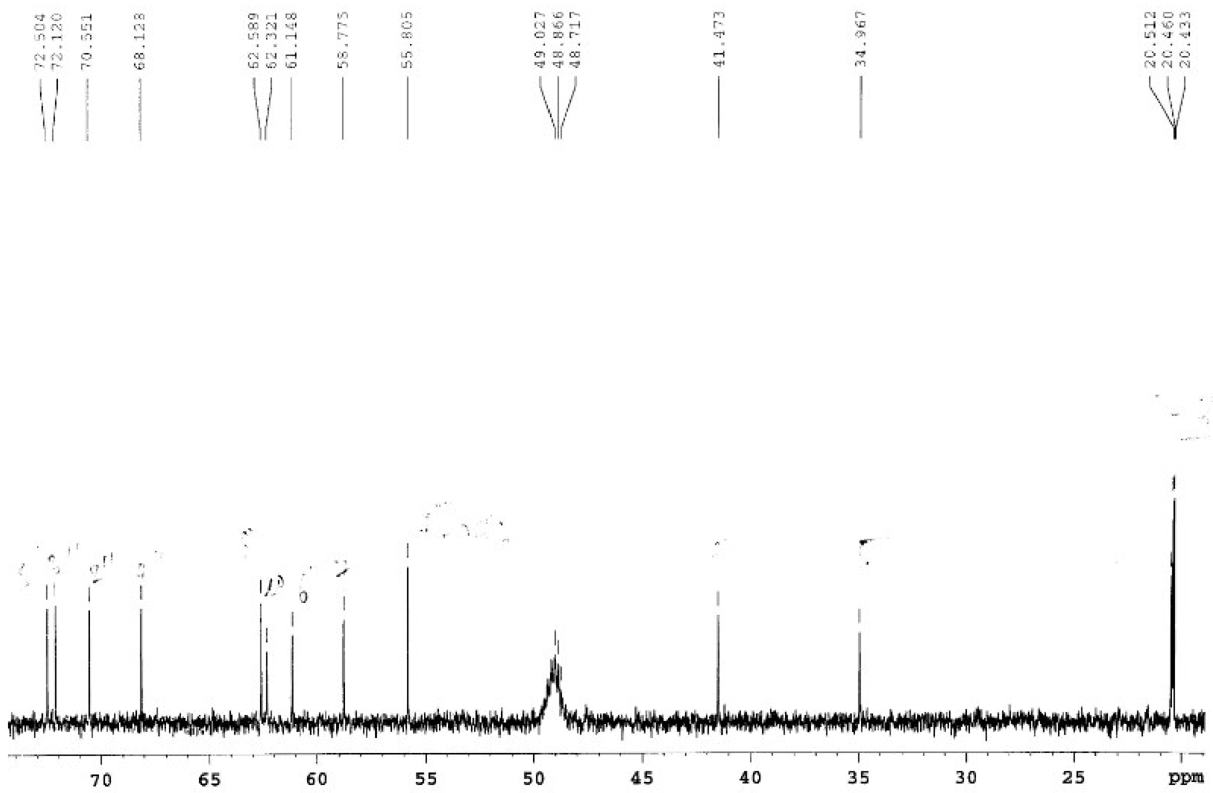


$^{13}$C-NMR spectrum (expansion) of compound **7**

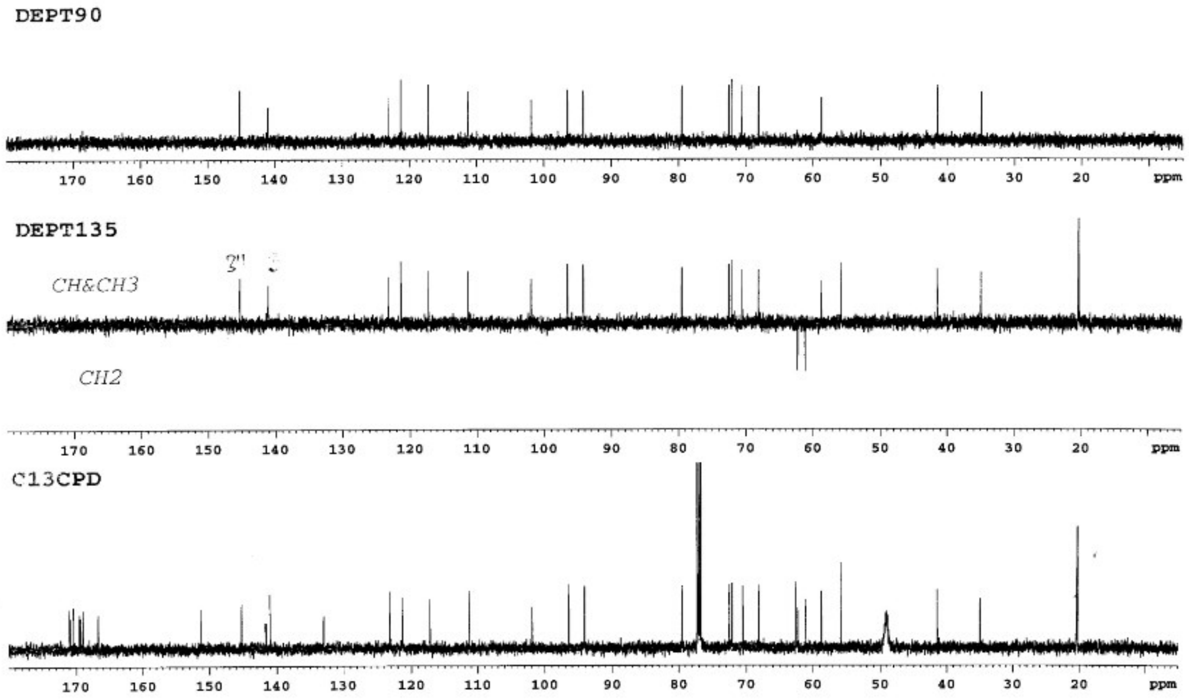


DEPT spectrum of compound **7**

### 1.8. Compound 8

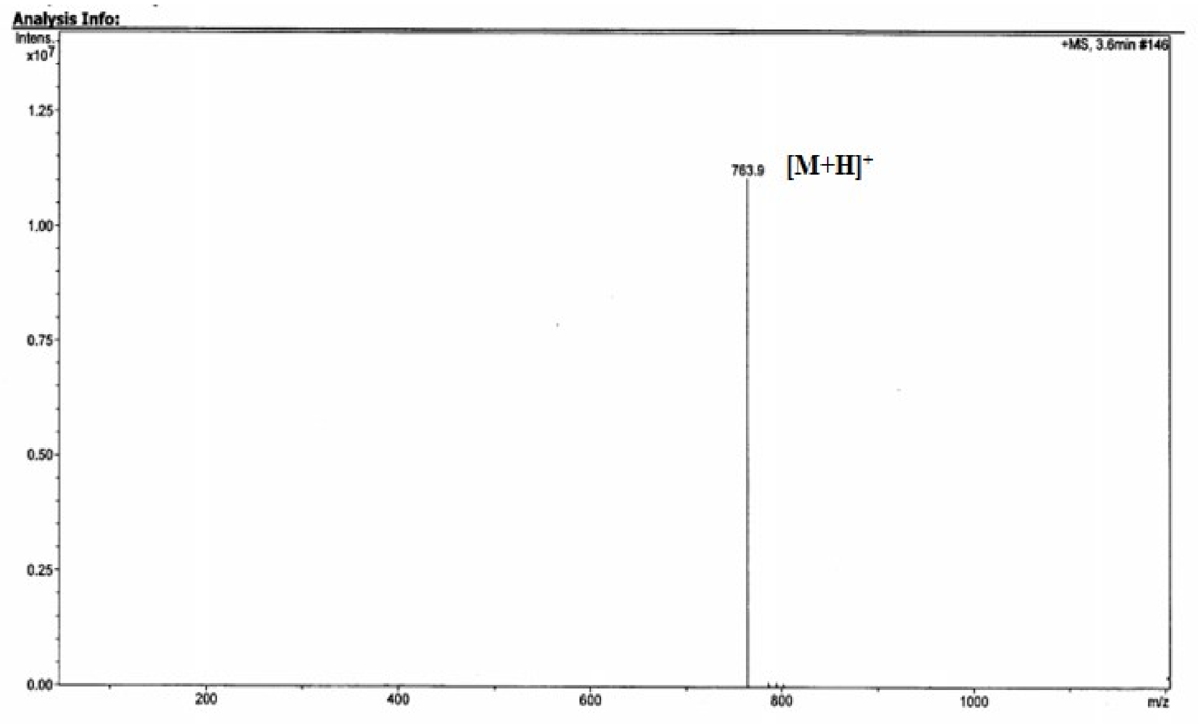


(+)-ESI-MS spectrum of compound **8**

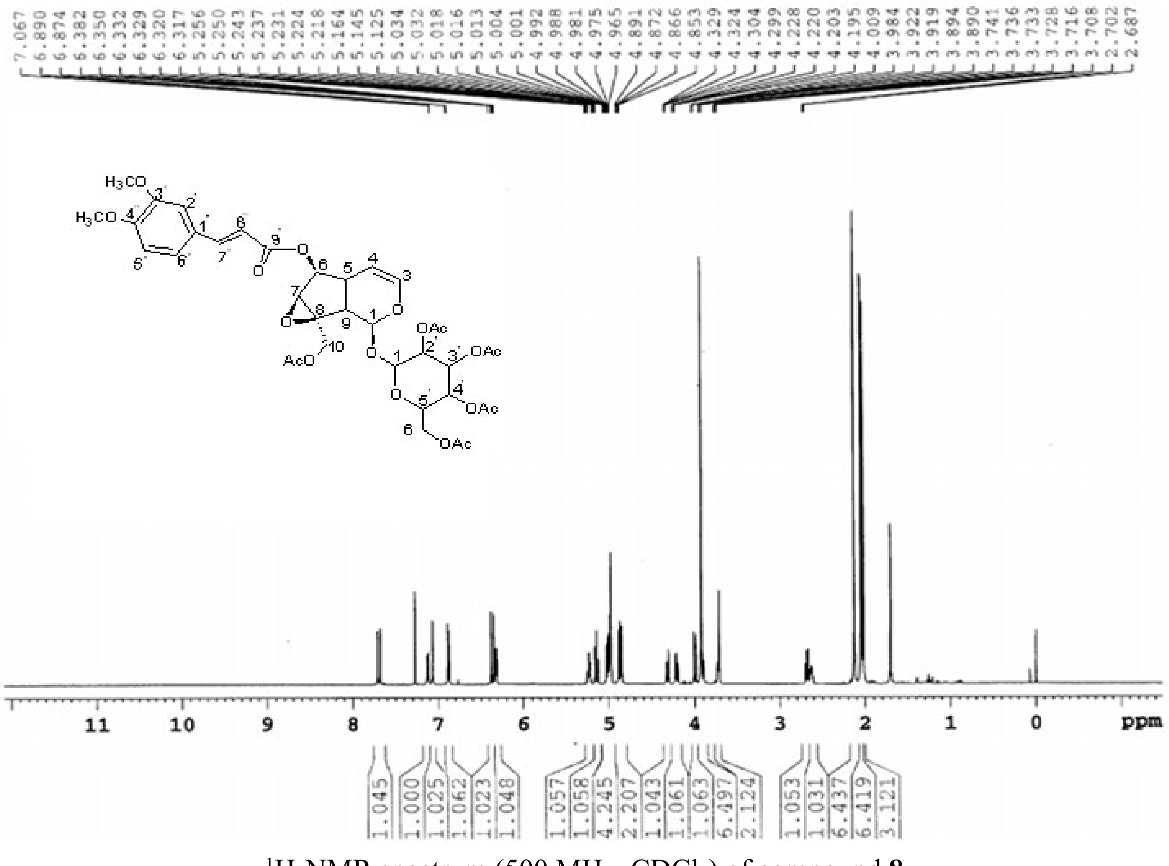

$^{1}$H-NMR spectrum (500 MHz, $CDCl_3$) of compound **8**

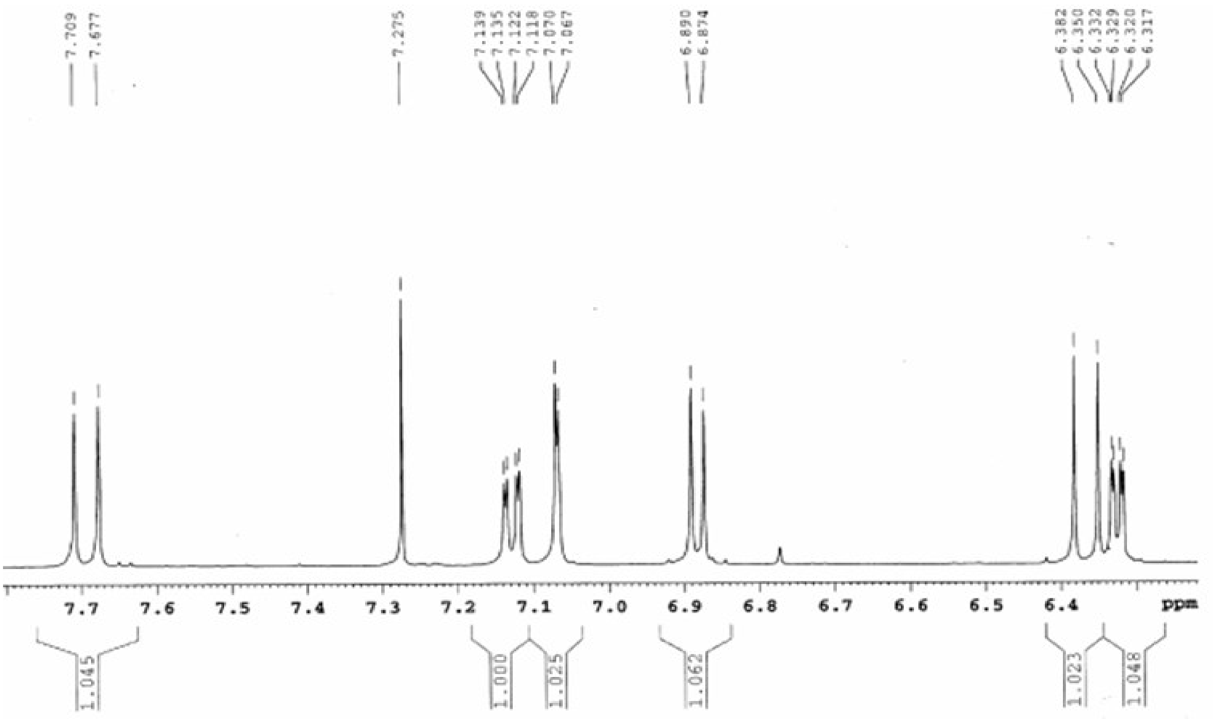


$^1$H-NMR spectrum (expansion) of compound **8**

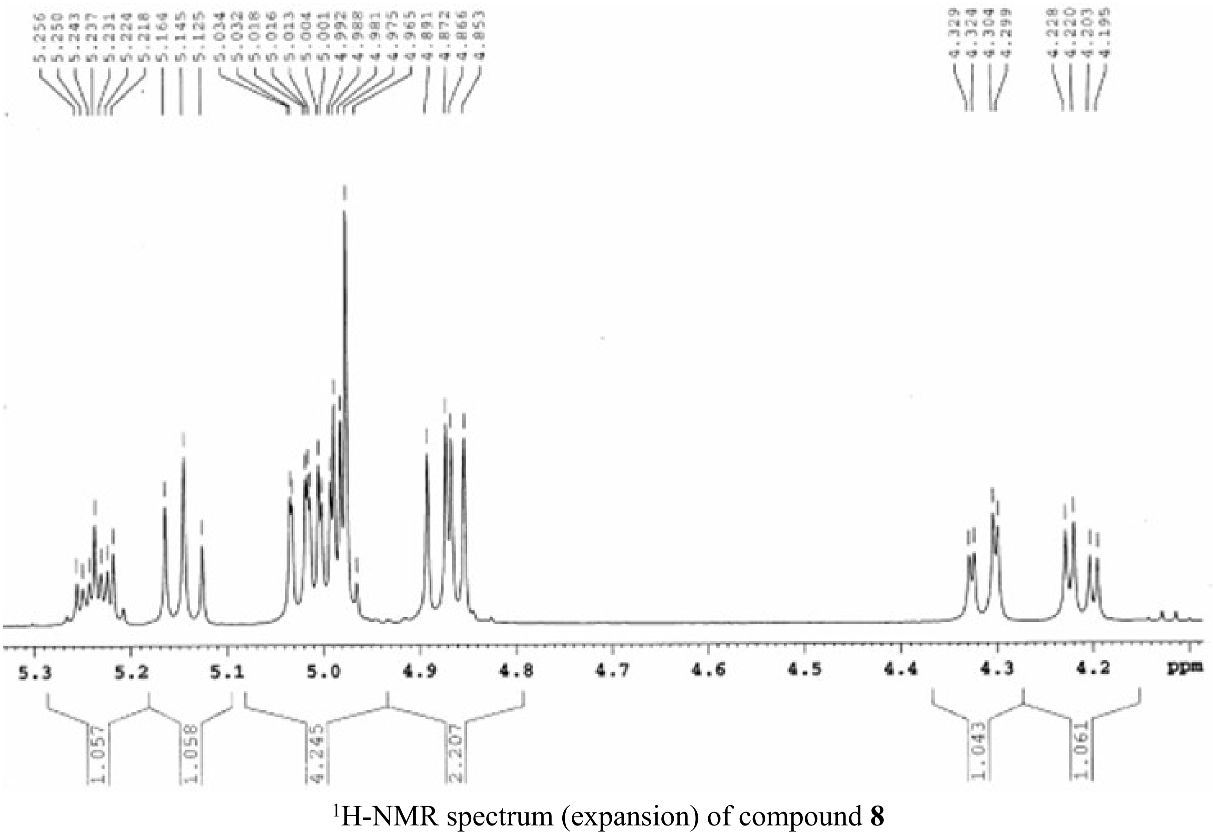


$^1$H-NMR spectrum (expansion) of compound **8**

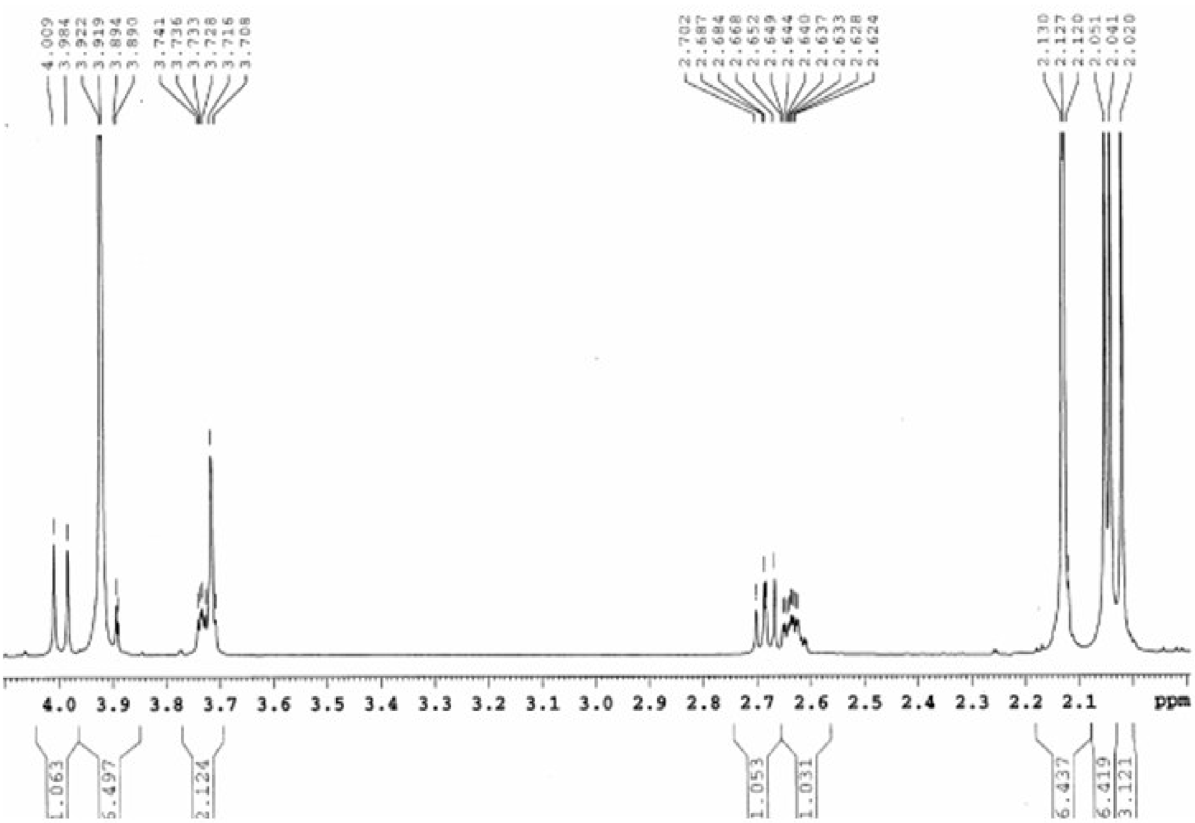


$^{1}$H-NMR spectrum (expansion) of compound **8**

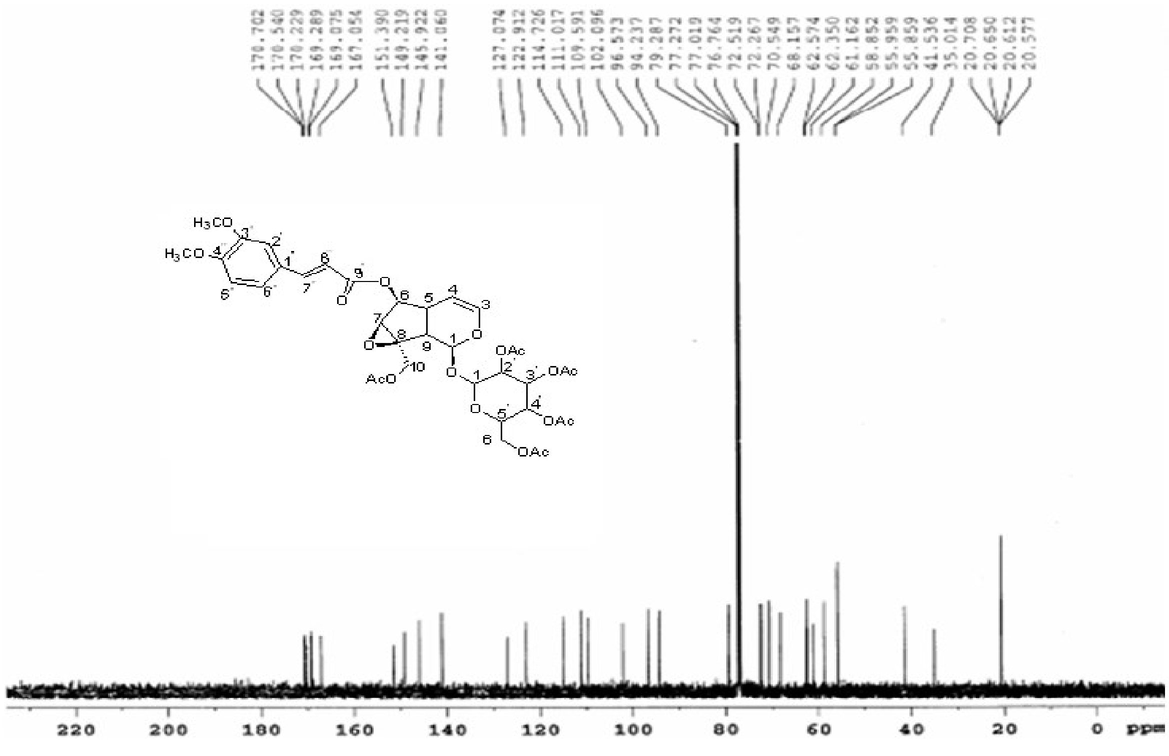


$^{13}$C-NMR spectrum of compound **8**

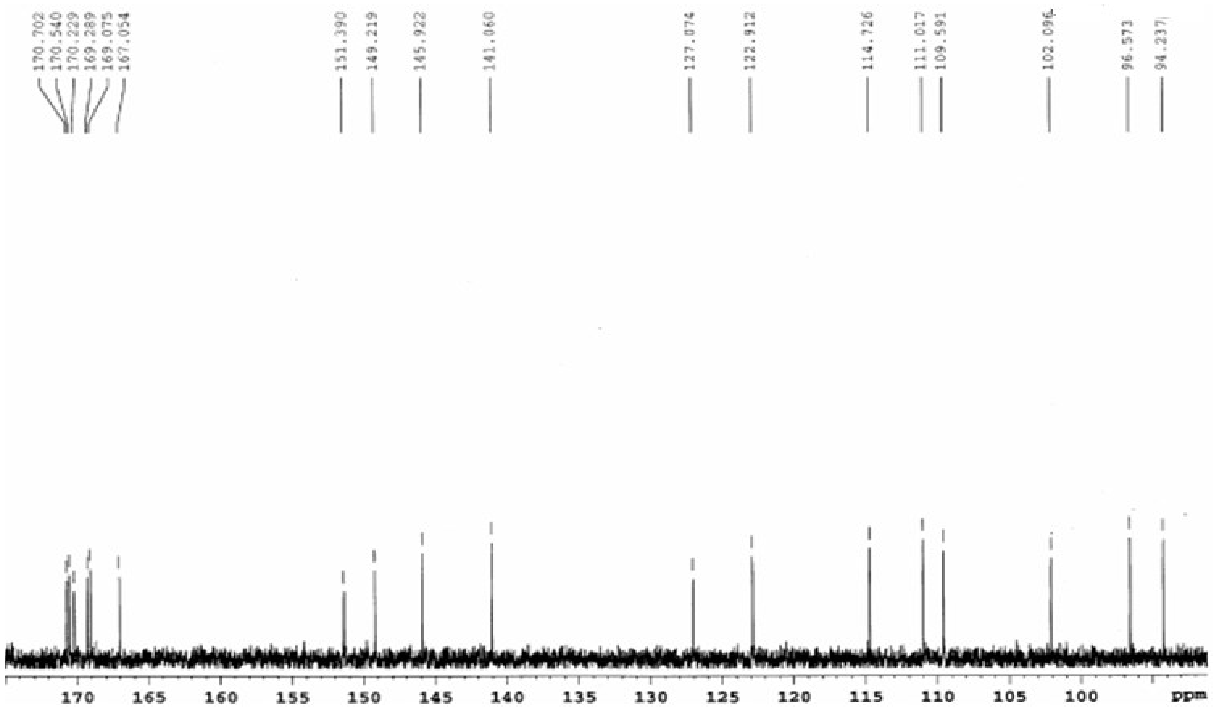

$^{13}$C-NMR spectrum (expansion) of compound **8**

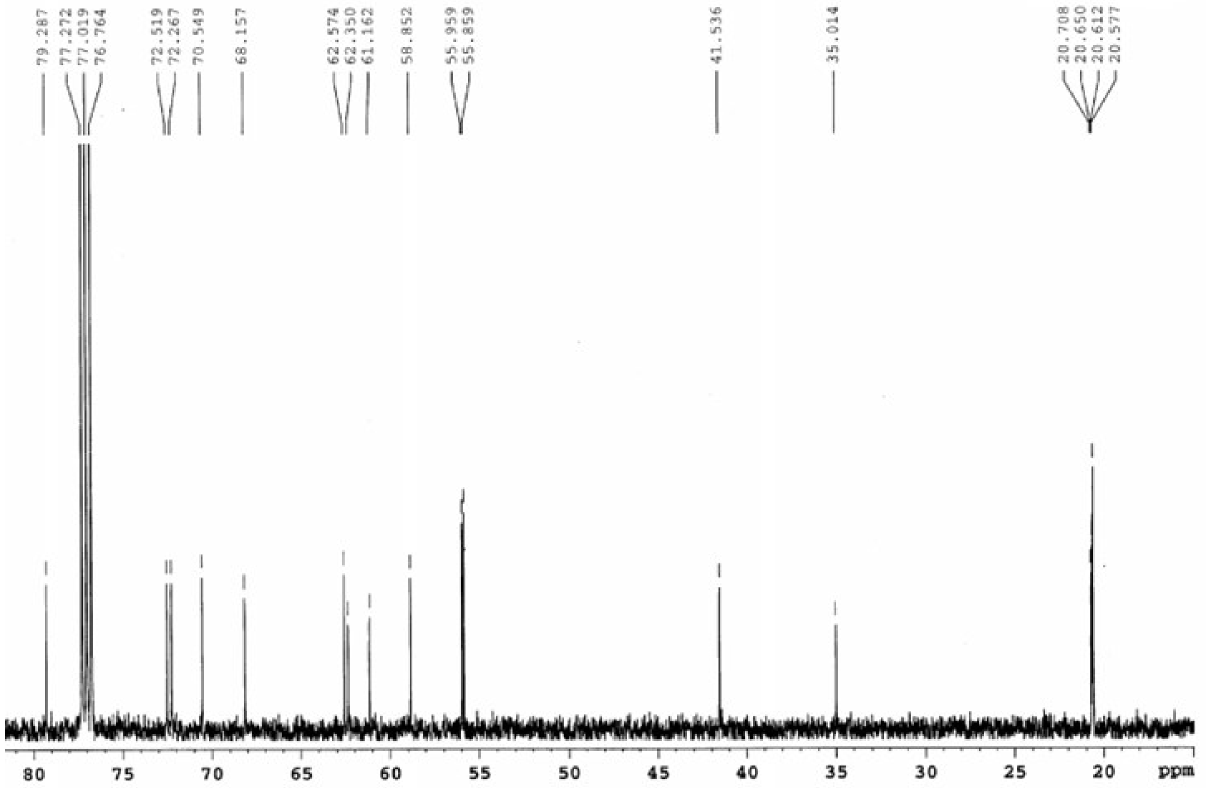

$^{13}$C-NMR spectrum (expansion) of compound **8**

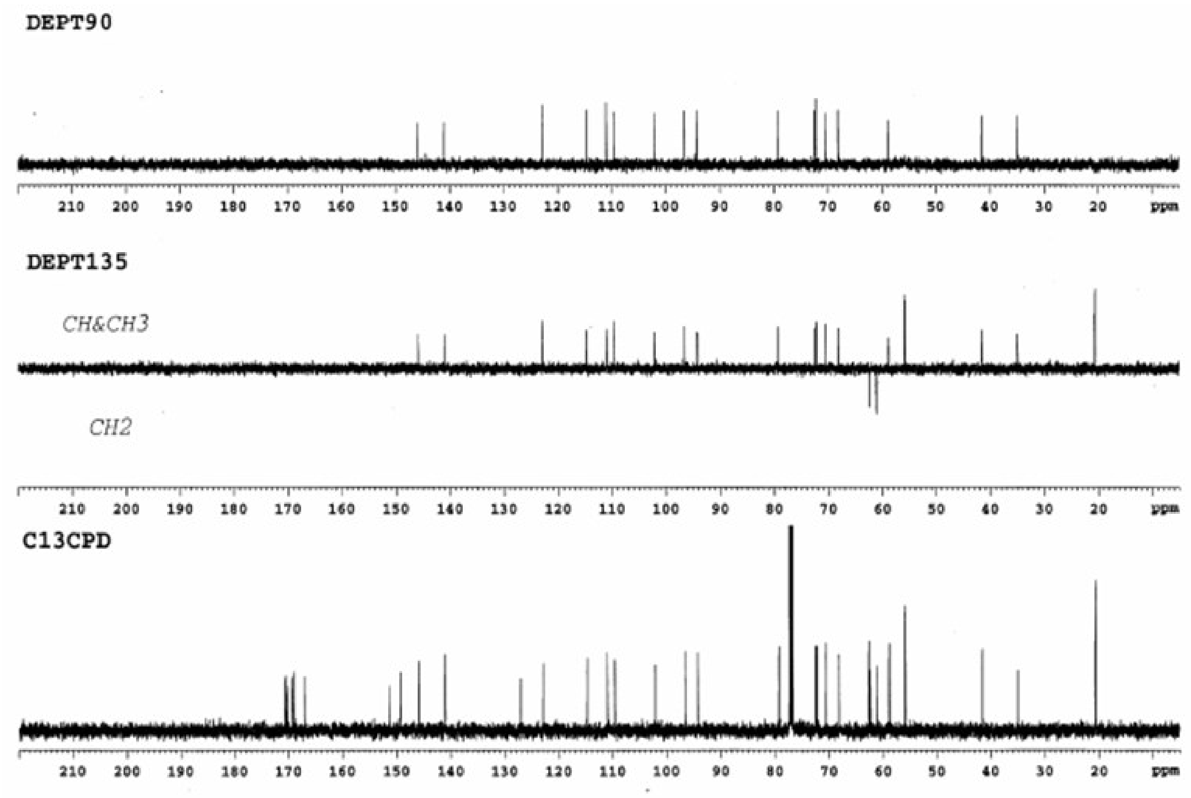


DEPT spectrum of compound **8**

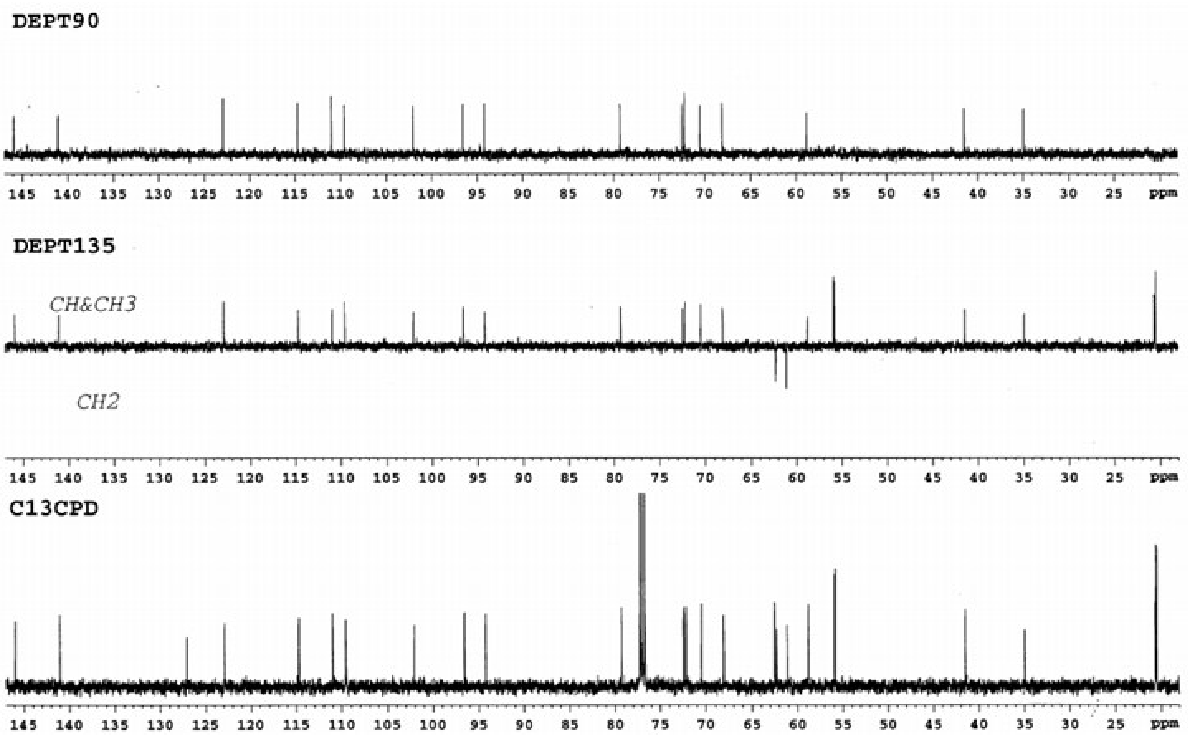


DEPT spectrum (expansion) of compound **8**

## 1.9. Compound 9

INSTITUTE OF CHEMISTRY
VIETNAM ACADEMY OF SCIENCE AND TECHNOLOGY

*Sample name: QN1aceto2*
*Operator: Le Anh VHH*
*Method: +IDA TOF MS/MS*
*Date: 2021.02.26*

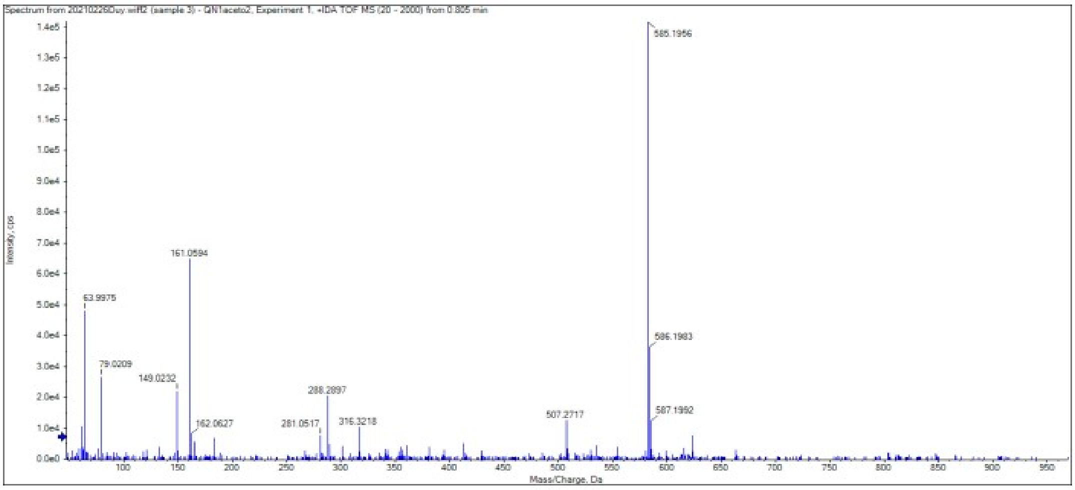


| Hit | Formula | m/z | RDB | ppm | MS Rank | MSMS ppm | MSMS Rank | Found |
|---|---|---|---|---|---|---|---|---|
| 1 | C28H34O12 | 585.19425 | 12.0 | 2.3 | 1 | | | NA/NA |

*Device Model:* SCIEX X500 QTOF

HR-ESI-MS spectrum of compound **9**

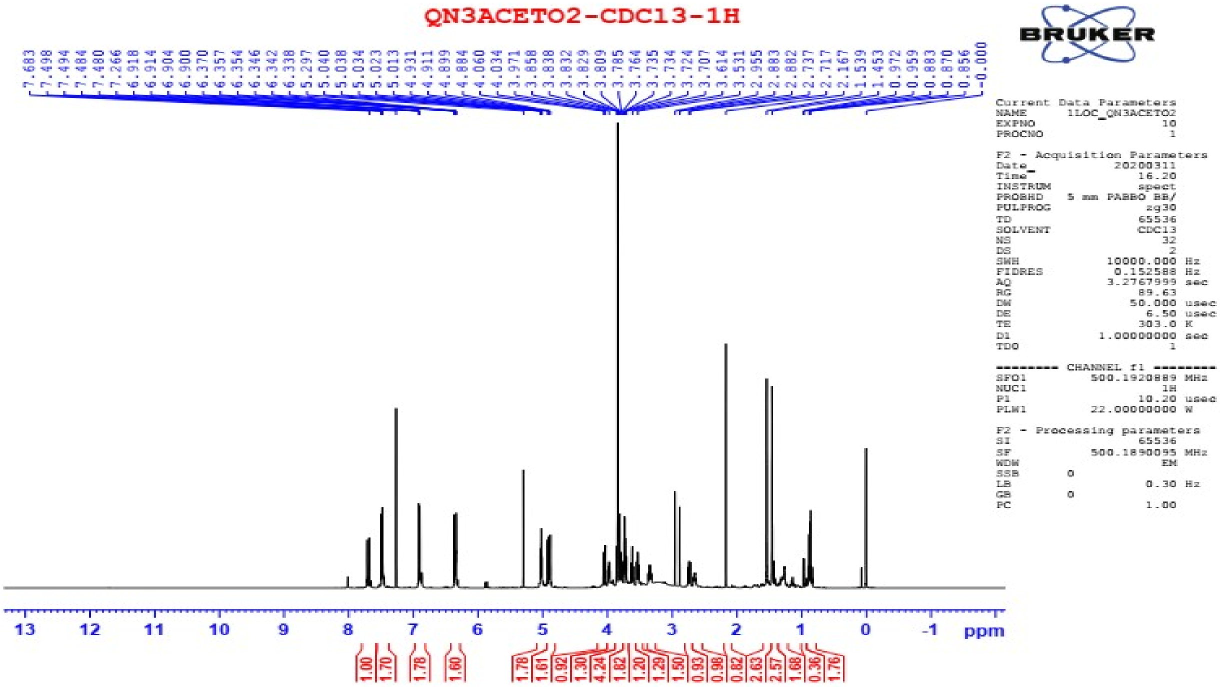


$^{1}$H-NMR spectrum of compound **9**

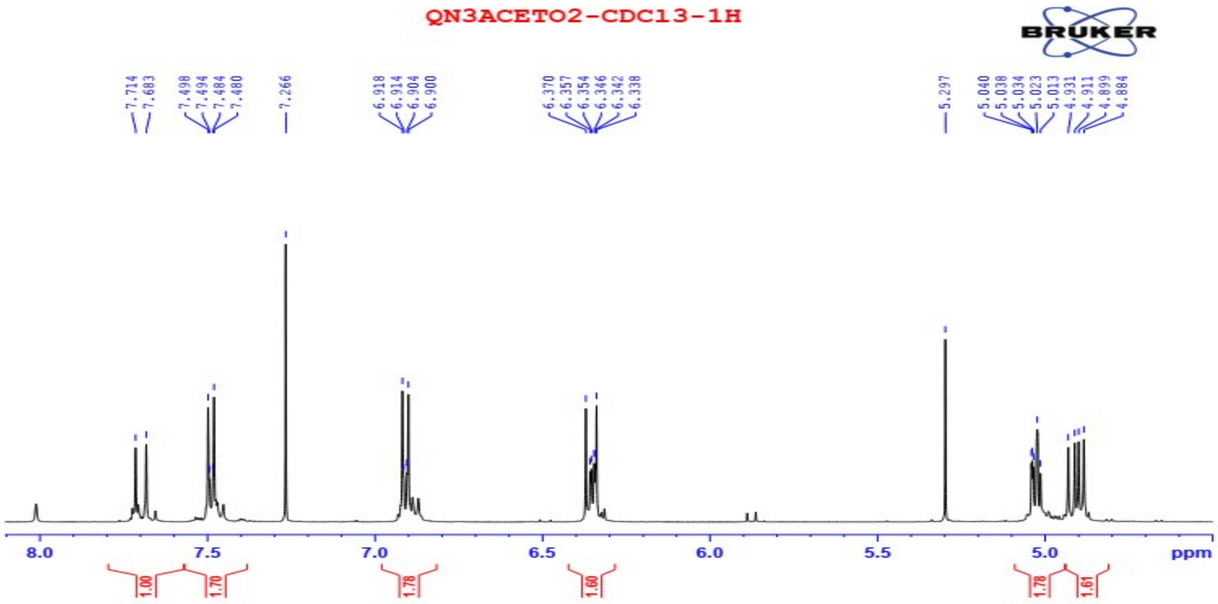


$^{1}$H-NMR spectrum (expansion) of compound **9**

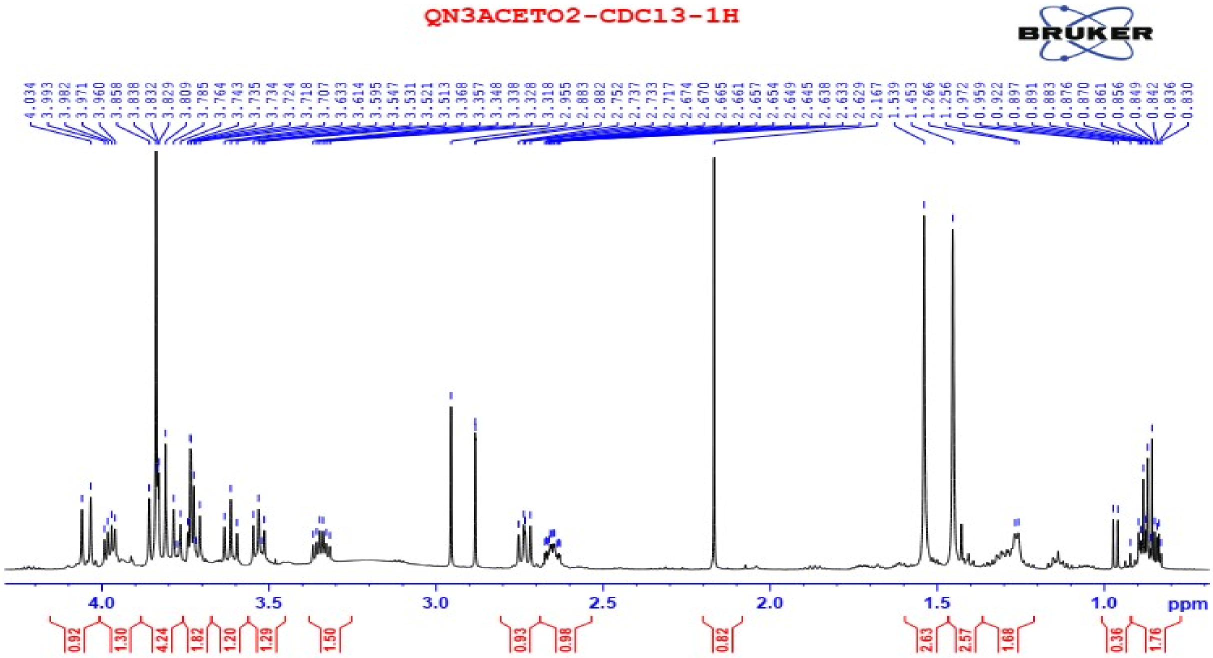


$^{1}$H-NMR spectrum (expansion) of compound **9**

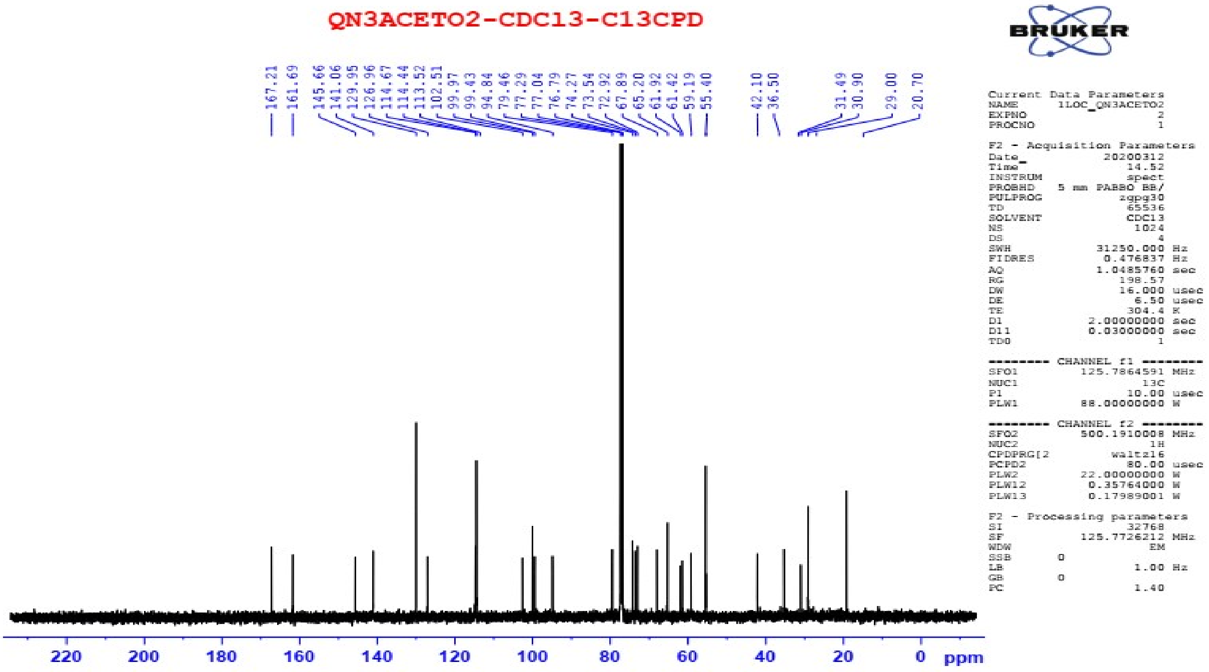


$^{13}$C-NMR spectrum of compound **9**

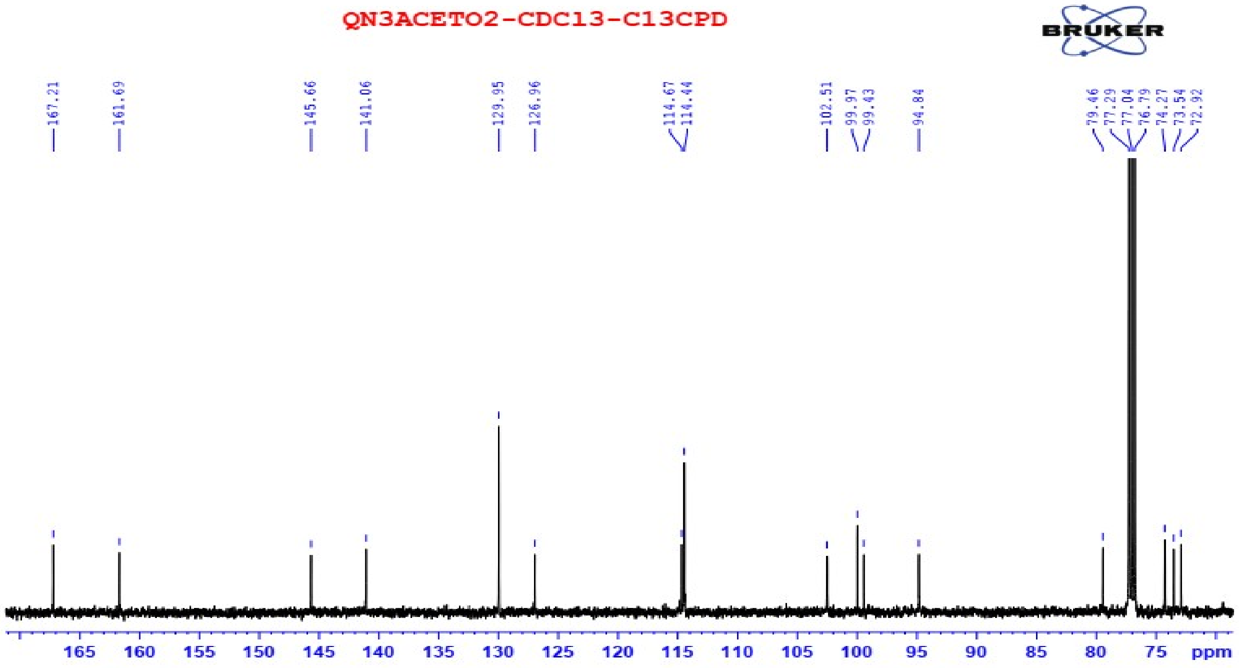


$^{13}$C-NMR spectrum (expansion) of compound **9**

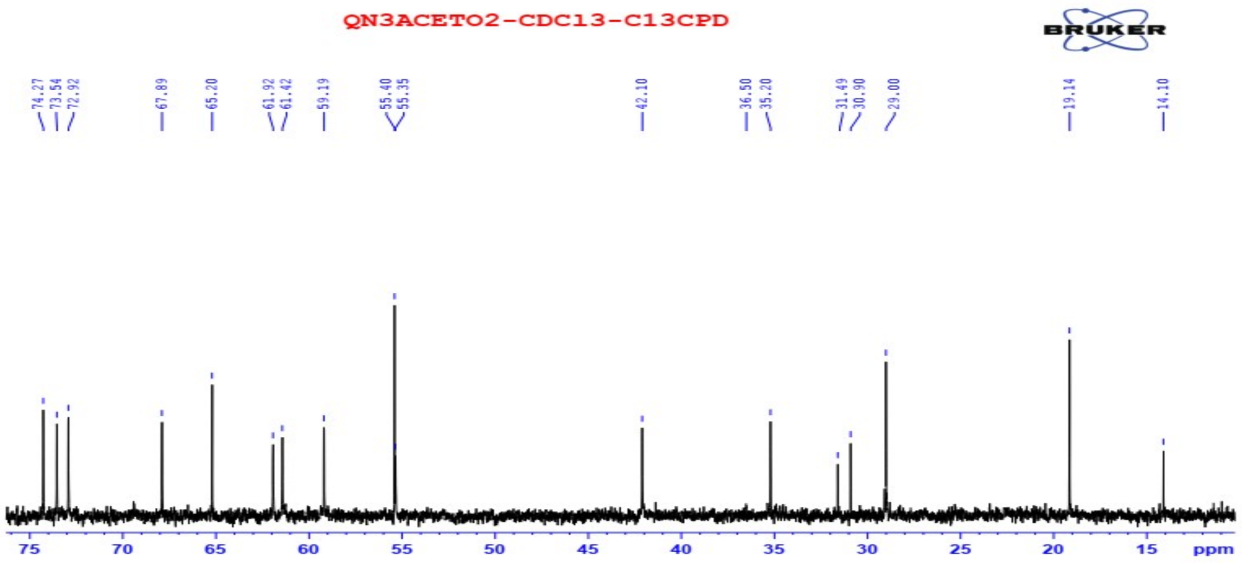


$^{13}$C-NMR spectrum (expansion) of compound **9**

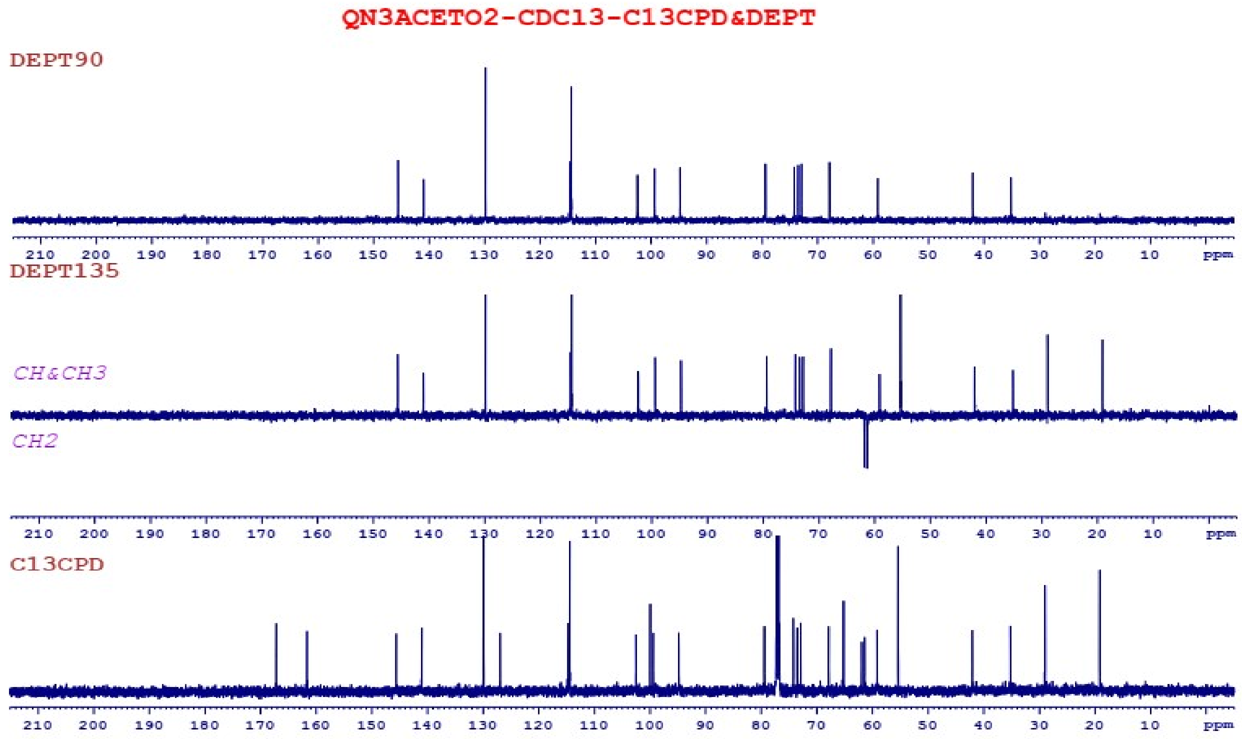


DEPT spectrum of compound **9**

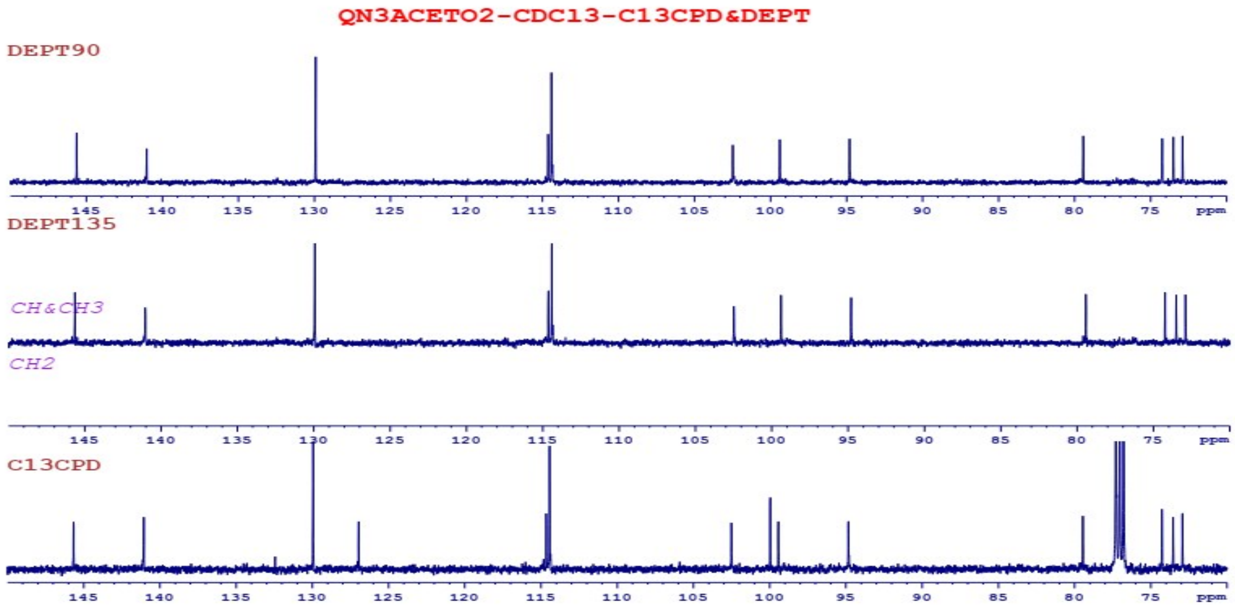


DEPT spectrum (epansion) of compound **9**

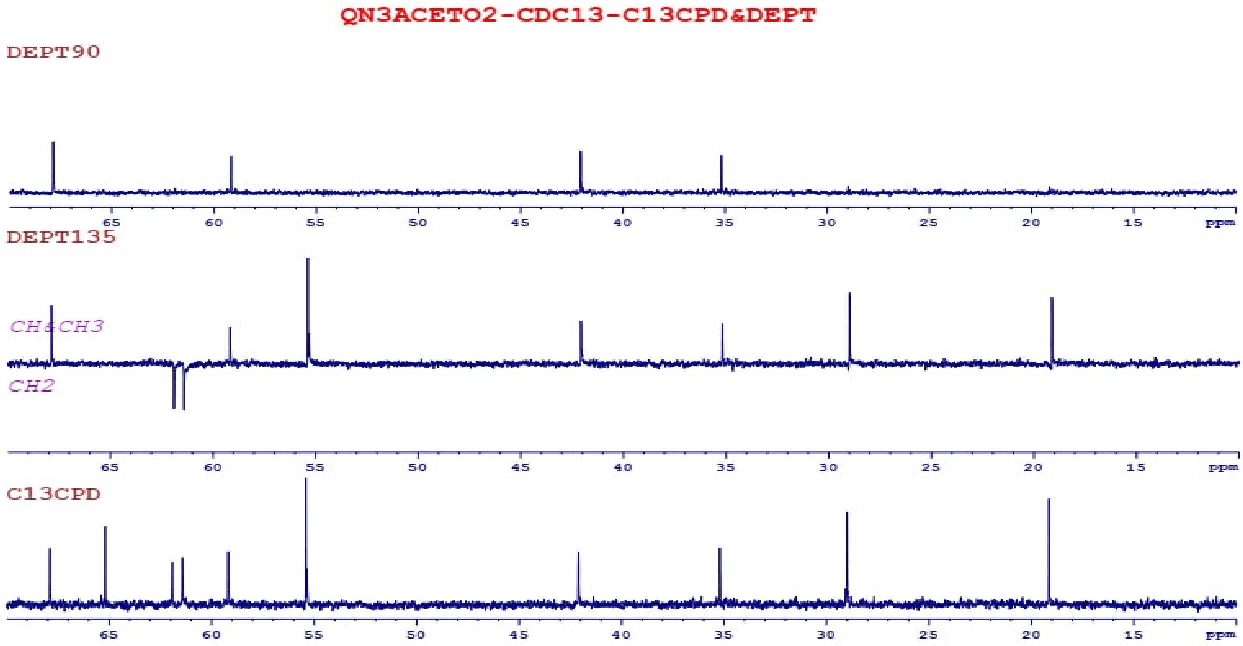


DEPT spectrum (epansion) of compound **9**

### 1.10. Compound 10

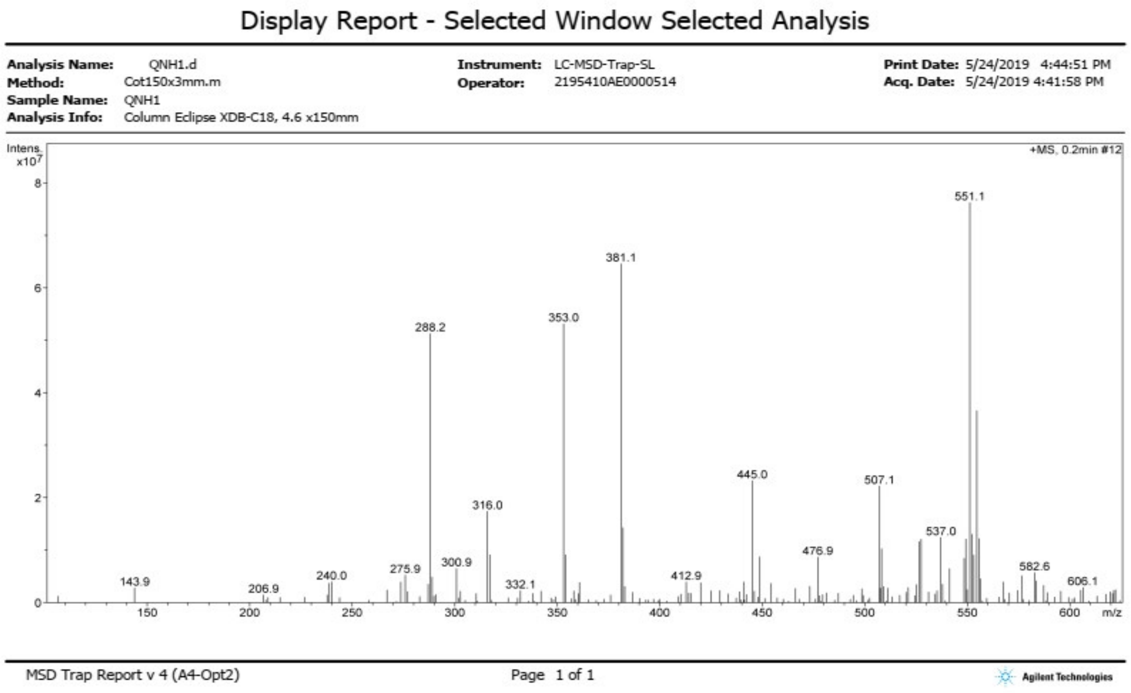


(+)-ESI-MS spectrum of compound **10**

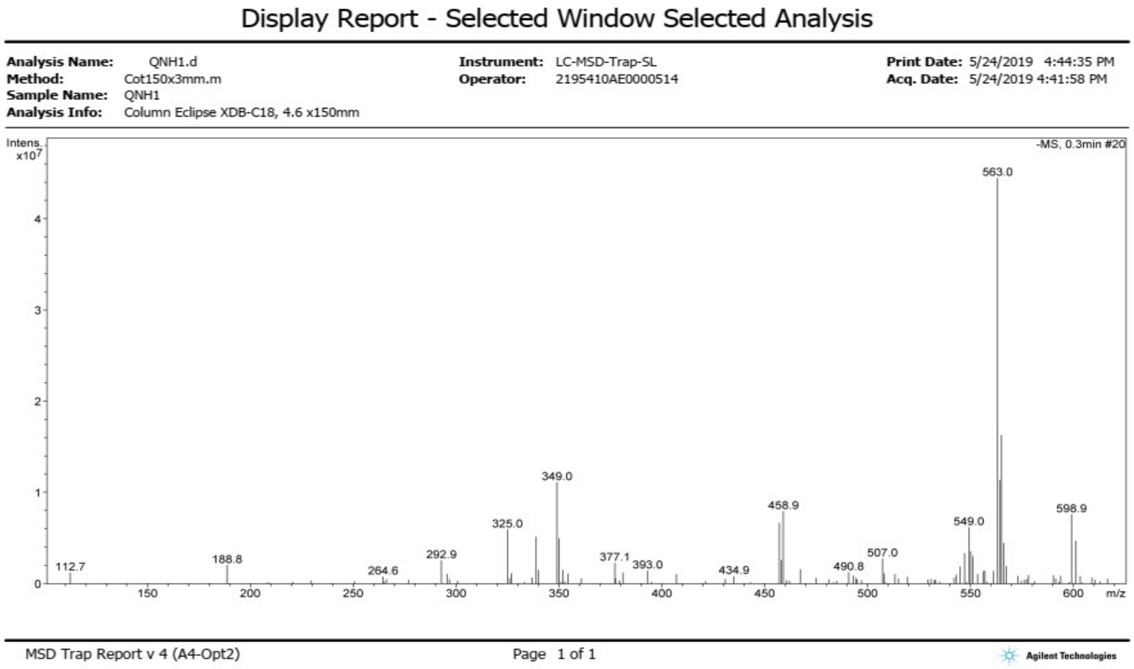


(-)-ESI-MS spectrum of compound **10**

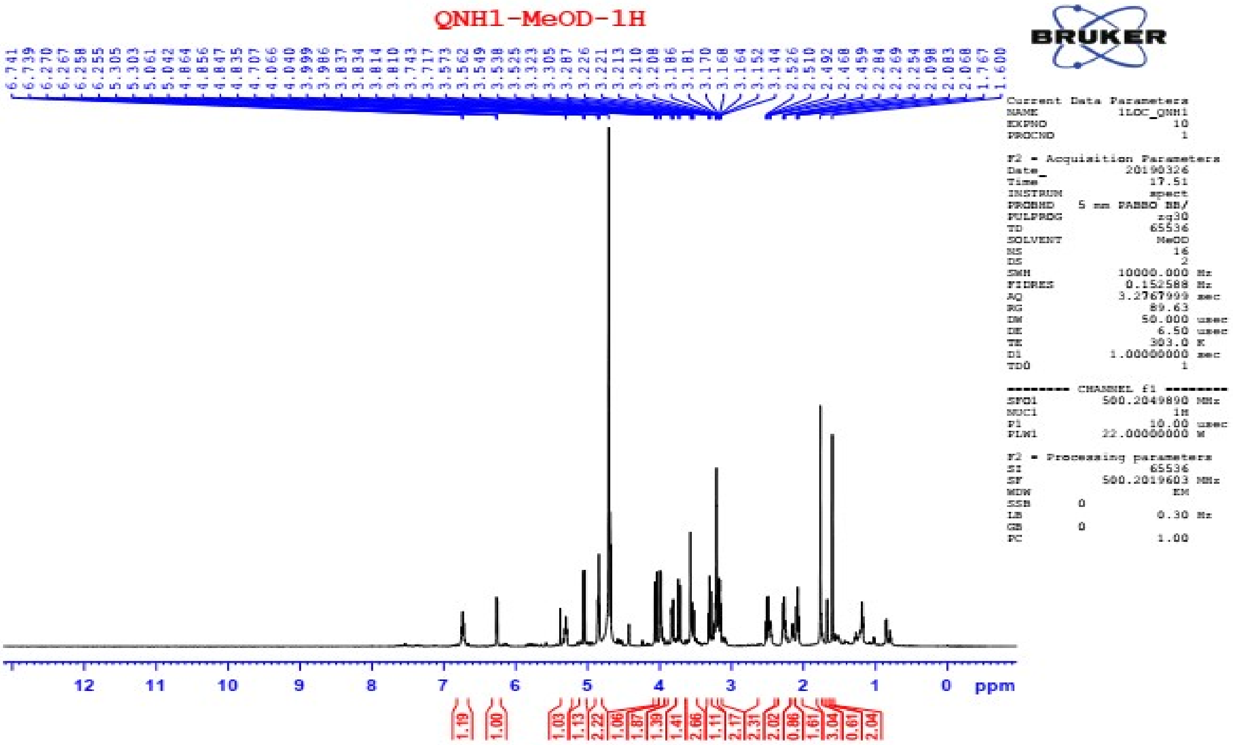

$^{1}$H-NMR spectrum of compound **10**

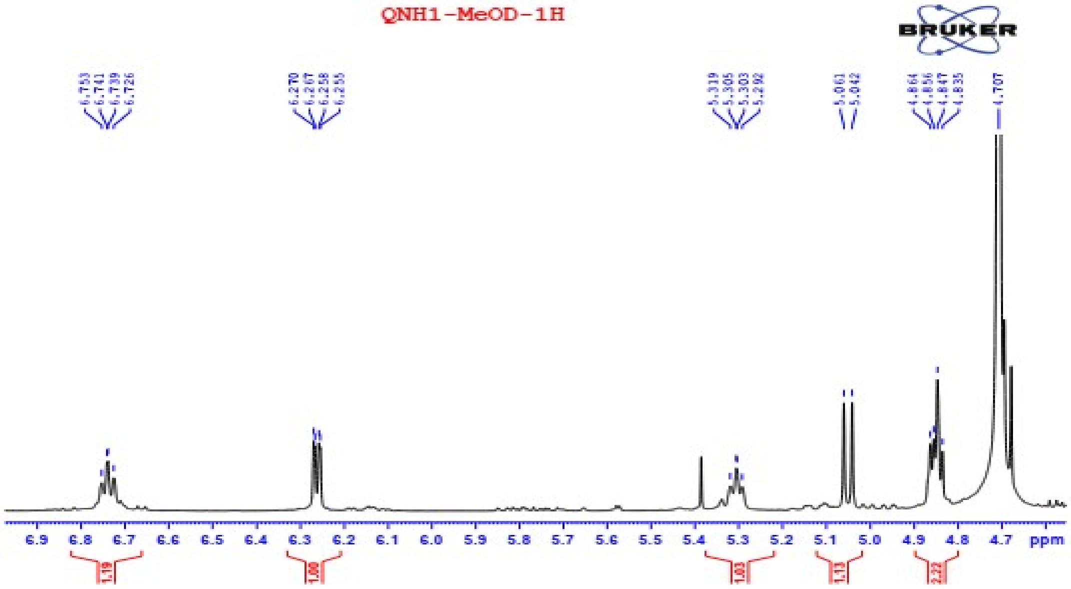

$^{1}$H-NMR spectrum (expansion) of compound **10**

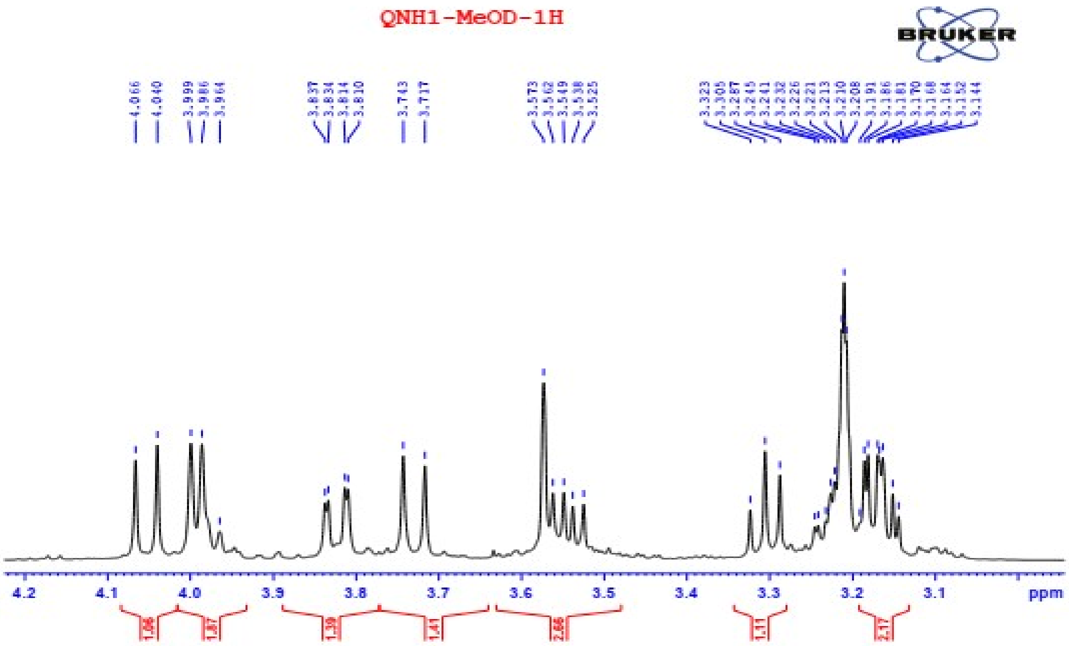

$^{1}$H-NMR spectrum (expansion) of compound **10**

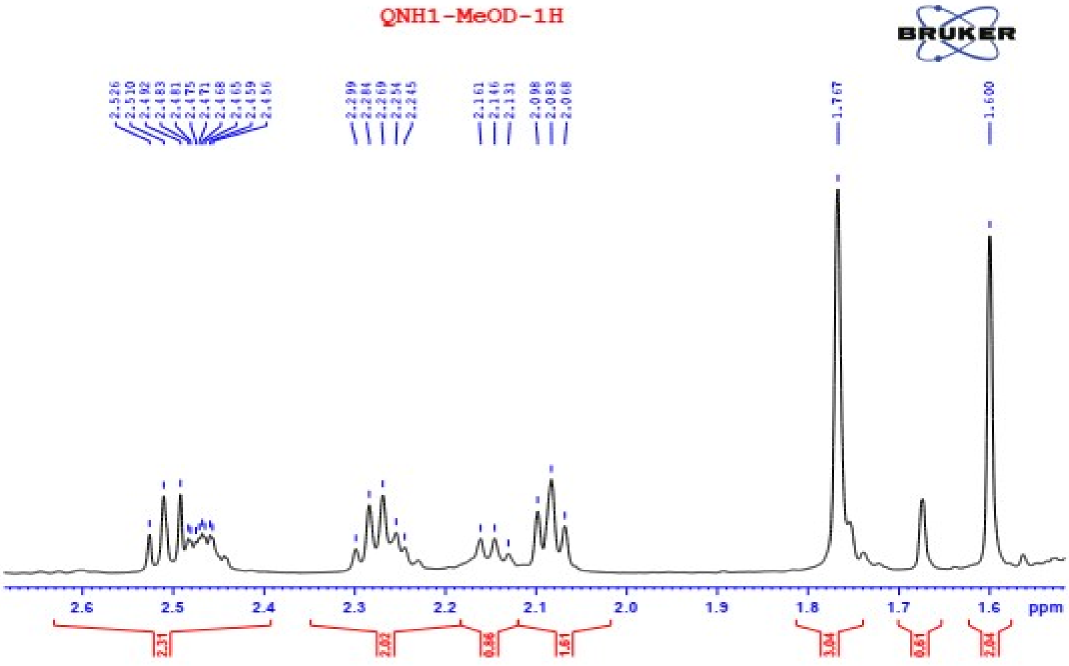

$^{1}$H-NMR spectrum (expansion) of compound **10**

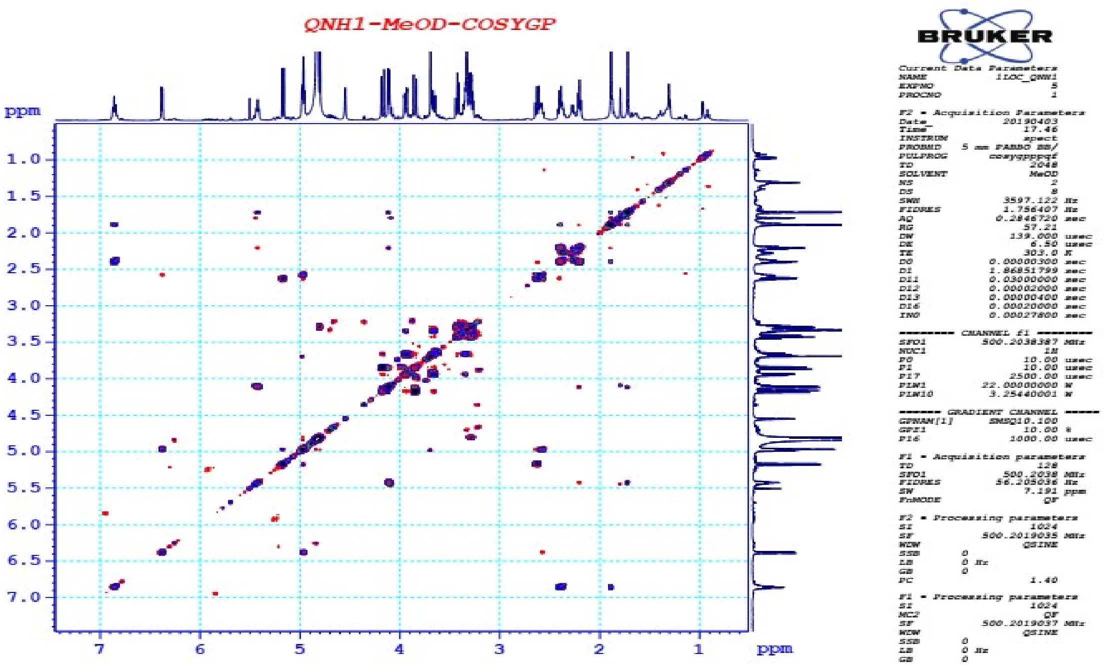


COSY spectrum of compound **10**

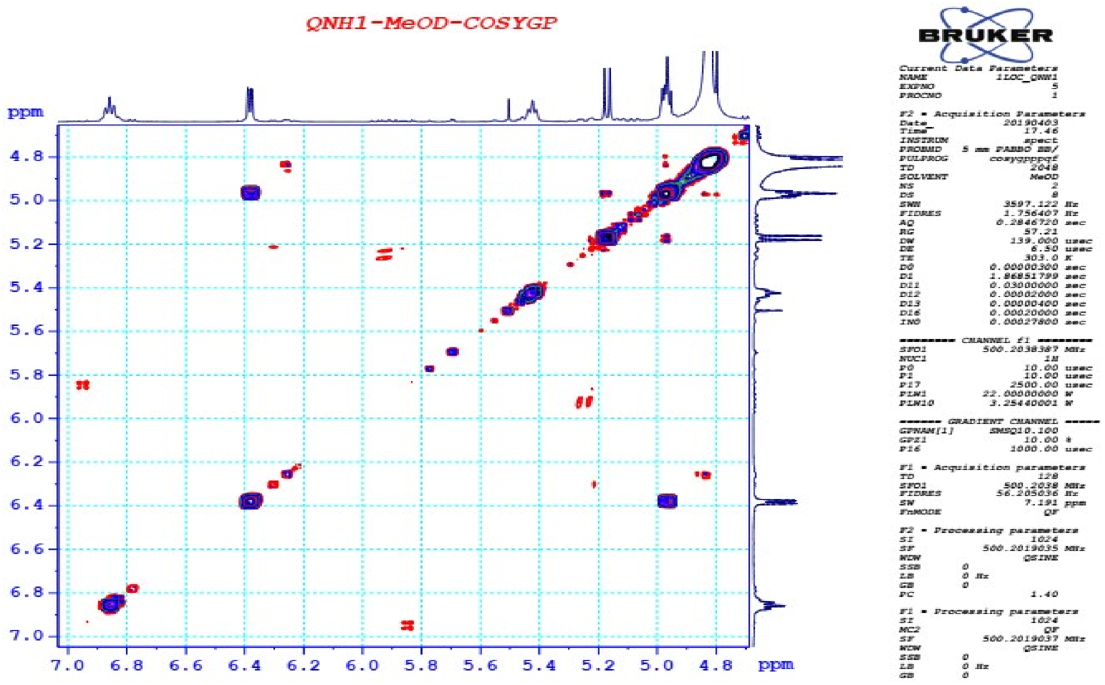


COSY spectrum (expansion) of compound **10**

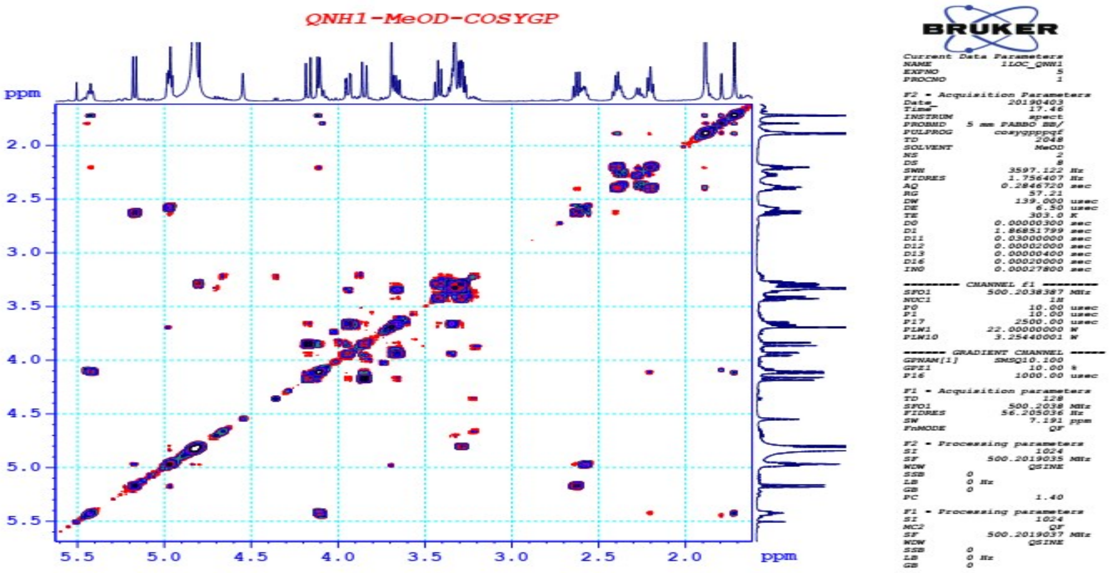


COSY spectrum (expansion) of compound **10**

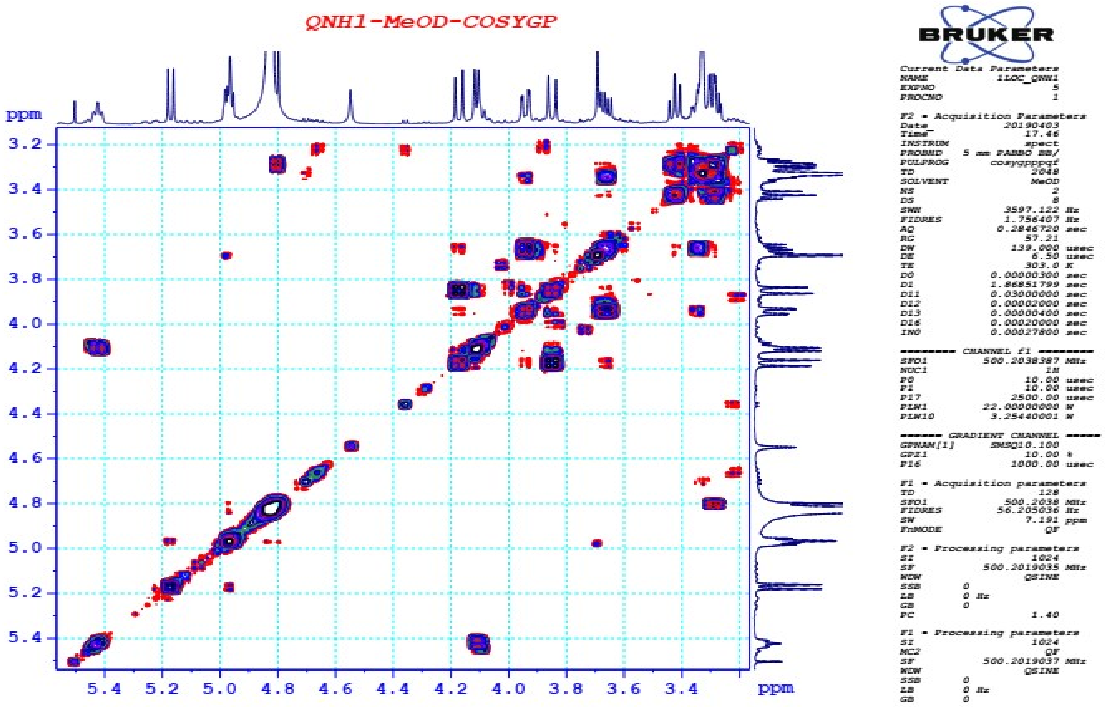


COSY spectrum (expansion) of compound **10**

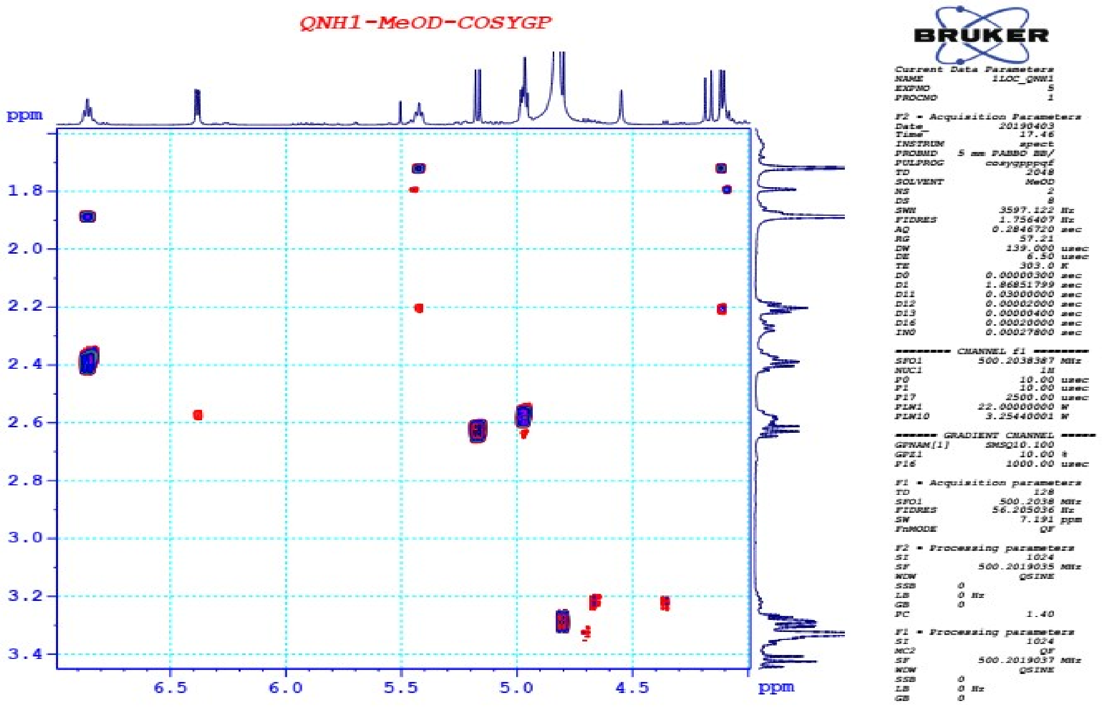


COSY spectrum (expansion) of compound **10**

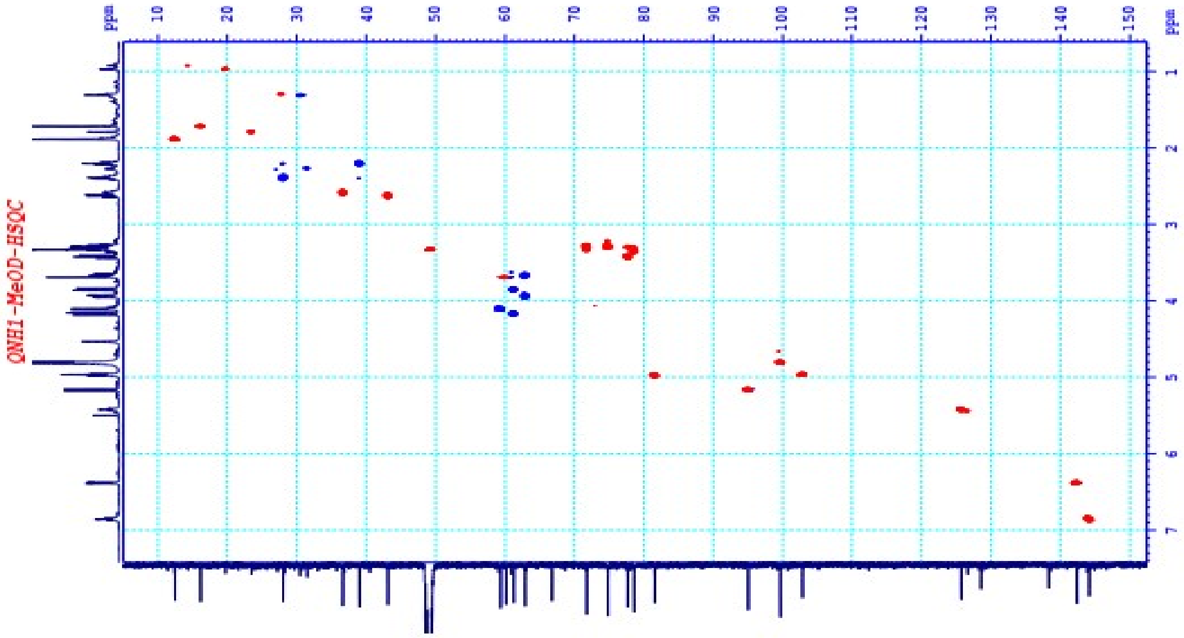


HSQC spectrum of compound **10**

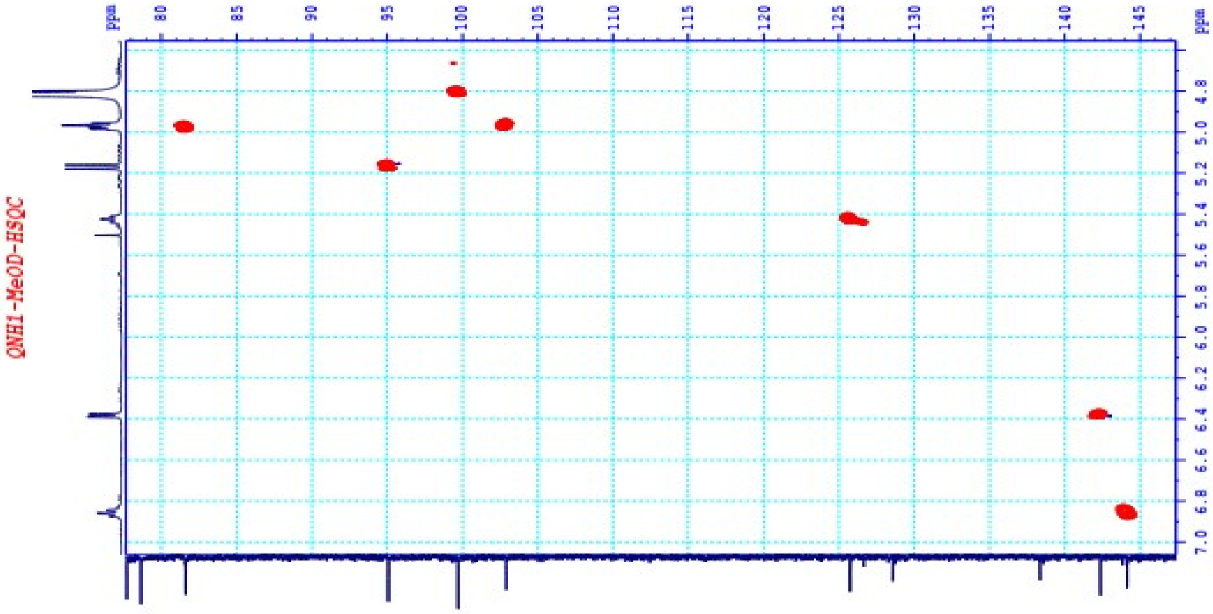


HSQC spectrum (epansion) of compound **10**

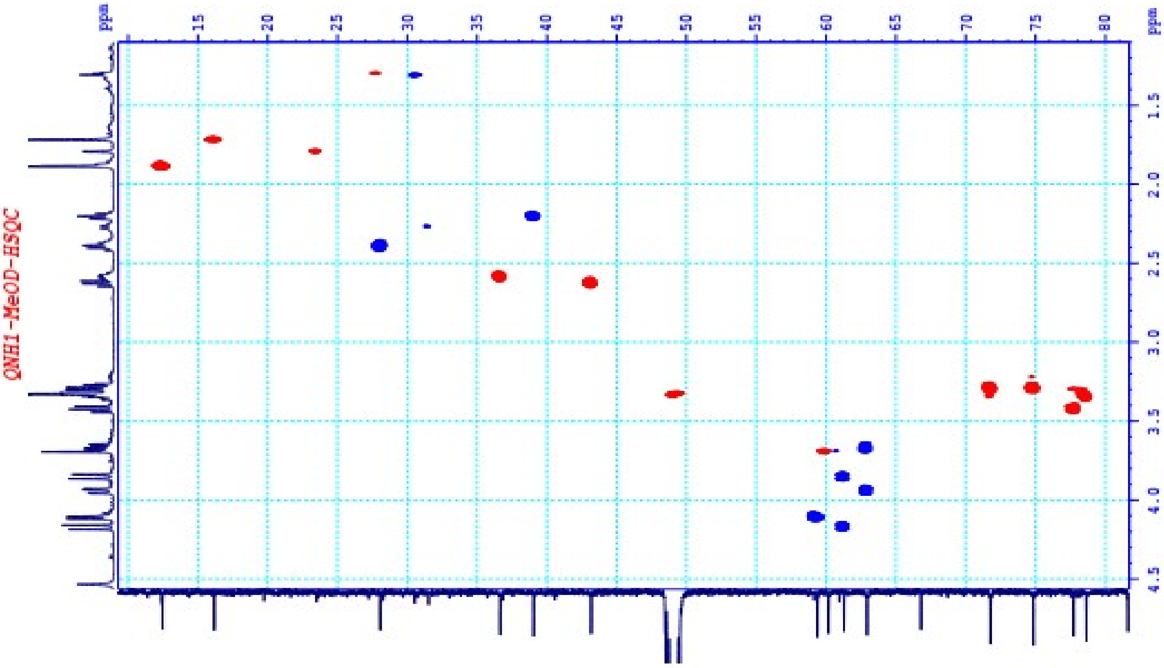


HSQC spectrum (epansion) of compound **10**

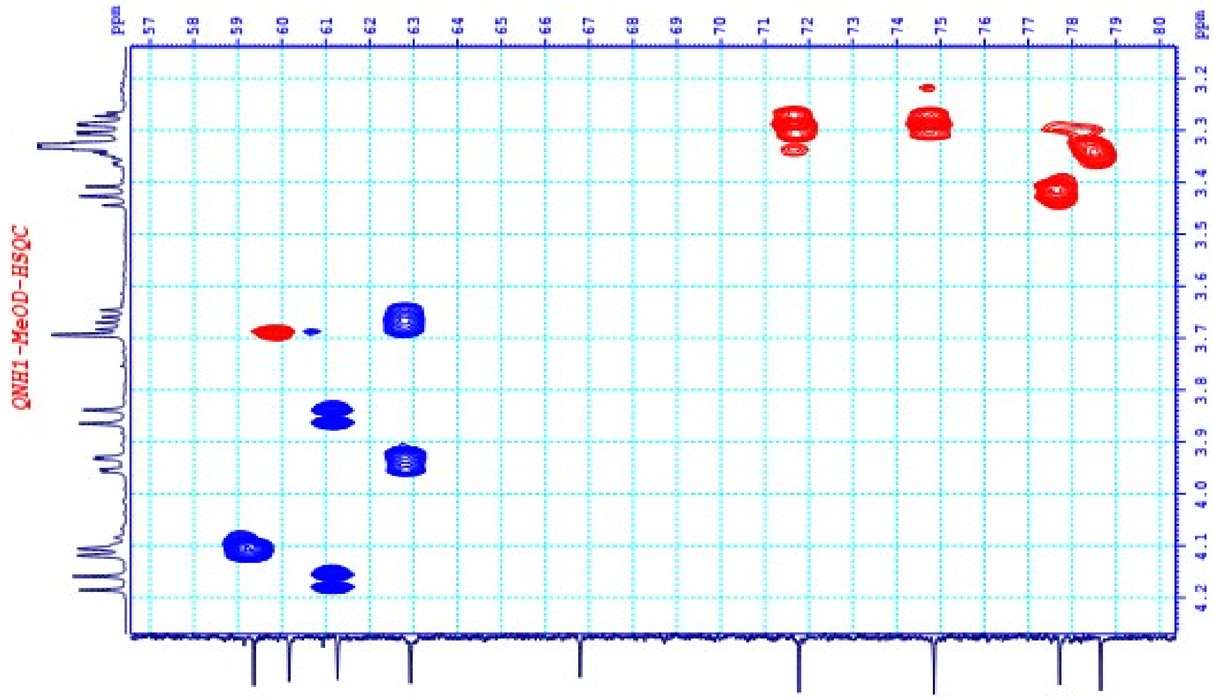


HSQC spectrum (epansion) of compound **10**

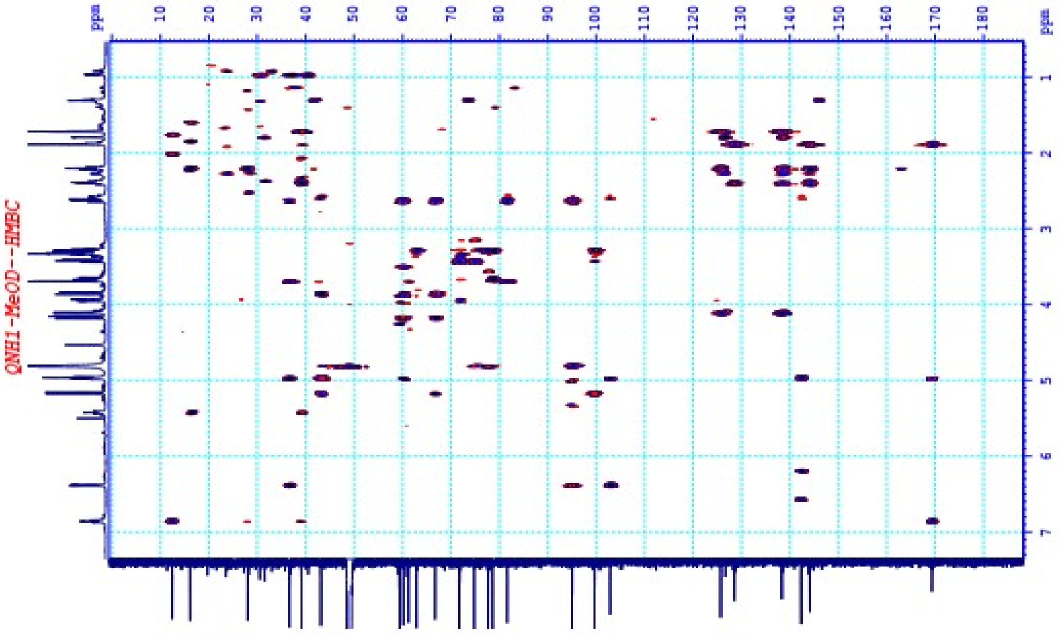


HMBC spectrum of compound **10**

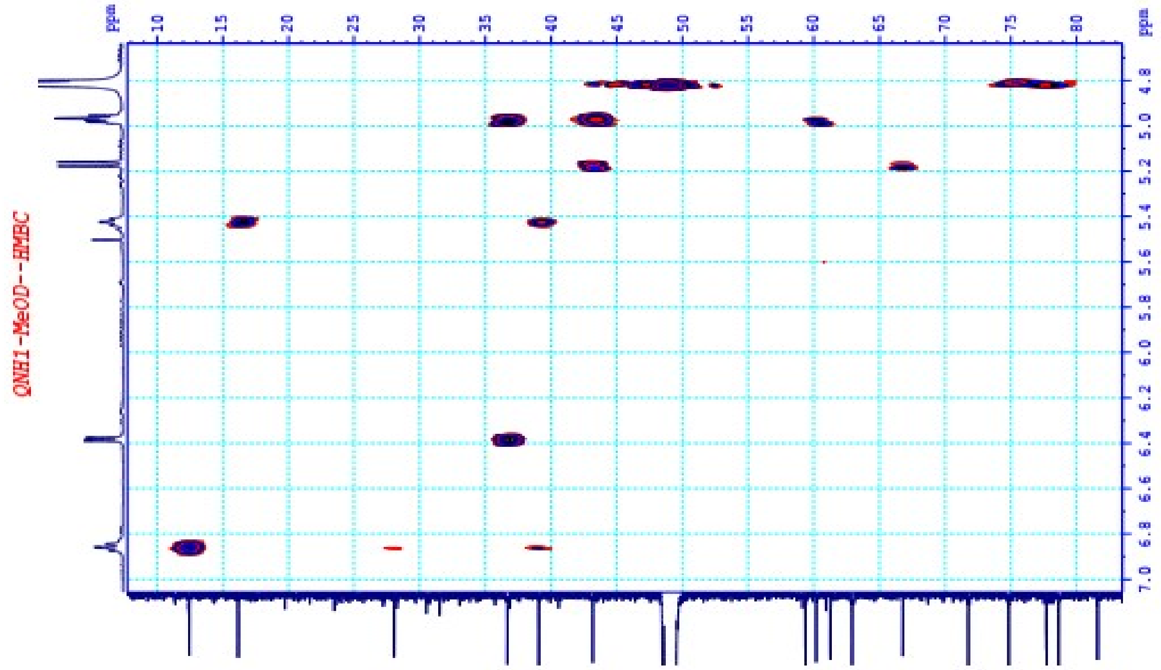


HMBC spectrum (epansion) of compound **10**

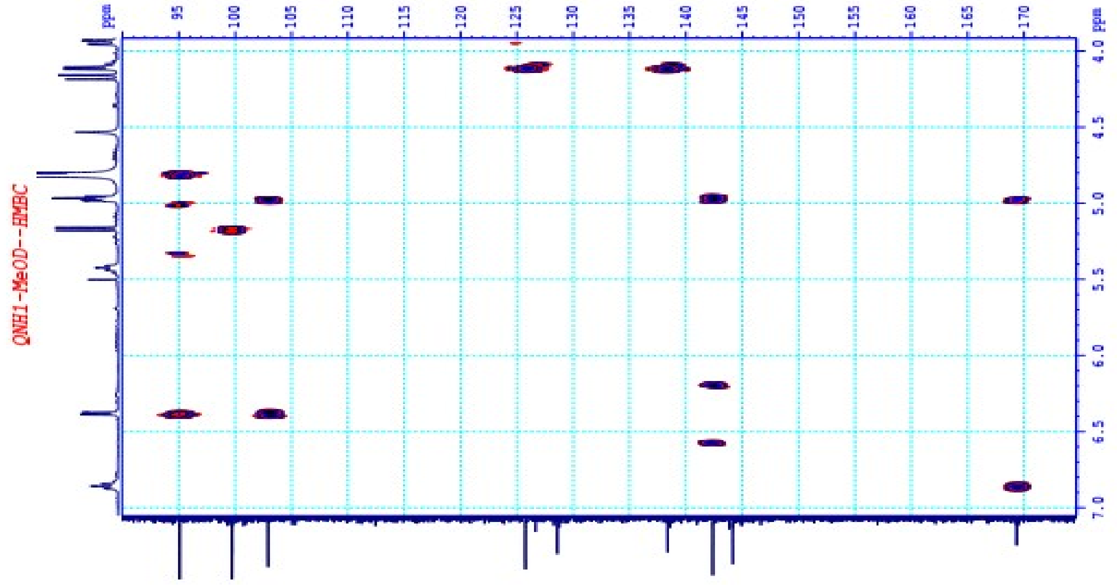


HMBC spectrum (epansion) of compound **10**

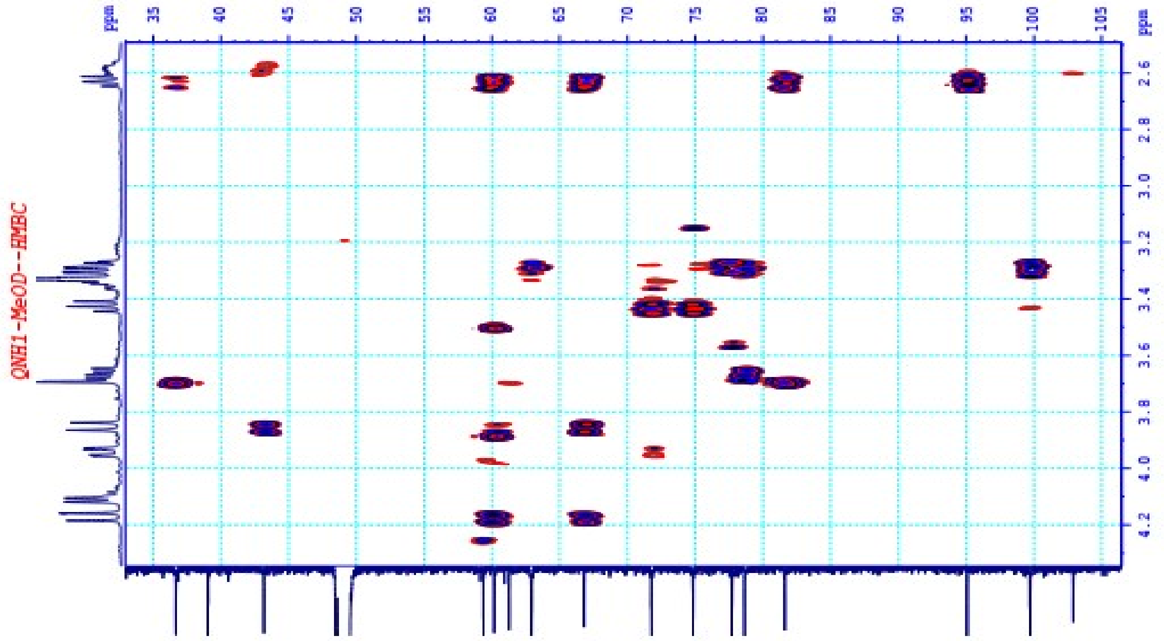


HMBC spectrum (epansion) of compound **10**

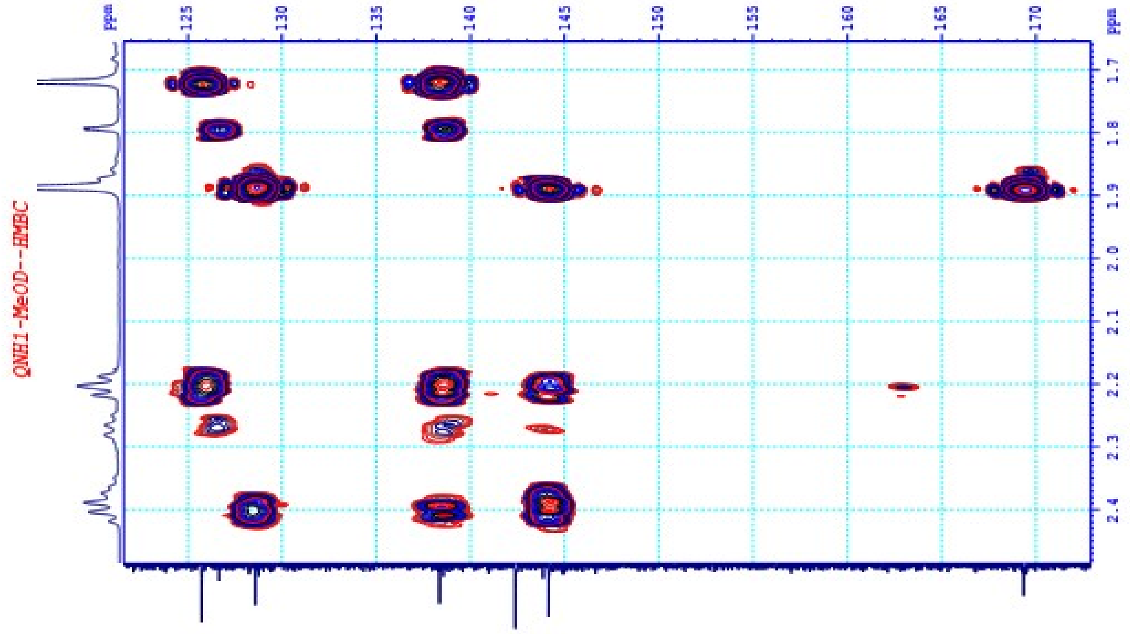


HMBC spectrum (epansion) of compound **10**

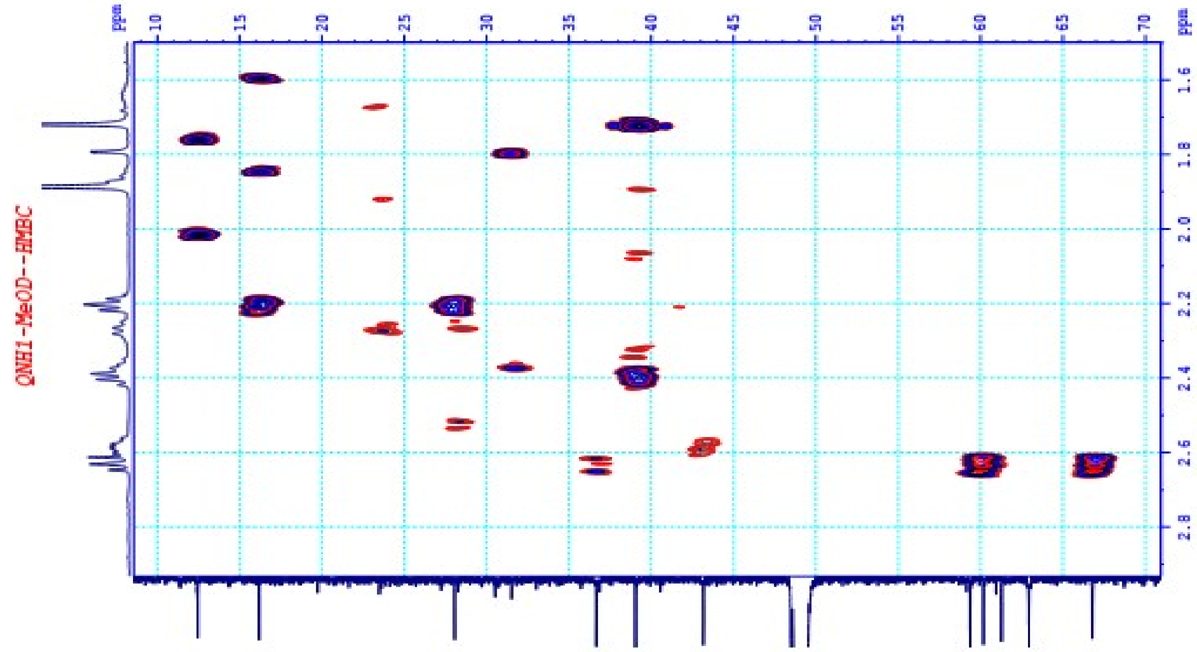


HMBC spectrum (epansion) of compound **10**

## 1.11. Compound 11

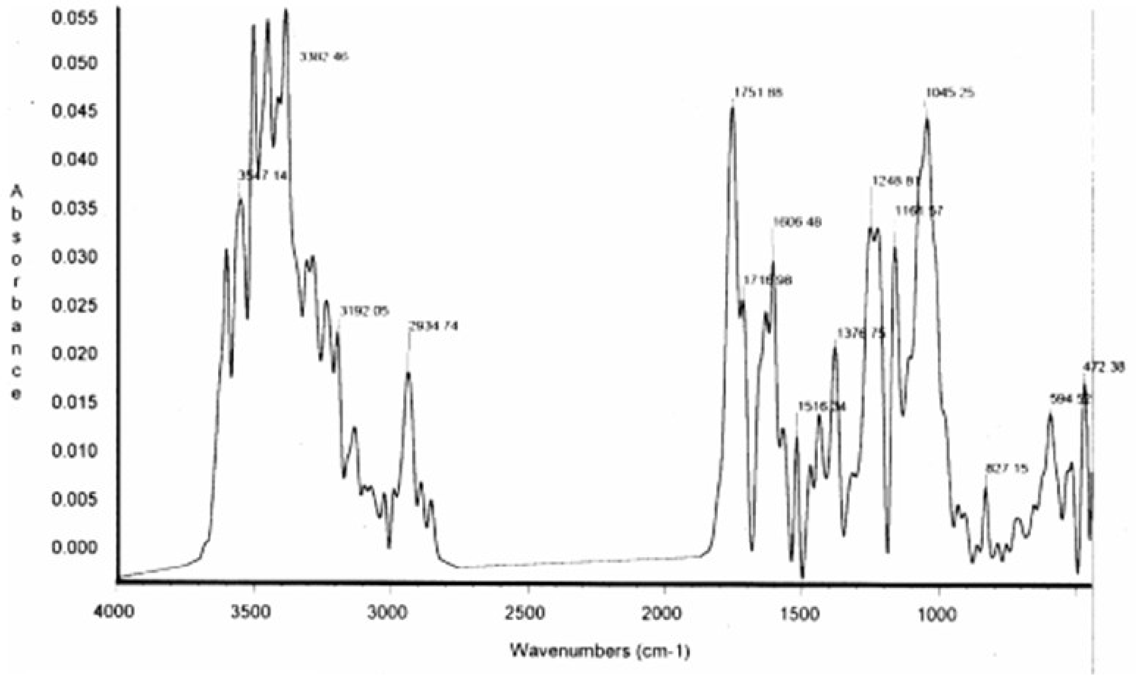


IR spectrum of compound **11**

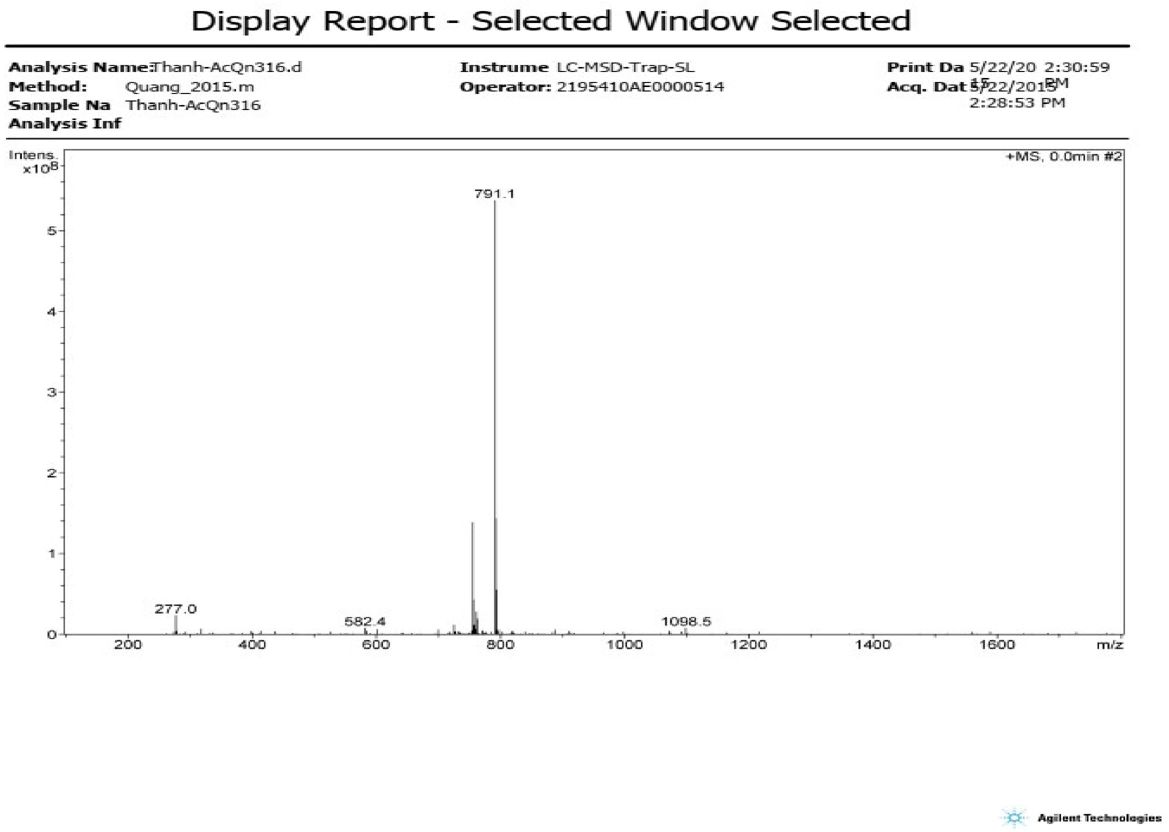


(+)-ESI-MS spectrum of compound **11**

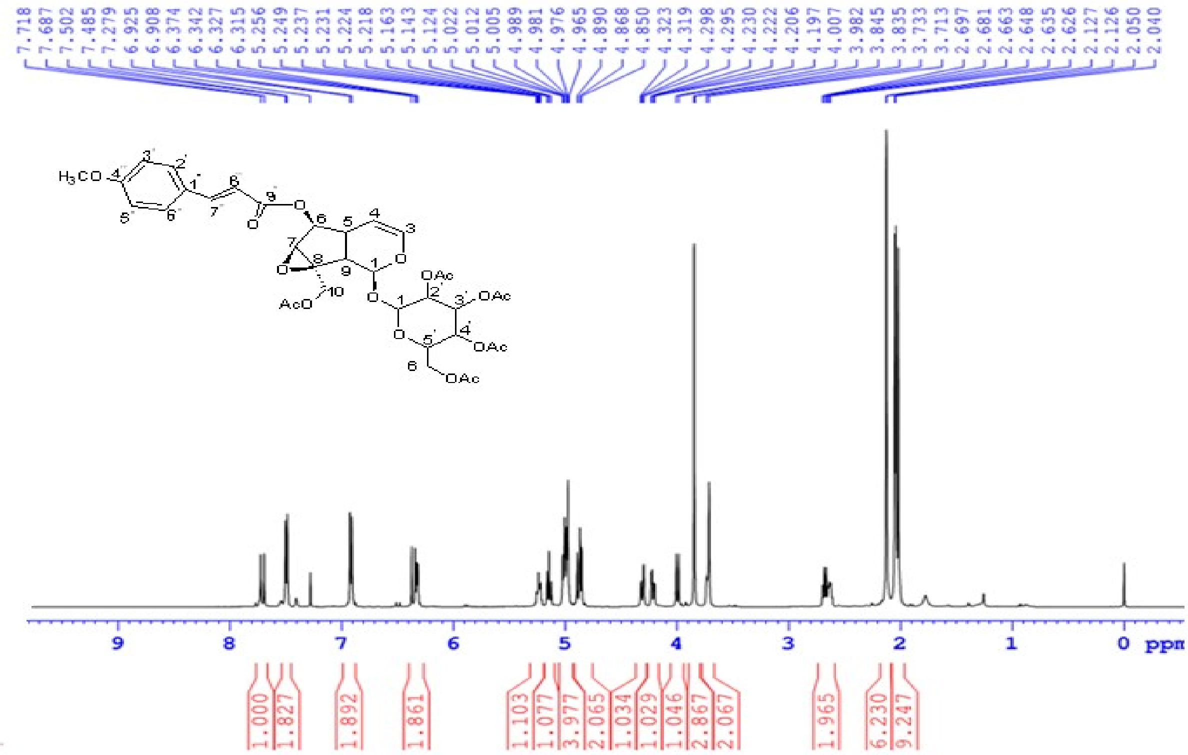

$^1$H-NMR spectrum (500 MHz, $CDCl_3$) of compound **11**

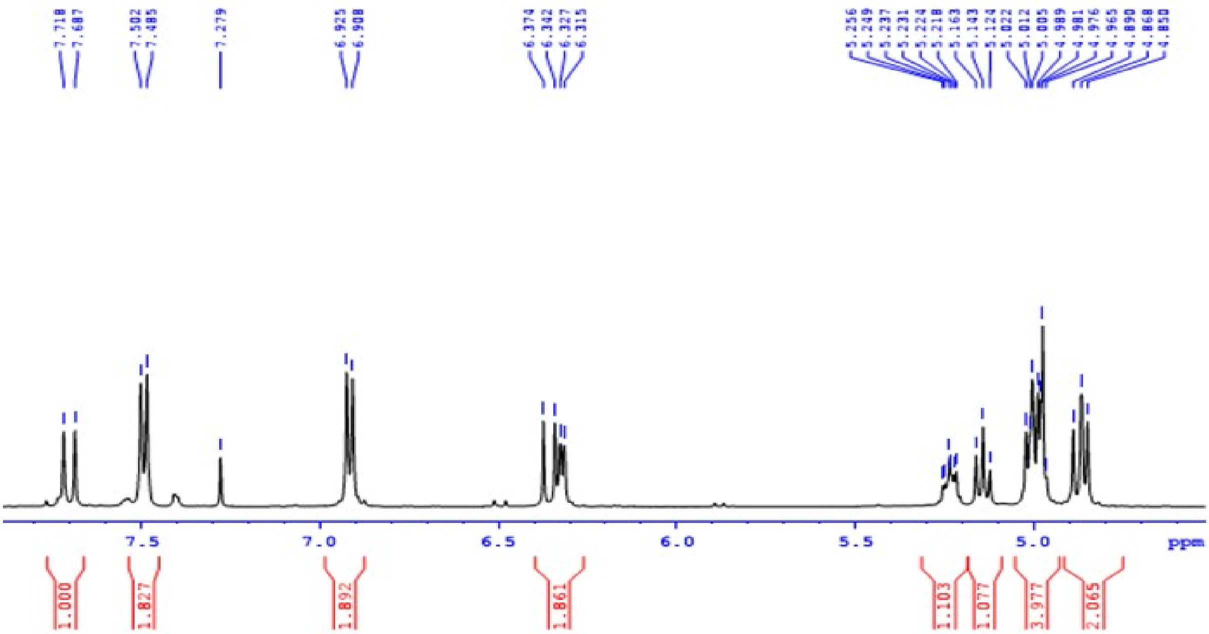

$^1$H-NMR spectrum (expansion) of compound **11**

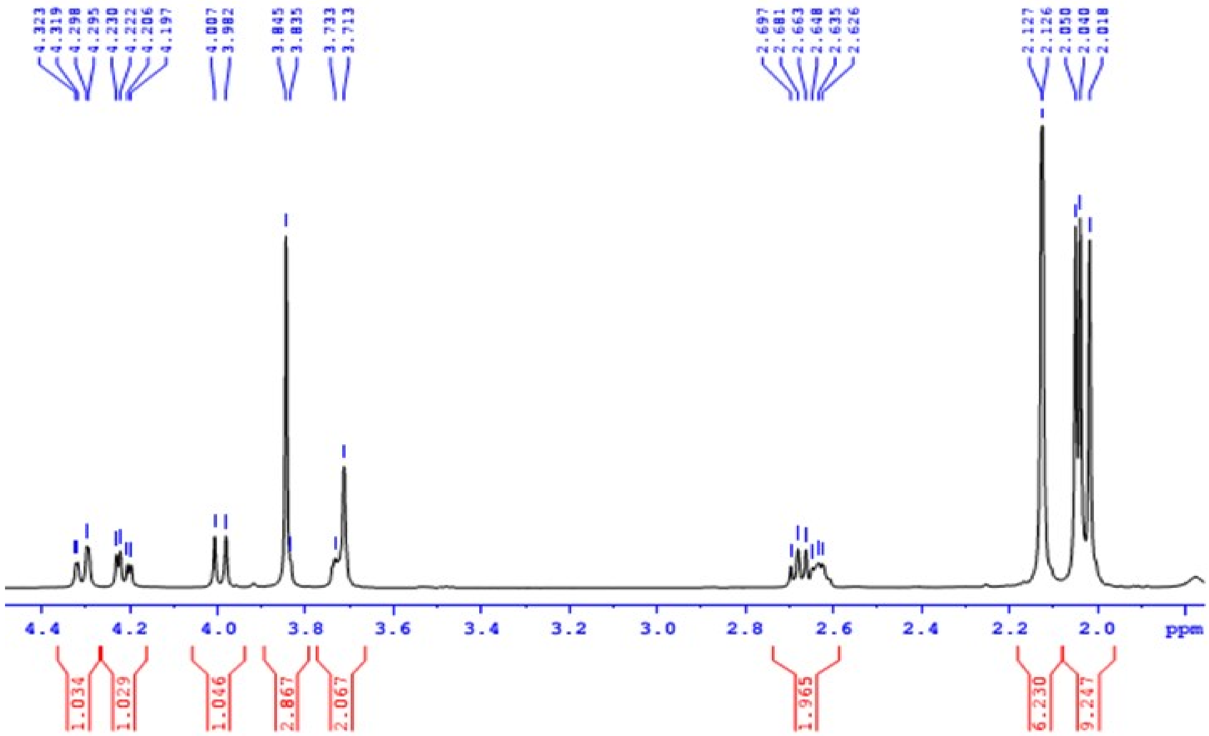


$^{1}$H-NMR spectrum (expansion) of compound **11**

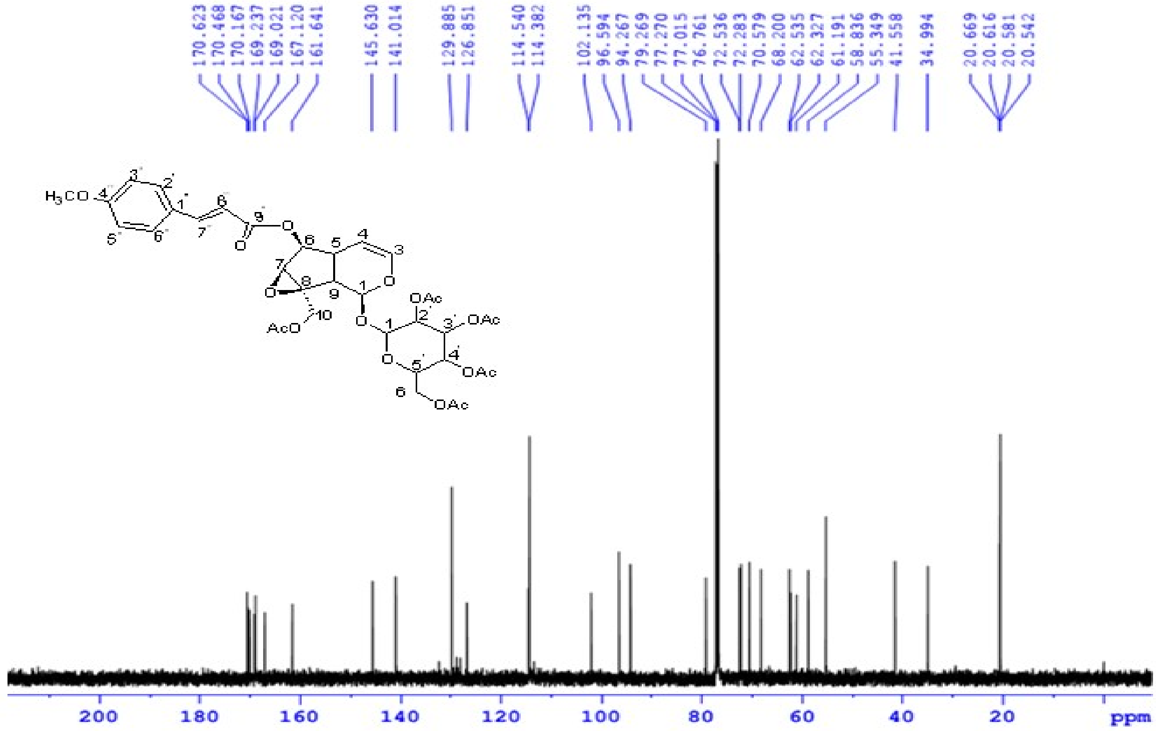


$^{13}$C-NMR (125 MHz, $CDCl_3$) spectrum of compound **11**

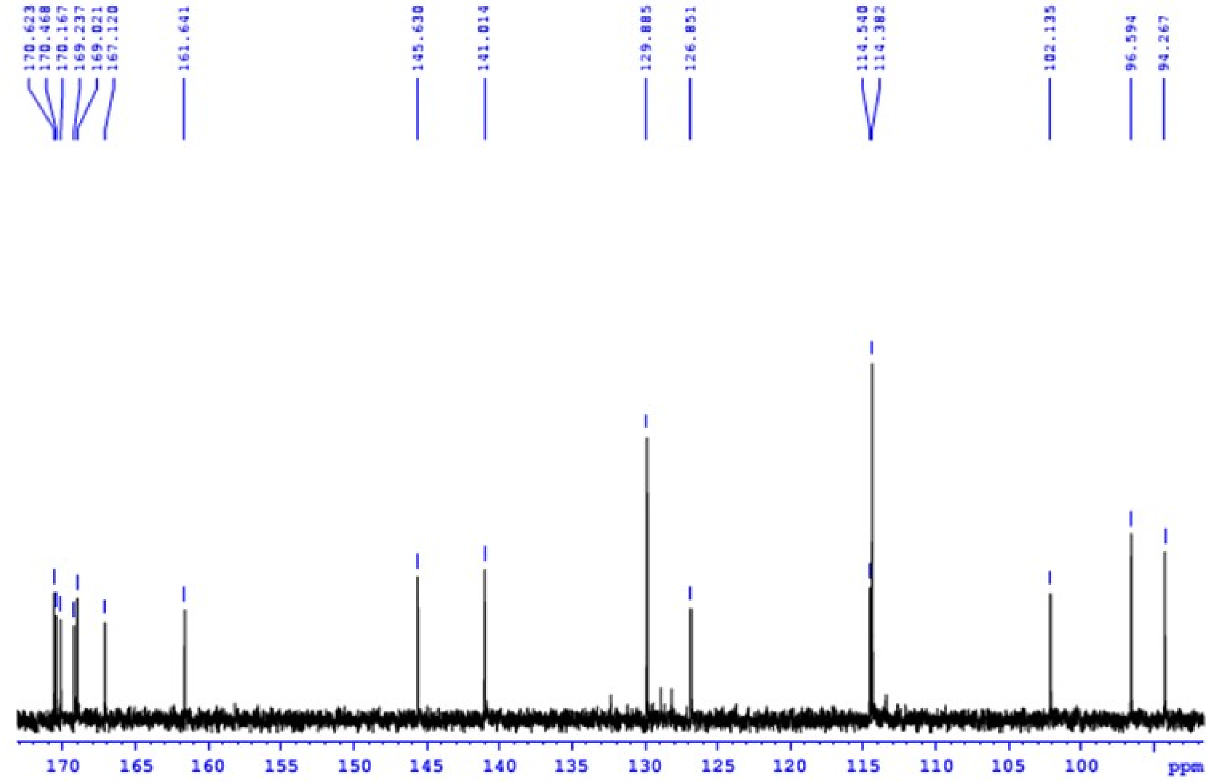


$^{13}$C-NMR spectrum (expansion) of compound **11**

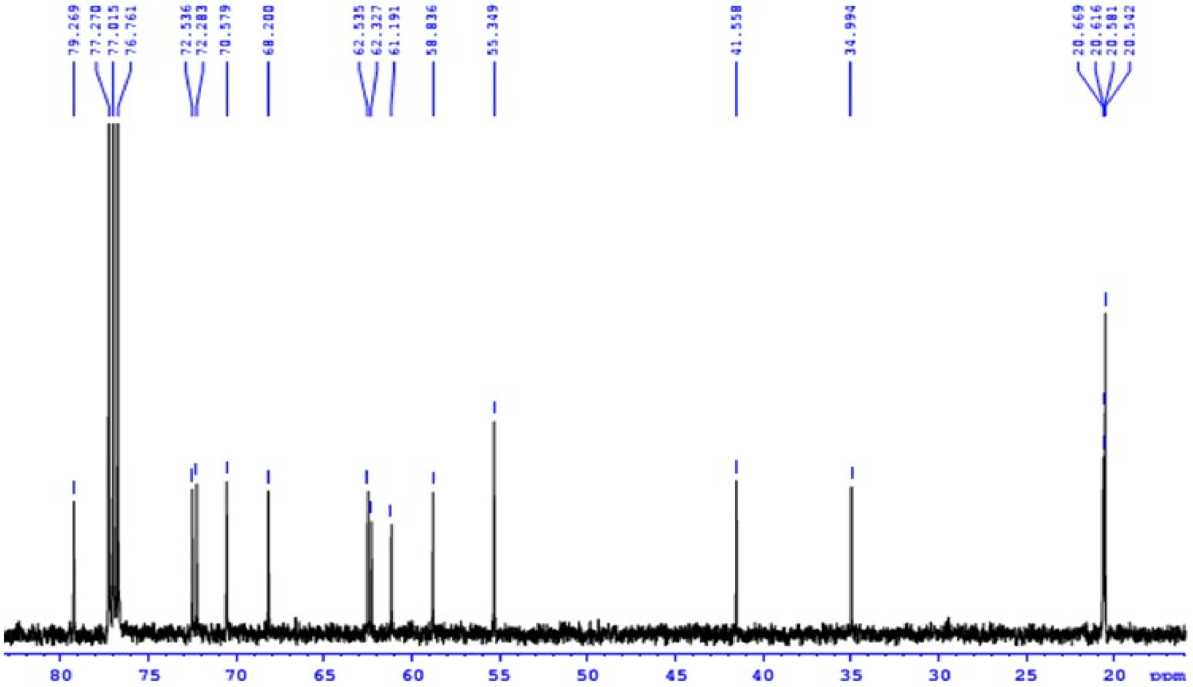


$^{13}$C-NMR spectrum (expansion) of compound **11**

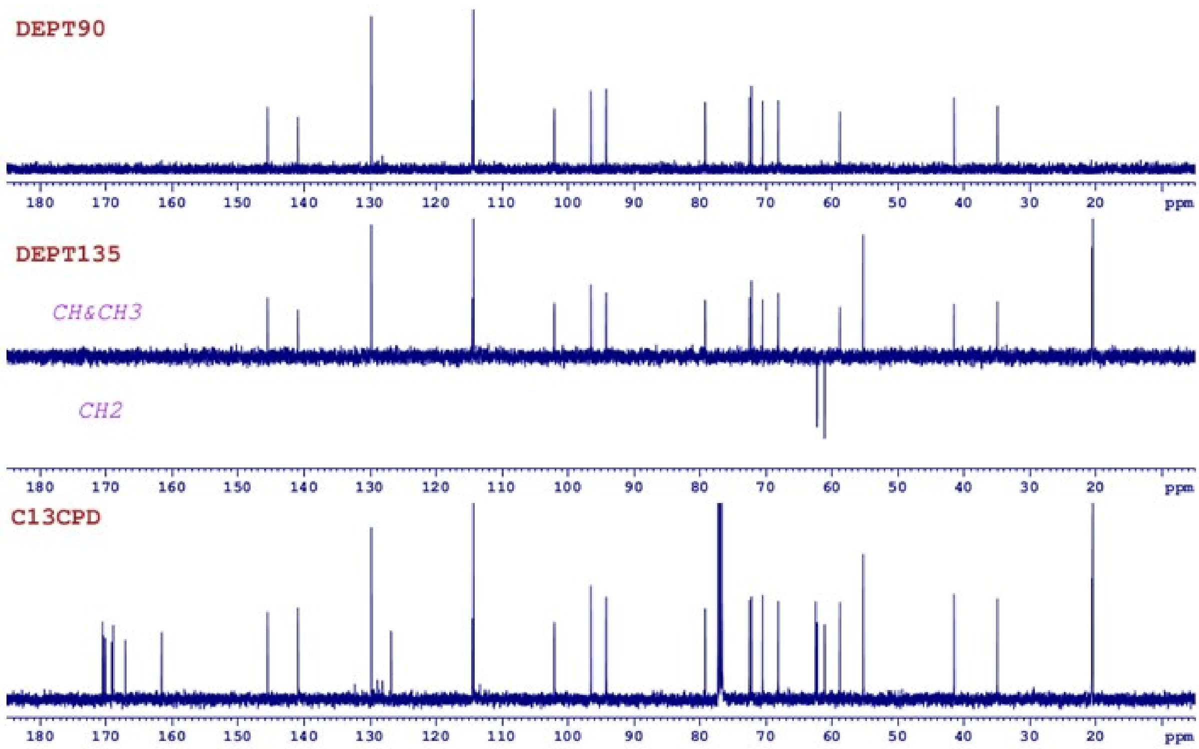


DEPT spectrum of compound **11**

## 1.12. Compound 12

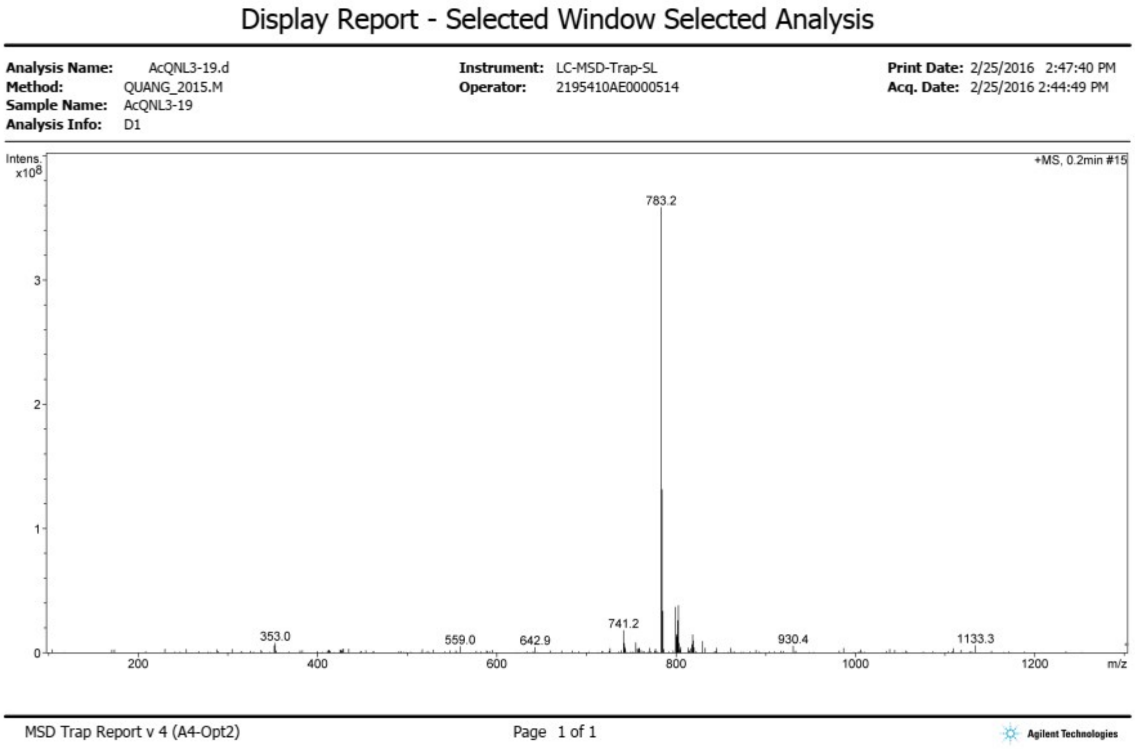


(+)-ESI-MS spectrum of compound **12**

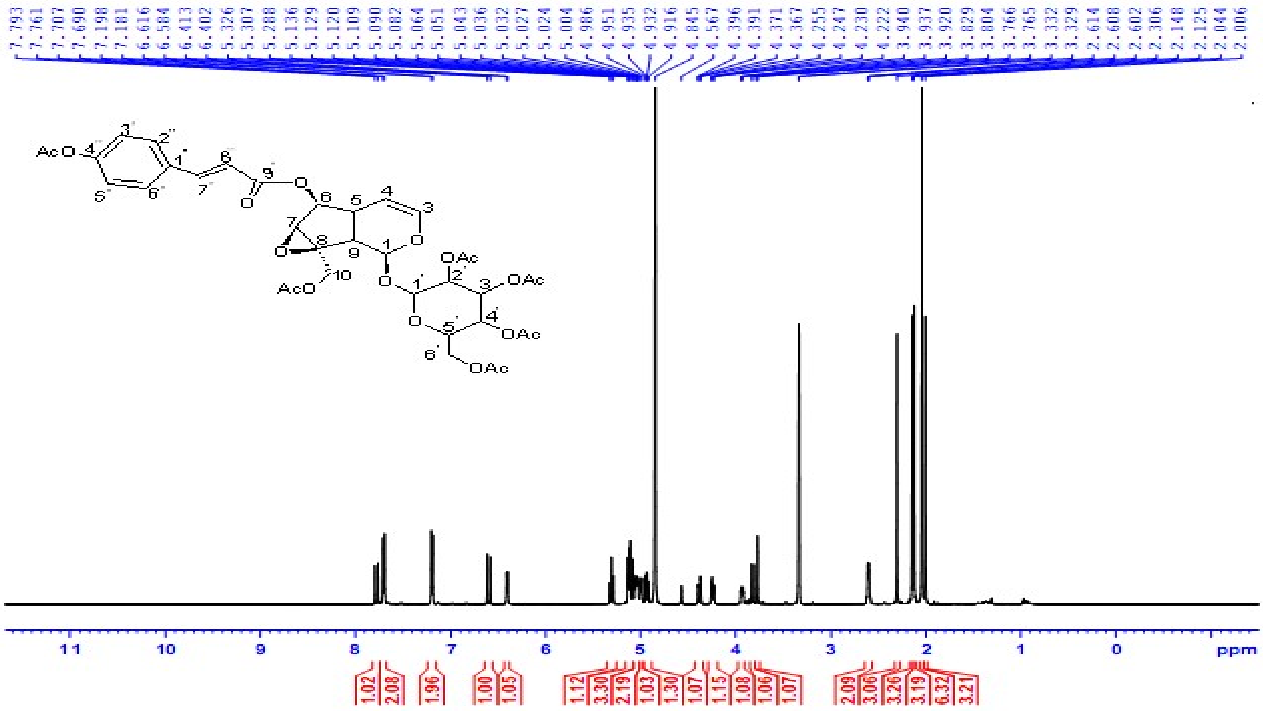


$^{1}$H-NMR spectrum (500 MHz, $CDCl_3$) of compound **12**

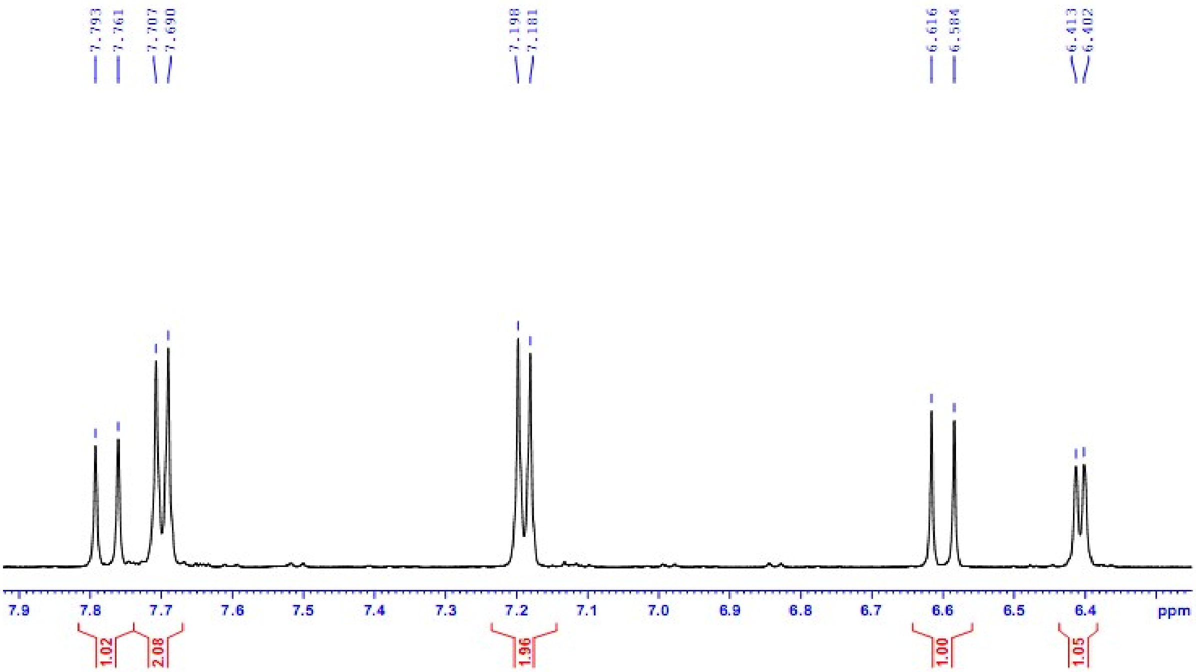


$^1$H-NMR spectrum (expansion) of compound **12**

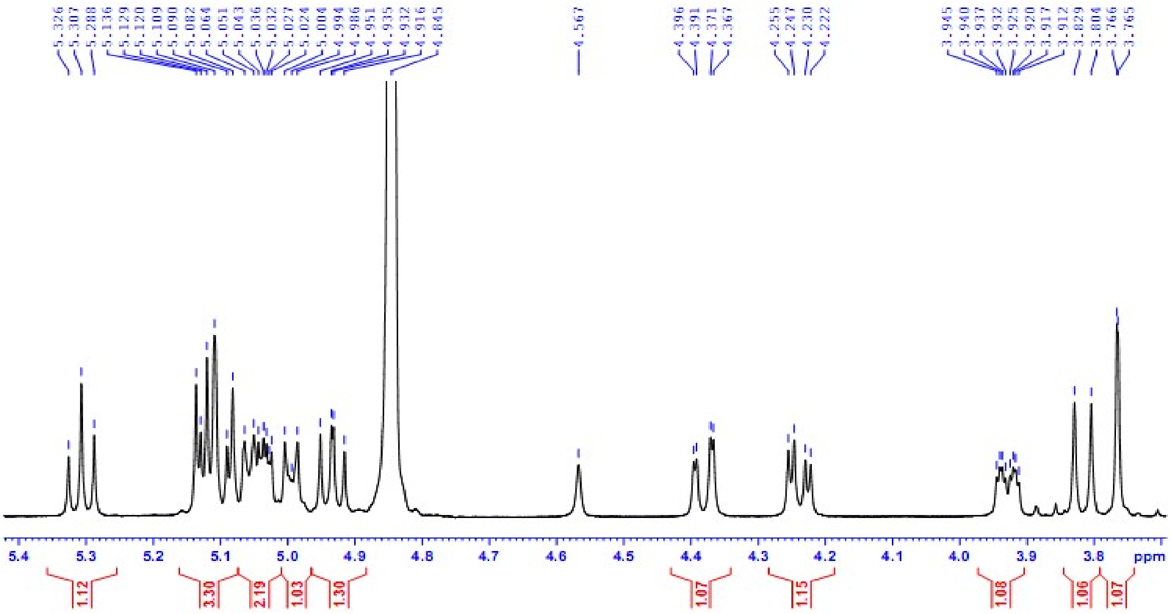


$^1$H-NMR spectrum (expansion) of compound **12**

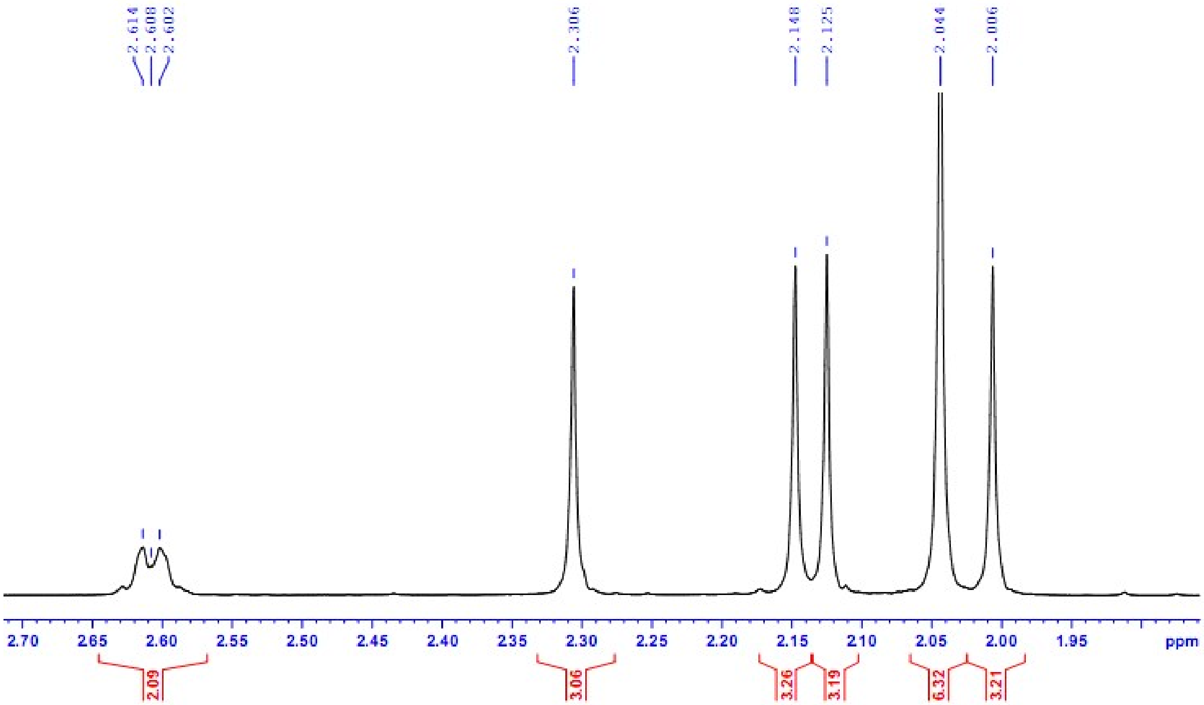


$^{1}$H-NMR spectrum (expansion) of compound **12**

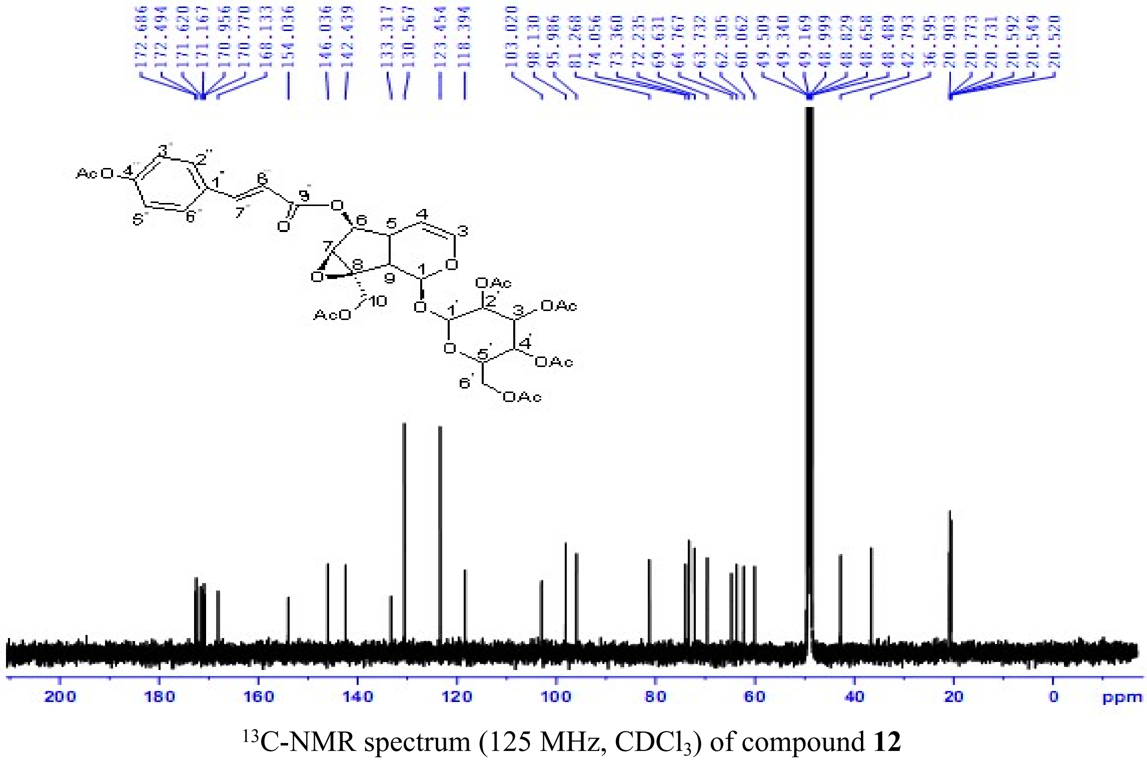


$^{13}$C-NMR spectrum (125 MHz, $CDCl_3$) of compound **12**

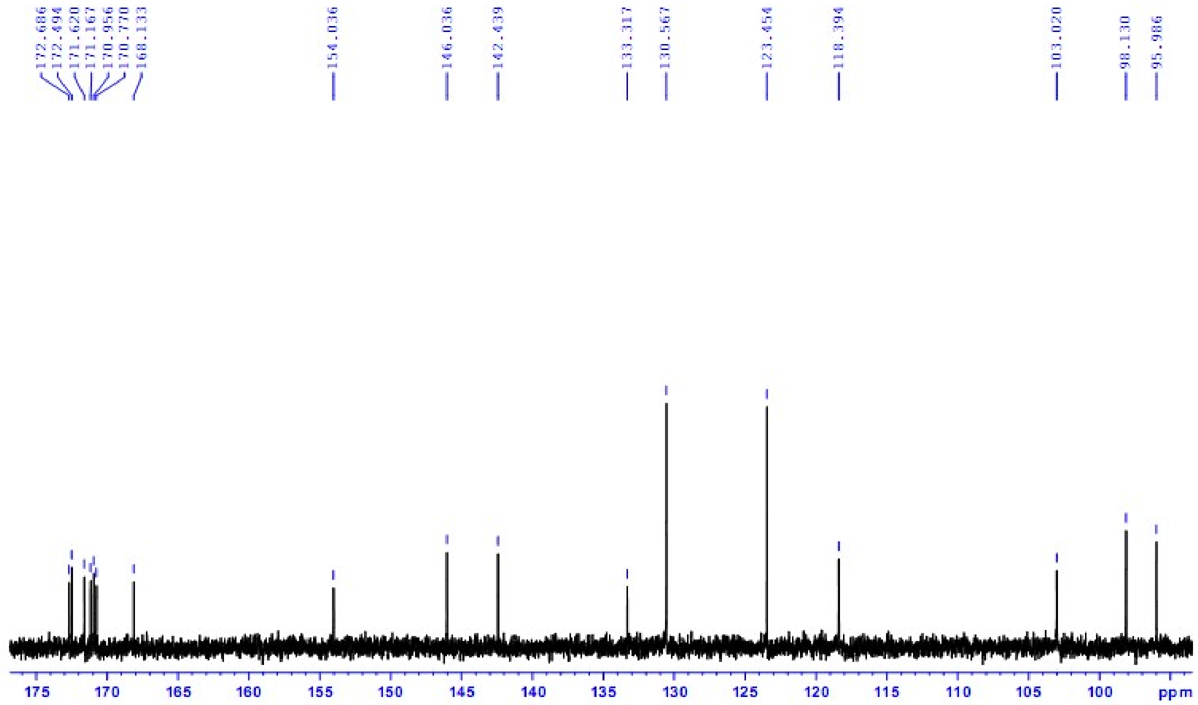


$^{13}$C-NMR spectrum (expansion) of compound **12**

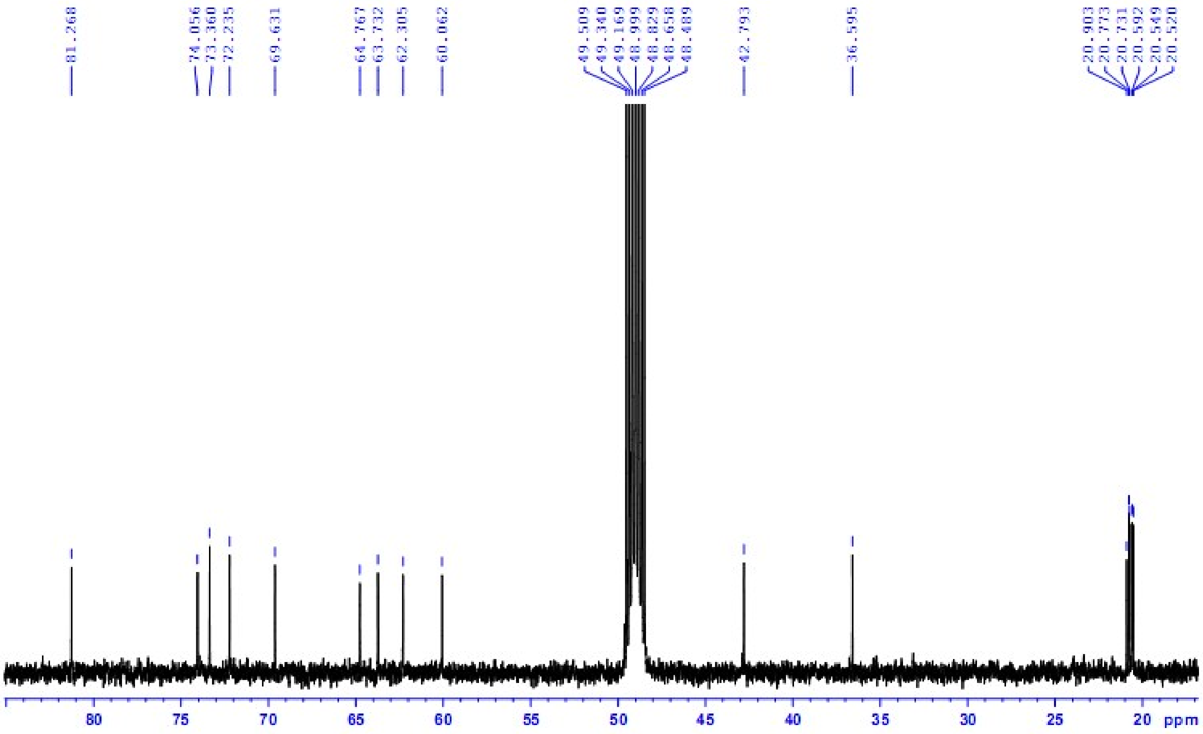


$^{13}$C-NMR spectrum (expansion) of compound **12**

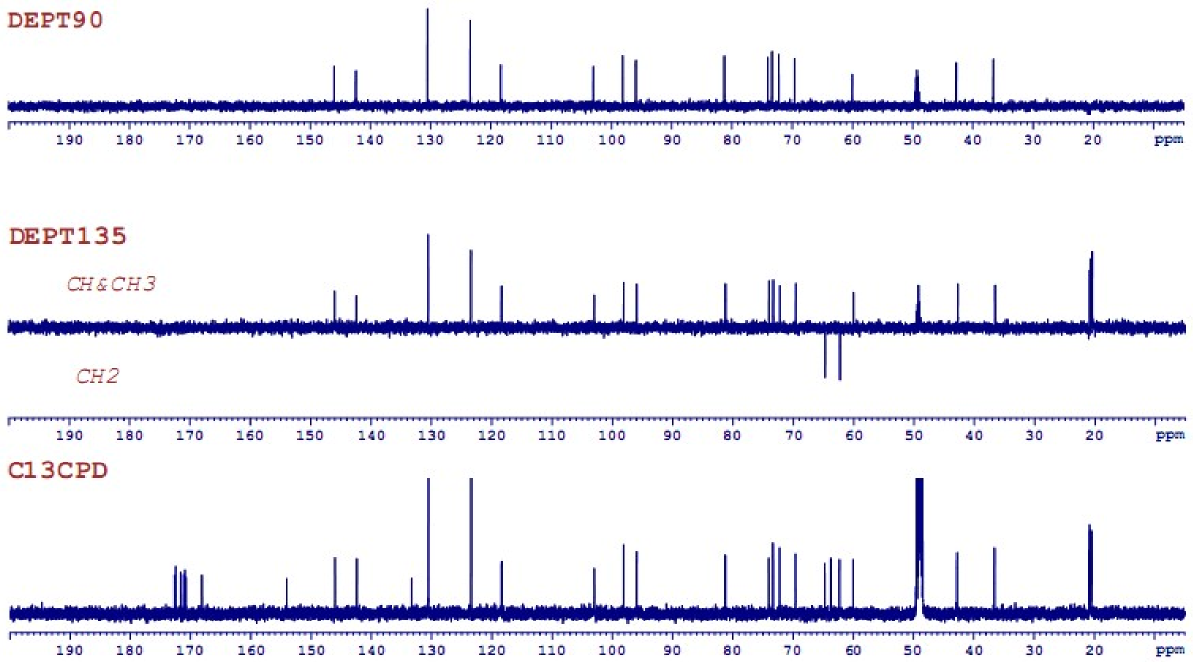

DEPT spectrum of compound **12**

**1.13. Compound 13**

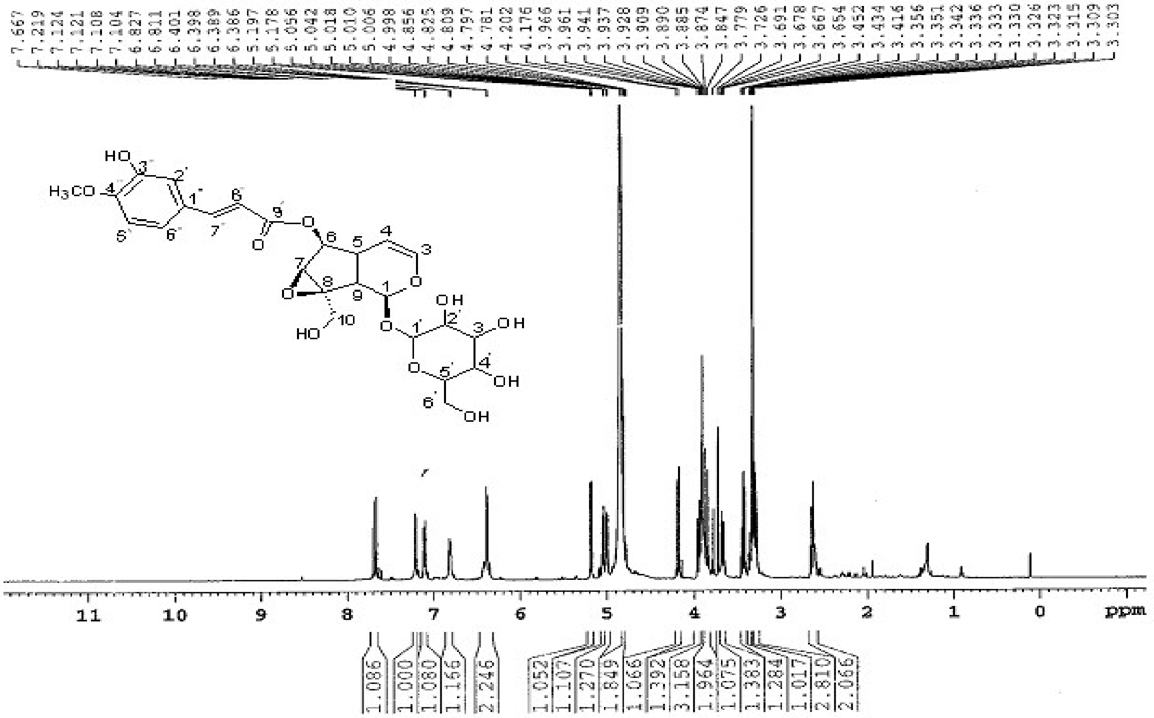


$^1$H-NMR spectrum (500 MHz, $CD_3OD$) of compound **13**

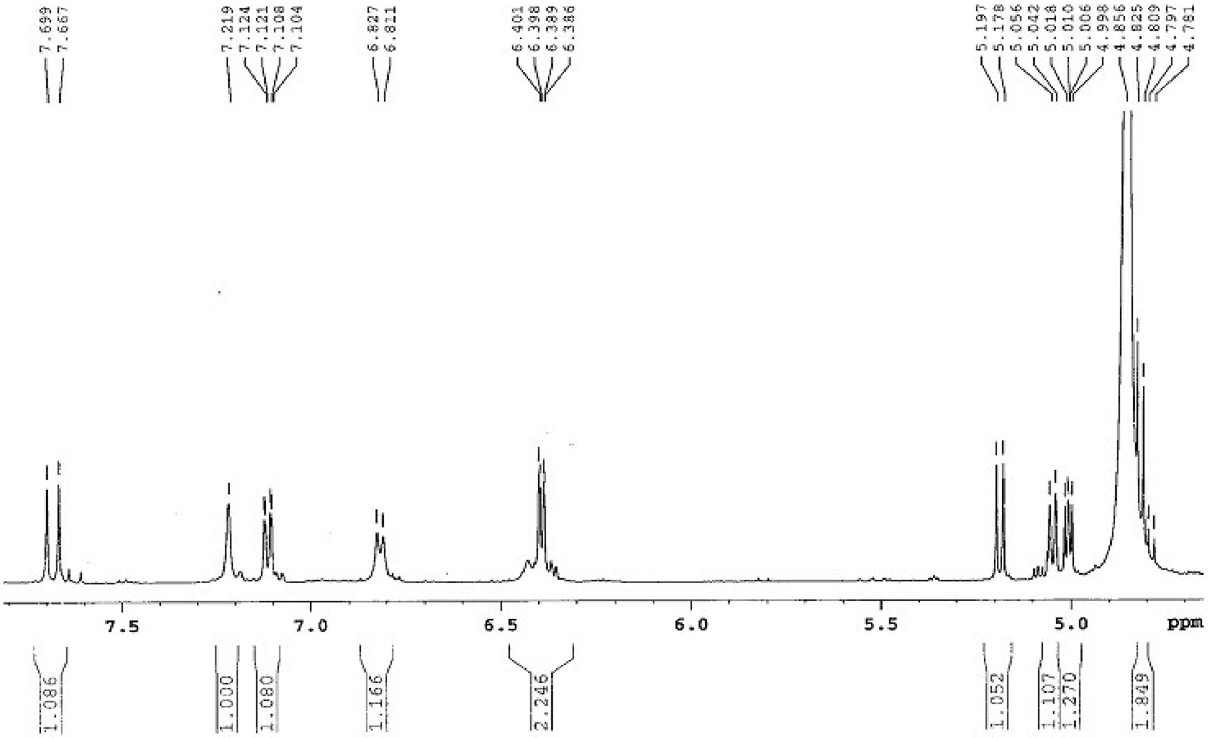


$^1$H-NMR spectrum (expansion) of compound **13**

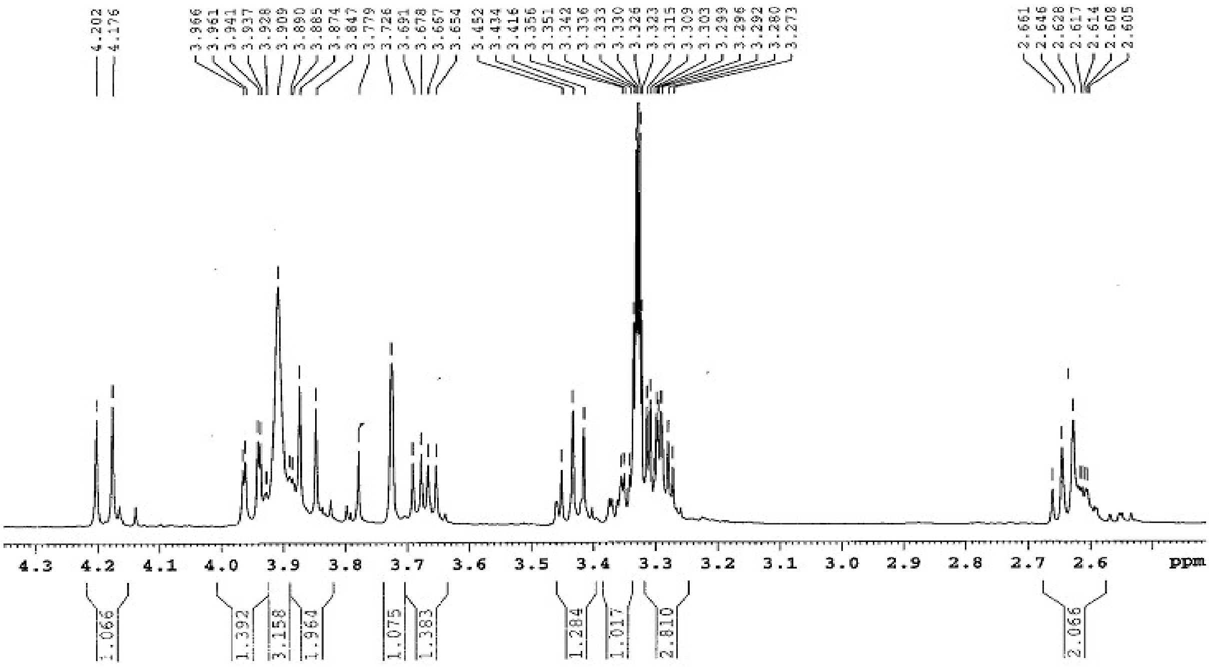


$^{1}$H-NMR spectrum (expansion) of compound **13**

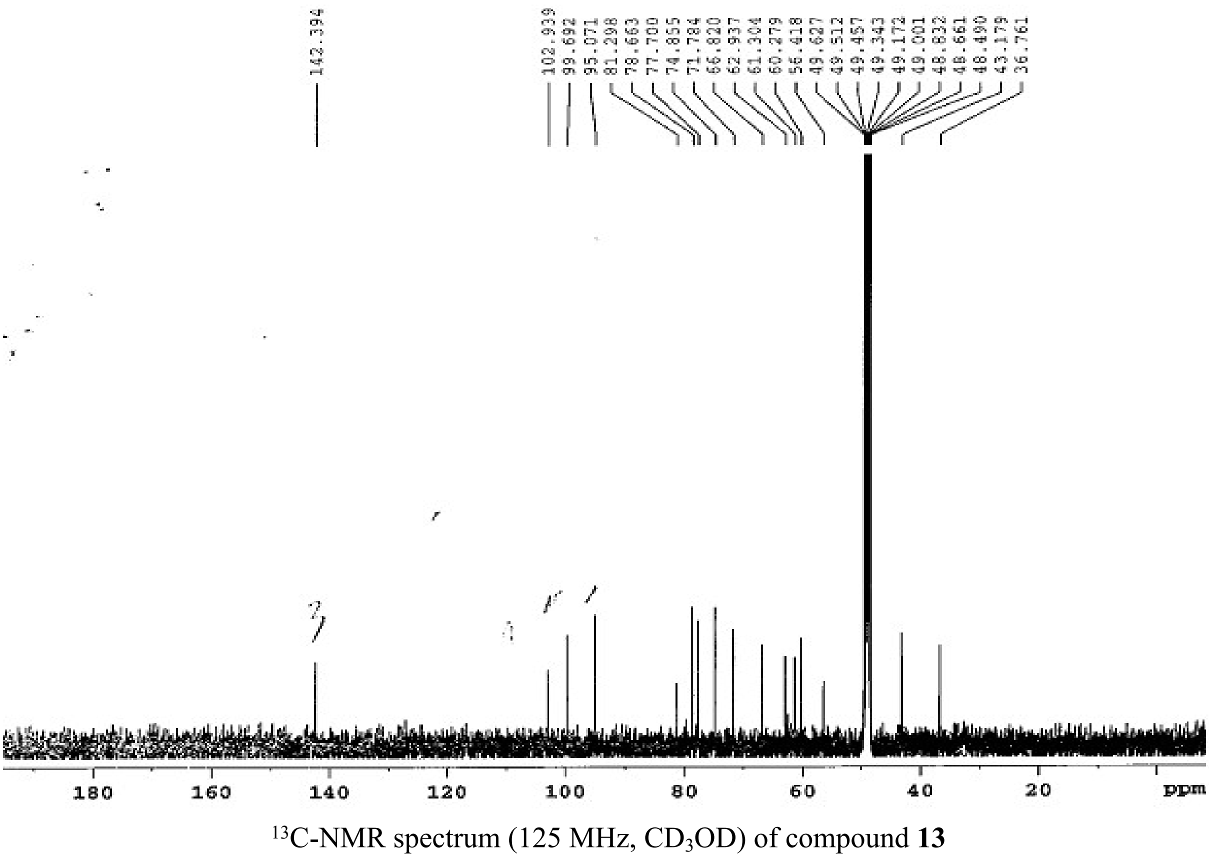


$^{13}$C-NMR spectrum (125 MHz, $CD_3OD$) of compound **13**

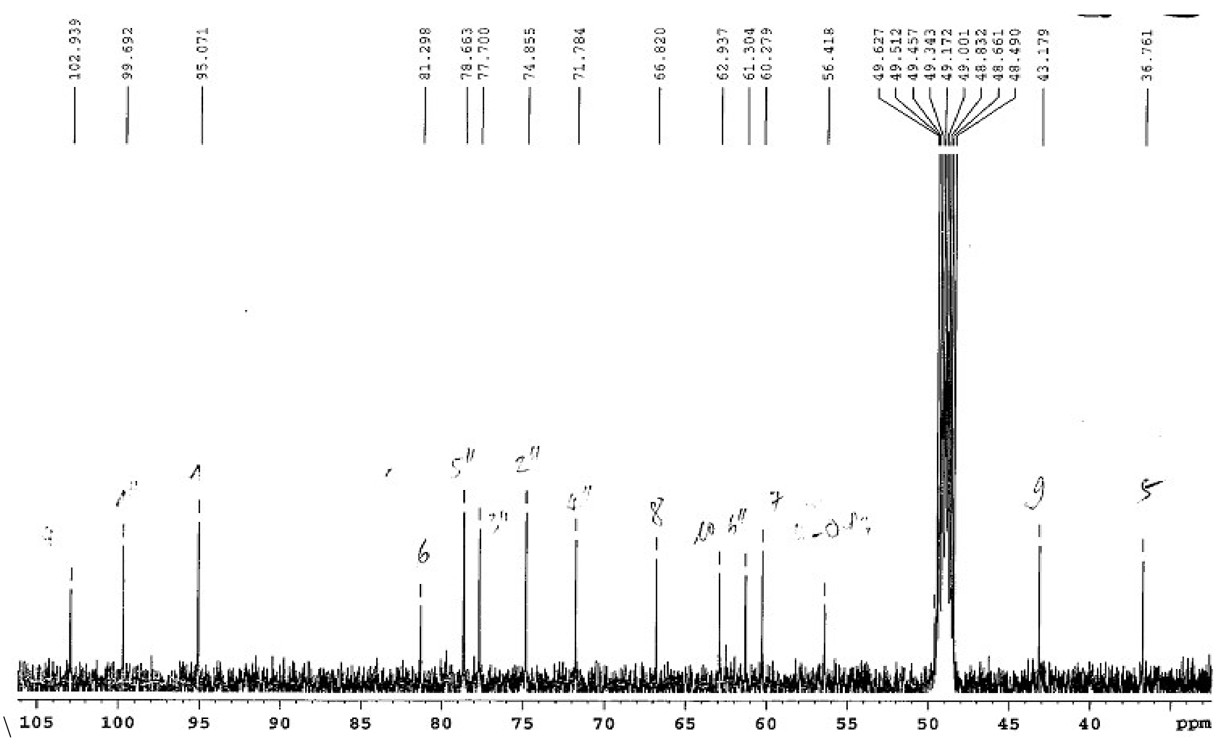


$^{13}$C-NMR spectrum (expansion) of compound **13**

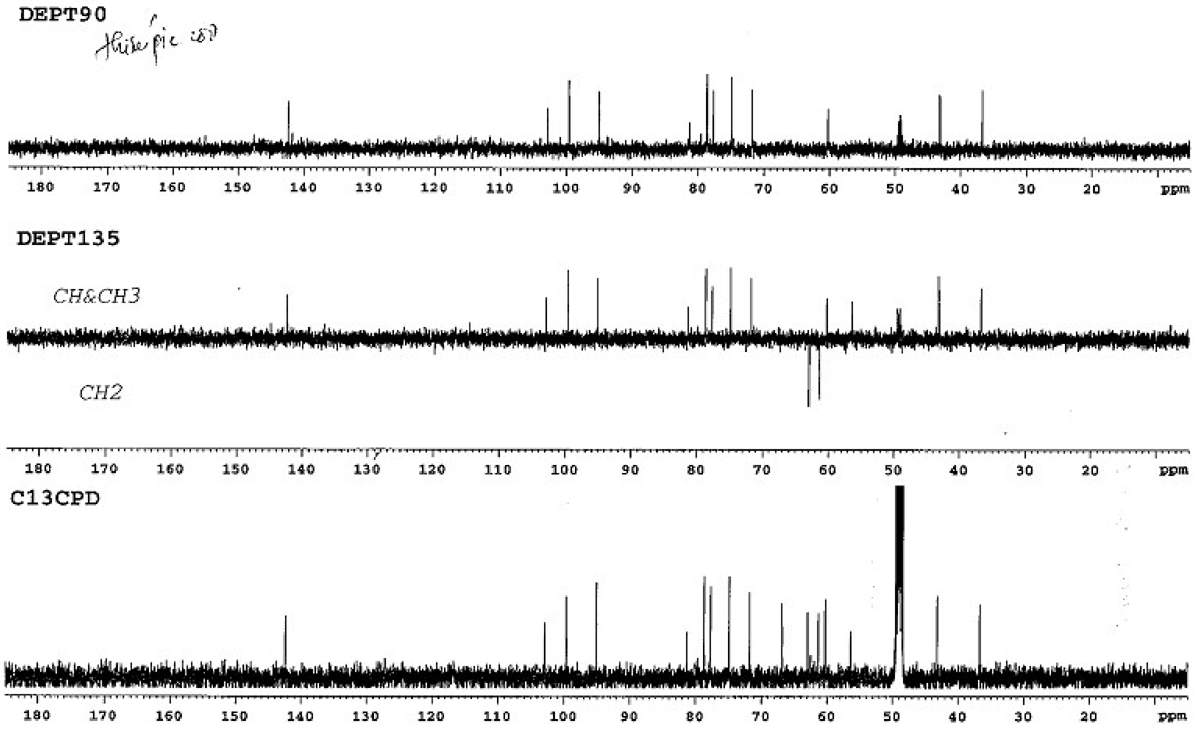


DEPT spectrum of compound **13**

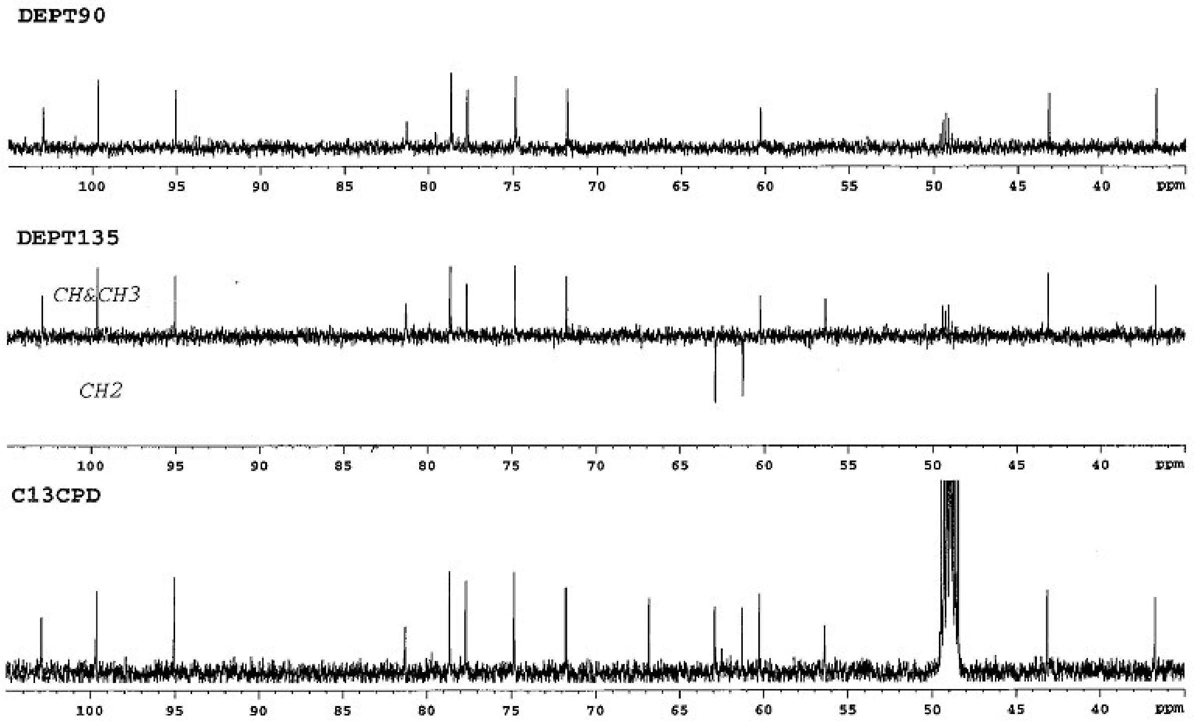


DEPT spectrum (expansion) of compound **13**

**1.14. Compound 14**

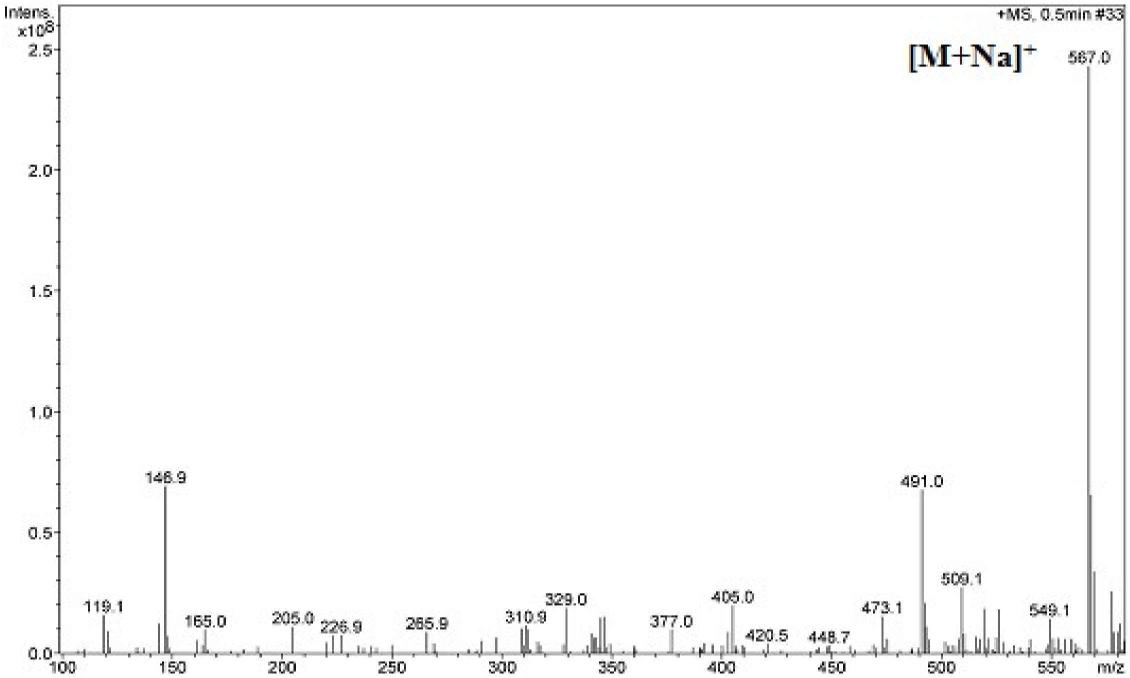


(+)-ESI-MS spectrum of compound **14**

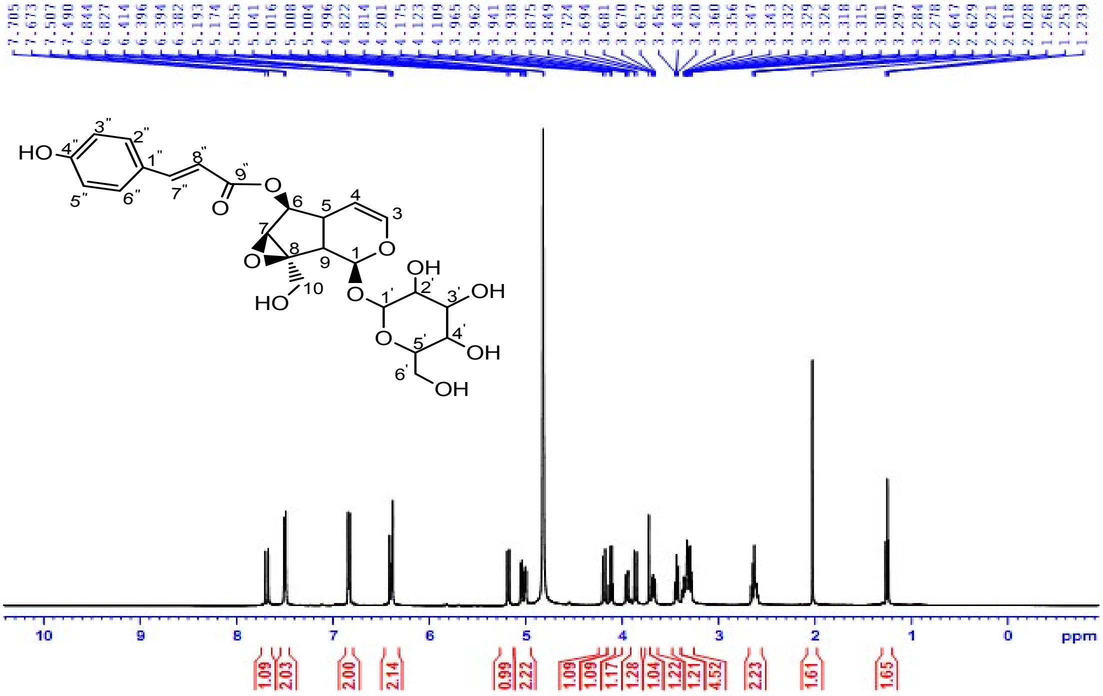


$^{1}$H-NMR spectrum (500 MHz, $CD_3OD$) of compound **14**

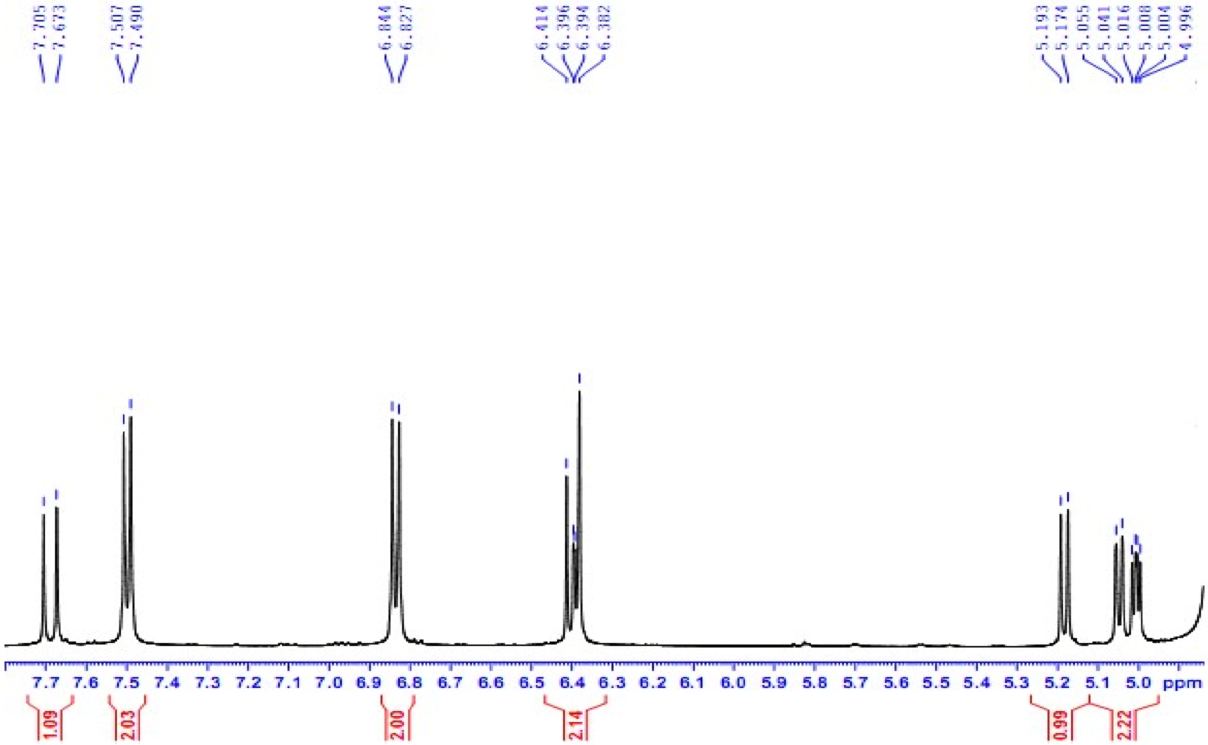


$^1$H-NMR spectrum (expansion) of compound **14**

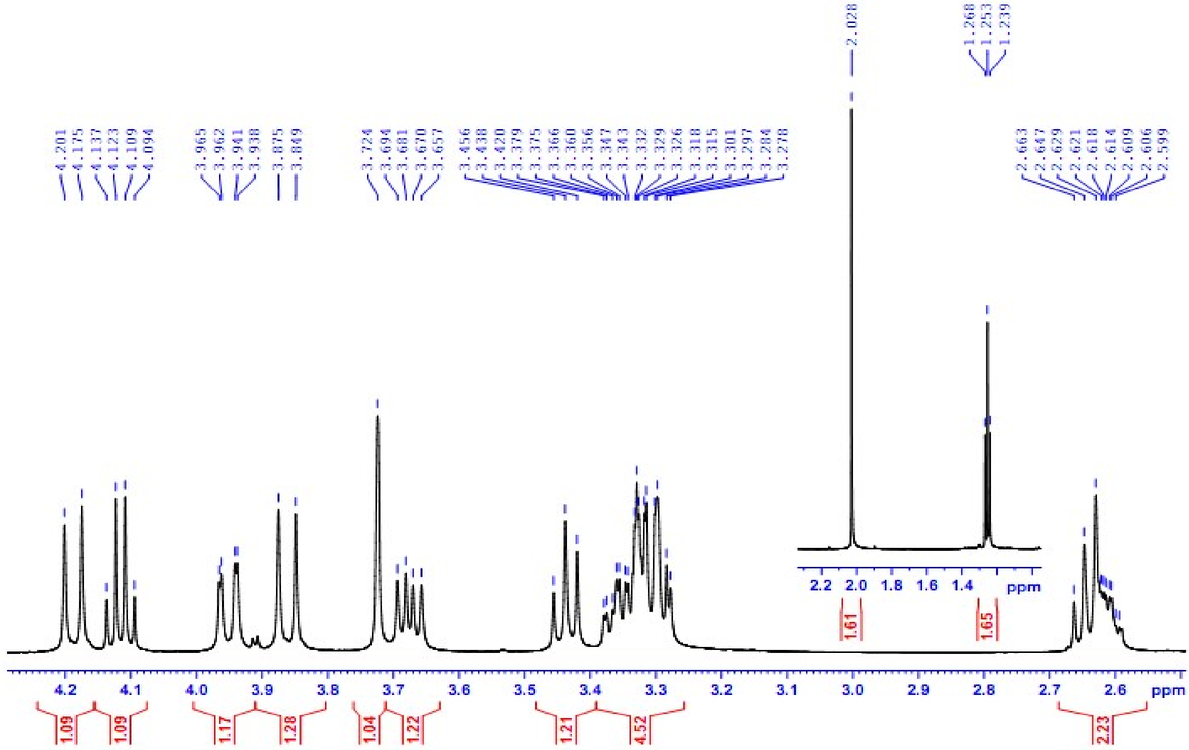


$^1$H-NMR spectrum (expansion) of compound **14**

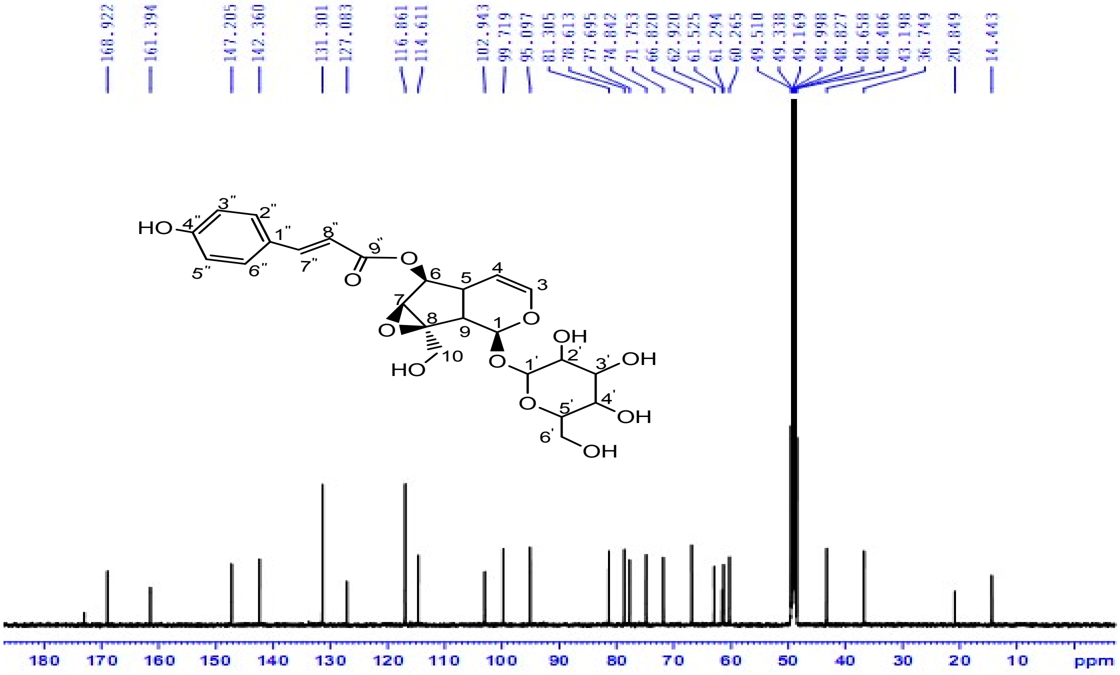


$^{13}$C-NMR spectrum (125 MHz, $CD_3OD$) of compound **14**

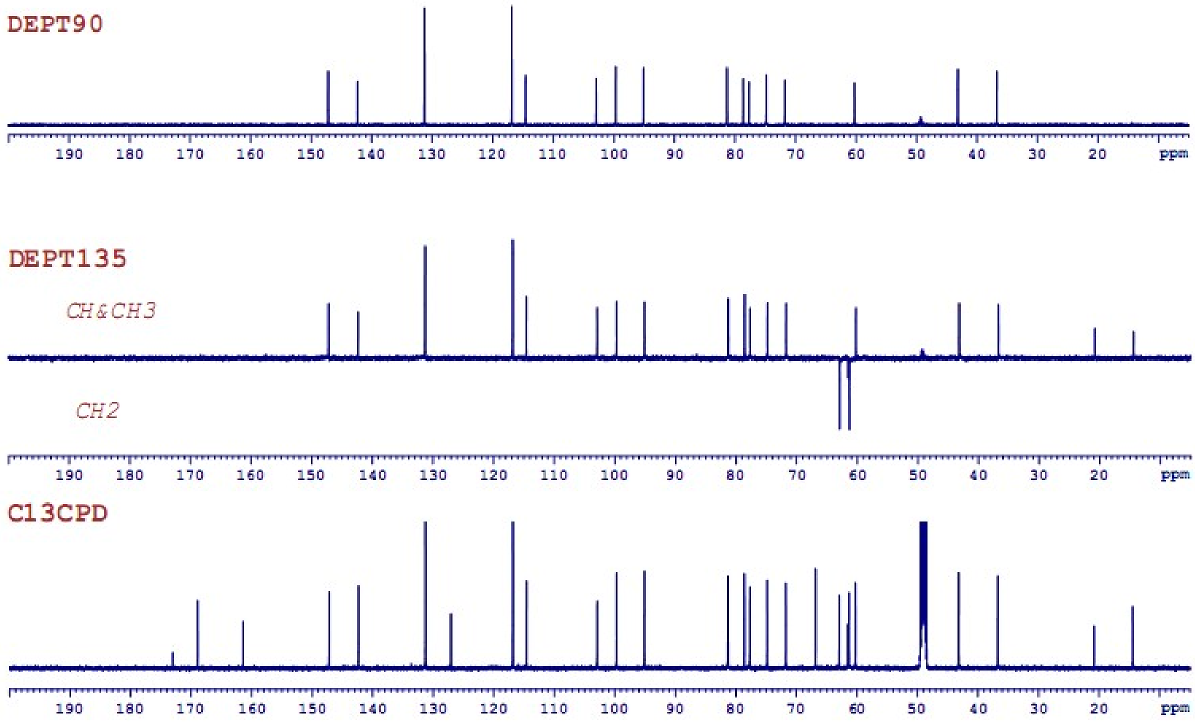


DEPT spectrum of compound **14**

## 1.15. Compound 15

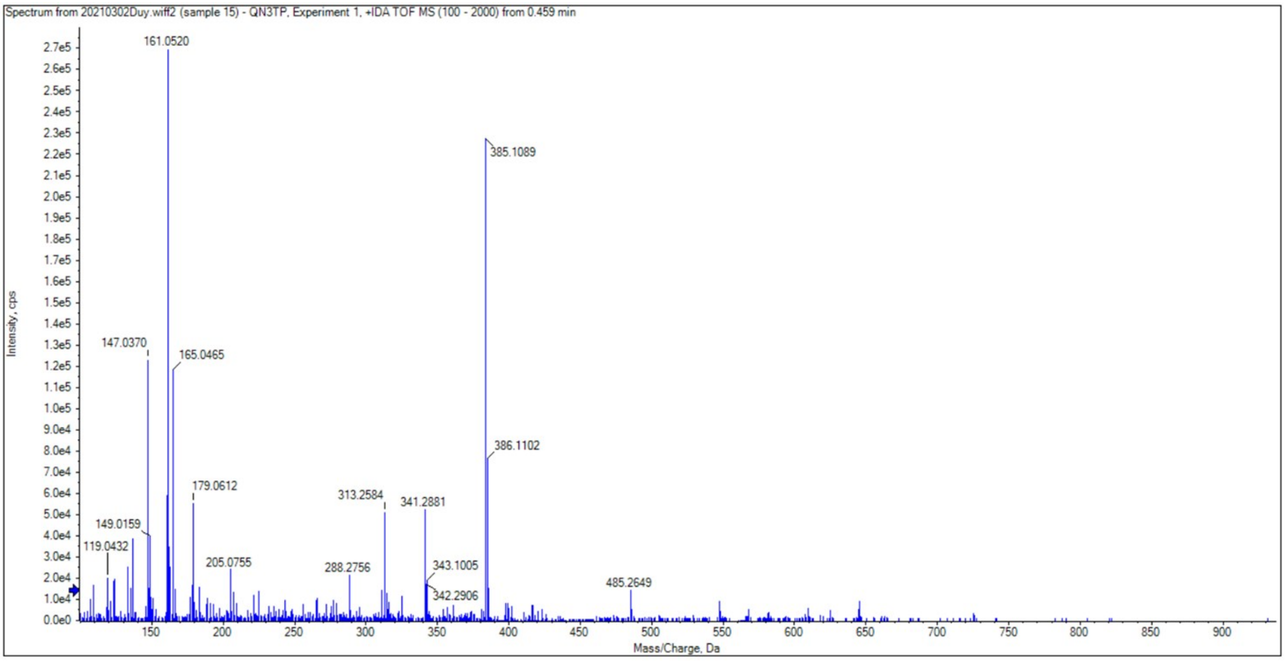


HR-ESI-MS spectrum of compound **15**

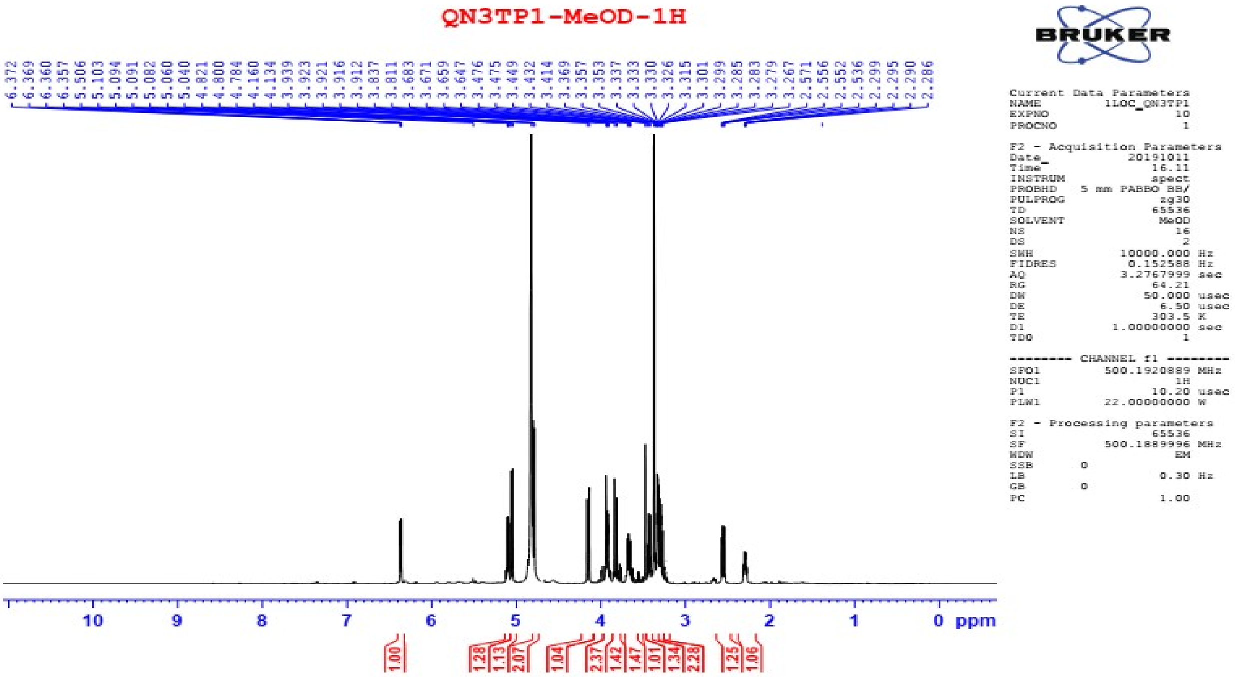


$^1$H-NMR spectrum of compound **15**

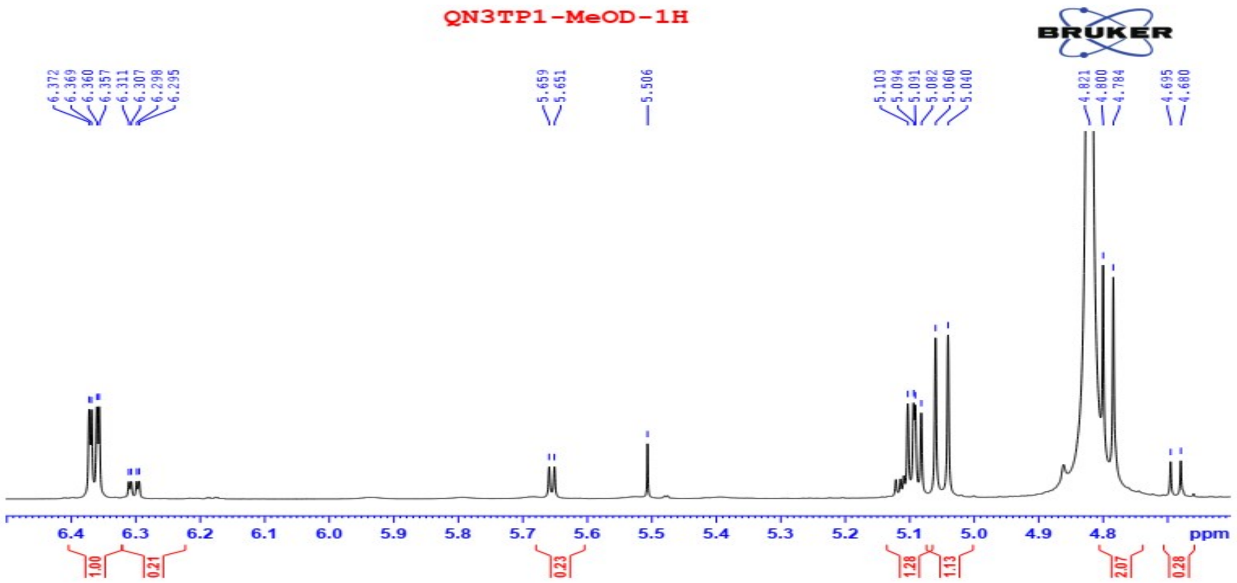


$^{1}$H-NMR spectrum (epansion) of compound **15**

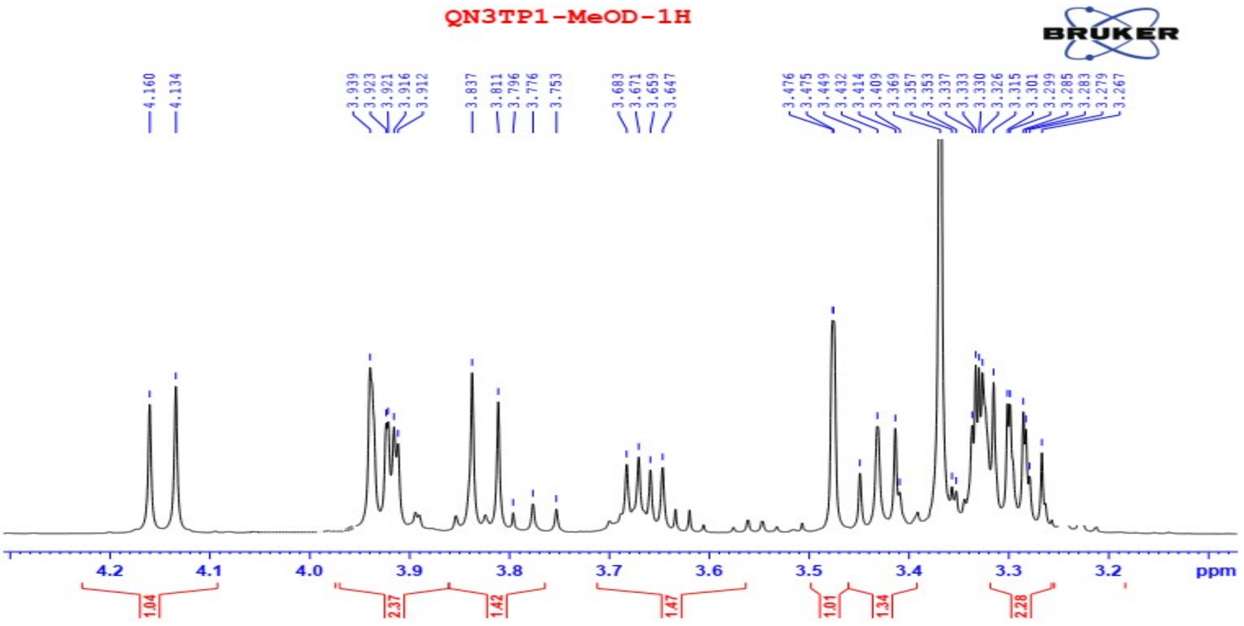


$^{1}$H-NMR spectrum (expansion) of compound **15**

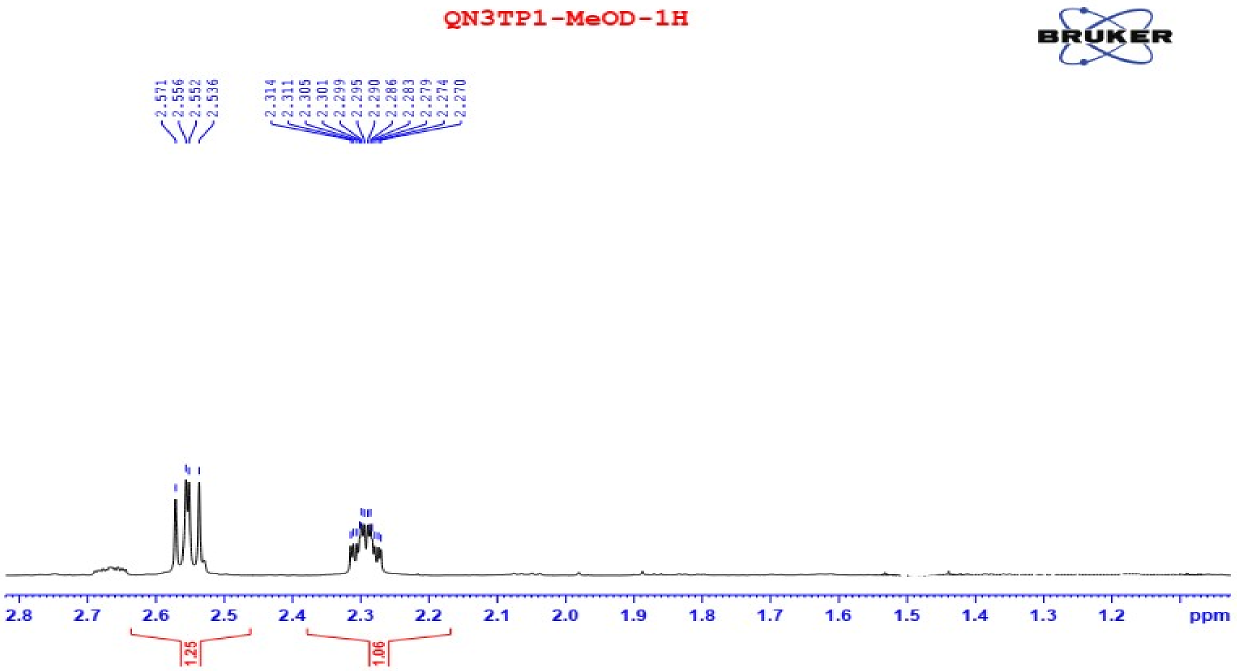


$^{1}$H-NMR spectrum (epansion) of compound **15**

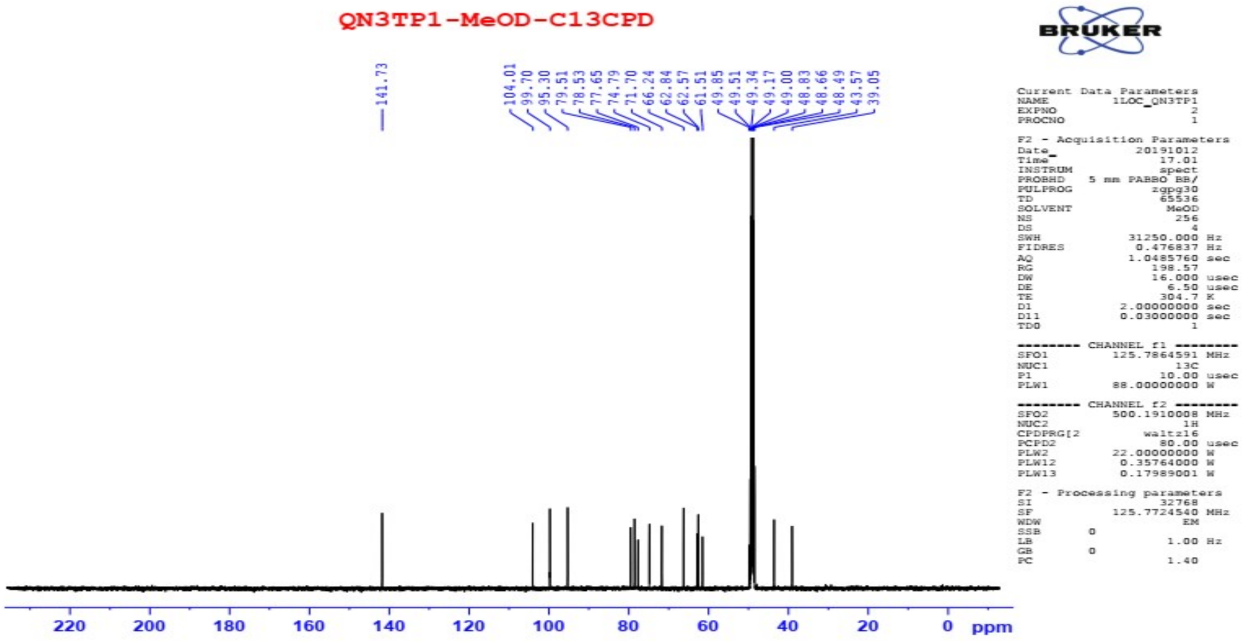


$^{13}$C-NMR spectrum of compound **15**

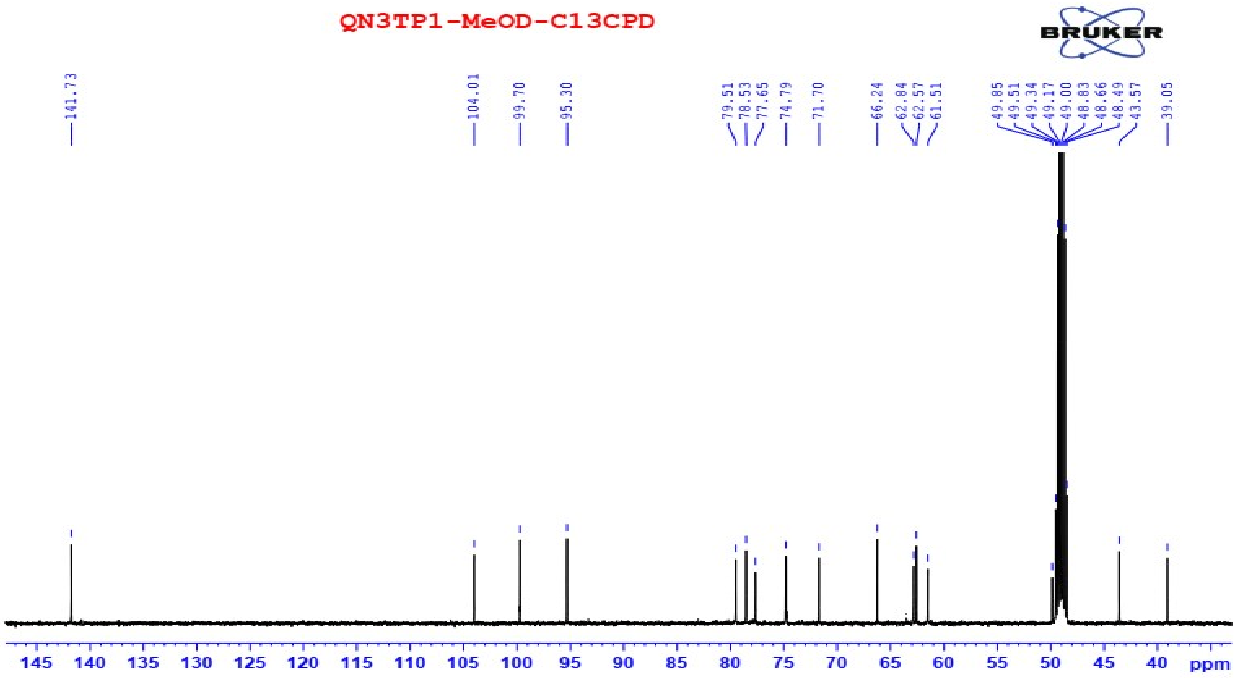


$^{13}$C-NMR spectrum (expansion) of compound **15**

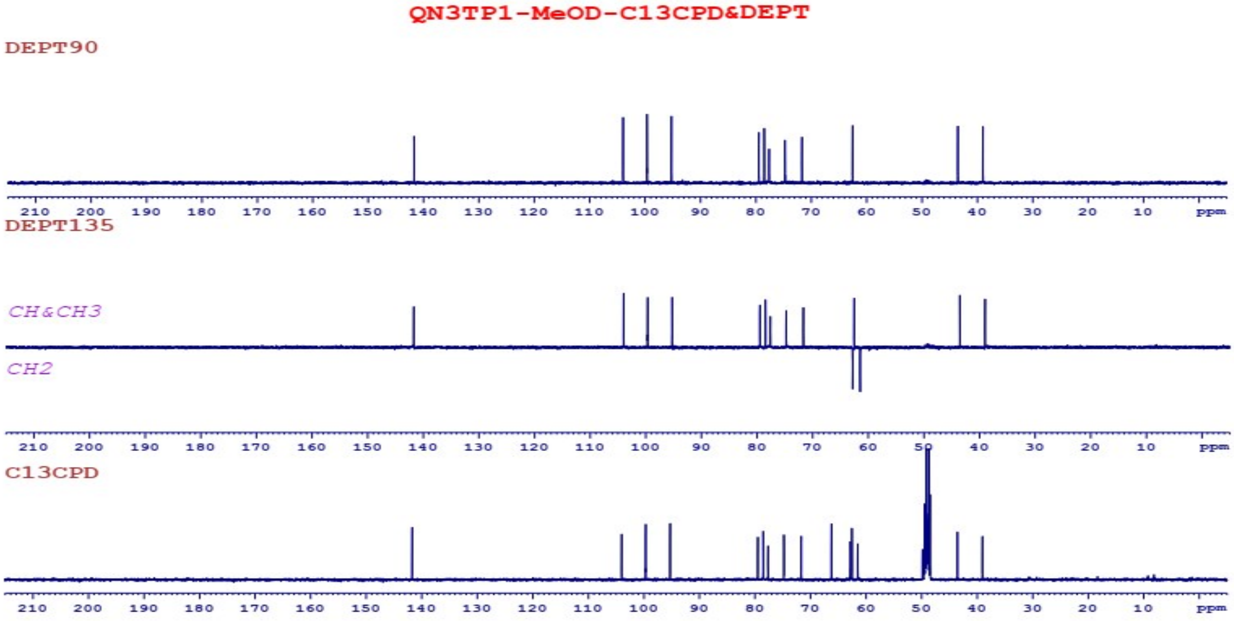


DEPT spectrum of compound **15**

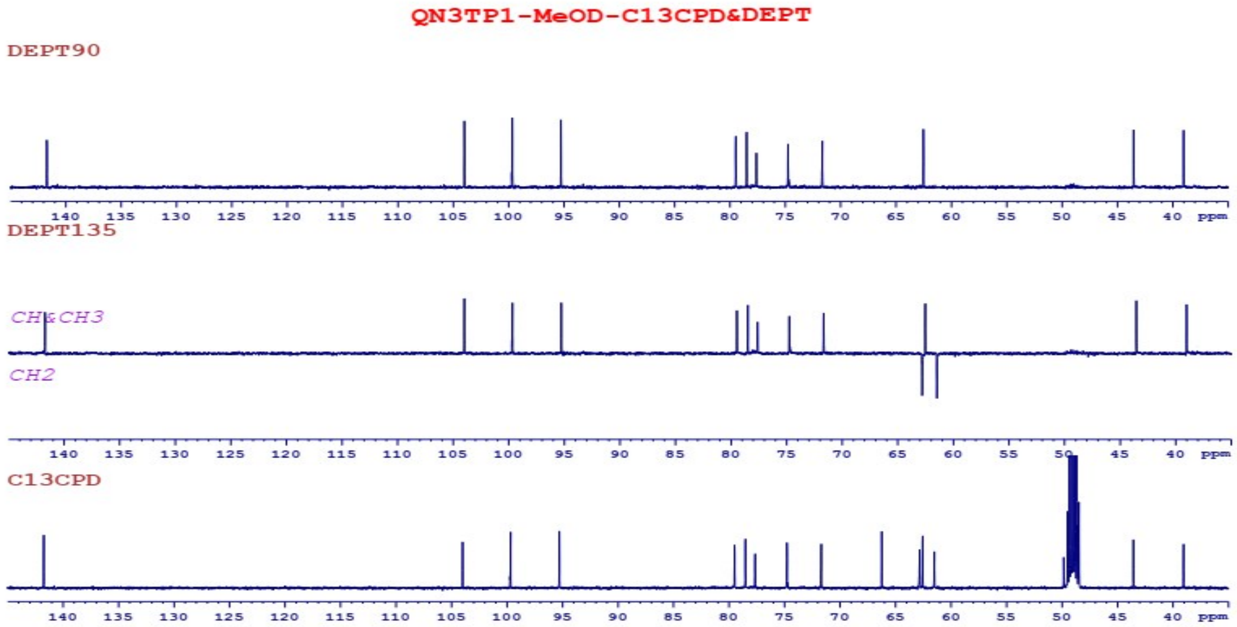


DEPT spectrum (epansion) of compound **15**

## 2. COMPUTATIONAL VIRTUAL RENDERINGS

### 2.1. Visual presentation and in-pose interaction map of ligand-3W37 inhibitory complexes

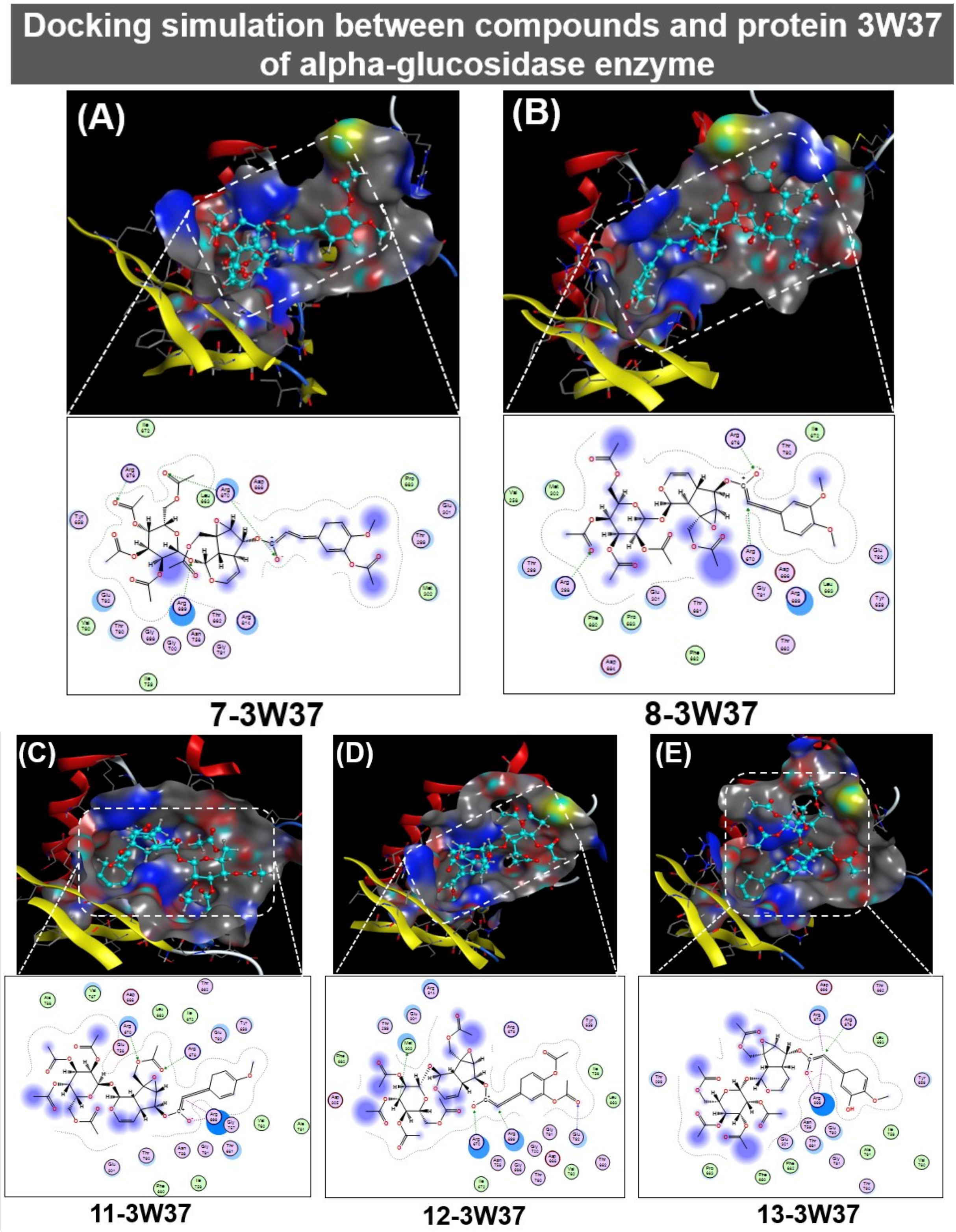

**2.2. Visual presentation and in-pose interaction map of ligand-3AJ7 inhibitory complexes**

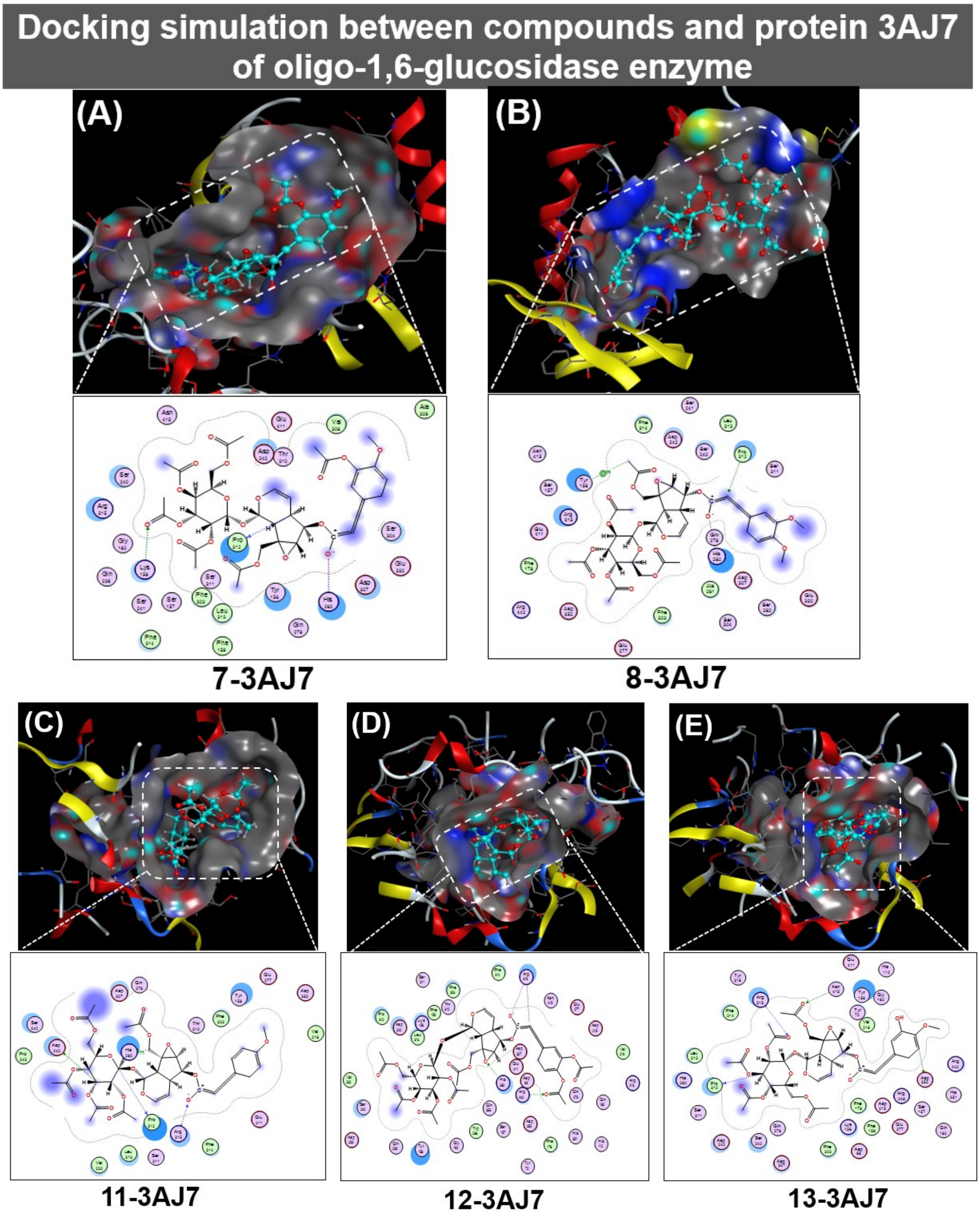

**2.3. Descriptive denotation for in-pose interactions projected by MOE 2015.10 molecular docking simulation**

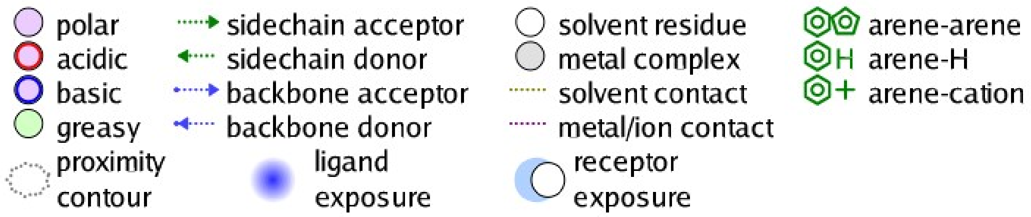

### 2.4. Molecular docking simulation results for inhibitory complexes

**Table 1.** Molecular docking simulation results for inhibitory complexes with docking score energy (DS), root-mean-square deviation (RMSD); critical interactions between the complexes and the protein 3W37 with amino acids: **1-3W37**, **2-3W37**, **3-3W37**, **4-3W37**, **5-3W37**, **6-3W37**, **7-3W37**, **8-3W37**, **9-3W37**, **10-3W37**, **11-3W37**, **12-3W37**, **13-3W37**, **14-3W37**, and **15-3W37**

| **Ligand-protein complex** | | | **Hydrogen bond** | | | | | | **van de Waals interaction** |
|---|---|---|---|---|---|---|---|---|---|
| **Name** | **DS** | **RMSD** | **L** | **P** | | **T** | **D** | **E** | |
| **1-3W37** | -14.1 | 1.47 | O | O | Ile 759 | H-donor | 3.23 | -1.4 | Thr 299, Glu 301, Ile 672, Tyr 659, Val 760, Thr 790, Leu 663, Gly 791, Thr 662, Asn 758, Asp 666, Gly 698, Gly 700, Arg 676 |
| | | | C | S | Met 302 | H-donor | 3.79 | -1.0 | |
| | | | O | N | Arg 699 | H-acceptor | 2.75 | -2.7 | |
| | | | O | N | Arg 699 | H-acceptor | 3.39 | -0.9 | |
| | | | O | N | Glu 792 | H-acceptor | 2.95 | -1.6 | |
| | | | C | N | Arg 670 | H-acceptor | 3.80 | -8.7 | |
| | | | C | N | Arg 670 | ionic | 3.80 | -0.9 | |
| **2-3W37** | -15.1 | 1.96 | O | N | Arg 814 | H-acceptor | 3.13 | -2.0 | Leu 663, Thr 662, Tyr 659, Glu 792, Phe 680, Val 760, Thr 790, Gly 791, Ile 759, Thr 681, Gly 698, Asn 758, Ile 672, Glu 301 |
| | | | C | N | Arg 670 | H-acceptor | 2.87 | -8.1 | |
| | | | O | N | Arg 670 | H-acceptor | 2.90 | -1.7 | |
| | | | O | N | Arg 676 | H-acceptor | 3.11 | -3.2 | |
| | | | C | N | Arg 699 | ionic | 3.17 | -3.4 | |
| | | | C | N | Arg 699 | ionic | 3.95 | -0.6 | |
| | | | C | O | Asp 666 | ionic | 3.51 | -1.9 | |
| | | | C | N | Arg 670 | ionic | 2.87 | -5.4 | |
| | | | C | N | Arg 670 | ionic | 3.55 | -1.7 | |
| | | | O | N | Arg 670 | ionic | 2.90 | -5.2 | |
| | | | O | N | Arg 676 | ionic | 3.43 | -2.2 | |
| | | | O | N | Arg 676 | ionic | 3.11 | -3.8 | |
| **3-3W37** | -12.9 | 1.33 | C | O | Asp 666 | H-donor | 3.34 | -0.7 | Leu 663, Tyr 659, Ile 759, Thr 681, Gly 700, Gly 698, Gly 791, Glu 792, Thr 790, Arg 670, Phe 680, Pro 683, Glu 301, Thr 299 |
| | | | O | N | Arg 814 | H-acceptor | 3.02 | -2.8 | |
| | | | O | N | Arg 699 | H-acceptor | 3.12 | -1.7 | |
| | | | O | N | Arg 676 | H-acceptor | 3.20 | -0.9 | |
| **4-3W37** | -12.3 | 1.72 | O | O | Asp 666 | H-donor | 3.22 | -1.2 | Thr 790, Gly 791, Arg 670, Glu 792, Tyr 659, Leu 663, Arg 676, Thr 681, Glu 301, Ile 672, Phe 680 |
| | | | O | O | Asp 666 | H-donor | 2.79 | -1.9 | |
| | | | O | N | Arg 814 | H-acceptor | 3.22 | -0.9 | |
| | | | O | N | Arg 814 | H-acceptor | 2.93 | -3.4 | |
| | | | O | N | Arg 699 | H-acceptor | 2.93 | -1.2 | |
| | | | 6-ring | N | Arg 699 | H-acceptor | 4.65 | -1.2 | |
| **5-3W37** | -11.6 | 1.40 | O | N | Arg 699 | H-acceptor | 2.92 | -3.3 | Phe 680, Asn 758, Arg 814, Phe 816, Thr 681, Ile 759, Phe 815, Leu 663, Gly 791, Thr 662, Asp 666, Tyr 659, Arg 670, Ile 672, Thr 790, |
| | | | O | N | Glu 792 | H-acceptor | 2.90 | -1.4 | |

| | | | | | | | | | |
|---|---|---|---|---|---|---|---|---|---|
| | | | O | N | Arg 676 | H-acceptor | 2.90 | -5.1 | |
| **6-3W37** | - | - | - | - | - | - | - | - | - |
| **7-3W37** | -8.1 | 1.84 | O | N | Arg 699 | H-acceptor | 2.64 | -2.8 | Ile 659, Arg 814, Gly 791, Asn 758, Gly 700, Gly 698, Thr 790, Val 760, Glu 792, Tyr 659, Leu 663, Asp 666, Pro 683, Glu 301, Thr 299, Met 302 |
| | | | O | N | Arg 670 | H-acceptor | 2.93 | -1.0 | |
| | | | O | N | Arg 676 | H-acceptor | 2.67 | -0.8 | |
| | | | O | N | Arg 670 | H-acceptor | 2.74 | -5.6 | |
| | | | O | N | Arg 670 | ionic | 3.94 | -0.6 | |
| | | | O | N | Arg 670 | ionic | 2.93 | -4.9 | |
| **8-3W37** | -10.5 | 1.76 | C | N | Arg 670 | H-acceptor | 3.48 | -1.0 | Glu 792, Tyr 659, Leu 663, Arg 699, Asp 666, Gly 791, Thr 662, Thr 681, Phe 682. Glu 301, Pro 683, Asp 684, Phe 680, Thr 299, Val 258, Met 302, Thr 790, Ile 672 |
| | | | O | N | Arg 676 | H-acceptor | 2.90 | -0.9 | |
| | | | O | N | Arg 298 | H-acceptor | 3.18 | -1.5 | |
| | | | C | N | Arg 670 | ionic | 3.24 | -3.0 | |
| | | | C | N | Arg 670 | ionic | 3.48 | -2.0 | |
| | | | O | N | Arg 676 | ionic | 2.90 | -5.2 | |
| **9-3W37** | -13.3 | 1.69 | O | O | Glu 792 | H-donor | 2.88 | -1.3 | Tyr 659, Ile 759, Gly 791, Leu 663, Thr 662, Asp 666, Glu 301, Met 302, Thr 299 |
| | | | O | N | Arg 699 | H-acceptor | 3.10 | -2.0 | |
| | | | O | N | Arg 670 | H-acceptor | 3.25 | -0.8 | |
| | | | C | N | Arg 670 | ionic | 3.46 | -2.0 | |
| | | | O | N | Arg 670 | ionic | 3.91 | -0.7 | |
| | | | O | N | Arg 670 | ionic | 2.81 | -5.9 | |
| **10-3W37** | -14.7 | 1.52 | O | O | Asp 666 | H-donor | 3.21 | -0.8 | Glu 301, Phe 680, Met 302, Leu 663, Gly 791, Thr 662, Thr 790, Glu 792, Arg 699, Ile 672, Arg 676, Tyr 659, Gly 698, Arg 814 |
| | | | O | O | Asp 666 | H-donor | 2.71 | -1.1 | |
| | | | O | N | Arg 670 | H-acceptor | 3.18 | -0.3 | |
| | | | O | O | Thr 299 | H-acceptor | 2.95 | -1.2 | |
| | | | C | N | Arg 670 | ionic | 3.24 | -3.1 | |
| | | | O | N | Arg 670 | ionic | 3.30 | -2.8 | |
| | | | O | N | Arg 670 | ionic | 3.30 | -2.6 | |
| **11-3W37** | -10.1 | 1.46 | O | N | Arg 670 | H-acceptor | 2.92 | -1.5 | Ala 788, Val 787, Asp 666, Leu 663, Ile 672, Thr 662, Glu 792, Tyr 659, Gly 757, Val 760, Ala 761, Thr 681, Gly 791, Asn 758, Thr 790, Glu 301, Phe 680, Ile 759, Glu 765 |
| | | | O | N | Arg 676 | H-acceptor | 2.96 | -2.3 | |
| | | | C | N | Arg 699 | ionic | 3.15 | -3.6 | |
| | | | O | N | Arg 699 | ionic | 3.92 | -0.7 | |
| **12-3W37** | -7.8 | 1.41 | C | S | Met 302 | H-donor | 4.12 | -1.0 | Asp 305, Phe 680, Thr 299, Glu 301, Arg 814, Arg 676, Tyr 659, Ile 759, Leu 663, Thr 662, Val 760, Asp 666, Gly 791, Gly 700, Thr 790, Gly 698, Asn 758, Ile 672 |
| | | | C | N | Arg 699 | H-acceptor | 3.35 | -7.9 | |
| | | | O | N | Arg 670 | H-acceptor | 2.78 | -4.8 | |
| | | | O | N | Glu 792 | H-acceptor | 3.05 | -1.3 | |
| | | | C | N | Arg 699 | ionic | 3.35 | -2.5 | |
| | | | O | N | Arg 670 | ionic | 3.12 | -3.7 | |
| | | | O | N | Arg 670 | ionic | 2.78 | -6.1 | |
| **13-3W37** | -8.8 | 1.47 | O | N | Arg 670 | H-acceptor | 3.16 | -1.1 | Glu 301, Tyr 659, Val 760, Asn 758, Thr 662, Glu 792, Ala 761, Leu 663, Ile 759, Gly 791, Thr 681, Asp 666, Ile 672, Phe 680, Thr 299, Asp 305, Met 302, Pro 683 |
| | | | O | N | Arg 676 | H-acceptor | 2.98 | -4.8 | |
| | | | C | N | Arg 670 | ionic | 3.84 | -0.8 | |
| | | | C | N | Arg 699 | ionic | 3.42 | -2.2 | |
| | | | O | N | Arg 670 | ionic | 3.58 | -1.6 | |
| | | | O | N | Arg 676 | ionic | 2.98 | -4.6 | |
| | | | O | N | Arg 676 | ionic | 3.70 | -1.2 | |

| | | | | | | | | | |
|---|---|---|---|---|---|---|---|---|---|
| **14-3W37** | -13.0 | 1.01 | C | N | Arg 676 | H-acceptor | 3.74 | -6.0 | Glu 301, Ile 672, Leu 663, Arg 670, Tyr 659, Glu 792, Thr 662, Gly 791, Thr 790, Phe 815, Phe 816, Arg 814, Thr 299 |
| | | | C | O | Asp 666 | ionic | 2.94 | -4.9 | |
| | | | C | N | Arg 676 | ionic | 3.74 | -1.1 | |
| | | | C | N | Arg 699 | ionic | 3.90 | -0.7 | |
| **15-3W37** | -12.3 | 0.67 | O | O | Asp 666 | H-donor | 3.26 | -1.0 | Gly 698, Tyr 659, Leu 663. Gly 791, Thr 790, Ile 759, Arg 670, Glu 792, Ile 672 |
| | | | O | N | Arg 699 | H-acceptor | 3.22 | -2.0 | |
| | | | O | N | Arg 814 | H-acceptor | 3.17 | -0.6 | |
| | | | O | N | Arg 699 | H-acceptor | 2.99 | -2.5 | |
| | | | O | N | Arg 676 | H-acceptor | 2.97 | -3.4 | |
| **DS**: Docking score energy (kcal.mol$^{-1}$); **RMSD**: Root-mean-square deviation (Å) | | | | | | | | | |
| **L**: Ligand; **P**: Protein; **T**: Type; **D**: Distance (Å); **E**: Energy (kcal.mol$^{-1}$); | | | | | | | | | |

**Table 2.** Molecular docking simulation results for inhibitory complexes with docking score energy (DS), root-mean-square deviation (RMSD); critical interactions between the complexes and the protein 3AJ7 with amino acids: **1-3AJ7**, **2-3AJ7**, **3-3AJ7**, **4-3AJ7**, **5-3AJ7**, **6-3AJ7**, **7-3AJ7**, **8-3AJ7**, **9-3AJ7**, **10-3AJ7**, **11-3AJ7**, **12-3AJ7**, **13-3AJ7**, **14-3AJ7**, and **15-3AJ7**

| **Ligand-protein complex** | | | **Hydrogen bond** | | | | | | **van der Waals interaction** |
|---|---|---|---|---|---|---|---|---|---|
| **Name** | **DS** | **RMSD** | **L** | **P** | | **T** | **D** | **E** | |
| **1-3AJ7** | -11.4 | 1.14 | C | O | Glu 277 | H-donor | 2.99 | -0.8 | Val 216, His 112, Gln 182, Tyr 72, Arg 442, Phe 176, His 351, Phe 303, Phe 159, Gln 353, Glu 411, Gln 279, Tyr 158, Phe 314, Ser 240, Arg 315, Pro 312, Leu 313, Asp 242, Val 232 |
| | | | C | O | Glu 277 | H-donor | 3.36 | -0.7 | |
| | | | O | O | Asp 215 | H-donor | 2.67 | -3.1 | |
| | | | O | O | Asp 352 | H-donor | 2.58 | -3.7 | |
| | | | O | O | Asp 352 | H-donor | 2.91 | -0.8 | |
| | | | O | O | Asp 69 | H-donor | 2.83 | -3.1 | |
| | | | O | N | Arg 213 | H-acceptor | 3.20 | -1.7 | |
| **2-3AJ7** | -13.5 | 1.64 | O | O | Glu 411 | H-donor | 2.63 | -2.4 | Val 216, Asp 215, Arg 442, Phe 178, Phe 159, Phe 303, Tyr 316, Pro 312, Phe 314, Asp 307, Ser 240, Ser 241, Asp 242, Lys 156, Leu 313 |
| | | | C | O | Asp 353 | H-donor | 3.24 | -0.7 | |
| | | | O | O | Glu 277 | H-donor | 2.61 | -3.5 | |
| | | | O | O | Glu 277 | H-donor | 3.11 | -1.8 | |
| | | | O | O | Glu 277 | H-donor | 2.88 | -1.7 | |
| | | | O | O | Glu 279 | H-donor | 2.58 | -14.8 | |
| | | | C | C | Tyr 158 | H-aceptor | 4.00 | -1.0 | |
| | | | O | C | Arg 315 | H-aceptor | 3.22 | -0.8 | |
| | | | C | N | His 280 | Ionic | 3.45 | -2.1 | |
| **3-3AJ7** | -10.6 | 1.71 | O | O | Glu 411 | H-donor | 2.74 | -2.9 | Gln 182, Val 216, Phe 303, Arg 442, His 112, His 351. Arg 446, Tyr 158, Phe 178, Phe 159, Arg 315, Gln 279, Asp 307, Pro 312, Asp 242, His 280 |
| | | | C | O | Asp 69 | H-donor | 3.35 | -0.7 | |
| | | | O | O | Asp 352 | H-donor | 2.99 | -2.3 | |
| | | | O | O | Asp 215 | H-donor | 2.71 | -4.4 | |
| | | | O | O | Glu 277 | H-donor | 2.57 | -2.7 | |
| | | | O | O | Asp 69 | H-donor | 2.80 | -4.8 | |
| | | | O | N | Arg 213 | H-acceptor | 3.24 | -1.4 | |
| | | | C | 6-ring | Tyr 72 | H-π | 4.10 | -0.6 | |
| **4-3AJ7** | -10.2 | 1.79 | O | O | Pro 312 | H-donor | 2.99 | -1.1 | Lys 156, Glu 411, Phe 159, Gln 279, Ser 157, Gly 160, His 280, Ser 311, Phe 314, Tyr 316, Ser 240, Thr 310, Asp 242, Asp 307, Ser 304 |
| | | | O | O | Asn 415 | H-donor | 2.79 | -2.1 | |
| | | | O | O | Leu 313 | H-donor | 2.88 | -1.3 | |
| | | | O | O | Tyr 158 | H-donor | 2.96 | -2.0 | |
| | | | O | N | Arg 315 | H-acceptor | 3.09 | -2.3 | |
| | | | O | B | Asn 415 | H-acceptor | 2.89 | -1.1 | |
| **5-3AJ7** | -11.7 | 1.80 | O | C | His 280 | H-acceptor | 3.32 | -0.7 | Gln 239, Ser 157, Phe 314, Glu 160, Tyr 158, Phe 159, Glu 411, Phe 303, Arg 315, Leu 313, Pro 312, Ser 240, Asp 242, Thr 245, Ser 311, Gln 279, Tyr 316, Asp 307, Ser 304, Thr 310, Val 308, Gly 309 |
| | | | O | N | Lys 156 | H-acceptor | 2.56 | -0.2 | |
| | | | O | N | Asn 415 | H-acceptor | 3.11 | -2.1 | |
| | | | O | N | Ser 241 | H-acceptor | 3.00 | -2.8 | |

| | | | | | | | | | |
|---|---|---|---|---|---|---|---|---|---|
| **6-3AJ7** | - | - | - | - | - | - | - | - | - |
| **7-3AJ7** | -9.3 | 1.98 | C | O | Pro 312 | H-donor | 3.31 | -0.7 | Asn 415, Ser 240, Arg 315, Gly 160, Gln 239, Ser 241, Phe 314, Ser 157, Ser 311, Phe 303, Leu 313, Phe 159, Tyr 158, Gln 279, Asp 242, Thr 310, Glu 411, Val 308, Asp 307, Glu 332, Ser 304, Ala 329 |
| | | | O | N | Lys 156 | H-acceptor | 3.27 | -2.6 | |
| | | | O | N | His 280 | ionic | 3.53 | -1.8 | |
| **8-3AJ7** | -8.8 | 1.90 | C | C | Pro 312 | H-acceptor | 4.19 | -0.8 | Arg 442, Phe 178, Glu 411, Arg 315, Ser 157, Asn 415, Asp 352, Glu 277, Phe 303, Ala 281, Ser 304, Asp 307, Ser 282, Glu 332, Phe 314, Asp 242, Ser 240, Ser 241, Leu 313, Ser 311 |
| | | | O | N | His 280 | ionic | 3.19 | -3.3 | |
| | | | O | N | His 280 | ionic | 3.19 | -3.3 | |
| | | | C | 6-ring | Tyr 158 | H-π | 4.03 | -1.2 | |
| **9-3AJ7** | -11.5 | 1.74 | O | O | Glu 411 | H-donor | 2.77 | -5.1 | Val 216, Phe 178, Asp 362, Glu 277, Arg 442, Phe 314, Tyr 158, Leu 313, Asp 242, Pro 312, Thr 310, Ser 304, Asp 307, Gln 279, Phe 303, Phe 159, Asp 215 |
| | | | O | N | Arg 315 | H-acceptor | 2.95 | -2.6 | |
| | | | C | N | His 280 | ionic | 3.68 | -1.3 | |
| | | | O | N | His 280 | ionic | 3.06 | -4.1 | |
| **10-3AJ7** | -12.4 | 1.33 | O | O | Asp 242 | H-donor | 3.07 | -1.2 | Arg 446, Gln 353, Glu 411, His 351, Arg 442, Asp 352, Asp 69, Val 216, Asp 215, Glu 277, Phe 178, Phe 159, Lys 156, Tyr 158, Pro 312, Ser 311, Phe 303, Ser 240, Phe 314, Gln 279, Asp 307 |
| | | | O | O | Ser 157 | H-donor | 2.79 | -1.2 | |
| | | | O | O | Leu 313 | H-donor | 2.77 | -1.1 | |
| | | | O | C | His 280 | H-acceptor | 3.05 | -0.8 | |
| | | | O | N | Ser 241 | H-acceptor | 3.04 | -0.6 | |
| | | | O | N | Arg 315 | ionic | 3.52 | -1.8 | |
| | | | O | 6-ring | Tyr 72 | H-π | 4.08 | -0.8 | |
| **11-3AJ7** | -6.3 | 1.99 | C | O | Pro 312 | H-donor | 3.02 | -0.9 | Pro 243, Ser 240, Val 232, Leu 313, Ser 311, Phe 314, Glu 411, Val 216, Asp 252, Glu 277, Tyr 158, Phe 303, Thr 310, Gln 279, Asp 307 |
| | | | C | O | Asp 242 | H-donor | 3.14 | -1.0 | |
| | | | O | N | Arg 315 | H-acceptor | 3.05 | -1.7 | |
| | | | C | 5-ring | His 280 | H-π | 4.60 | -0.6 | |
| **12-3AJ7** | -6.4 | 1.06 | O | C | His 280 | H-acceptor | 3.18 | -1.8 | Val 232, Ser 240, Asp 233, Gln 239, Tyr 158, Gly 160, Trp 238, Ser 157, Tyr 72, Asp 352, Phe 178, His 351, Gln 353, His 112, Gln 182, Arg 213, Val 216, Asp 215, Glu 277, Asn 415, Asp 69, Glu 411, Phe 314, Phe 303, Thr 310, Phe 159, Ser 311, Lys 156, Leu 313, Asp 242, Pro 312, Gln 279 |
| | | | O | N | Arg 442 | H-acceptor | 2.99 | -4.3 | |
| | | | C | N | Arg 315 | ionic | 3.56 | -1.7 | |
| | | | O | N | Arg 315 | ionic | 3.25 | -3.0 | |
| | | | O | N | Arg 315 | ionic | 3.42 | -2.2 | |
| | | | O | N | Arg 315 | ionic | 3.86 | -0.8 | |
| **13-3AJ7** | -7.5 | 1.99 | C | O | Pro 312 | H-donor | 3.18 | -1.6 | Tyr 316, Phe 314, Leu 313, His 380, Ser 311, Asp 242, Ser 240, Gln 279, Asp 307, Phe 303, Lys 156, Asp 69, Phe 178, Phe 159, Asp 215, Glu 277, Arg 446, Ser 157, Gln 182, His 315, Arg 442, Val 216, Tyr 72, Tyr 158, Gly 160, His 112, Glu 411 |
| | | | C | O | Asp 352 | H-donor | 3.30 | -0.7 | |
| | | | O | N | Asn 415 | H-acceptor | 2.94 | -1.0 | |
| | | | O | N | Arg 315 | H-acceptor | 3.29 | -1.5 | |
| **14-3AJ7** | -14.5 | 1.71 | C | O | Asp 242 | H-donor | 3.54 | -0.7 | Glu 411, Asp 215, Val 216, Phe 159, Tyr 158, Arg 315, Phe 178, Phe 303, Thr 310, Ser 240, Arg |
| | | | C | O | Glu 277 | ionic | 3.82 | -0.9 | |

| | | | | | | | | | |
|---|---|---|---|---|---|---|---|---|---|
| | | | C | O | Glu 277 | ionic | 2.93 | -5.0 | 213, Val 232, Leu 246, Leu 313, Asp 307, Pro 312, Gln 279, Ser 311, Phe 314, His 280 |
| | | | C | O | Asp 352 | ionic | 3.39 | -2.3 | |
| | | | C | O | Glu 277 | ionic | 3.77 | -1.0 | |
| **15-3AJ7** | -11.0 | 1.12 | O | O | Asp 242 | H-donor | 2.69 | -4.9 | Arg 315, Leu 313, Tyr 158, Lys 156, Ser 157, Gln 279, Ser 240, Ser 241, Phe 314, Pro 312 |
| | | | O | O | Asp 242 | H-donor | 2.77 | -2.6 | |
| | | | O | C | His 280 | H-acceptor | 3.18 | -1.0 | |
| **DS**: Docking score energy ($kcal.mol^{-1}$); **RMSD**: Root-mean-square deviation (Å) | | | | | | | | | |
| **L**: Ligand; **P**: Protein; **T**: Type; **D**: Distance (Å); **E**: Energy ($kcal.mol^{-1}$) | | | | | | | | | |

**Table 3.** Molecular docking simulation results for controlled drugs as inhibitory complexes with docking score energy (DS), root-mean-square deviation (RMSD); critical interactions between the complexes and the two protein 3W37, 3AJ7 with amino acids: **D1-3W37**, **D2-3W37**, **D3-3W37**; **D1-3AJ7**, **D2-3AJ7**, and **D3-3AJ7**

| **Ligand-protein complex** | | | **Hydrogen bond** | | | | | | **van der Waals interaction** |
|---|---|---|---|---|---|---|---|---|---|
| **Name** | **DS** | **RMSD** | **L** | **P** | | **T** | **D** | **E** | |
| **D1-3W37** | -14.2 | 1.58 | O | O | Asp 666 | H-donor | 2.70 | -2.1 | Phe 861, Gly 791, Phe 815, Leu 663, Tyr 659, Pro 300, Thr 681, Gly 698, Phe 680, Pro 683, Thr 299, Ile 672, Arg 670, Thr 790 |
| | | | O | O | Glu 792 | H-donor | 2.77 | -1.9 | |
| | | | O | O | Glu 301 | H-donor | 2.89 | -1.4 | |
| | | | O | N | Arg 699 | H-acceptor | 2.94 | -1.5 | |
| | | | O | N | Arg 699 | H-acceptor | 3.54 | -0.6 | |
| | | | O | N | Arg 676 | H-acceptor | 3.52 | -0.6 | |
| | | | O | N | Arg 814 | H-acceptor | 2.99 | -1.6 | |
| | | | O | N | Arg 699 | H-acceptor | 2.94 | -2.2 | |
| **D2-3W37** | -11.1 | 0.68 | O | O | Asp 666 | H-donor | 2.77 | -3.6 | Gly 791, Glu 792, Thr 662, Thr 790, Leu 663, Ile 759, Tyr 659, Ile 672 |
| | | | O | O | Asp 666 | H-donor | 2.76 | -3.3 | |
| | | | O | N | Arg 676 | H-acceptor | 2.84 | -4.2 | |
| | | | O | N | Arg 670 | H-acceptor | 2.92 | -2.0 | |
| | | | O | N | Arg 699 | H-acceptor | 3.23 | -2.2 | |
| **D3-3W37** | -12.7 | 1.52 | O | O | Asp 666 | H-donor | 2.83 | -2.3 | Val 760, Leu 663, Thr 790, Asn 758, Gly 791, Arg 670, Ile 672, Tyr 659, Gly 698, Ile 701 |
| | | | O | O | Glu 792 | H-donor | 2.91 | -1.5 | |
| | | | O | O | Ile 759 | H-donor | 2.68 | -2.2 | |
| | | | O | N | Arg 699 | H-acceptor | 2.80 | -0.9 | |
| | | | O | N | Arg 676 | H-acceptor | 3.30 | -1.7 | |
| | | | O | N | Gly 700 | H-acceptor | 2.76 | -0.8 | |
| **D1-3AJ7** | -15.3 | 1.79 | O | O | Glu 411 | H-donor | 2.88 | -1.5 | Lys 156, Ser 157, Phe 159, Gln 279, Leu 313, Arg 442, Gln 353, Asp 215, Asp 352, Phe 178, Arg 213, Val 216, Arg 315, Phe 303, Thr 310, Phe 314, Ser 311, Pro 312, Ser 241, Ser 240 |
| | | | O | O | Asp 307 | H-donor | 3.27 | -1.0 | |
| | | | O | O | Glu 277 | H-donor | 2.61 | -1.3 | |
| | | | O | O | Glu 277 | H-donor | 3.12 | -1.2 | |
| | | | O | O | Glu 277 | H-donor | 2.63 | -3.4 | |
| | | | O | O | Asp 242 | H-donor | 2.96 | -1.9 | |
| | | | O | O | Asp 69 | H-donor | 2.96 | -2.9 | |
| | | | C | 6-ring | Tyr 158 | H-π | 3.95 | -0.7 | |
| | | | O | 5-ring | His 280 | H-π | 3.71 | -0.9 | |
| **D2-3AJ7** | -10.7 | 1.03 | O | O | Asp 233 | H-donor | 2.70 | -3.7 | Leu 313, Asn 317, Asn 235, Ile 419, Ser 236, Ala 418, His 423, Phe 433 |
| | | | O | O | Glu 422 | H-donor | 2.97 | -1.5 | |
| | | | O | O | Glu 429 | H-donor | 3.00 | -1.4 | |
| | | | O | N | Lys 156 | H-acceptor | 3.01 | -0.7 | |
| | | | C | 6-ring | Phe 314 | H-π | 3.68 | -1.0 | |
| **D3-3AJ7** | -13.6 | 0.94 | O | O | Glu 277 | H-donor | 2.82 | -1.7 | Glu 411, Tyr 158, Gln 353, His 351, Asp 69, Tyr 72, Val 216, Phe 303, Gln 279, Phe 159, Phe 178, Arg 315 |
| | | | O | O | Asp 352 | H-donor | 2.84 | -1.4 | |
| | | | O | O | Asp 215 | H-donor | 2.81 | -3.2 | |

| | | | | | | | | | |
|---|---|---|---|---|---|---|---|---|---|
| | | | N | O | Glu 277 | H-donor | 3.27 | -3.2 | |
| | | | N | O | Asp 352 | H-donor | 3.09 | -1.8 | |
| | | | C | O | Asp 352 | H-donor | 3.44 | -0.8 | |
| | | | O | N | Arg 442 | H-acceptor | 2.96 | -2.1 | |
| | | | O | N | Arg 213 | H-acceptor | 3.02 | 0.5 | |
| | | | N | O | Asp 215 | ionic | 3.93 | -0.6 | |
| | | | N | O | Glu 277 | ionic | 3.82 | -0.9 | |
| | | | N | O | Glu 277 | ionic | 3.27 | -2.9 | |
| | | | N | O | Asp 352 | ionic | 3.09 | -3.9 | |
| **DS**: Docking score energy (kcal.mol$^{-1}$); **RMSD**: Root-mean-square deviation (Å) | | | | | | | | | |
| **L**: Ligand; **P**: Protein; **T**: Type; **D**: Distance (Å); **E**: Energy (kcal.mol$^{-1}$) | | | | | | | | | |
